# Enantioenriched 1,4-Benzoxazepines via Chiral Brønsted Acid-Catalyzed Enantioselective Desymmetrization of 3-Substituted Oxetanes

**DOI:** 10.1021/acs.joc.3c01929

**Published:** 2023-11-21

**Authors:** Martin Nigríni, Viraj A. Bhosale, Ivana Císařová, Jan Veselý

**Affiliations:** † Department of Organic Chemistry, Faculty of Science, Charles University, Hlavova 2030/8, 128 43 Prague 2, Czech Republic; ‡ Department of Inorganic Chemistry, Faculty of Science, Charles University, Hlavova 2030/8, 128 43 Prague 2, Czech Republic

## Abstract

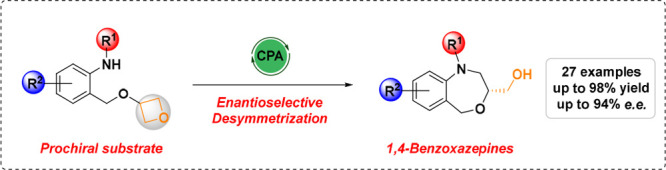

Herein, we present a highly enantioselective desymmetrization of 3-substituted oxetanes enabled by a confined chiral phosphoric acid. This metal-free process allows effective access to chiral seven-membered 1,4-benzoxazepines with a high degree of enantiocontrol, under mild reaction conditions. The developed synthetic strategy tolerates a broad substrate scope and demonstrates its synthetic utility in various enantioselective product transformations, thus proving its effectiveness in diverse scenarios.

## Introduction

**Figure 1:**
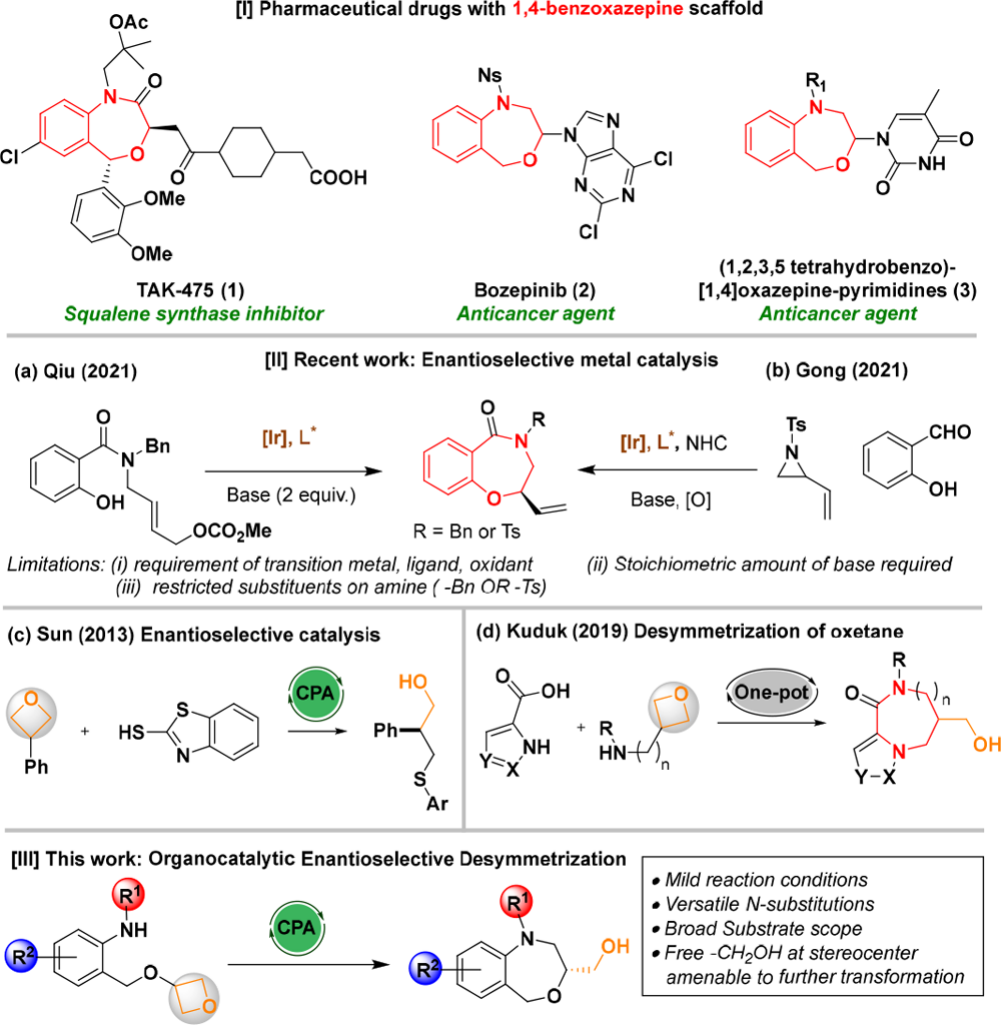
Promising pharmaceutical drugs with the seven-membered 1,4-BZOs scaffold, recent reports, and our work.

The seven-membered 1,4-benzoxazepine (1,4-BZOs) scaffold is a fascinating versatile pharmacophore that constitutes the integral backbone of a significant proportion of pharmaceutical drugs^1^ (Figure 1).

For example, the potent squalene synthase inhibitor TAK-475^1a^ is used to lower plasma cholesterol levels. It is noteworthy that bozepinib^1b^ containing a 1,4-BZOs scaffold showed high antiproliferative activity on human breast adenocarcinoma cancerous cell lines. In addition, the anticancer activity showed by (1,2,3,5-tetrahydro-4,1-benzoxazepine-3-yl)pyrimidines^1c^ highlights the importance of the seven-membered 1,4-BZOs scaffold as a potentially useful pharmacophore (Figure 1). The structural diversity coupled with the biological activity of 1,4-BZOs has attracted a great deal of interest, which has led to the development of several nonchiral synthetic methods.^2^ Exploiting catalytic asymmetric strategies for this seven-membered heterocycle offers a significant challenge and remains in its infancy.^3^ For instance, the Gallagher group^3a^ reported a single example of chiral 1,4-BZOs through nucleophilic cleavage of enantiomerically pure 1,2-cyclic sulfamidates with phenol, followed by a Mitsunobu reaction. Unfortunately, sufficiently acidic NH and activation via a suitable activator (PPh_3_ or DEAD) are necessary to achieve seven-membered 1,4-BZO ring formation through the usual Mitsunobu mechanism. The Saá group^3b^ reported a single example of enantioenriched α-vinyl 1,4-BZOs formed via enantioselective rhodium-catalyzed hydro-functionalizations of allenes, albeit with poor enantioselectivity. To the best of our knowledge, only a few examples illustrate the efficient preparation of enantioenriched 1,4-BZOs. Recently, Qiu^3c^ reported a systematic study of iridium-catalyzed construction of enantioenriched α-vinyl 1,4-BZOs via intramolecular asymmetric allylic etherification of salicylic acid derivatives, and Gong^3d^ disclosed a NHC/Ir/urea co-catalyzed formal [4+3] annulation reaction of anthranilaldehyde with vinyl aziridine (Figure 1). Very recently, during the preparation of the manuscript, the Qiu group reported the enantioselective construction of pyrimidine-fused oxazepines through iridium-catalyzed intramolecular asymmetric allylic etherification of pyrimidinemethanols.^3e^ However, in addition to the use of transition metals, chiral ligands, and oxidants, the elegant methods mentioned above also require suitable substituents on the amine (-Ts or -Bn), which inevitably requires an additional N deprotection step for further N derivatization of 1,4-BZOs, limiting widespread use. Therefore, new methods for the preparation of optically active 1,4-BZO from cheap raw materials with a number of N substituents are being sought while respecting the principle of sustainability and atom step economy. Oxetanes are small, strained ring motifs and have recently emerged as significantly versatile building blocks used in a variety of synthetically important nonchiral transformations via strategic manipulations due to their intrinsic high energies.^4^ Nevertheless, new strategies for exploiting oxetanes as potential synthetic intermediates to synthesize biologically important heterocyclic motifs through enantioselective desymmetrization continue to emerge.^5a−5i^ One of the first works using opening of 3-substituted oxetanes was examined by Sun in the presence of a sulfur nucleophile as an effective way to synthesize chiral compounds.^5j^ Interestingly, Kuduk investigated one-pot synthesis of larger ring systems using mild intramolecular oxetane ring opening.^5k^ In 2021, we disclosed a versatile synthesis of chiral 3,4-dihydro-2*H*-1,4-benzoxazines^5b^ along with preliminary studies of enantioenriched 1,4-BZOs through enantioselective desymmetrization of 3-tethered oxetanes. Compared with the well-developed synthesis of constitutionally stable six-membered chiral N,O-heterocyclic compounds, the enantioselective construction of a seven-membered heterocycle is highly challenging due to the unfavorable kinetics, thermodynamics, and less ordered ring-closing transition state.^5e,5f^ As a continuation of our study, we wonder whether further systematic optimization of the developed design of ring expansion of the 3-tethered oxetanes could afford better results for enantioenriched seven-membered 1,4-BZOs that account for a significant portion of therapeutics.

## Results and Discussion

Inspired by our previous work on the efficient preparation of benzoxazines,^5b^ we started our investigation by examining the enantioselective desymmetrization of amine **1a** in the presence of diphenyl phosphate at 25 °C. The reaction did not provide any product. However, after the reaction mixture had been heated to 45 °C, the desired benz[1,4]oxazepine **2a** was formed in 62% yield. On the basis of the initial results, we continued our investigation with chiral phosphoric acids CPA* (Table 1). Unfortunately, the reaction in the presence of (*R*)**-CPA-1** provided product **2a** in poor yield and as a racemate. Introducing a phenyl ring on the BINOL scaffold in catalyst (*R*)**-CPA-2** provided the product in good yield and more satisfying enantiocontrol. Interestingly, when we influenced the aromaticity of the phenyl ring with electron-withdrawing groups, such as the CF_3_ group in catalyst (*R*)**-CPA-3**, the reaction produced **2a** with a decreased enantioselectivity. Similarly, using chiral phosphoric acid (*R*)**-CPA-4** with SiPh_3_ groups did not improve the yield or enantioselectivity of product **2a**. Thus, we tested chiral phosphoric acids with a more sterically hindered substituent such as naphthyl. In the presence of (*R*)**-CPA-5** bearing a 1-naphthyl group, the reaction provided **2a** in good yield (85%) and high enantioselectivity (81%). Notably, using catalyst (*R*)**-CPA-6** with the regioisomeric 2-naphthyl group did not lead to any product, probably because of the high steric hindrance. Last but not least, we also investigated (*R*)**-CPA-7** containing sterically hindered triisopropylphenyl groups. Interestingly, this catalyst provides almost the same results as (*R*)**-CPA-5**, 82% yield and 82% enantiomeric purity. Having identified the influence of various substituents on the BINOL backbone, we investigated SPINOL-derived chiral phosphoric acid (*R*)**-CPA-8** bearing a 1-naphthyl group.

**Table 1:**
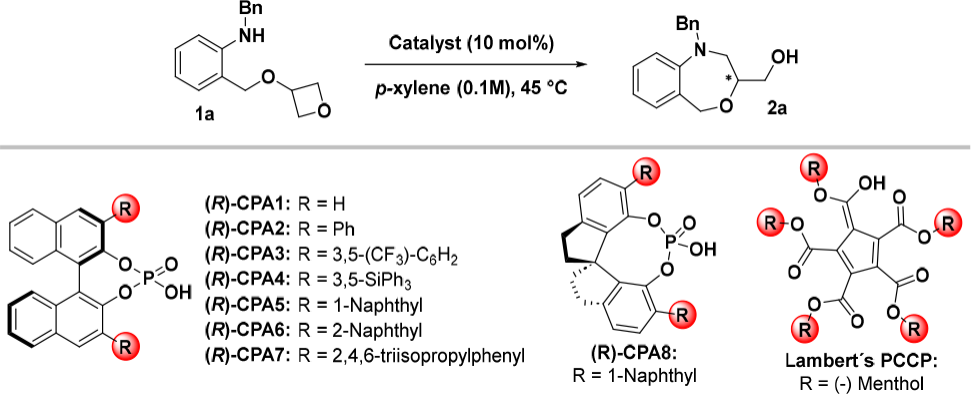
Catalyst Screening^a^

entry	catalyst	time (h)	yield (%)^b^	ee (%)^c^
1	(C_6_H_5_O)_2_P(O)OH	24	62	0
2	(*R*)**-CPA-1**	72	30	0
3	(*R*)**-CPA-2**	48	52	61
4	(*R*)**-CPA-3**	42	28	32
5	(*R*)**-CPA-4**	72	10	25
6	(*R*)**-CPA-5**	24	81	81
7	(*R*)**-CPA-6**	72	nr	nr
8	(*R*)**-CPA-7**	24	82	82
9	(*R*)**-CPA-8**	40	85	92
10	PCCP	72	60	12

a Determined by ^1^H NMR of the crude reaction mixture.

b Isolated yields after column chromatography.

c IA column (95/5 heptane/isopropanol, 1 mL/min).

Catalyst (*R*)**-CPA-8** provided the desired product in high yield (85%) and, more interestingly, with high enantioselectivity (92%, entry 8). We also tested Lambert’s PCCP catalyst containing five (−)-menthol substituents. Unfortunately, this chiral catalyst was not suitable for this transformation and did not provide **2a** in satisfactory enantioselectivity. We eventually identified chiral phosphoric acid (*R*)**-CPA-8** with a SPINOL backbone as an optimal catalyst for the studied transformation, enabling product formation in high enantioselectivity (92%). Having identified these optimized conditions, we investigated the effect of the solvent on the reaction.

We started our investigation with aromatic solvents, such as toluene, benzene, chlorobenzene, and *p*-xylene (Table 2). Interestingly, the highest yield of **2a** (85%) with high enantiomeric purity (92% ee) was obtained in *p*-xylene. Conversely, chlorinated solvents such as DCM and chloroform did not provide product **2a**, even after a prolonged reaction time. Further investigation of ethereal and dipolar aprotic solvents, such as acetonitrile, did not result in higher yields or stereoselectivities. Thus, *p*-xylene was selected as the optimal solvent, affording the desired product **2a** in 85% yield with high enantiomeric purity (92% ee). Last but not least, different temperatures were tested; the reaction did not proceed at room temperature, and at 60 °C, we obtained the desired product in a lower yield of 72% with the same enantiomeric purity (92% ee). On the basis of these observations, we selected 45 °C as the suitable reaction temperature.

**Table 2:**
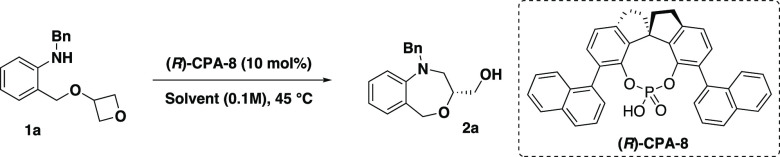
Solvent Screening^a^

entry	solvent	time (h)	yield (%)^b^	ee (%)^c^
1	toluene	24	60	91
2	benzene	20	79	91
3	*p*-xylene	40	85	92
4	chlorobenzene	35	64	88
5	DCM	72	traces	nd
5	CHCl_3_	72	traces	nd
6	MeCN	72	11	57
7	1,4-dioxane	72	traces	nd
8	*tert*-butyl methyl ether	72	20	92

a Determined by ^1^H NMR of the crude reaction mixture.

b Isolated yields after column chromatography.

c IA column (95/5 heptane/isopropanol, 1 mL/min).

With the optimized reaction conditions in hand, we began exploring the scope of enantioselective CPA-catalyzed desymmetrization by varying oxetane derivative **1** (Table 3). First, we assessed the effect of the electronic properties of the benzyl substituent in the amine group. In general, the reaction tolerates both electron-rich and electron-withdrawing groups on the benzyl substituent, affording benz[1,4]oxazepines **2b**–**2k** in good yields with a high degree of enantiocontrol. Chiral benz[1,4]oxazepines **2b** and **2c** containing electron-donating groups on the benzyl moiety in the *para* position were prepared in good yields (69–70%) and enantioselectivities (both 88% ee). More highly enantiomerically pure benz[1,4]oxazepines **2d**–**2h** were obtained with substrates bearing electron-withdrawing groups on the benzyl moiety in the *para* position. For example, benz[1,4]oxazepine **2h** was isolated in excellent yield (96%) with high enantiomeric purity (94% ee). It is noteworthy that substrates bearing *meta*- and *ortho*-substituted aromatic rings with halogens afforded the corresponding benz[1,4]oxazepines (**2i** and **2j**, respectively) in high yields (92–98%) with high enantioselectivities (90–92% ee). Interestingly, introducing a strongly electron-withdrawing nitro group at the *meta* position (**2k**) resulted in lower enantiomeric purity (88% ee). Notably, substrates with a bulkier naphthyl substituent (**1l**) and a heteroaromatic thienyl moiety (**1m**) also performed well and delivered products **2l** and **2m** in high yields (78–85%) with a high degree of enantiocontrol (90–92% ee). Changing the *N*-benzyl group to an *N*-allyl group was also tolerated, providing a high yield of benz[1,4]oxazepane **2n**. However, using amine **1o** bearing an *N*-methyl group afforded the product in poor yield (30%) and enantioselectivity (65% ee). Unfortunately, changing the benzyl group to the Boc group (**1p**) led to no product, probably due to the lower nucleophilicity of the amine moiety. Then, we introduced a phenyl ring on the starting amine, which afforded the desired product **2q** in high yield (75%) with high enantiomeric purity (90% ee). Similarly, the use of a starting amine bearing an electron-rich *p*-methoxyphenyl group afforded the desired product **2r** in good yield (60%) with excellent enantioselectivity (94% ee). Despite extensive attempts, we could not obtain chiral benz[1,4]oxazepines bearing an electron-poor *p*-trifluoromethylphenyl group (**2s**) and bulky naphthyl substituent (**2t**). However, the use of a starting amine bearing a *p*-bromophenyl group afforded the desired product **2u** in good yield (61%); unfortunately, we were unable to determine the enantiomeric purity of this product.

**Table 3:**
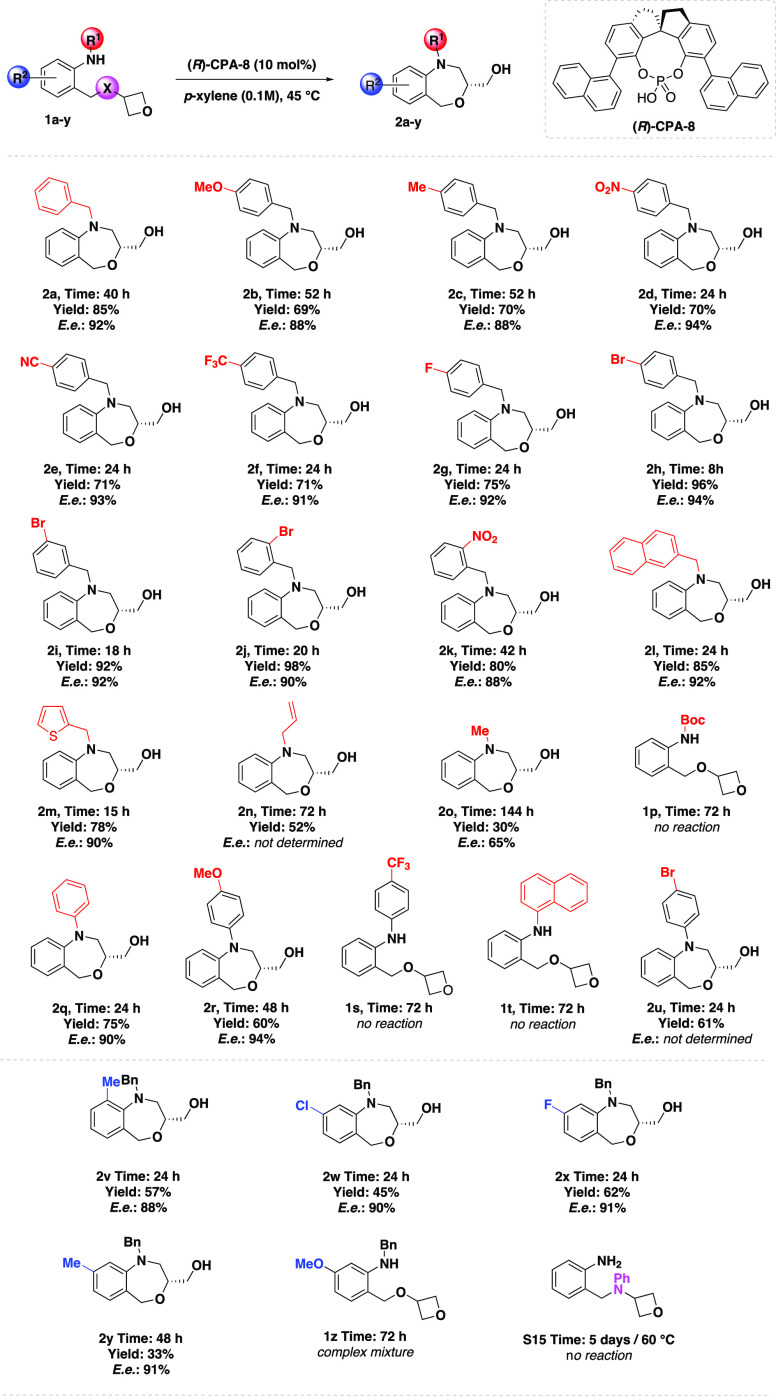
Substrate Scope^a^

a Performed with **1a**–**y** (0.05 mmol) and the (*R*)-**CPA-8** catalyst (10 mol %) in *p*-xylene (0.5 mL, 0.1 M) at 45 °C. Isolated yields with enantiomeric excesses (ee) determined by HPLC analysis.

Next, we investigated the influence of different substituents on the aromatic ring of starting anilines. We started with a derivative containing a methyl group in the *ortho* position, which provided the desired product **2v** in moderate yield (57%) with good enantioselectivity (88% ee). Then, we focused on the synthesis of halogenated derivatives. Unfortunately, we were successful only in the preparation of chlorinated and fluorinated aromatic rings in the *meta* position due to the unexpected parasitic reaction and decomposition of the starting material. In the case of the chlorinated derivative, the reaction provided final product **2w** in good yield (45%) with high enantioselectivity (90% ee). Fluorinated benz[1,4]oxazepine **2x** was obtained in higher yield (62%) with a similar level of enantiocontrol (91% ee). Notably, the substrate containing a methyl group at the *metha* position (**2y**) afforded the desired product in lower yield (33%) but with high enantioselectivity (91% ee). Interestingly, introducing a methoxy group at the *metha* position (**1z**) resulted in a complex mixture of products after 72 h. Further investigation with starting amine **S15** revealed the inefficient synthesis of the 1,4-benzodiazepine ring via enantioselective desymmetrization. No product was obtained from the reaction using nitrogen containing 3-substituted oxetane after 5 days at 60 °C. Despite several attempts to further modify starting amine **S15** with benzyl or tosyl groups, we were unsuccessful.

**Figure 2:**
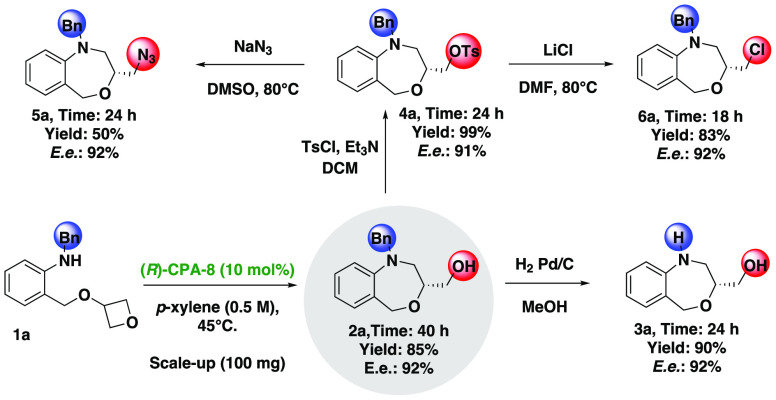
Synthetic transformations and applications.

To show the applicability of the developed desymmetrization process, we performed a reaction with **1a** at 1 mmol scale, giving chiral benz[1,4]oxazepine **2a** with the same efficacy [85% yield and 92% ee (Figure 2)]. As an example of further transformations, benz[1,4]oxazepine **2a** was converted into compounds **3a**–**6a**. The benzyl group on the nitrogen atom was successfully cleaved using the Pd/C system under a hydrogen atmosphere in methanol, giving compound **3a** in good yield (90%) without changing the enantiomeric purity of the product. In addition, we tosylated alcohol **4a** in a nearly quantitative yield. Tosylate **4a** treated with NaN_3_ afforded the corresponding product **5a**. Unfortunately, the reaction provided azide **5a** in low yield (50%) but with the same enantiomeric purity (92% ee). To further investigate the reactivity of tosylated product **4a**, the reaction with LiCl was tested. The reaction was carried out in DMF, providing the desired product **6a** in good yield (83%) with the same enantiomeric purity (92% ee).

**Figure 3:**
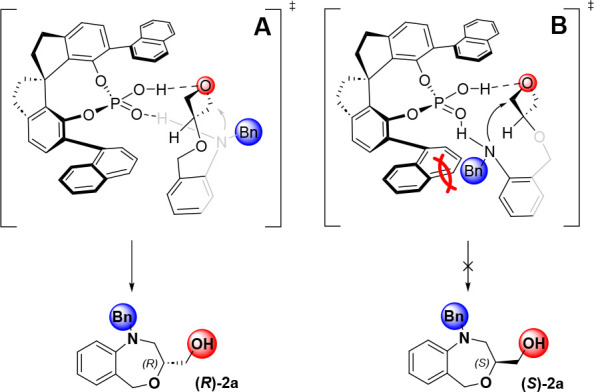
Plausible transition state.

Last but not least, we constructed a plausible transition state for the formation of the (*R*) isomer (Figure 3). On the basis of the pioneering work published by Sun, the authors proposed two transition states for activation of 3-substituted oxetanes.^5j^ In our case, oxetane was lacking a hydrogen bond donor substituent and a large substituent had to be oriented opposite the catalyst pocket, which favored nucleophilic attack on the reactive center from the back side due to the steric interactions with the catalysts, a bulky naphtyl substituent and a substrate.

## Conclusion

In summary, we have developed an effective method for the synthesis of chiral 1,4-benzoxazepines via enantioselective desymmetrization of 3-substituted oxetanes. The reaction is efficiently catalyzed by SPINOL-derived chiral phosphoric acid, affording benz[1,4]oxazepines in good to high yields (≤98%) with high enantioselectivity (≤94% ee). To demonstrate the versatility of the developed method, a broad range of substrates were tested. In addition, selected transformations of the final product were realized, including N-debenzylation and conversion of the hydroxyl group to other functionalities.

## Experimental Section

**General Experimental Procedures, Reaction Setup, Reagents, and Solvents.** All reactions were carried out under an inert argon or nitrogen atmosphere using standard oven-dried round-bottom flasks, unless otherwise stated. All enantiomeric desymmetrization reactions and further transformations were performed in a 4.0 mL vial. All reagents were used as supplied from commercial sources without further purification unless otherwise stated. Tetrahydrofuran, Et_2_O, DCM, and toluene were purified by distillation on a site under an inert atmosphere via the following processes. Tetrahydrofuran and Et_2_O were predried over a sodium wire and then distilled. DCM and toluene were distilled from calcium hydride under an inert atmosphere. Methanol was predried over magnesium turnings and iodine and then distilled under an inert atmosphere. EtOAc and *n*-hexanes were distilled. The heating source included an oil bath and modular aluminum heating blocks for 4 mL vials. Analytical thin-layer chromatography was performed using precoated Merck aluminum silica gel plates (Silica gel 60 F254). Visualization was achieved by ultraviolet fluorescence (λ = 254 and 365 nm) and/or staining with potassium permanganate (KMnO_4_) or ceric ammonium molybdate (CAM). Flash column chromatography was performed using silica gel (230–400 mesh). All ratios of eluents are quoted as v/v. Chiral HPLC was carried out using a LC20AD Shimadzu liquid chromatograph with a SPDM20A diode array detector with Daicel Chiralpak IA, Daicel Chiralpak IB, Daicel Chiralpak IC, Daicel Chiralpak AD, Daicel, and Daicel Chiralpak OD-H columns (4.6 mm × 250 mm, 5.0 μm) in a mixed solvent system of heptane and isopropanol at 25 °C unless otherwise stated. The racemic compounds were prepared by treating the respective oxetanes with diphenyl phosphate (0.2 equiv) in *p*-xylene (0.1 M) at 45 °C on modular aluminum heating blocks for vials. Samples for chiral HPLC were prepared by dissolving the corresponding products in heptane/i-PrOH [80/20 or 80/10 (v/v)]. The names of the compounds were generated by the computer program Chem Draw according to the guidelines specified by IUPAC. ^1^H NMR spectra were recorded on 400 or 600 MHz Bruker spectrometers. Chemical shifts are reported in parts per million, and the spectra are calibrated to the resonance resulting from incomplete deuteration of the solvent (CDCl_3_, 7.26 ppm). ^13^C NMR spectra were recorded on the same spectrometers with complete proton decoupling using CDCl_3_ as the internal standard (^13^CDCl_3_, 77.16 ppm). ^19^F NMR spectra were recorded on the same spectrometer. Data are reported as follows: chemical shift δ, multiplicity (s, singlet; d, doublet; t, triplet; q, quartet; p, pentet; bs, broad singlet; m, multiplet or combinations thereof; ^13^C and all other nuclides except ^1^H are singlets unless otherwise stated). ^1^H NMR signals are reported in parts per million to two decimal places, and all other nuclide signals to one decimal place (^13^C NMR signals are reported in parts per million to two decimal places where the first decimal is exactly the same). Coupling constants are reported in hertz to a maximum of three significant figures. High-resolution mass spectra were recorded with a LCQ Fleet spectrometer and an LC-QTOF instrument at the Department of Chemistry at Charles University. The ionization method is noted: positive/negative electrospray ionization (±ESI) or atmospheric-pressure photoionization (±APPI). Samples for measurement of HRMS were prepared by dissolving the corresponding sample in a methanol/CH_3_CN mixture. The masses reported as “found” and “calculated for” are the mass/charge ratios. Measured values are reported to four decimal places and are within ±5 ppm of the calculated value. The calculated values are based on the most abundant isotope unless otherwise stated in the chemical formula. IR DRIFT or ATR spectra were recorded with a Nicolet AVATAR 370 FT-IR instrument and are in units of inverse centimeters. Optical rotations were measured in spectrophotometric grade CHCl_3_ or MeOH on an AU-Tomatica polarimeter, Autopol III using a sodium lamp (λ = 589 nm, the sodium D line). [α]_D_ values are reported at the stated temperature, with a concentration in units of grams per 100 mL. Single-crystal X-ray diffraction of the molecule and the absolute structure of **2d** were determined by performing an X-ray diffraction experiment on a Bruker D8 VENTURE Kappa Duo PHOTONIII instrument with IμS microfocus sealed tube Cu Kα (λ = 1.54178) at a temperature of 120(2) K. The absolute configuration of product **2d** was confirmed through a single X-ray analysis. The absolute configurations of other products were assigned tentatively by analogy.

### Procedure for the Synthesis of *N*-Phenyloxetan-3-amine (**S1**)

The contents of the round-bottom flask charged with aniline (5.3 mmol, 494 mg, 1 equiv), oxetan-3-one (13.4 mmol, 956 mg, 2.5 equiv), and AcOH (10.7 mmol, 0.612 mL, 2 equiv) were dissolved in MeOH (0.3 M) at 0 °C. The reaction mixture was stirred for 3 h; then NaCNBH_3_ (10.7 mmol, 672 mg, 2 equiv) was added at 0 °C, and the mixture stirred until the starting material had been fully converted (controlled by TLC). After completion of the reaction, the crude was quenched with saturated NaHCO_3_, the MeOH was removed on a rotary evaporator under reduced pressure, and the compound extracted with EtOAc (thrice), washed with Brine (once), concentrated on a rotary evaporator, and purified by silica gel column chromatography using a 5/1 hexane/EtOAc mixture, which gave the corresponding 3-substituted oxetane.

#### *N*-Phenyloxetan-3-amine (**S1**)

The product was obtained by column chromatography (silica, 5/1 hexane/EtOAc) as a white oil in 88% (696 mg) yield: ^1^H NMR (400 MHz, CDCl_3_) δ 7.24–7.14 (m, 2H), 6.78 (tq, *J* = 7.6, 1.0 Hz, 1H), 6.56–6.45 (m, 2H), 5.01 (td, *J* = 6.3, 5.9, 0.8 Hz, 2H), 4.68–4.59 (m, 1H), 4.54 (t, *J* = 6.1 Hz, 2H), 4.20 (brs, 1H); ^13^C{^1^H} NMR (101 MHz, CDCl_3_) δ 146.2, 129.6 (2C), 118.8, 113.2, 79.4 (2C), 48.7; IR (ATR) 3315, 2875, 1599, 1518, 1491, 1315, 1269, 964, 945, 750 cm^–1^; HRMS (ESI) *m*/*z* [M + H]^+^ calcd for C_9_H_12_NO 150.0913, found 150.0911.

### General Procedure A for the Synthesis of Starting Materials. Synthesis of Substituted Oxetanes (**S2–S6**)

The contents of the round-bottom flask charged with the corresponding 1-(bromomethyl)-2-nitrobenzene (1 equiv) and K_2_CO_3_ (2 equiv) were dissolved in acetonitrile. Then, oxetan-3-ol (1.3 equiv) was added to the reaction mixture, and the mixture stirred until the starting material had been fully converted (controlled by TLC). The reaction mixture was filtered through Celite and washed with an excess of EtOAc. The solvent was removed on a rotary evaporator under reduced pressure and purified by silica gel column chromatography using a 5/1 hexane/EtOAc mixture, which gave the corresponding aniline compounds.

#### 3-[(2-Nitrobenzyl)oxy]oxetane (**S2**)

The product was obtained by column chromatography (silica, 5/1 hexane/EtOAc) as a yellow oil in 45% (871 mg) yield: ^1^H NMR (400 MHz, CDCl_3_) δ 8.07 (dd, *J* = 8.3, 1.4 Hz, 1H), 7.82 (dd, *J* = 7.8, 1.3 Hz, 1H), 7.66 (td, *J* = 7.6, 1.4 Hz, 1H), 7.46 (td, *J* = 7.8, 1.5 Hz, 1H), 4.84–4.79 (m, 3H), 4.70 (dq, *J* = 10.9, 5.1 Hz, 3H); ^13^C{^1^H} NMR (101 MHz, CDCl_3_) δ 147.2, 134.2, 133.9, 128.8, 128.4, 124.8, 78.5 (2C), 72.8, 67.4; IR (ATR) 3111, 3089, 3039, 2927, 2900, 1520, 1338, 1309, 1196, 1176, 1032, 1005, 972, 937, 870, 731 cm^–1^; HRMS (ESI) *m*/*z* [M + Na]^+^ calcd for C_10_H_11_NNaO_4_ 232.0580, found 232.0582.

#### 3-[(3-Methyl-2-nitrobenzyl)oxy]oxetane (**S3**)

The product was obtained by column chromatography (silica, 5/1 hexane/EtOAc) as a yellow oil in 40% (827 mg) yield: ^1^H NMR (400 MHz, CDCl_3_) δ 7.42–7.34 (m, 1H), 7.27 (dd, *J* = 6.9, 2.6 Hz, 0H), 4.76–4.69 (m, 1H), 4.63–4.56 (m, 1H), 4.47 (s, 1H), 2.35 (s, 1H); ^13^C{^1^H} NMR (101 MHz, CDCl_3_) δ 150.3, 131.4 (2C), 130.6 (2C), 127.2, 78.5 (2C), 72.7, 67.0, 17.8; IR (ATR) 2951, 2927, 2875, 1525, 1471, 1456, 1365, 1313, 1284, 1257, 1184, 1136, 1109, 1038, 970, 866, 852, 785, 748 cm^–1^; HRMS (ESI) *m*/*z* [M + Na]^+^ calcd for C_11_H_13_NNaO_4_ 246.0737, found 246.0741.

#### 3-[(4-Chloro-2-nitrobenzyl)oxy]oxetane (**S4**)

The product was obtained by column chromatography (silica, 1/1 hexane/EtOAc) as a yellow oil in 15% (338 mg) yield: ^1^H NMR (400 MHz, CDCl_3_) δ 8.09 (d, *J* = 2.1 Hz, 1H), 7.81 (dt, *J* = 8.4, 1.0 Hz, 1H), 7.64 (dd, *J* = 8.4, 2.2 Hz, 1H), 4.83 (ddd, *J* = 6.9, 4.4, 1.3 Hz, 2H), 4.78 (s, 2H), 4.75–4.71 (m, 1H), 4.71–4.66 (m, 2H); ^13^C{^1^H} NMR (101 MHz, CDCl_3_) δ 147.4, 134.2, 134.0, 133.0, 130.1, 124.9, 78.5 (2C), 73.0, 67.0; IR (ATR) 3114, 2978, 2947, 1595, 1566, 1356, 1336, 1234, 1184, 1041, 1005, 964, 931, 781, cm^–1^; HRMS (ESI) *m*/*z* [M + Na]^+^ calcd for C_10_H_10_ClNNaO_4_ 266.0191, found 266.0188.

#### 3-[(4-Fluoro-2-nitrobenzyl)oxy]oxetane (**S5**)

The product was obtained by column chromatography (silica, 5/1 hexane/EtOAc) as a yellow oil in 25% (526 mg) yield: ^1^H NMR (400 MHz, CDCl_3_) δ 7.87–7.79 (m, 2H), 7.40 (ddd, *J* = 8.7, 7.4, 2.7 Hz, 1H), 4.83 (ddd, *J* = 6.8, 4.3, 1.3 Hz, 2H), 4.78 (t, *J* = 1.3 Hz, 2H), 4.76–4.70 (m, 1H), 4.71–4.66 (m, 2H); ^13^C{^1^H} NMR (101 MHz, CDCl_3_) δ 161.45 (d, *J* = 250.9 Hz, 1C), 147.56 (d, *J* = 8.4 Hz, 1C), 130.7 (d, *J* = 7.8 Hz, 1C), 121.2 (d, *J* = 20.9 Hz, 1C), 112.5 (d, *J* = 26.6 Hz, 1C), 78.5 (2C), 72.9, 67.0; ^19^F NMR (376 MHz, CDCl_3_) δ −111.07 to −111.16 (m); IR (ATR) 3122, 2966, 2947, 1620, 1589, 1414, 1319, 1286, 1257, 1039, 1024, 972, 948, 814, 768, cm^–1^; HRMS (ESI) *m*/*z* [M + Na]^+^ calcd for C_10_H_10_FNNaO_4_ 250.0486, found 250.0487.

#### *N*-(2-Nitrobenzyl)-*N*-phenyloxetan-3-amine (**S6**)

The product was obtained by column chromatography (silica, 5/1 hexane/EtOAc) as a yellow oil in 48% (1.26 g) yield: ^1^H NMR (400 MHz, CDCl_3_) δ 8.17 (dd, *J* = 8.2, 1.3 Hz, 1H), 7.82 (dq, *J* = 7.8, 1.1 Hz, 1H), 7.65 (td, *J* = 7.6, 1.3 Hz, 1H), 7.54–7.44 (m, 1H), 7.26–7.16 (m, 2H), 6.85 (tt, *J* = 7.3, 1.1 Hz, 1H), 6.54–6.45 (m, 2H), 5.03–4.94 (m, 1H), 4.93–4.85 (m, 4H), 4.68 (dd, *J* = 6.9, 6.1 Hz, 2H); ^13^C{^1^H} NMR (101 MHz, CDCl_3_) δ 147.9, 147.4, 135.8, 134.4, 129.7 (2C), 129.5, 128.4, 125.6, 119.8, 114.3 (2C), 76.3, 54.1, 52.1; IR (ATR) 3094, 2966, 2945, 1597, 1576, 1402, 1365, 1342, 1211, 1180, 974, 947, 793 cm^–1^; HRMS (ESI) *m*/*z* [M + Na]^+^ calcd for C_16_H_16_N_2_NaO_3_ 307.1053, found 307.1052.

### General Procedure B for the Synthesis of Starting Materials. Synthesis of Substituted Oxetanes (**S7** and **S8**)

To a suspension of NaH (60 wt %, 1.2 equiv) in THF (0.5 M) was added oxetan-3-ol (1.0 equiv) dropwise at 0 °C. The mixture was stirred at room temperature for 30 min, and a solution of benzyl bromide (1.2 equiv) was added dropwise. The reaction mixture was stirred at 60 °C overnight and then allowed to cool to room temperature. A saturated aqueous NH_4_Cl solution was added, and the organic layer was separated. The aqueous layer was extracted with Et_2_O (twice). The combined organic layers were washed with brine, dried over MgSO_4_, and concentrated under reduced pressure. The residue was purified by flash column chromatography (5/1 hexane/EtOAc) to give the desired product.

#### 3-[(4-Methyl-2-nitrobenzyl)oxy]oxetane (**S7**)

The product was synthesized by general procedure B and obtained by column chromatography (silica, 5/1 hexane/EtOAc) as a yellow oil in 58% (938 mg) yield: ^1^H NMR (400 MHz, CDCl_3_) δ 7.89 (d, *J* = 1.8 Hz, 1H), 7.67 (d, *J* = 7.9 Hz, 1H), 7.47 (d, *J* = 7.9 Hz, 1H), 4.84–4.79 (m, 2H), 4.77 (s, 2H), 4.75–4.66 (m, 3H), 2.44 (s, 3H); ^13^C{^1^H} NMR (101 MHz, CDCl_3_) δ 147.2, 138.9, 134.7, 131.2, 128.9, 125.2, 78.7 (2C), 72.8, 67.4, 21.0; IR (ATR) 3078, 2925, 2875, 1523, 1358, 1294, 1132, 970, 839, 818, 715 cm^–1^; HRMS (ESI) *m*/*z* [M + Na]^+^ calcd for C_11_H_13_NNaO_4_ 246.0737, found 246.0736.

#### 3-[(4-Methoxy-2-nitrobenzyl)oxy]oxetane (**S8**)

The product was synthesized by general procedure B and obtained by column chromatography (silica, 5/1 hexane/EtOAc) as a yellow oil in 53% (859 mg) yield: ^1^H NMR (400 MHz, CDCl_3_) δ 7.67 (d, *J* = 8.7 Hz, 1H), 7.58 (d, *J* = 2.7 Hz, 1H), 7.19 (dd, *J* = 8.7, 2.7 Hz, 1H), 4.84–4.77 (m, 2H), 4.74 (s, 2H), 4.68 (q, *J* = 5.6, 5.0 Hz, 3H), 3.88 (s, 3H); ^13^C{^1^H} NMR (101 MHz, CDCl_3_) δ 159.4, 148.1, 130.3, 126.0, 120.3, 109.6, 78.7 (2C), 72.7, 67.3, 56.0; IR (ATR) 3111, 2958, 2879, 2839, 1525, 1336, 1238, 1101, 1030, 970, 858, 756 cm^–1^; HRMS (ESI) *m*/*z* [M + Na]^+^ calcd for C_11_H_13_NNaO_5_ 262.0686, found 262.0683.

### General Procedure for the Reduction of the Nitro Group (**S9–S14**)

The round-bottom flask charged with nitrobenzene (1 equiv), NiCl_2_·6H_2_O (0.2 equiv), and a 10/1 CH_3_CN/H_2_O mixture was cooled to 0 °C. After the mixture had been stirred for 5 min, NaBH_4_ (4 equiv) was added portionwise. A fine black precipitate immediately formed. Then, the mixture was stirred for the next 15 min at room temperature, and the reaction later quenched with aqueous NH_4_Cl. The reaction mixture was filtered through Celite and washed with excess MeOH. The MeOH was removed under high vacuum to afford the crude product. The residue obtained was dissolved in EtOAc and washed with water (thrice). The organic layer was collected and dried over Na_2_SO_4_. The solvent was removed on a rotary evaporator under reduced pressure and purified by silica gel column chromatography using a 5/1 hexane/EtOAc mixture, which gave the corresponding aniline compounds.

#### 2-[(Oxetan-3-yloxy)methyl]aniline (**S9**)

The product was obtained by column chromatography (silica, 1/1 hexane/EtOAc) as a yellow oil in 92% (394 mg) yield: ^1^H NMR (400 MHz, CDCl_3_) δ 7.15 (td, *J* = 7.6, 1.5 Hz, 1H), 7.02 (dd, *J* = 7.7, 1.1 Hz, 1H), 6.72 (t, *J* = 7.7 Hz, 2H), 4.67 (dt, *J* = 6.0, 3.4 Hz, 2H), 4.64–4.56 (m, 3H), 4.48 (s, 2H), 4.25 (s, 2H); ^13^C{^1^H} NMR (101 MHz, CDCl_3_) δ 145.9, 130.1, 129.9, 121.7, 118.5, 116.2, 78.8 (2C), 71.8, 70.4; IR (ATR) 3354, 2943, 2873, 1606, 1585, 1493, 1311, 1294, 1269, 1016, 964, 931, 752 cm^–1^; HRMS (ESI) *m*/*z* [M + Na]^+^ calcd for C_10_H_13_NNaO_2_ 202.0838, found 202.0839.

#### 2-Methyl-6-[(oxetan-3-yloxy)methyl]aniline (**S10**)

The product was obtained by column chromatography (silica, 1/1 hexane/EtOAc) as a yellow oil in 83% (359 mg) yield: ^1^H NMR (400 MHz, CDCl_3_) δ 6.26 (dd, *J* = 8.0, 1.4 Hz, 1H), 6.77 (dt, *J* = 7.5, 1.2 Hz, 1H), 6.70 (t, *J* = 7.8 Hz, 1H), 5.43 (brs, 2H), 5.23 (ddd, *J* = 11.2, 6.1, 5.1 Hz, 1H), 4.97 (ddd, *J* = 7.2, 6.1, 1.0 Hz, 2H), 4.88–4.79 (m, 2H), 2.29 (s, 3H); ^13^C{^1^H} NMR (101 MHz, CDCl_3_) δ 145.0, 131.7, 123.8 (2C), 119.7, 109.2 (2C), 78.2 (2C), 70.8, 17.6; IR (ATR) 3357, 2873, 1606, 1585, 1489, 1373, 1311, 1293, 1159, 1107, 964, 931, 750, cm^–1^; HRMS (ESI) *m*/*z* [M + Na]^+^ calcd for C_11_H_15_NNaO_2_ 216.0995, found 216.0995.

#### 5-Chloro-2-[(oxetan-3-yloxy)methyl]aniline (**S11**)

The product was obtained by column chromatography (silica, 1/1 hexane/EtOAc) as a yellow oil in 75% (329 mg) yield: ^1^H NMR (400 MHz, CDCl_3_) δ 6.95–6.88 (m, 1H), 6.66 (d, *J* = 7.3 Hz, 2H), 4.72–4.64 (m, 2H), 4.61–4.53 (m, 3H), 4.42 (s, 2H), 4.21 (s, 2H); ^13^C{^1^H} NMR (101 MHz, CDCl_3_) δ 147.4, 135.2, 130.9, 119.8, 117.9, 115.5, 78.7 (2C), 71.8, 69.7; IR (ATR) 3456, 2947, 2873, 1601, 1576, 1493, 1458, 1388, 1358, 1203, 1182, 964, 928, 789, 773 cm^–1^; HRMS (ESI) *m*/*z* [M + Na]^+^ calcd for C_10_H_12_ClNNaO_2_ 236.0449, found 236.0450.

#### 5-Fluoro-2-[(oxetan-3-yloxy)methyl]aniline (**S12**)

The product was obtained by column chromatography (silica, 1/1 hexane/EtOAc) as a yellow oil in 76% (330 mg) yield: ^1^H NMR (400 MHz, CDCl_3_) δ 7.03–6.87 (m, 1H), 6.46–6.29 (m, 2H), 4.67 (qt, *J* = 6.2, 1.8 Hz, 2H), 4.63–4.53 (m, 3H), 4.42 (s, 2H), 4.19 (brs, 2H); ^13^C{^1^H} NMR (101 MHz, CDCl_3_) δ 164.13 (d, *J* = 244.6 Hz, 1C), 148.05 (d, *J* = 11.1 Hz, 1C), 131.37 (d, *J* = 10.2 Hz, 1C), 117.35 (d, *J* = 2.7 Hz, 1C), 104.61 (d, *J* = 21.6 Hz, 1C), 102.67 (d, *J* = 24.7 Hz, 1C), 78.8 (2C), 71.8, 69.8; ^19^F NMR (376 MHz, CDCl_3_) δ −112.85 (ddd, *J* = 10.4, 8.7, 6.3 Hz); IR (ATR) 3464, 2964, 2949, 2922, 2873, 1608, 1591, 1444, 1390, 1254, 1225, 1070, 1033, 1007, 993, 968, 771 cm^–1^; HRMS (ESI) *m*/*z* [M + Na]^+^ calcd for C_10_H_12_FNNaO_2_ 220.0744, found 220.0744.

#### 5-Methyl-2-[(oxetan-3-yloxy)methyl]aniline (**S13**)

The product was obtained by column chromatography (silica, 1/1 hexane/EtOAc) as a yellow oil in 89% (192 mg) yield: ^1^H NMR (400 MHz, CDCl_3_) δ 6.89 (d, *J* = 7.8 Hz, 1H), 6.52 (d, *J* = 5.0 Hz, 2H), 4.69–4.64 (m, 2H), 4.62–4.56 (m, 3H), 4.44 (s, 2H), 4.22 (s, 2H), 2.26 (s, 3H); ^13^C{^1^H} NMR (101 MHz, CDCl_3_) δ 146.1, 139.9, 130.1, 119.2, 118.8, 116.8, 78.9 (2C), 71.7, 70.2, 21.4; IR (ATR) 3444, 3363, 2970, 2877, 1618, 1514, 1360, 1134, 962, 872, 798 cm^–1^; HRMS (ESI) *m*/*z* [M + Na]^+^ calcd for C_11_H_15_NNaO_2_ 216.0995, found 216.0993.

#### 5-Methoxy-2-[(oxetan-3-yloxy)methyl]aniline (**S14**)

The product was obtained by column chromatography (silica, 1/1 hexane/EtOAc) as a yellow oil in 85% (329 mg) yield: ^1^H NMR (400 MHz, CDCl_3_) δ 7.15 (td, *J* = 7.7, 1.6 Hz, 1H), 7.02 (dd, *J* = 7.4, 1.5 Hz, 1H), 6.75–6.65 (m, 2H), 4.72–4.64 (m, 2H), 4.59 (d, *J* = 4.6 Hz, 3H), 4.48 (s, 2H), 4.13 (s, 2H); ^13^C{^1^H} NMR (101 MHz, CDCl_3_) δ 146.3, 130.1, 129.9, 121.5, 118.3, 115.9, 78.9 (2C), 71.8, 70.4; IR (ATR) 3446, 3363, 2947, 1682, 1614, 1510, 1331, 1203, 1169, 1030, 958, 835, 779 cm^–1^; HRMS (ESI) *m*/*z* [M + Na]^+^ calcd for C_11_H_15_NNaO_3_ 232.0944, found 232.0942.

### General Procedure for the Reduction of the Nitro Group (**S15**)

A dried glass reaction tube equipped with a magnetic stir bar was charged with aromatic nitro compounds (0.88 mmol, 224 mg, 1.0 equiv), B_2_pin_2_ (2.7 mmol, 686 mg, 3.1 equiv), and KOtBu (1.06 mmol, 119 mg, 1.2 equiv); i-PrOH (5.0 mL) was added, and the mixture was then stirred in the preheated oil base at 110 °C for 2 h. The progress of the reaction was monitored by TLC. After the mixture had been cooled to room temperature, the crude product was diluted with ethyl acetate and then washed with a saturated NaCl solution. The organic layers were dried over anhydrous Na_2_SO_4_, concentrated in vacuo, and purified by flash column chromatography to give the pure products.

#### *N*-(2-Aminobenzyl)-*N*-phenyloxetan-3-amine (**S15**)

The product was obtained by column chromatography (silica, 1/1 hexane/EtOAc) as a yellow oil in 90% (201 mg) yield: ^1^H NMR (400 MHz, CDCl_3_) δ 7.26–7.21 (m, 2H), 7.11 (td, *J* = 7.6, 1.6 Hz, 1H), 7.05 (dd, *J* = 7.5, 1.5 Hz, 1H), 6.93 (tt, *J* = 7.3, 1.1 Hz, 1H), 6.79–6.73 (m, 2H), 6.70 (ddd, *J* = 7.8, 5.2, 1.2 Hz, 2H), 4.70–4.62 (m, 3H), 4.60–4.52 (m, 2H), 4.23 (s, 2H), 4.10 (brs, 2H); ^13^C{^1^H} NMR (101 MHz, CDCl_3_) δ 149.2, 145.1, 129.6, 129.5, 128.9, 128.6, 122.7, 121.4, 118.8, 118.1, 115.9, 113.2, 79.4, 76.6, 55.2, 55.1; IR (ATR) 3419, 2951, 2871, 1495, 1456, 1348, 1311, 1265, 1155, 1095, 974, 931, 735 cm^–1^; HRMS (ESI) *m*/*z* [M + Na]^+^ calcd for C_16_H_18_N_2_NaO 277.1311, found 277.1313.

### Synthesis of Substituted Amines for Enantiomeric Desymmetrization

A 5 mL vial equipped with a magnetic stirrer was charged with amine (1 equiv), K_2_CO_3_ (2 equiv), and acetonitrile (3 mL) at room temperature. To the resulting solution was added the corresponding substituted bromide or iodine (1.1 equiv) at room temperature. The reaction mixture was stirred for 5–24 h and monitored by TLC. After the starting material had been fully consumed, the reaction mixture was filtered through Celite and evaporated under reduced pressure, and the residue was purified by silica gel column chromatography (5/1 hexane/EtOAc) to afford the desired product

#### *N*-Benzyl-2-[(oxetan-3-yloxy)methyl]aniline (**1a**)

The product was obtained by column chromatography (silica, 1/1 hexane/EtOAc) as a white solid in 79% (297 mg) yield: ^1^H NMR (400 MHz, CDCl_3_) δ 7.42–7.33 (m, 4H), 7.29 (ddd, *J* = 9.4, 4.1, 1.8 Hz, 2H), 7.24–7.17 (m, 2H), 7.04 (dd, *J* = 7.3, 1.4 Hz, 1H), 6.72–6.63 (m, 2H), 5.11 (s, 1H), 4.70–4.63 (m, 2H), 4.63–4.58 (m, 1H), 4.58–4.53 (m, 2H), 4.52 (s, 2H), 4.41 (s, 2H); ^13^C{^1^H} NMR (101 MHz, CDCl_3_) δ 147.5, 139.4, 130.1, 129.9, 128.8 (2C), 127.4, 127.3 (2C), 121.1, 116.9, 111.1, 78.8 (2C), 71.6, 70.8, 47.9; IR (ATR) 3365, 2979, 2958, 1603, 1583, 1452, 1387, 1358, 1215, 1194, 1051, 976, 955, 787, 737 cm^–1^; HRMS (ESI) *m*/*z* [M + Na]^+^ calcd for C_17_H_19_NNaO_2_ 292.1308, found 292.1306.

#### *N*-(4-Methoxybenzyl)-2-[(oxetan-3-yloxy)methyl]aniline (**1b**)

The product was obtained by column chromatography (silica, 1/1 hexane/EtOAc) as a yellow oil in 74% (309 mg) yield: ^1^H NMR (400 MHz, CDCl_3_) δ 7.34–7.28 (m, 2H), 7.21 (td, *J* = 8.0, 1.6 Hz, 1H), 7.03 (dd, *J* = 7.5, 1.6 Hz, 1H), 6.93–6.88 (m, 2H), 6.70–6.63 (m, 2H), 5.02 (s, 1H), 4.67–4.62 (m, 2H), 4.62–4.57 (m, 1H), 4.57–4.52 (m, 2H), 4.50 (s, 2H), 4.33 (s, 2H), 3.81 (s, 3H); ^13^C{^1^H} NMR (101 MHz, CDCl_3_) δ 158.9, 147.6, 131.4, 130.1, 129.9, 128.7 (2C), 121.0, 116.8, 114.2 (2C), 111.1, 78.9 (2C), 71.6, 70.8, 55.4, 47.3; IR (ATR) 3365, 2958, 2925, 2873, 1601, 1583, 1421, 1390, 1242, 1182, 1034, 1001, 976, 955, 802, 756 cm^–1^; HRMS (ESI) *m*/*z* [M + H]^+^ calcd for C_18_H_21_NNaO_3_ 322.1414, found 322.1414.

#### *N*-(4-Methylbenzyl)-2-[(oxetan-3-yloxy)methyl]aniline (**1c**)

The product was obtained by column chromatography (silica, 1/1 hexane/EtOAc) as a yellow oil in 81% (320 mg) yield: ^1^H NMR (400 MHz, CDCl_3_) δ 7.27 (d, *J* = 8.2 Hz, 2H), 7.23–7.07 (m, 4H), 7.03 (dd, *J* = 7.6, 1.6 Hz, 1H), 6.67 (dd, *J* = 7.4, 6.3 Hz, 2H), 4.69–4.62 (m, 2H), 4.62–4.56 (m, 1H), 4.57–4.52 (m, 2H), 4.51 (s, 2H), 4.36 (s, 2H), 2.35 (s, 3H); ^13^C{^1^H} NMR (101 MHz, CDCl_3_) δ 147.5, 136.9, 136.3, 130.1, 129.9, 129.5 (2C), 127.4 (2C), 121.1, 116.8, 111.1, 78.9 (2C), 71.6, 70.8, 47.7, 21.2; IR (ATR) 3415, 2945, 2920, 1599, 1581, 1435, 1329, 1296, 1178, 1159, 1109, 995, 962, 804, 723 cm^–1^; HRMS (ESI) *m*/*z* [M + H]^+^ calcd for C_18_H_22_NO_2_ 284.1645, found 284.1648.

#### *N*-(4-Nitrobenzyl)-2-[(oxetan-3-yloxy)methyl]aniline (**1d**)

The product was obtained by column chromatography (silica, 1/1 hexane/EtOAc) as a yellow oil in 86% (377 mg) yield: ^1^H NMR (400 MHz, CDCl_3_) δ 8.23–8.18 (m, 2H), 7.53 (d, *J* = 8.9 Hz, 2H), 7.15 (td, *J* = 7.9, 1.5 Hz, 1H), 7.05 (dd, *J* = 7.4, 1.5 Hz, 1H), 6.69 (td, *J* = 7.4, 1.0 Hz, 1H), 6.47 (d, *J* = 8.0 Hz, 1H), 5.29 (t, *J* = 5.5 Hz, 1H), 4.71–4.67 (m, 2H), 4.62 (ddd, *J* = 11.0, 9.9, 4.7 Hz, 1H), 4.58–4.53 (m, 6H); ^13^C{^1^H} NMR (101 MHz, CDCl_3_) δ 147.45, 147.3, 146.8, 130.2, 129.4, 127.7, 124.1, 123.8, 121.5, 117.6, 111.1, 78.8 (2C), 71.9, 70.9, 47.2; IR (ATR) 3400, 2947, 2877, 2848, 1604, 1597, 1427, 1342, 1327, 1244, 1092, 1078, 999, 966, 924, 843, 791 cm^–1^; HRMS (ESI) *m*/*z* [M + H]^+^ calcd for C_17_H_18_N_2_NaO_4_ 337.1159, found 337.1157.

#### 4-[({2-[(Oxetan-3-yloxy)methyl]phenyl}amino)methyl]benzonitrile (**1e**)

The product was obtained by column chromatography (silica, 1/1 hexane/EtOAc) as a yellow oil in 82% (337 mg) yield: ^1^H NMR (400 MHz, CDCl_3_) δ 7.65–7.59 (m, 2H), 7.50–7.45 (m, 2H), 7.15 (td, *J* = 7.8, 1.7 Hz, 1H), 7.04 (dd, *J* = 7.4, 1.6 Hz, 1H), 6.69 (td, *J* = 7.4, 1.1 Hz, 1H), 6.47 (dd, *J* = 8.1, 1.1 Hz, 1H), 5.29 (brs, 1H), 4.71–4.66 (m, 2H), 4.65–4.58 (m, 1H), 4.57–4.53 (m, 4H), 4.50 (s, 2H); ^13^C{^1^H} NMR (101 MHz, CDCl_3_) δ 146.8, 145.3, 132.6 (2C), 130.1 (2C), 127.7 (2C), 121.4, 118.9, 117.5, 111.1, 78.8 (2C), 71.9, 70.9, 47.4; IR (ATR) 3404, 3047, 2871, 2225, 1604, 1587, 1414, 1387, 1273, 1182, 1053, 968, 926, 854, 750 cm^–1^; HRMS (APCI) *m*/*z* [M + H]^+^ calcd for C_18_H_19_N_2_O_2_ 295.1441, found 295.1433.

#### 2-[(Oxetan-3-yloxy)methyl]-*N*-[4-(trifluoromethyl)benzyl]aniline (**1f**)

The product was obtained by column chromatography (silica, 1/1 hexane/EtOAc) as a yellow oil in 82% (470 mg) yield: ^1^H NMR (400 MHz, CDCl_3_) δ 7.60 (d, *J* = 8.0 Hz, 2H), 7.54–7.43 (m, 2H), 7.17 (td, *J* = 7.7, 1.6 Hz, 1H), 7.04 (dd, *J* = 7.3, 1.6 Hz, 1H), 6.69 (td, *J* = 7.4, 1.1 Hz, 1H), 6.55 (dd, *J* = 8.2, 1.1 Hz, 1H), 5.25 (s, 1H), 4.69–4.65 (m, 2H), 4.62 (ddd, *J* = 10.5, 5.4, 4.5 Hz, 1H), 4.57–4.53 (m, 4H), 4.49 (s, 2H); ^13^C{^1^H} NMR (101 MHz, CDCl_3_) δ 147.1, 143.8, 130.2, 130.1, 129.6 (q, *J* = 32.4 Hz, 1C), 127.4, 125.8 (q, *J* = 3.8 Hz, 1C), 124.3 (q, *J* = 272.0 Hz, 1C) 121.3, 117.3, 111.1, 78.8, 71.8, 70.9, 47.3; ^19^F NMR (376 MHz, CDCl_3_) δ −62.4 (s); IR (ATR) 3410, 2951, 2873, 1587, 1516, 1464, 1323, 1286, 1117, 1109, 970, 926, 852, 748 cm^–1^; HRMS (ESI) *m*/*z* [M + H]^+^ calcd for C_18_H_19_F_3_NO_2_ 338.1362, found 338.1363.

#### *N*-(4-Fluorobenzyl)-2-[(oxetan-3-yloxy)methyl]aniline (**1g**)

The product was obtained by column chromatography (silica, 1/1 hexane/EtOAc) as a yellow oil in 69% (277 mg) yield: ^1^H NMR (400 MHz, CDCl_3_) δ 7.34 (tt, *J* = 6.9, 3.4 Hz, 2H), 7.20 (td, *J* = 8.0, 1.6 Hz, 1H), 7.04 (tt, *J* = 6.6, 2.2 Hz, 3H), 6.68 (td, *J* = 7.4, 1.0 Hz, 1H), 6.62 (d, *J* = 8.1 Hz, 1H), 5.12 (s, 1H), 4.68–4.63 (m, 2H), 4.63–4.56 (m, 1H), 4.56–4.52 (m, 2H), 4.52 (s, 2H), 4.37 (s, 2H); ^13^C{^1^H} NMR (101 MHz, CDCl_3_) δ 162.15 (d, *J* = 245.1 Hz, 1C), 147.3, 135.11 (d, *J* = 3.2 Hz, 1C), 130.1, 130.0, 128.9 (d, *J* = 8.0 Hz, 2C), 121.2, 117.1, 115.61 (d, *J* = 21.4 Hz, 2C), 111.1, 78.8 (2C), 71.7, 70.8, 47.1; ^19^F NMR (376 MHz, CDCl_3_) δ −115.58 (ddd, *J* = 14.0, 8.7, 5.4 Hz); IR (ATR) 3359, 2974, 2952, 2939, 1601, 1585, 1508, 1414, 1385, 1259, 1240, 1221, 1105, 1095, 960, 926, 881, 860, 758, cm^–1^; HRMS (ESI) *m*/*z* [M + H]^+^ calcd for C_17_H_19_FNO_2_ 288.1394, found 288.1400.

#### *N*-(4-Bromobenzyl)-2-[(oxetan-3-yloxy)methyl]aniline (**1h**)

The product was obtained by column chromatography (silica, 1/1 hexane/EtOAc) as a yellow oil in 75% (552 mg) yield: ^1^H NMR (400 MHz, CDCl_3_) δ 7.46 (d, *J* = 8.4 Hz, 2H), 7.24 (d, *J* = 8.4 Hz, 2H), 7.21–7.15 (m, 1H), 7.04 (dd, *J* = 7.3, 1.2 Hz, 1H), 6.70 (t, *J* = 7.3 Hz, 1H), 6.59 (d, *J* = 8.1 Hz, 1H), 5.30 (brs, 1H), 4.66 (t, *J* = 5.9 Hz, 2H), 4.60 (dt, *J* = 10.7, 5.1 Hz, 1H), 4.57–4.53 (m, 2H), 4.52 (s, 2H), 4.37 (s, 2H); ^13^C{^1^H} NMR (101 MHz, CDCl_3_) δ 146.8, 138.3, 131.9 (2C), 130.1, 130.1, 129.1 (2C), 121.6, 121.1, 117.6, 111.6, 78.8 (2C), 71.8, 70.8, 47.5; IR (ATR) 3365, 2983, 2952, 2939, 2918, 1601, 1583, 1512, 1487, 1385, 1356, 1323, 1242, 1209, 1190, 972, 958, 926, 839, 808, 789 cm^–1^; HRMS (ESI) *m*/*z* (M + H)^+^ calcd for C_17_H_19_BrNO_2_ 348.0594, found 348.0592.

#### *N*-(3-Bromobenzyl)-2-[(oxetan-3-yloxy)methyl]aniline (**1i**)

The product was obtained by column chromatography (silica, 1/1 hexane/EtOAc) as a yellow oil in 76% (559 mg) yield: ^1^H NMR (400 MHz, CDCl_3_) δ 7.52 (s, 1H), 7.40 (d, *J* = 7.8 Hz, 1H), 7.30 (d, *J* = 7.9 Hz, 1H), 7.24–7.16 (m, 2H), 7.04 (dd, *J* = 7.3, 1.4 Hz, 1H), 6.70 (t, *J* = 7.4 Hz, 1H), 6.59 (d, *J* = 8.1 Hz, 1H), 4.72–4.66 (m, 2H), 4.65–4.59 (m, 1H), 4.59–4.55 (m, 2H), 4.53 (s, 2H), 4.39 (s, 2H); ^13^C{^1^H} NMR (101 MHz, CDCl_3_) δ 147.1, 142.0, 130.4, 130.4, 130.3, 130.2, 130.1, 125.8, 122.9, 121.2, 117.2, 111.1, 78.8 (2C), 71.6, 70.8, 47.2; IR (ATR) 3404, 2945, 2870, 1606, 1587, 1427, 1387, 1358, 1182, 1122, 970, 926, 858, 779 cm^–1^; HRMS (ESI) *m*/*z* [M + H]^+^ calcd for C_17_H_19_BrNNO_2_ 348.0594, found 348.0591

#### *N*-(2-Bromobenzyl)-2-[(oxetan-3-yloxy)methyl]aniline (**1j**)

Product **14** was obtained by column chromatography (silica, 1/1 hexane/EtOAc) as a yellow oil in 76% (559 mg) yield: ^1^H NMR (400 MHz, CDCl_3_) δ 7.59 (dd, *J* = 7.9, 1.2 Hz, 1H), 7.38 (dd, *J* = 7.6, 1.6 Hz, 1H), 7.30–7.24 (m, 2H), 7.17 (dtd, *J* = 17.7, 7.9, 1.7 Hz, 2H), 7.04 (dd, *J* = 7.4, 1.5 Hz, 1H), 6.69 (td, *J* = 7.4, 1.0 Hz, 1H), 6.59 (d, *J* = 8.0 Hz, 1H), 5.43 (s, 1H), 4.69–4.64 (m, 2H), 4.64–4.60 (m, 1H), 4.60–4.56 (m, 2H), 4.53 (s, 2H), 4.47 (s, 2H); ^13^C{^1^H} NMR (101 MHz, CDCl_3_) δ 147.0, 138.0, 133.0, 130.1, 130.0, 129.2, 128.9, 127.7, 123.5, 121.3, 117.2, 111.3, 78.8 (2C), 71.6, 70.8, 48.1; IR (ATR) 3408, 2945, 2870, 1606, 1585, 1568, 1441, 1387, 1358, 1184, 1163, 968, 926, 858, 791 cm^–1^; HRMS (ESI) *m*/*z* [M + H]^+^ calcd for C_17_H_19_BrNO_2_ 348.0594, found 348.0593.

#### *N*-(2-Nitrobenzyl)-2-[(oxetan-3-yloxy)methyl]aniline (**1k**)

Product **8** was obtained by column chromatography (silica, 1/1 hexane/EtOAc) as a yellow oil in 89% (378 mg) yield: ^1^H NMR (400 MHz, CDCl_3_) δ 8.09–8.01 (m, 1H), 7.63 (d, *J* = 7.6 Hz, 1H), 7.60–7.55 (m, 1H), 7.43 (t, *J* = 7.1 Hz, 1H), 7.19–7.10 (m, 1H), 7.09–7.01 (m, 1H), 6.67 (t, *J* = 7.1 Hz, 1H), 6.48 (d, *J* = 8.1 Hz, 1H), 5.36 (s, 1H), 4.80 (s, 2H), 4.74–4.56 (m, 5H), 4.53 (s, 2H); ^13^C{^1^H} NMR (101 MHz, CDCl_3_) δ 148.5, 146.8, 135.5, 133.7, 130.1, 130.1, 129.8, 128.2, 125.3, 121.3, 117.3, 110.9, 78.7 (2C), 71.7, 70.7, 45.2; IR (ATR) 3431, 3384, 2949, 2924, 2877, 1599, 1581, 1446, 1360, 1259, 1113, 935, 904, 864, 758 cm^–1^; HRMS (ESI) *m*/*z* [M + H]^+^ calcd for C_17_H_18_N_2_NaO_4_ 337.1159, found 337.1158.

#### *N*-(Naphthalen-2-ylmethyl)-2-[(oxetan-3-yloxy)methyl]aniline (**1l**)

The product was obtained by column chromatography (silica, 1/1 hexane/EtOAc) as a yellow oil in 93% (414 mg) yield: ^1^H NMR (400 MHz, CDCl_3_) δ 7.90–7.77 (m, 4H), 7.55–7.43 (m, 3H), 7.19 (td, *J* = 8.1, 1.5 Hz, 1H), 7.05 (dd, *J* = 7.3, 1.3 Hz, 1H), 6.72–6.64 (m, 2H), 5.21 (s, 1H), 4.69–4.64 (m, 2H), 4.64–4.60 (m, 1H), 4.60–4.51 (m, 6H); ^13^C{^1^H} NMR (101 MHz, CDCl_3_) δ 147.6, 136.9, 133.6, 132.9, 130.2, 129.9, 128.6, 127.9, 127.8, 126.3, 125.9 (2C), 125.6, 121.1, 116.9, 111.2, 78.9 (2C), 71.7, 70.9, 48.0; IR (ATR) 3394, 2945, 2870, 1585, 1385, 1358, 1180, 1126, 970, 926, 891, 748 cm^–1^; HRMS (ESI) *m*/*z* [M + Na]^+^ calcd for C_21_H_21_NNaO_2_ 342.1464, found 342.1462.

#### 2-[(Oxetan-3-yloxy)methyl]-*N*-(thiophen-2-ylmethyl)aniline (**1m**)

The product was obtained by column chromatography (silica, 1/1 hexane/EtOAc) as a yellow oil in 73% (280 mg) yield: ^1^H NMR (400 MHz, CDCl_3_) δ 7.27–7.18 (m, 2H), 7.07–7.00 (m, 2H), 6.98 (dd, *J* = 5.1, 3.5 Hz, 1H), 6.74 (dd, *J* = 8.2, 1.0 Hz, 1H), 6.70 (td, *J* = 7.4, 1.1 Hz, 1H), 5.22 (brs, 1H), 4.71–4.57 (m, 3H), 4.58 (s, 2H), 4.60–4.51 (m, 2H), 4.50 (s, 2H); ^13^C{^1^H} NMR (101 MHz, CDCl_3_) δ 147.0, 143.1, 130.1, 130.0, 127.0 (2C), 125.1, 124.7, 117.4, 111.3, 78.8 (2C), 71.6, 70.8, 43.1; IR (ATR) 3390, 2945, 2870, 1585, 1512, 1309, 1259, 1105, 966, 926, 850, 733 cm^–1^; HRMS (ESI) *m*/*z* [M + Na]^+^ calcd for C_15_H_17_NNaO_2_S 298.0872, found 298.0873.

#### *N*-Allyl-2-[(oxetan-3-yloxy)methyl]aniline (**1n**)

The product was obtained by column chromatography (silica, 1/1 hexane/EtOAc) as a yellow oil in 67% (205 mg) yield: ^1^H NMR (400 MHz, CDCl_3_) δ 7.23 (td, *J* = 8.0, 1.6 Hz, 1H), 7.02 (d, *J* = 7.5 Hz, 1H), 6.70–6.65 (m, 2H), 6.05–5.95 (m, 1H), 5.30 (dq, *J* = 17.2, 1.7 Hz, 1H), 5.19 (dq, *J* = 10.3, 1.5 Hz, 1H), 4.68–4.64 (m, 2H), 4.64–4.54 (m, 3H), 4.50 (s, 2H), 3.84 (d, *J* = 5.2 Hz, 2H); ^13^C{^1^H} NMR (101 MHz, CDCl_3_) δ 147.3, 135.3, 130.1, 130.0, 121.2, 116.9, 116.3, 111.2, 78.9 (2C), 71.6, 70.8, 46.1; IR (ATR) 3396, 2949, 2871, 1682, 1645, 1585, 1514, 1387, 1360, 1182, 968, 924, 860, 748 cm^–1^; HRMS (ESI) *m*/*z* [M + H]^+^ calcd for C_13_H_18_NO_2_ 220.1332, found 220.1334.

#### *N*-Methyl-2-[(oxetan-3-yloxy)methyl]aniline (**1o**)

The product was obtained by column chromatography (silica, 1/1 hexane/EtOAc) as a yellow oil in 78% (210 mg) yield: ^1^H NMR (400 MHz, CDCl_3_) δ 7.26 (dd, *J* = 15.5, 1.6 Hz, 1H), 7.00 (dd, *J* = 7.7, 1.7 Hz, 1H), 6.71–6.67 (m, 1H), 6.65 (d, *J* = 7.7 Hz, 2H), 4.69–4.63 (m, 2H), 4.59–4.53 (m, 3H), 4.46 (s, 2H), 2.89 (s, 3H); ^13^C{^1^H} NMR (101 MHz, CDCl_3_) δ 148.8, 130.2 (2C), 129.9, 116.5, 110.1, 78.9 (2C), 71.7, 70.9, 30.5; IR (ATR) 3408, 2945, 2871, 2816, 2789, 1606, 1585, 1452, 1388, 1267, 1049, 1007, 968, 947, 928, 860, 748 cm^–1^; HRMS (ESI) *m*/*z* [M + Na]^+^ calcd for C_11_H_15_NNaO_2_ 216.0995, found 216.0995.

#### *tert*-Butyl {2-[(Oxetan-3-yloxy)methyl]phenyl}carbamate (**1p**)

The product was obtained by column chromatography (silica, 1/1 hexane/EtOAc) as a yellow oil in 75% (292 mg) yield: ^1^H NMR (400 MHz, CDCl_3_) δ 7.95 (d, *J* = 8.1 Hz, 1H), 7.48 (s, 1H), 7.36–7.29 (m, 1H), 7.10 (dd, *J* = 7.5, 1.4 Hz, 1H), 6.99 (td, *J* = 7.4, 1.1 Hz, 1H), 4.67 (dtd, *J* = 7.9, 4.4, 2.2 Hz, 2H), 4.63–4.56 (m, 3H), 4.49 (s, 2H), 1.53 (s, 9H); ^13^C{^1^H} NMR (101 MHz, CDCl_3_) δ 153.1, 138.3, 129.7, 129.4, 125.4, 123.0, 121.0, 80.6, 78.6 (2C), 72.0, 70.3, 28.5 (3C); IR (ATR) 3381, 2978, 2933, 2875, 1591, 1516, 1392, 1234, 1024, 955, 903, 858, 754 cm^–1^; HRMS (ESI) *m*/*z* [M + H]^+^ calcd for C_15_H_21_NNaO_4_ 302.1363, found 302.1359.

#### *N*-Benzyl-2-methyl-6-[(oxetan-3-yloxy)methyl]aniline (**1v**)

The product was obtained by column chromatography (silica, 1/1 hexane/EtOAc) as a yellow oil in 78% (308 mg) yield: ^1^H NMR (400 MHz, CDCl_3_) δ 7.33 (d, *J* = 3.7 Hz, 3H), 7.31–7.27 (m, 2H), 7.19–7.15 (m, 2H), 7.02 (dd, *J* = 7.6, 1.7 Hz, 1H), 6.92 (t, *J* = 7.5 Hz, 1H), 4.66–4.59 (m, 2H), 4.55–4.50 (m, 3H), 4.32 (s, 2H), 4.22 (s, 2H), 2.37 (s, 3H); ^13^C{^1^H} NMR (101 MHz, CDCl_3_) δ 139.3, 132.1, 129.3, 128.8, 128.4, 128.3, 128.3, 127.7, 127.3, 126.8, 125.5, 78.7 (2C), 71.8, 69.7, 57.1, 53.5, 18.8; IR (ATR) 3390, 2947, 2924, 2873, 1616, 1595, 1373, 1360, 1203, 1043, 970, 945, 862, 733 cm^–1^; HRMS (ESI) *m*/*z* [M + Na]^+^ calcd for C_18_H_21_NNaO_2_ 306.1464, found 306.1465.

#### *N*-Benzyl-5-chloro-2-[(oxetan-3-yloxy)methyl]aniline (**1w**)

The product was obtained by column chromatography (silica, 1/1 hexane/EtOAc) as a yellow oil in 68% (288 mg) yield: ^1^H NMR (400 MHz, CDCl_3_) δ 7.38–7.34 (m, 4H), 7.34–7.27 (m, 2H), 6.93 (d, *J* = 8.4 Hz, 1H), 6.65–6.61 (m, 2H), 4.65 (td, *J* = 5.8, 1.0 Hz, 2H), 4.60–4.54 (m, 1H), 4.54–4.50 (m, 2H), 4.46 (s, 2H), 4.36 (s, 2H); ^13^C{^1^H} NMR (101 MHz, CDCl_3_) δ 148.5, 138.7, 135.9, 130.8, 128.9 (2C), 127.6, 127.5 (2C), 119.6, 116.6, 111.1, 78.8 (2C), 71.7, 70.2, 47.8; IR (ATR) 3396, 2947, 2870, 1577, 1510, 1471, 1325, 1281, 1180, 1009, 970, 928, 860, 735 cm^–1^; HRMS (ESI) *m*/*z* [M + Na]^+^ calcd for C_17_H_18_ClNNaO_2_ 326.0918, found 326.0917.

#### *N*-Benzyl-5-fluoro-2-[(oxetan-3-yloxy)methyl]aniline (**1x**)

The product was obtained by column chromatography (silica, 1/1 hexane/EtOAc) as a yellow oil in 69% (277 mg) yield: ^1^H NMR (400 MHz, CDCl_3_) δ 7.36 (d, *J* = 4.3 Hz, 4H), 7.33–7.27 (m, 2H), 6.98–6.93 (m, 1H), 6.35 (s, 1H), 6.34–6.30 (m, 1H), 4.65 (td, *J* = 5.7, 1.0 Hz, 2H), 4.61–4.56 (m, 1H), 4.56–4.51 (m, 2H), 4.47 (s, 2H), 4.36 (s, 2H); ^13^C{^1^H} NMR (101 MHz, CDCl_3_) δ 164.7 (d, *J* = 244.1 Hz, 1C), 149.30 (d, *J* = 11.2 Hz, 1C), 138.7, 131.1 (d, *J* = 10.5 Hz, 1C), 128.8, 128.9 (2C), 127.6, 127.4 (2C), 102.9 (d, *J* = 21.8 Hz, 1C), 98.5 (d, *J* = 26.4 Hz, 1C), 78.8 (2C), 71.6, 70.2, 47.8; ^19^F NMR (376 MHz, CDCl_3_) δ −111.44 (ddd, *J* = 11.6, 8.8, 6.4 Hz); IR (ATR) 3357, 2978, 2956, 2920, 2873, 1610, 1591, 1518, 1387, 1361, 1290, 1221, 1026, 1003, 976, 958, 831, 744 cm^–1^; HRMS (ESI) *m*/*z* [M + Na]^+^ calcd for C_17_H_18_FNNaO_2_ 310.1214, found 310.1214.

#### *N*-Benzyl-5-methyl-2-[(oxetan-3-yloxy)methyl]aniline (**1y**)

The product was obtained by column chromatography (silica, 1/1 hexane/EtOAc) as a yellow oil in 72% (106 mg) yield: ^1^H NMR (400 MHz, CDCl_3_) δ 7.41–7.33 (m, 4H), 7.32–7.27 (m, 1H), 6.91 (dd, *J* = 8.1, 4.3 Hz, 1H), 6.49 (dt, *J* = 4.4, 2.1 Hz, 2H), 5.06 (s, 1H), 4.66–4.62 (m, 2H), 4.61–4.56 (m, 1H), 4.56–4.51 (m, 2H), 4.48 (s, 2H), 4.39 (s, 2H), 2.27 (s, 3H); ^13^C{^1^H} NMR (101 MHz, CDCl_3_) δ 147.4, 140.1, 139.5, 129.9, 128.8, 128.3, 127.5, 127.4, 118.4, 117.7, 111.9, 78.9 (2C), 71.4, 70.6, 47.9, 21.9; IR (ATR) 3400, 3028, 2920, 1614, 1522, 1300, 1122, 970, 739, 698 cm^–1^; HRMS (ESI) *m*/*z* [M + Na]^+^ calcd for C_18_H_21_NNaO_2_ 306.1464, found 306.1465.

#### *N*-Benzyl-5-methoxy-2-[(oxetan-3-yloxy)methyl]aniline (**1z**)

The product was obtained by column chromatography (silica, 1/1 hexane/EtOAc) as a yellow oil in 83% (119 mg) yield: ^1^H NMR (400 MHz, CDCl_3_) δ 7.36 (d, *J* = 7.1 Hz, 4H), 7.29 (d, *J* = 6.2 Hz, 1H), 6.93 (d, *J* = 7.8 Hz, 1H), 6.19 (d, *J* = 7.8 Hz, 2H), 5.08 (s, 1H), 4.64 (dd, *J* = 6.6, 5.0 Hz, 2H), 4.58 (q, *J* = 4.8 Hz, 1H), 4.53 (dd, *J* = 6.8, 4.4 Hz, 2H), 4.46 (s, 2H), 4.37 (s, 2H), 3.73 (s, 2H); ^13^C{^1^H} NMR (101 MHz, CDCl_3_) δ 161.6, 148.9, 139.3, 130.9, 128.8, 128.8, 128.4, 127.4, 127.4, 114.2, 100.9, 98.1, 78.9 (2C), 71.3, 70.4, 55.2, 47.9; IR (ATR) 3408, 3086, 2870, 1614, 1522, 1356, 1122, 970, 739, 698 cm^–1^; HRMS (ESI) *m*/*z* [M + Na]^+^ calcd for C_18_H_21_NNaO_3_ 322.1414, found 322.1416.

### Synthesis of Substituted Amines for Enantiomeric Desymmetrization (**1q–1u**)

A 5 mL vial equipped with a magnetic stirrer was charged with amine (1 equiv), Pd(OAc)_2_ (10 mol %), PPh_3_ (20 mol %), Cs_2_CO_3_ (2 equiv), and toluene (3 mL) at room temperature. The resulting solution was refluxed for 24–72 h and monitored by TLC. After the starting material had been fully consumed, the reaction mixture was filtered through Celite and evaporated under reduced pressure, and the residue was purified by silica gel column chromatography (5/1 hexane/EtOAc) to afford the desired product.

#### 2-[(Oxetan-3-yloxy)methyl]-*N*-phenylaniline (**1q**)

The product was obtained by column chromatography (silica, 1/1 hexane/EtOAc) as a yellow oil in 82% (356 mg) yield: ^1^H NMR (400 MHz, CDCl_3_) δ 7.36 (dd, *J* = 8.1, 0.9 Hz, 1H), 7.31–7.26 (m, 2H), 7.24 (dd, *J* = 8.1, 1.6 Hz, 1H), 7.17 (dd, *J* = 7.5, 1.4 Hz, 1H), 7.11–7.06 (m, 2H), 6.95 (tt, *J* = 7.6, 1.1 Hz, 1H), 6.89 (td, *J* = 7.4, 1.2 Hz, 1H), 4.74–4.67 (m, 2H), 4.67–4.59 (m, 2H), 4.52 (s, 1H); ^13^C{^1^H} NMR (101 MHz, CDCl_3_) δ 143.3, 142.9, 130.4, 129.7, 129.5, 125.3, 121.2, 120.6, 118.4, 117.1, 78.8, 72.0, 70.6; IR (ATR) 3383, 2947, 2870, 1593, 1510, 1423, 1387, 1254, 1178, 1007, 968, 931, 818 cm^–1^; HRMS (ESI) *m*/*z* [M + Na]^+^ calcd for C_16_H_17_NNaO_2_ 278.1151, found 278.1152.

#### *N*-{2-[(Oxetan-3-yloxy)methyl]phenyl}naphthalen-1-amine (**1r**)

The product was obtained by column chromatography (silica, 1/1 hexane/EtOAc) as a yellow oil in 92% (391 mg) yield: ^1^H NMR (400 MHz, CDCl_3_) δ 8.01–7.95 (m, 1H), 7.88 (dd, *J* = 6.8, 2.7 Hz, 1H), 7.57 (d, *J* = 8.0 Hz, 1H), 7.54–7.46 (m, 2H), 7.42 (t, *J* = 7.8 Hz, 1H), 7.36 (dd, *J* = 7.5, 1.2 Hz, 1H), 7.20 (ddd, *J* = 7.8, 6.6, 1.7 Hz, 2H), 7.13 (dd, *J* = 8.6, 1.2 Hz, 1H), 6.87 (td, *J* = 7.3, 1.2 Hz, 1H), 4.80–4.69 (m, 5H), 4.64 (s, 2H); ^13^C{^1^H} NMR (101 MHz, CDCl_3_) δ 144.7, 138.5, 134.9, 130.3, 129.8, 128.8, 127.5, 126.3, 126.1, 125.9, 124.4, 122.8, 121.6, 120.0, 116.7, 115.7, 78.9, 72.2, 70.9; IR (ATR) 3694, 2952, 2918, 2870, 2850, 1606, 1577, 1423, 1358, 1298, 1032, 1007, 970, 930, 860, 735 cm^–1^; HRMS (ESI) *m*/*z* [M + Na]^+^ calcd for C_20_H_19_NNaO_2_ 328.1308, found 328.1308.

#### *N*-2-[(Oxetan-3-yloxy)methyl][4-(trifluoromethyl)phenyl]aniline (**1s**)

The product was obtained by column chromatography (silica, 1/1 hexane/EtOAc) as a yellow oil in 86% (388 mg) yield: ^1^H NMR (400 MHz, CDCl_3_) δ 7.49 (d, *J* = 8.5 Hz, 2H), 7.42 (d, *J* = 7.6 Hz, 1H), 7.31 (td, *J* = 7.8, 1.5 Hz, 1H), 7.23 (dd, *J* = 7.5, 1.4 Hz, 1H), 7.06 (d, *J* = 8.4 Hz, 2H), 7.01 (td, *J* = 7.4, 1.1 Hz, 1H), 6.86 (s, 1H), 4.72 (td, *J* = 5.4, 2.0 Hz, 2H), 4.67–4.57 (m, 3H), 4.50 (s, 2H); ^13^C{^1^H} NMR (101 MHz, CDCl_3_) δ 146.5, 141.4, 130.6, 129.8, 127.3, 126.9 (q, *J* = 3.8 Hz, 1C), 124.71 (d, *J* = 270.6, 1C Hz), 126.1, 122.6, 121.99 (d, *J* = 32.8 Hz, 1C), 119.4, 116.0 (2C), 78.7 (2C), 72.2, 70.3; ^19^F NMR (376 MHz, CDCl_3_) δ −61.48; IR 3381, 2952, 2922, 2873, 1618, 1595, 1525, 1406, 1317, 1265, 1065, 970, 933, 862, 737 cm^–1^; HRMS (ESI) *m*/*z* [M + Na]^+^ calcd for C_17_H_16_F_3_NNaO_2_ 346.1025, found 346.1018.

#### *N*-(4-Methoxyphenyl)-2-[(oxetan-3-yloxy)methyl]aniline (**1t**)

The product was obtained by column chromatography (silica, 1/1 hexane/EtOAc) as a yellow oil in 45% (179 mg) yield: ^1^H NMR (400 MHz, CDCl_3_) δ 7.18 (ddd, *J* = 8.7, 7.3, 1.6 Hz, 1H), 7.10 (ddd, *J* = 8.2, 6.6, 1.3 Hz, 2H), 7.08–7.05 (m, 2H), 6.91–6.85 (m, 2H), 6.78 (td, *J* = 7.3, 1.3 Hz, 1H), 6.47 (s, 1H, NH), 4.74–4.66 (m, 2H), 4.66–4.59 (m, 3H), 4.54 (s, 2H), 3.81 (s, 3H, -CH_3_); ^13^C{^1^H} NMR (101 MHz, CDCl_3_) δ 155.4, 145.2, 135.6, 130.3, 129.8, 122.4 (2C), 119.2, 114.9 (2C), 114.8, 78.9 (2C), 71.9, 70.8, 55.8; IR (ATR) 3383, 2999, 2949, 2870, 2833, 1593, 1583, 1404, 1358, 1180, 1009, 968, 930, 823, 748 cm^–1^; HRMS (ESI) *m*/*z* [M + Na]^+^ calcd for C_17_H_19_NNaO_3_ 308.1257, found 308.1255.

#### *N*-(4-Bromophenyl)-2-[(oxetan-3-yloxy)methyl]aniline (**1u**)

The product was obtained by column chromatography (silica, 1/1 hexane/EtOAc) as a yellow oil in 68% (127 mg) yield: ^1^H NMR (400 MHz, CDCl_3_) δ 7.38–7.34 (m, 1H), 7.34–7.27 (m, 1H), 7.20–7.15 (m, 1H), 6.98–6.87 (m, 1H), 6.65 (s, 1H), 4.75–4.67 (m, 1H), 4.66–4.57 (m, 1H), 4.50 (s, 1H); ^13^C{^1^H} NMR (101 MHz, CDCl_3_) δ 142.7, 142.2, 132.4 (2C), 130.5, 129.8, 125.8, 121.3, 119.6 (2C), 117.5, 112.9, 78.8 (2C), 72.1, 70.4; IR (ATR) 3386, 3037, 2852, 1579, 1489, 1230, 1072, 812, 775 cm^–1^; HRMS (ESI) *m*/*z* [M + Na]^+^ calcd for C_16_H_16_BrNNaO_2_ 356.0257, found 356.0255.

### General Procedure for Enantioselective Desymmetrization of Oxetane Derivatives (**2a–2y**)

To a 1 mL vial equipped with a magnetic stirrer were added an oxetane derivative (0.05 mmol, 1 equiv), (*R*)-**CPA-8** (0.005 mmol, 10 mol %), and *p*-xylene (0.5 mL, 0.1 M) at room temperature. The resulting solution was stirred at 45 °C for 24–72 h and monitored by TLC. After the starting material had been fully consumed, the reaction mixture was evaporated under reduced pressure, and the residue was purified by silica gel column chromatography (1/1 hexane/EtOAc) to afford the desired product

#### (*R*)-(1-Benzyl-1,2,3,5-tetrahydrobenzo[*e*][1,4]oxazepin-3-yl)methanol (**2a**)

The product was obtained by column chromatography (silica, 1/1 hexane/EtOAc) as a yellow oil in 85% (11.5 mg) yield: ^1^H NMR (400 MHz, CDCl_3_) δ 7.42–7.38 (m, 2H), 7.35 (ddd, *J* = 7.6, 6.7, 1.3 Hz, 2H), 7.31–7.26 (m, 2H), 7.26–7.23 (m, 1H), 7.06 (dd, *J* = 8.0, 1.1 Hz, 1H), 6.97 (td, *J* = 7.4, 1.1 Hz, 1H), 4.86–4.69 (m, 2H), 4.59 (d, *J* = 13.9 Hz, 1H), 4.22 (d, *J* = 13.9 Hz, 1H), 3.70–3.61 (m, 1H), 3.49–3.36 (m, 2H), 3.09 (dd, *J* = 13.8, 1.9 Hz, 1H), 2.73 (dd, *J* = 13.8, 9.4 Hz, 1H), 1.88 (s, 1H); ^13^C{^1^H} NMR (101 MHz, CDCl_3_) δ 138.6, 132.0, 129.7, 128.9 (2C), 128.6 (2C), 128.4, 127.4, 121.5, 117.3, 112.5, 80.7, 72.7, 63.8, 58.1, 55.5; IR (ATR) 3357, 2947, 2947, 2924, 2877, 2848, 1599, 1495, 1333, 1311, 1292, 1267, 1047, 962, 943, 827, 758 cm^–1^; HRMS (ESI) *m*/*z* [M + Na]^+^ calcd for C_17_H_19_NNaO_2_ 292.1308, found 292.1308; [α]^25^_D_ = −110.1° (*c* = 0.28, CHCl_3_); enantiomeric excess (ee) 92%; retention times *t*_major_ = 23.8 min and *t*_minor_ = 16.9 min determined by HPLC (Chiralpak column IA, 95/5 *n*-heptane/isopropanol, flow rate of 1.0 mL/min, 25 °C, λ = 209 nm).

#### (*R*)-[1-(4-Methoxybenzyl)-1,2,3,5-tetrahydrobenzo[*e*][1,4]oxazepin-3-yl]methanol (**2b**)

The product was obtained by column chromatography (silica, 1/1 hexane/EtOAc) as a yellow oil in 69% (10.3 mg) yield: ^1^H NMR (400 MHz, CDCl_3_) δ 7.33–7.27 (m, 3H), 7.26–7.22 (m, 1H), 7.06 (d, *J* = 8.0 Hz, 1H), 6.97 (t, *J* = 7.4 Hz, 1H), 6.91–6.86 (m, 2H), 4.85–4.68 (m, 2H), 4.51 (d, *J* = 13.5 Hz, 1H), 4.13 (d, *J* = 13.5 Hz, 1H), 3.82 (s, 3H), 3.67–3.56 (m, 1H), 3.42 (qd, *J* = 11.4, 5.4 Hz, 2H), 3.08 (d, *J* = 13.7 Hz, 1H), 2.67 (dd, *J* = 13.3, 9.8 Hz, 1H); ^13^C{^1^H} NMR (101 MHz, CDCl_3_) δ 159.1, 131.9, 129.8 (3C), 129.1, 121.7, 117.5, 114.1 (3C), 80.8, 72.8, 63.9, 57.5, 55.4, 55.3; IR (ATR) 3415, 2949, 2949, 2933, 2879, 2835, 1610, 1599, 1585, 1439, 1302, 1286, 1032, 962, 933, 818, 750 cm^–1^; HRMS (ESI) *m*/*z* [M + Na]^+^ calcd for C_18_H_21_NNaO_3_ 322.1414, found 322.1414; [α]^25^_D_ = −10.8° (*c* = 0.65, CHCl_3_); enantiomeric excess (ee) 88%; retention times *t*_major_ = 9.4 min and *t*_minor_ = 7.8 min determined by HPLC (Chiralpak column IA, 80/20 *n*-heptane/isopropanol, flow rate of 1.0 mL/min, 25 °C, λ = 250 nm).

#### (*R*)-[1-(4-Methylbenzyl)-1,2,3,5-tetrahydrobenzo[*e*][1,4]oxazepin-3-yl]methanol (**2c**)

The product was obtained by column chromatography (silica, 1/1 hexane/EtOAc) as a yellow oil in 70% (9.9 mg) yield: ^1^H NMR (400 MHz, CDCl_3_) δ 7.70–7.64 (m, 2H), 7.57–7.51 (m, 2H), 7.28–7.24 (m, 2H), 7.03–6.95 (m, 2H), 4.87–4.74 (m, 2H), 4.69 (d, *J* = 14.9 Hz, 1H), 4.32 (d, *J* = 14.8 Hz, 1H), 3.71 (dtd, *J* = 9.4, 4.2, 2.0 Hz, 1H), 3.58–3.40 (m, 2H), 3.06 (dd, *J* = 13.9, 2.0 Hz, 1H), 2.84 (dd, *J* = 13.9, 9.5 Hz, 1H), 1.28 (s, 3H); ^13^C{^1^H} NMR (101 MHz, CDCl_3_) δ 152.5, 137.1, 135.8, 131.8, 129.8, 129.4 (2C), 129.1, 128.5, 127.4, 121.4, 117.3, 80.9, 72.9, 64.0, 57.8, 55.4, 21.3; IR (ATR) 3394, 2924, 2873, 2852, 1599, 1585, 1514, 1394, 1361, 1215, 1178, 1003, 968, 933, 847, 748 cm^–1^; HRMS (ESI) *m*/*z* [M + H]^+^ calcd for C_18_H_21_NNaO_2_ 306.1464, found 306.1468; [α]^25^_D_ = −8.2° (*c* = 1.04, CHCl_3_); enantiomeric excess (ee) 92%; retention times *t*_major_ = 28.1 min and *t*_minor_ = 25.2 min determined by HPLC (Chiralpak column IG, 95/5 *n*-heptane/isopropanol, flow rate of 1.0 mL/min, 25 °C, λ = 250 nm).

#### (*R*)-[1-(4-Nitrobenzyl)-1,2,3,5-tetrahydrobenzo[*e*][1,4]oxazepin-3-yl]methanol (**2d**)

The product was obtained by column chromatography (silica, 1/1 hexane/EtOAc) as a yellow oil in 70% (14.8 mg) yield: ^1^H NMR (400 MHz, CDCl_3_) δ 8.29–8.13 (m, 2H), 7.58 (d, *J* = 8.8 Hz, 2H), 7.26 (d, *J* = 1.0 Hz, 1H), 7.24 (d, *J* = 7.5 Hz, 1H), 7.02–6.93 (m, 2H), 4.88–4.66 (m, 3H), 4.35 (d, *J* = 15.0 Hz, 1H), 3.71 (tdd, *J* = 9.4, 4.1, 1.9 Hz, 1H), 3.58–3.38 (m, 2H), 3.05 (dd, *J* = 13.8, 1.9 Hz, 1H), 2.84 (dd, *J* = 13.9, 9.5 Hz, 1H), 1.88 (dd, *J* = 7.2, 5.0 Hz, 1H); ^13^C{^1^H} NMR (101 MHz, CDCl_3_) δ 151.3, 147.5, 146.6, 131.9, 130.1, 129.1, 128.9 (2C), 124.1 (2C), 122.2, 117.2, 80.8, 72.90, 63.9, 57.8, 56.8; IR (ATR) 3350, 2945, 2879, 2848, 1597, 1518, 1437, 1344, 1263, 1227, 1014, 918, 852, 760 cm^–1^; HRMS (ESI) *m*/*z* [M + Na]^+^ calcd for C_17_H_18_N_2_NaO_4_ 337.1159, found 337.1160; [α]^25^_D_ = −16.0° (*c* = 0.75, CHCl_3_); enantiomeric excess (ee) 94%; retention times *t*_major_ = 13.9 min and *t*_minor_ = 11.9 min determined by HPLC (Chiralpak column IA, 80/20 *n*-heptane/isopropanol, flow rate of 1.0 mL/min, 25 °C, λ = 220 nm).

#### (*R*)-4-{[3-(Hydroxymethyl)-2,3-dihydrobenzo[*e*][1,4]oxazepin-1(5*H*)-yl]methyl}benzonitrile (**2e**)

The product was obtained by column chromatography (silica, 1/1 hexane/EtOAc) as a yellow oil in 71% (7.4 mg) yield: ^1^H NMR (400 MHz, CDCl_3_) δ 7.68–7.61 (m, 2H), 7.52 (d, *J* = 8.4 Hz, 2H), 7.23 (dd, *J* = 5.9, 1.6 Hz, 2H), 7.01–6.90 (m, 2H), 4.85–4.61 (m, 3H), 4.30 (d, *J* = 14.9 Hz, 1H), 3.69 (tdd, *J* = 9.4, 4.1, 1.9 Hz, 1H), 3.54–3.38 (m, 2H), 3.03 (dd, *J* = 13.8, 1.9 Hz, 1H), 2.82 (dd, *J* = 13.9, 9.5 Hz, 1H), 1.95–1.85 (m, 1H); ^13^C{^1^H} NMR (101 MHz, CDCl_3_) δ 137.9, 134.5, 130.8 (2C), 128.9 (2C), 128.6 (2C), 127.8 (2C), 121.5, 118.0, 80.7, 72.2, 63.9, 58.2, 55.6; IR (ATR) 3406, 2951, 2924, 2871, 2227, 1604, 1587, 1516, 1464, 1414, 1388, 1360, 1182, 1126, 968, 926, 818, 750 cm^–1^; HRMS (APCI) *m*/*z* [M + H]^+^ calcd for C_18_H_19_N_2_O_2_ 295.1441, found 295.1433; [α]^25^_D_ = −35.0° (*c* = 1.0, CHCl_3_); enantiomeric excess (ee) 93%; retention times *t*_major_ = 13.9 min and *t*_minor_ = 11.7 min determined by HPLC (Chiralpak column IA, 80/20 *n*-heptane/isopropanol, flow rate of 1.0 mL/min, 25 °C, λ = 254 nm).

#### (*R*)-{1-[4-(Trifluoromethyl)benzyl]-1,2,3,5-tetrahydrobenzo[*e*][1,4]oxazepin-3-yl}methanol (**2f**)

The product was obtained by column chromatography (silica, 1/1 hexane/EtOAc) as a yellow oil in 71% (12.0 mg) yield: ^1^H NMR (400 MHz, CDCl_3_) δ 7.61 (d, *J* = 8.2 Hz, 2H), 7.55–7.49 (m, 2H), 7.28 (d, *J* = 1.7 Hz, 1H), 7.24 (d, *J* = 1.7 Hz, 1H), 7.03–6.94 (m, 2H), 4.86–4.72 (m, 2H), 4.66 (d, *J* = 14.4 Hz, 1H), 4.30 (d, *J* = 14.4 Hz, 1H), 3.73–3.65 (m, 1H), 3.53–3.38 (m, 2H), 3.06 (dd, *J* = 13.8, 1.9 Hz, 1H), 2.80 (dd, *J* = 13.9, 9.5 Hz, 1H); ^13^C{^1^H} NMR (101 MHz, CDCl_3_) δ 151.7, 143.1, 131.9, 130.0, 129.1, 128.6, 125.7 (q, *J* = 3.8 Hz), 121.9, 117.3, 80.8, 72.9, 64.0, 57.9, 56.3; ^19^F NMR (376 MHz, CDCl_3_) δ −62.4; IR (ATR) 3427, 2927, 2881, 2850, 1618, 1601, 1495, 1321, 1284, 1259, 1159, 1016, 995, 937, 820 cm^–1^; HRMS (ESI) *m*/*z* [M + H]^+^ calcd for C_18_H_19_F_3_NO_2_ 338.1362, found 338.1359; [α]^25^_D_ = −21.1° (*c* = 0.9, CHCl_3_); enantiomeric excess (ee) 91%; retention times *t*_major_ = 7.9 min and *t*_minor_ = 6.5 min determined by HPLC (Chiralpak column IA, 80/20 *n*-heptane/isopropanol, flow rate of 1.0 mL/min, 25 °C, λ = 220 nm).

#### (*R*)-[1-(4-Fluorobenzyl)-1,2,3,5-tetrahydrobenzo[*e*][1,4]oxazepin-3-yl]methanol (**2g**)

The product was obtained by column chromatography (silica, 1/1 hexane/EtOAc) as a yellow oil in 75% (14.4 mg) yield: ^1^H NMR (400 MHz, CDCl_3_) δ 7.37 (dd, *J* = 8.6, 5.5 Hz, 2H), 7.28 (d, *J* = 1.6 Hz, 1H), 7.26–7.23 (m, 2H), 7.07–7.02 (m, 2H), 7.02–6.99 (m, 1H), 6.97 (dd, *J* = 7.4, 0.9 Hz, 1H), 4.84–4.68 (m, 2H), 4.55 (d, *J* = 13.7 Hz, 1H), 4.19 (d, *J* = 13.8 Hz, 1H), 3.64 (s, 1H), 3.44 (qd, *J* = 11.4, 5.4 Hz, 2H), 3.07 (d, *J* = 13.8 Hz, 1H), 2.73 (dd, *J* = 13.8, 9.6 Hz, 1H); ^13^C{^1^H} NMR (101 MHz, CDCl_3_) δ 162.2 (d, *J* = 245.0 Hz, 1C), 131.9, 130.0 (d, *J* = 8.0 Hz, 1C), 129.9 (2C), 129.1 (2C), 121.8, 117.4, 115.61 (d, *J* = 21.4 Hz, 1C), 80.9, 72.9, 64.0, 57.4, 55.7, 29.9; ^19^F NMR (376 MHz, CDCl_3_) δ −115.2; IR (ATR) 3408, 3340, 2925, 2908, 2873, 2848, 1601, 1508, 1496, 1365, 1329, 1292, 1277, 1155, 1022, 933, 899, 862, 756 cm^–1^; HRMS (APCI) *m*/*z* [M + H]^+^ calcd for C_17_H_19_FNO_2_ 288.1394, found 288.1390; [α]^25^_D_ = −4.8° (*c* = 0.42, CHCl_3_); enantiomeric excess (ee) 94%; retention times *t*_major_ = 7.9 min and *t*_minor_ = 6.5 min determined by HPLC (Chiralpak column IA, 80/20 *n*-heptane/isopropanol, flow rate of 1.0 mL/min, 25 °C, λ = 250 nm).

#### (*R*)-[1-(4-Bromobenzyl)-1,2,3,5-tetrahydrobenzo[*e*][1,4]oxazepin-3-yl]methanol (**2h**)

The product was obtained by column chromatography (silica, 1/1 hexane/EtOAc) as a yellow oil in 96% (16.7 mg) yield: ^1^H NMR (400 MHz, CDCl_3_) δ 7.47 (d, *J* = 8.4 Hz, 2H), 7.34–7.17 (m, 4H), 7.05–6.92 (m, 2H), 4.85–4.69 (m, 2H), 4.54 (d, *J* = 14.1 Hz, 1H), 4.18 (d, *J* = 14.1 Hz, 1H), 3.66 (s, 1H), 3.52–3.36 (m, 2H), 3.06 (d, *J* = 13.0 Hz, 1H), 2.75 (dd, *J* = 13.8, 9.6 Hz, 1H); ^13^C{^1^H} NMR (101 MHz, CDCl_3_) δ 137.3, 132.0, 131.9 (3C), 130.3 (2C), 129.9, 129.1, 122.3, 121.5, 117.9, 80.5, 72.8, 63.9, 57.7, 56.0; IR (ATR) 3350, 2924, 2877, 2852, 1666, 1599, 1495, 1361, 1313, 1290, 1267, 1109, 1011, 935, 868, 756 cm^–1^; HRMS (ESI) *m*/*z* [M + H]^+^ calcd for C_17_H_18_BrNNaO_2_ 370.0413, found 370.0411; [α]^25^_D_ = −45.5° (*c* = 1.2, CHCl_3_); enantiomeric excess (ee) 94%; retention times *t*_major_ = 27.8 min and *t*_minor_ = 21.2 min determined by HPLC (Chiralpak IA column, 95/5 *n*-heptane/isopropanol, flow rate of 1.0 mL/min, 25 °C, λ = 250 nm).

#### (*R*)-[1-(3-Bromobenzyl)-1,2,3,5-tetrahydrobenzo[*e*][1,4]oxazepin-3-yl]methanol (**2i**)

The product was obtained by column chromatography (silica, 1/1 hexane/EtOAc) as a yellow oil in 92% (16.0 mg) yield: ^1^H NMR (400 MHz, CDCl_3_) δ 7.54 (s, 1H), 7.41 (d, *J* = 7.9 Hz, 1H), 7.35 (d, *J* = 7.7 Hz, 1H), 7.29–7.18 (m, 3H), 7.06–6.95 (m, 2H), 4.92–4.70 (m, 2H), 4.58 (d, *J* = 14.1 Hz, 1H), 4.20 (d, *J* = 14.1 Hz, 1H), 3.77–3.63 (m, 1H), 3.56–3.35 (m, 2H), 3.10 (d, *J* = 13.0 Hz, 1H), 2.79 (dd, *J* = 13.8, 9.6 Hz, 1H); ^13^C{^1^H} NMR (101 MHz, CDCl_3_) δ 140.6, 132.0, 131.7, 130.9, 130.3, 130.0, 129.1, 127.3 (2C), 122.9, 122.5, 118.0, 80.4, 72.8, 63.9, 57.8, 56.1; IR (ATR) 3363, 2945, 2927, 2879, 2848, 1597, 1570, 1454, 1361, 1298, 1257, 1196, 1036, 995, 960, 847, 750 cm^–1^; HRMS (ESI) *m*/*z* [M + H]^+^ calcd for C_17_H_18_BrNNaO_2_ 370.0413, found 370.0411; [α]^25^_D_ = −47.1° (*c* = 1.3, CHCl_3_); enantiomeric excess (ee) 92%; retention times *t*_major_ = 28.8 min and *t*_minor_ = 23.4 min determined by HPLC (Chiralpak IA column, 95/5 *n*-heptane/isopropanol, flow rate of 1.0 mL/min, 25 °C, λ = 211 nm).

#### (*R*)-[1-(2-Bromobenzyl)-1,2,3,5-tetrahydrobenzo[*e*][1,4]oxazepin-3-yl]methanol (**2j**)

The product was obtained by column chromatography (silica, 1/1 hexane/EtOAc) as a yellow oil in 98% (17.1 mg) yield: ^1^H NMR (400 MHz, CDCl_3_) δ 7.57 (dd, *J* = 8.0, 1.3 Hz, 1H), 7.48 (d, *J* = 7.7 Hz, 1H), 7.33–7.21 (m, 3H), 7.15 (td, *J* = 7.6, 1.8 Hz, 1H), 7.05–6.89 (m, 2H), 4.80 (q, *J* = 13.0 Hz, 2H), 4.63 (d, *J* = 14.8 Hz, 1H), 4.42 (d, *J* = 14.8 Hz, 1H), 3.77–3.64 (m, 1H), 3.59–3.42 (m, 2H), 3.18 (d, *J* = 12.2 Hz, 1H), 2.91 (dd, *J* = 14.0, 9.3 Hz, 1H), 1.99 (brs, 1H); ^13^C{^1^H} NMR (101 MHz, CDCl_3_) δ 137.0, 133.3 (2C), 131.7, 130.7, 130.0, 129.2, 129.0, 127.6, 124.4, 122.1, 118.0, 80.2, 72.8, 63.9, 58.1, 56.4; IR (ATR) 3377, 2949, 2925, 2879, 2850, 1662, 1599, 1568, 1495, 1327, 1263, 1198, 1026, 957, 935, 823, 748 cm^–1^; HRMS (ESI) *m*/*z* [M + H]^+^ calcd for C_17_H_18_BrNNaO_2_ 370.0413, found 370.0410; [α]^25^_D_ = −49.0° (*c* = 1.31, CHCl_3_); enantiomeric excess (ee) 90%; retention times *t*_major_ = 22.6 min and *t*_minor_ = 18.8 min determined by HPLC (Chiralpak IA column, 95/5 *n*-heptane/isopropanol, flow rate of 1.0 mL/min, 25 °C, λ = 211 nm).

#### (*R*)-[1-(2-Nitrobenzyl)-1,2,3,5-tetrahydrobenzo[*e*][1,4]oxazepin-3-yl]methanol (**2k**)

The product was obtained by column chromatography (silica, 1/1 hexane/EtOAc) as a yellow oil in 80% (15.4 mg) yield: ^1^H NMR (400 MHz, CDCl_3_) δ 7.88 (dd, *J* = 8.0, 1.3 Hz, 1H), 7.61–7.52 (m, 2H), 7.43 (ddd, *J* = 8.7, 7.0, 2.0 Hz, 1H), 7.21 (d, *J* = 7.5 Hz, 2H), 6.99–6.92 (m, 2H), 5.01 (d, *J* = 15.3 Hz, 1H), 4.75 (d, *J* = 13.0 Hz, 1H), 4.63 (d, *J* = 13.0 Hz, 1H), 4.50 (d, *J* = 15.3 Hz, 1H), 3.70–3.62 (m, 1H), 3.46 (qd, *J* = 11.4, 5.4 Hz, 2H), 3.04 (dd, *J* = 13.9, 1.9 Hz, 1H), 2.84 (dd, *J* = 13.9, 9.5 Hz, 1H); ^13^C{^1^H} NMR (101 MHz, CDCl_3_) δ 151.1, 133.8, 132.9, 131.9, 130.9, 130.1, 129.0, 128.6, 125.1, 122.0, 117.2, 80.4, 72.8, 64.0, 57.2, 55.4; IR (ATR) 3346, 3315, 3252, 2949, 2883, 2854, 1604, 1583, 1560, 1454, 1360, 1265, 1161, 1011, 974, 943, 825, 756 cm^–1^; HRMS (ESI) *m*/*z* [M + H]^+^ calcd for C_17_H_18_N_2_NaO_4_ 337.1159, found 337.1156; [α]^25^_D_ = −42.9° (*c* = 1.1, MeOH); enantiomeric excess (ee) 88%; retention times *t*_major_ = 27.7 min and *t*_minor_ = 24.3 min determined by HPLC (Chiralpak column IA, 90/10 *n*-heptane/isopropanol, flow rate of 1.0 mL/min, 25 °C, λ = 220 nm).

#### (*R*)-[1-(Naphthalen-2-ylmethyl)-1,2,3,5-tetrahydrobenzo[*e*][1,4]oxazepin-3-yl]methanol (**2l**)

The product was obtained by column chromatography (silica, 1/1 hexane/EtOAc) as a yellow oil in 85% (13.6 mg) yield: ^1^H NMR (400 MHz, CDCl_3_) δ 7.83 (ddd, *J* = 12.4, 6.8, 3.5 Hz, 4H), 7.58 (dd, *J* = 8.5, 1.7 Hz, 1H), 7.53–7.44 (m, 2H), 7.33–7.26 (m, 1H), 7.11 (dd, *J* = 8.0, 1.1 Hz, 1H), 6.98 (td, *J* = 7.3, 1.1 Hz, 1H), 4.87–4.77 (m, 2H), 4.74 (d, *J* = 13.9 Hz, 1H), 4.36 (d, *J* = 13.9 Hz, 1H), 3.64 (dddd, *J* = 9.5, 6.8, 4.0, 1.9 Hz, 1H), 3.46–3.30 (m, 2H), 3.13 (dd, *J* = 13.8, 1.9 Hz, 1H), 2.75 (dd, *J* = 13.9, 9.5 Hz, 1H); ^13^C{^1^H} NMR (101 MHz, CDCl_3_) δ 152.5, 136.4, 133.5, 133.0, 131.9, 129.9, 129.1, 128.6, 127.9, 127.9, 127.3, 126.5, 126.4, 126.0, 121.6, 117.3, 80.9, 72.9, 64.0, 58.4, 55.6; IR (ATR) 3390, 2947, 2925, 2879, 2850, 1599, 1508, 1439, 1354, 1259, 1215, 1159, 953, 941, 854, 746 cm^–1^; HRMS (ESI) *m*/*z* [M + Na]^+^ calcd for C_21_H_21_NNaO_2_ 342.1464, found 342.1462; [α]^25^_D_ = −69.9° (*c* = 0.78, CHCl_3_); enantiomeric excess (ee) 92%; retention times *t*_major_ = 23.0 min and *t*_minor_ = 25.0 min determined by HPLC (Chiralpak column IG, 90/10 *n*-heptane/isopropanol, flow rate of 1.0 mL/min, 25 °C, λ = 225 nm).

#### (*R*)-[1-(Thiophen-2-ylmethyl)-1,2,3,5-tetrahydrobenzo[*e*][1,4]oxazepin-3-yl]methanol (**2m**)

The product was obtained by column chromatography (silica, 1/1 hexane/EtOAc) as a yellow oil in 78% (10.7 mg) yield: ^1^H NMR (400 MHz, CDCl_3_) δ 7.32–7.26 (m, 1H), 7.26–7.21 (m, 2H), 7.08 (d, *J* = 8.0 Hz, 1H), 7.05–7.03 (m, 1H), 7.00 (td, *J* = 7.4, 1.2 Hz, 1H), 6.96 (dd, *J* = 5.1, 3.4 Hz, 1H), 4.82–4.72 (m, 2H), 4.69 (dd, *J* = 14.2, 1.1 Hz, 1H), 4.48 (d, *J* = 14.1 Hz, 1H), 3.83–3.75 (m, 1H), 3.55–3.40 (m, 2H), 3.20 (dd, *J* = 13.7, 1.9 Hz, 1H), 2.72 (dd, *J* = 13.8, 9.5 Hz, 1H); ^13^C{^1^H} NMR (101 MHz, CDCl_3_) δ 132.2, 129.9, 129.1 (2C), 126.7 (2C), 125.9, 125.5, 122.0, 117.2, 81.1, 72.9, 64.1, 55.5, 53.3; IR (ATR) 3406, 3103, 2945, 2925, 2879, 2846, 1599, 1581, 1435, 1352, 1263, 1236, 1109, 955, 935, 829, 758 cm^–1^; HRMS (ESI) *m*/*z* [M + Na]^+^ calcd for C_15_H_17_NNaO_2_S 298.0872, found 298.0866; [α]^25^_D_ = −60.1° (*c* = 0.69, CHCl_3_); enantiomeric excess (ee) 90%; retention times *t*_major_ = 19.2 min and *t*_minor_ = 16.7 min determined by HPLC (Chiralpak column IG, 90/10 *n*-heptane/isopropanol, flow rate of 1.0 mL/min, 25 °C, λ = 209 nm).

#### (*R*)-(1-Allyl-1,2,3,5-tetrahydrobenzo[*e*][1,4]oxazepin-3-yl)methanol (**2n**)

The product was obtained by column chromatography (silica, 1/1 hexane/EtOAc) as a yellow oil in 52% (5.7 mg) yield: ^1^H NMR (400 MHz, CDCl_3_) δ 7.35–7.15 (m, 3H), 7.02–6.96 (m, 1H), 5.98–5.92 (m, 1H), 5.37–5.21 (m, 2H), 4.79 (d, *J* = 13.1 Hz, 1H), 4.68 (d, *J* = 13.1 Hz, 1H), 4.01 (dd, *J* = 14.4, 5.1 Hz, 1H), 3.88–7.78 (m, 2H), 3.71–3.49 (m, 2H), 3.31 (d, *J* = 13.6 Hz, 1H), 2.77 (brs, 1H); ^13^C{^1^H} NMR (101 MHz, CDCl_3_) δ 134.3, 131.6, 129.9 (2C), 128.9 (2C), 126.8, 118.7, 80.2, 72.6, 63.9, 57.2, 55.4; IR (ATR) 3400, 2947, 2925, 2879, 2850, 1599, 1579, 1454, 1356, 1323, 1259, 1161, 1036, 989, 922, 829, 756 cm^–1^; HRMS (ESI) *m*/*z* [M + H]^+^ calcd for C_13_H_18_NO_2_ 220.1332, found 220.1335; enantiomeric excess (ee) not determined because of rapid decomposition during HPLC.

#### (*R*)-(1-Methyl-1,2,3,5-tetrahydrobenzo[*e*][1,4]oxazepin-3-yl)methanol (**2o**)

The product was obtained by column chromatography (silica, 1/1 hexane/EtOAc) as a yellow oil in 30% (2.9 mg) yield: ^1^H NMR (600 MHz, CDCl_3_) δ 7.28 (td, *J* = 7.8, 1.7 Hz, 1H), 7.19 (dd, *J* = 7.5, 1.7 Hz, 1H), 6.96 (m, 2H), 4.77 (d, *J* = 13.0 Hz, 1H), 4.64 (d, *J* = 12.9 Hz, 1H), 3.88 (s, 1H), 3.64 (dd, *J* = 11.4, 4.0 Hz, 1H), 3.56 (dd, *J* = 11.4, 6.9 Hz, 1H), 3.12 (d, *J* = 13.9 Hz, 1H), 2.97 (s, 3H), 2.87–2.74 (m, 1H); ^13^C{^1^H} NMR (151 MHz, CDCl_3_) δ 131.3, 129.9, 129.0, 121.4, 116.2, 80.4, 73.1, 64.1, 59.6, 43.3; IR (ATR) 3373, 3107, 2949, 2879, 2852, 2787, 1599, 1579, 1439, 1379, 1294, 1257, 1182, 1147, 1009, 970, 951, 860, 748 cm^–1^; HRMS (ESI) *m*/*z* [M + Na]^+^ calcd for C_11_H_15_NNaO_2_ 216.0995, found 216.0995; [α]^25^_D_ = −16.9° (*c* = 0.4, CHCl_3_); enantiomeric excess (ee) 65%; retention times *t*_minor_ = 8.4 min and t_major_ = 11.3 min determined by HPLC (Chiralpak column IA, 80/20 *n*-heptane/isopropanol, flow rate of 1.0 mL/min, 25 °C, λ = 215 nm).

#### (*R*)-(1-Phenyl-1,2,3,5-tetrahydrobenzo[*e*][1,4]oxazepin-3-yl)methanol (**2q**)

The product was obtained by column chromatography (silica, 1/1 hexane/EtOAc) as a yellow oil in 75% (9.6 mg) yield: ^1^H NMR (400 MHz, CDCl_3_) δ 7.38–7.34 (m, 1H), 7.33–7.28 (m, 1H), 7.24–7.16 (m, 4H), 6.80 (td, *J* = 7.4, 1.0 Hz, 1H), 6.78–6.73 (m, 2H), 4.75–4.51 (m, 2H), 4.21 (dd, *J* = 14.9, 1.4 Hz, 1H), 3.97–3.85 (m, 1H), 3.75–3.57 (m, 2H), 3.18 (dd, *J* = 14.9, 9.8 Hz, 1H); ^13^C{^1^H} NMR (101 MHz, CDCl_3_) δ 147.79, 147.35, 136.68, 130.38, 129.51, 129.32, 127.55, 125.71, 118.91, 115.51, 78.88, 72.49, 64.13, 52.55; HRMS (ESI) *m*/*z* [M + H]^+^ calcd for C_16_H_17_NNaO_2_ 278.1151, found 278.1153; IR (ATR) 3392, 2925, 2871, 2852, 1593, 1576, 1360, 1300, 1265, 1230, 1194, 1034, 989, 949, 823, 746 cm^–1^; [α]^25^_D_ = −9.2° (*c* = 0.44, CHCl_3_); enantiomeric excess (ee) 90%; retention times *t*_minor_ = 6.1 min and *t*_major_ = 6.6 min determined by HPLC (Chiralpak column IA, 80/20 *n*-heptane/isopropanol, flow rate of 1.0 mL/min, 25 °C, λ = 210 nm).

#### (*R*)-[1-(4-Methoxyphenyl)-1,2,3,5-tetrahydrobenzo[*e*][1,4]oxazepin-3-yl]methanol (**2r**)

The product was obtained by column chromatography (silica, 1/1 hexane/EtOAc) as a yellow oil in 60% (8.6 mg) yield: ^1^H NMR (400 MHz, CDCl_3_) δ 7.28 (dd, *J* = 7.4, 1.7 Hz, 1H), 7.23–7.16 (m, 2H), 7.05 (td, *J* = 7.4, 1.3 Hz, 1H), 6.91 (dd, *J* = 8.0, 1.2 Hz, 1H), 6.84 (d, *J* = 3.7 Hz, 3H), 4.78 (d, *J* = 13.1 Hz, 1H), 4.65 (d, *J* = 13.1 Hz, 1H), 4.03 (dd, *J* = 14.4, 1.7 Hz, 1H), 3.85 (dddd, *J* = 9.5, 6.9, 4.0, 1.7 Hz, 1H), 3.78 (s, 3H), 3.68 (dd, *J* = 11.3, 3.9 Hz, 1H), 3.65–3.59 (m, 1H), 3.19 (ddd, *J* = 14.9, 9.7, 6.7 Hz, 1H), 2.01 (s, 1H); ^13^C{^1^H} NMR (101 MHz, CDCl_3_) δ 154.4, 149.5, 142.6, 134.5, 130.1, 129.1, 124.9, 123.8, 120.8 (2C), 114.9 (2C), 79.6, 72.8, 64.1, 55.8, 54.7; IR (ATR) 3400, 2929, 2871, 2854, 1593, 1576, 1404, 1360, 1232, 1194, 1034, 989, 947, 823, 746, cm^–1^; HRMS (ESI) *m*/*z* [M + H]^+^ calcd for C_17_H_19_NNaO_3_ 308.1257, found 308.1255; [α]^25^_D_ = −31.0° (*c* = 0.29, CHCl_3_); enantiomeric excess (ee) 94%; retention times *t*_major_ = 14.1 min and *t*_minor_ = 13.2 min determined by HPLC (Chiralpak column IB, 90/10 *n*-heptane/isopropanol, flow rate of 1.0 mL/min, 25 °C, λ = 248 nm).

#### (*R*)-[1-(4-Bromophenyl)-1,2,3,5-tetrahydrobenzo[*e*][1,4]oxazepin-3-yl]methanol (**2u**)

The product was obtained by column chromatography (silica, 1/1 hexane/EtOAc) as a yellow oil in 60% (8.6 mg) yield: ^1^H NMR (400 MHz, CDCl_3_) δ 7.39–7.26 (m, 4H), 7.25–7.16 (m, 2H), 6.63–6.56 (m, 2H), 4.69 (d, *J* = 13.1 Hz, 1H), 4.53 (d, *J* = 13.1 Hz, 1H), 4.18–4.11 (m, 1H), 3.88 (ddd, *J* = 10.6, 6.1, 2.7 Hz, 1H), 3.75–3.55 (m, 2H), 3.18 (dd, *J* = 15.1, 9.9 Hz, 1H), 1.81 (s, 1H); ^13^C{^1^H} NMR (101 MHz, CDCl_3_) δ 146.8, 146.6, 136.9, 132.3, 130.5, 129.5, 127.7, 126.3, 116.6, 110.7, 78.6, 72.4, 64.1, 52.5; IR (ATR) 3368, 2927, 2873, 1572, 1489, 1030, 752, 731 cm^–1^; HRMS (ESI) *m*/*z* [M + Na]^+^ calcd for C_16_H_16_BrNNaO_2_ 356.0257, found 356.0255; enantiomeric excess (ee) not determined.

#### (*R*)-(1-Benzyl-9-methyl-1,2,3,5-tetrahydrobenzo[*e*][1,4]oxazepin-3-yl)methanol (**2v**)

The product was obtained by column chromatography (silica, 1/1 hexane/EtOAc) as a yellow oil in 57% (8.1 mg) yield: ^1^H NMR (400 MHz, CDCl_3_) δ 7.41–7.36 (m, 3H), 7.36–7.32 (m, 2H), 7.32–7.27 (m, 1H), 7.19 (dd, *J* = 6.7, 2.4 Hz, 1H), 7.08–7.01 (m, 2H), 4.80–4.66 (m, 2H), 4.46 (d, *J* = 14.1 Hz, 1H), 4.19 (d, *J* = 14.1 Hz, 1H), 3.89–3.79 (m, 1H), 3.41 (qd, *J* = 11.5, 5.4 Hz, 2H), 3.23 (d, *J* = 14.9 Hz, 1H), 2.89 (dd, *J* = 14.8, 10.5 Hz, 1H), 2.43 (s, 3H); ^13^C{^1^H} NMR (101 MHz, CDCl_3_) δ 135.9, 131.6, 129.2 (2C), 128.8 (2C), 127.8, 127.6, 125.4, 72.8, 64.0, 56.9, 53.0, 19.4; IR 3402, 2922, 2873, 2854, 1597, 1495, 1361, 1311, 1263, 1221, 1165, 1043, 987, 945, 750 cm^–1^; HRMS (ESI) *m*/*z* [M + H]^+^ calcd for C_18_H_22_NO_2_ 284.1645, found 284.1642; [α]^25^_D_ = −22.5° (*c* = 0.45, CHCl_3_); enantiomeric excess (ee) 88%; retention times *t*_major_ = 17.2 min and *t*_minor_ = 15.4 min determined by HPLC (Chiralpak column IG, 95/5 *n*-heptane/isopropanol, flow rate of 1.0 mL/min, 25 °C, λ = 210 nm).

#### (*R*)-(1-Benzyl-8-chloro-1,2,3,5-tetrahydrobenzo[*e*][1,4]oxazepin-3-yl)methanol (**2w**)

The product was obtained by column chromatography (silica, 1/1 hexane/EtOAc) as a yellow oil in 45% (6.8 mg) yield: ^1^H NMR (400 MHz, CDCl_3_) δ 7.39–7.35 (m, 3H), 7.35–7.27 (m, 2H), 7.15 (d, *J* = 7.9 Hz, 1H), 7.02 (d, *J* = 2.0 Hz, 1H), 6.93 (dd, *J* = 7.9, 2.0 Hz, 1H), 4.78 (d, *J* = 13.0 Hz, 1H), 4.66 (d, *J* = 12.9 Hz, 1H), 4.54 (d, *J* = 13.8 Hz, 1H), 4.18 (d, *J* = 13.8 Hz, 1H), 3.61 (ddd, *J* = 7.2, 3.5, 1.5 Hz, 1H), 3.49–3.32 (m, 2H), 3.09 (dd, *J* = 13.9, 1.9 Hz, 1H), 2.71 (dd, *J* = 13.9, 9.4 Hz, 1H); ^13^C{^1^H} NMR (101 MHz, CDCl_3_) δ 153.4, 138.1, 134.5, 130.8, 130.1, 128.8 (2C), 128.5 (2C), 127.7, 121.3, 117.8, 80.8, 72.2, 63.9, 58.1, 55.5; IR (ATR) 3390, 2947, 2924, 2871, 2846, 1593, 1576, 1414, 1377, 1277, 1265, 1178, 1157, 1003, 966, 953, 860, 781 cm^–1^; HRMS (APCI) *m*/*z* [M + H]^+^ calcd for C_17_H_19_ClNO_2_ 304.1099, found 304.1089; [α]^25^_D_ = −34.2° (*c* = 1.2, CHCl_3_); enantiomeric excess (ee) 90%; retention times *t*_minor_ = 9.96 min and *t*_major_ = 13.20 min determined by HPLC (Chiralpak column IA, 90/10 *n*-heptane/isopropanol, flow rate of 1.0 mL/min, 25 °C, λ = 218 nm).

#### (*R*)-(1-Benzyl-8-fluoro-1,2,3,5-tetrahydrobenzo[*e*][1,4]oxazepin-3-yl)methanol (**2x**)

The product was obtained by column chromatography (silica, 1/1 hexane/EtOAc) as a yellow oil in 62% (8.9 mg) yield: ^1^H NMR (400 MHz, CDCl_3_) δ 7.40–7.32 (m, 4H), 7.32–7.27 (m, 1H), 7.17 (dd, *J* = 8.3, 6.6 Hz, 1H), 6.75 (dd, *J* = 11.3, 2.5 Hz, 1H), 6.64 (td, *J* = 8.2, 2.5 Hz, 1H), 4.79 (d, *J* = 13.0 Hz, 1H), 4.71–4.63 (m, 1H), 4.54 (d, *J* = 13.9 Hz, 1H), 4.19 (d, *J* = 13.9 Hz, 1H), 3.66 (dddd, *J* = 9.3, 6.2, 4.0, 1.8 Hz, 1H), 3.51–3.37 (m, 2H), 3.12 (dd, *J* = 13.9, 1.9 Hz, 1H), 2.77 (dd, *J* = 13.9, 9.4 Hz, 1H); ^13^C{^1^H} NMR (101 MHz, CDCl_3_) δ 164.5, 162.0, 138.0, 130.93 (d, *J* = 10.0 Hz) 128.8, 128.5, 127.7, 107.8 (d, *J* = 21.3 Hz), 105.1 (d, *J* = 23.9 Hz), 80.7, 72.2, 63.9, 58.2, 55.8; ^19^F NMR (376 MHz, CDCl_3_) δ −112.3 (q, *J* = 8.3 Hz); IR (ATR) 3379, 2947, 2922, 2873, 2848, 1610, 1589, 1520, 1377, 1358, 1171, 1107, 1028, 991, 968, 931, 829, 737 cm^–1^; HRMS (ESI) *m*/*z* [M + Na]^+^ calcd for C_17_H_18_FNNaO_2_ 310.1214, found 310.1211; [α]^25^_D_ = −16.5° (*c* = 0.4, CHCl_3_); enantiomeric excess (ee) 91%; retention times: *t*_minor_ = 19.9 min and *t*_major_ = 16.1 min determined by HPLC (Chiralpak column IG, 90/10 *n*-heptane/isopropanol, flow rate of 1.0 mL/min, 25 °C, λ = 208 nm).

#### (*R*)-(1-Benzyl-8-methyl-1,2,3,5-tetrahydrobenzo[*e*][1,4]oxazepin-3-yl)methanol (**2y**)

The product was obtained by column chromatography (silica, 1/1 hexane/EtOAc) as a yellow oil in 33% (4.7 mg) yield: ^1^H NMR (600 MHz, CDCl_3_) δ 7.43 (d, *J* = 7.1 Hz, 2H), 7.38–7.35 (m, 2H), 7.33–7.29 (m, 1H), 7.15 (d, *J* = 7.5 Hz, 1H), 6.97 (s, 1H), 6.84 (d, *J* = 7.5 Hz, 1H), 4.84–4.73 (m, 2H), 4.62 (d, *J* = 13.5 Hz, 1H), 4.26 (d, *J* = 13.6 Hz, 1H), 3.69 (d, *J* = 46.1 Hz, 1H), 3.45 (dq, *J* = 22.1, 7.5, 6.9 Hz, 2H), 3.17 (s, 1H), 2.81 (s, 1H), 2.34 (s, 3H); ^13^C{^1^H} NMR (151 MHz, CDCl_3_) δ 139.2, 129.8, 128.9, 128.8, 128.8, 128.6, 127.8, 72.4, 63.9, 63.9, 58.4, 55.7, 21.6; IR (ATR) 3339, 3026, 2873, 1608, 1495, 1109, 1028, 737, 696 cm^–1^; HRMS (ESI) *m*/*z* [M + Na]^+^ calcd for C_18_H_21_NNaO_2_ 306.1464, found 306.1465; [α]^25^_D_ = −15.4° (*c* = 0.35, CHCl_3_); enantiomeric excess (ee) 92%; retention times *t*_minor_ = 8.1 min and *t*_major_ = 14.6 min determined by HPLC (Chiralpak column IA, 95/5 *n*-heptane/isopropanol, flow rate of 1.0 mL/min, 25 °C, λ = 225 nm).

### Procedures for Further Transformation and Applications (**3a–6a**)

#### Synthesis of (*R*)-(1,2,3,5-Tetrahydrobenzo[*e*][1,4]oxazepin-3-yl)methanol (**3a**)

A 5 mL vial equipped with a magnetic stirrer was charged with compound **2a** (0.186 mmol, 50 mg, 1 equiv), methanol (2 mL), and Pd/C (10 wt %, 0.0186 mmol, 2 mg, 0.1 equiv) at room temperature. The resulting solution was stirred for 24 h under a hydrogen atmosphere and monitored by TLC. After the starting material had been fully consumed, the mixture was filtered through Celite and evaporated under reduced pressure, and the residue was purified by silica gel column chromatography (1/1 hexane/EtOAc) to afford the desired product as a yellow oil in 90% (30.6 mg) yield: ^1^H NMR (400 MHz, CDCl_3_) δ 7.19–7.10 (m, 2H), 6.87 (td, *J* = 7.4, 1.1 Hz, 1H), 6.80 (dd, *J* = 8.2, 0.9 Hz, 1H), 4.79 (d, *J* = 13.4 Hz, 1H), 4.61 (d, *J* = 13.4 Hz, 1H), 3.96–3.79 (m, 1H), 3.75 (dddd, *J* = 9.0, 7.2, 3.9, 1.8 Hz, 1H), 3.71–3.56 (m, 3H), 3.30 (dd, *J* = 13.3, 1.8 Hz, 1H), 2.86 (dd, *J* = 13.3, 9.1 Hz, 1H); ^13^C{^1^H} NMR (101 MHz, CDCl_3_) δ 149.7, 129.9, 129.6, 128.8, 120.9, 118.8, 82.5, 73.5, 64.2, 51.0; IR (ATR) 3325, 3205, 2968, 2951, 2897, 2879, 2856, 2839, 1606, 1591, 1373, 1284, 1265, 1163, 1003, 984, 939, 829, 762 cm^–1^; HRMS (ESI) *m*/*z* [M + Na]^+^ calcd for C_10_H_13_NNaO_2_ 202.0838, found 202.0836; [α]^25^_D_ = −31.1° (*c* = 0.52, CHCl_3_); enantiomeric excess (ee) 92%; retention times *t*_major_ = 38.3 min and *t*_minor_ = 28.5 min determined by HPLC (Chiralpak column IA, 95/5 *n*-heptane/isopropanol, flow rate of 1.0 mL/min, 25 °C, λ = 242 nm).

#### Synthesis of (*R*)-(1-Benzyl-1,2,3,5-tetrahydrobenzo[*e*][1,4]oxazepin-3-yl)methyl 4-Methylbenzenesulfonate (**4a**)

A 5 mL vial equipped with a magnetic stirrer was charged with compound **2a** (0.186 mmol, 50 mg, 1 equiv) and TsCl (0.223 mmol, 42.6 mg, 1.2 equiv) in anhydrous DCM (2 mL) at 0 °C. To the mixture was then added dropwise Et_3_N (0.372 mmol, 37.6 mg, 2 equiv) under a N_2_ atmosphere. The resulting solution was stirred for 24 h and monitored by TLC. After the starting material had been fully consumed, the mixture was evaporated under reduced pressure, and the residue was purified by silica gel column chromatography (5/1 hexane/EtOAc) to afford the desired product as a yellow oil in 99% (77.8 mg) yield: ^1^H NMR (400 MHz, CDCl_3_) δ 7.72–7.66 (m, 2H), 7.34 (d, *J* = 4.3 Hz, 4H), 7.31–7.27 (m, 3H), 7.23 (td, *J* = 7.8, 1.6 Hz, 1H), 7.17 (dd, *J* = 7.4, 1.5 Hz, 1H), 6.99 (d, *J* = 8.0 Hz, 1H), 6.92 (td, *J* = 7.4, 1.0 Hz, 1H), 4.73–4.51 (m, 3H), 4.21 (d, *J* = 14.1 Hz, 1H), 3.95 (dd, *J* = 10.3, 5.6 Hz, 1H), 3.81 (dd, *J* = 10.3, 5.4 Hz, 1H), 3.73 (dtd, *J* = 9.0, 5.5, 1.9 Hz, 1H), 3.16 (dd, *J* = 13.9, 1.8 Hz, 1H), 2.69 (dd, *J* = 13.9, 9.0 Hz, 1H), 2.44 (s, 3H); ^13^C{^1^H} NMR (101 MHz, CDCl_3_) δ 151.9, 144.9, 138.6, 132.9, 131.3, 129.9 (2C), 129.8, 129.1, 128.8 (2C), 128.4 (2C), 128.1 (2C), 127.5, 121.5, 117.2, 77.5, 72.7, 69.8, 58.1, 55.6, 21.8; HRMS (ESI) *m*/*z* [M + Na]^+^ calcd for C_24_H_25_NNaO_4_S 446.1396, found 446.1404; IR (ATR) 3086, 2954, 2924, 2885, 2846, 1599, 1495, 1360, 1308, 1263, 1221, 1176, 1018, 1005, 984, 931, 814, 758 cm^–1^; [α]^25^_D_ = −56.9° (*c* = 0.33, CHCl_3_); enantiomeric excess (ee) 91%; retention times *t*_minor_ = 13.5 min and *t*_major_ = 16.1 min determined by HPLC (Chiralpak column IA, 80/20 *n*-heptane/isopropanol, flow rate of 1.0 mL/min, 25 °C, λ = 209 nm).

#### Synthesis of (*R*)-3-(Azidomethyl)-1-benzyl-1,2,3,5-tetrahydrobenzo[*e*][1,4]oxazepine (**5a**)

A 5 mL vial equipped with a magnetic stirrer was charged with compound **4a** (0.118 mmol, 50 mg, 1 equiv) and dissolved in DMSO (2 mL) at room temperature. NaN_3_ (0.118 mmol, 7.7 mg, 1 equiv) was added to the mixture, and the resulting solution was stirred for 24 h and monitored by TLC. After the starting material had been fully consumed, the mixture was diluted with Et_2_O (10 mL) and washed with water (3 × 2.5 mL) and brine (2.5 mL). The organic layer was dried over Na_2_SO_4_ and evaporated under reduced pressure, and the residue was purified by silica gel column chromatography (5/1 hexane/EtOAc) to afford the desired product as a yellow oil in 50% (17.4 mg) yield: ^1^H NMR (400 MHz, CDCl_3_) δ 7.42–7.38 (m, 2H), 7.38–7.32 (m, 2H), 7.32–7.27 (m, 1H), 7.24 (s, 1H), 7.11 (d, *J* = 7.8 Hz, 1H), 7.03–6.97 (m, 1H), 4.88–4.71 (m, 2H), 4.61 (d, *J* = 13.7 Hz, 1H), 4.27 (d, *J* = 13.7 Hz, 1H), 3.76 (s, 1H), 3.29–3.14 (m, 2H), 3.04 (dd, *J* = 12.8, 4.5 Hz, 1H), 2.80 (dd, *J* = 13.6, 9.5 Hz, 1H); ^13^C{^1^H} NMR (101 MHz, CDCl_3_) δ 131.7, 129.9, 129.1, 128.8 (2C), 128.6 (2C), 127.7, 122.0, 117.6, 79.2, 72.7, 58.2, 56.4, 52.9; IR (DRIFT) 3062, 2954, 2922, 2850, 2100, 1599, 1581, 1365, 1292, 1259, 1157, 1012, 962, 933, 864, 762 cm^–1^; HRMS (APCI) *m*/*z* [M + H]^+^ calcd for C_17_H_19_N_2_O 267.1492, found 267.1480; [α]^25^_D_ = −25.0° (*c* = 0.88, CHCl_3_); enantiomeric excess (ee) 92%; retention times *t*_major_ = 5.6 min and *t*_minor_ = 6.3 min determined by HPLC (Chiralpak column IA, 80/20 *n*-heptane/isopropanol, flow rate of 1.0 mL/min, 25 °C, λ = 216 nm).

#### Synthesis of (*R*)-1-Benzyl-3-(chloromethyl)-1,2,3,5-tetrahydrobenzo[*e*][1,4]oxazepine (**6a**)

A 5 mL vial equipped with a magnetic stirrer was charged with compound **4a** (0.118 mmol, 50 mg, 1 equiv) and dissolved in DMF (2 mL). Then, the LiCl (0.118 mmol, 5 mg, 1 equiv) was added. The resulting solution was stirred for 24 h and monitored by TLC. After the starting material had been full consumed, the mixture was diluted with Et_2_O (10 mL) and washed with water (3 × 2.5 mL) and brine (2.5 mL). The organic layer was dried over Na_2_SO_4_ and evaporated under reduced pressure, and the residue was purified by silica gel column chromatography (5/1 hexane/EtOAc) to afford the desired product as a yellow oil in 83% (28.2 mg) yield: ^1^H NMR (400 MHz, CDCl_3_) δ 7.42–7.36 (m, 3H), 7.36–7.32 (m, 2H), 7.31–7.26 (m, 1H), 7.26–7.21 (m, 2H), 7.09 (d, *J* = 7.9 Hz, 1H), 6.98 (td, *J* = 7.4, 0.9 Hz, 1H), 4.90–4.71 (m, 2H), 4.62 (d, *J* = 13.8 Hz, 1H), 4.29 (d, *J* = 13.8 Hz, 1H), 3.78 (dt, *J* = 9.4, 5.9 Hz, 1H), 3.50–3.29 (m, 3H), 2.84 (dd, *J* = 13.9, 9.1 Hz, 1H); ^13^C{^1^H} NMR (101 MHz, CDCl_3_) δ 134.6, 131.5, 129.9, 129.9, 129.2, 129.1, 128.8, 128.7, 127.7, 121.9, 117.6, 79.7, 72.7, 58.2, 56.6, 44.6, 29.9; HRMS (ESI) *m*/*z* [M + H]^+^ calcd for C_17_H_19_ClNO 288.1150, found 288.1141; IR (ATR) 3350, 2954, 2924, 2852, 1599, 1583, 1360, 1296, 1248, 1178, 1155, 1026, 935, 874, 816, 756 cm^–1^; [α]^25^_D_ = −16.9° (*c* = 0.42, CHCl_3_); enantiomeric excess (ee) 92%; retention times *t*_major_ = 4.7 min and *t*_minor_ = 5.3 min determined by HPLC (Chiralpak column IA, 95/5 *n*-heptane/isopropanol, flow rate of 1.0 mL/min, 25 °C, λ = 211 nm).

## Data Availability

The data underlying this study are available in the published article and its Supporting Information.

## Supporting Information Available

Experimental procedures and spectral data for all prepared compounds, copies of the ^1^H NMR, ^13^C NMR, and ^19^F NMR spectra and HPLC chromatographs, and crystallographic data for compound **2d** (PDF)
FAIR data, including the primary NMR FID files, for compounds **S1–S15**, **1a–1x**, **2a–2x**, and **3a–6a** (ZIP)
