# Supplementary material for: Enantioenriched 1,4-Benzoxazepines via Chiral Brønsted Acid-Catalyzed Enantioselective Desymmetrization of 3-Substituted Oxetanes
Source: J Org Chem. 2023 Nov 21;88(24):17024–36. doi: 10.1021/acs.joc.3c01929 (PMC10729023; doi:10.1021/acs.joc.3c01929)
Supplement: Supplementary file 1 — jo3c01929_si_001.pdf [file jo3c01929_si_001.pdf]

Enantioenriched 1,4-Benzoxazepines *via* Chiral Brønsted Acid Catalyzed Enantioselective Desymmetrization of 3-substituted Oxetanes

*Martin Nigríni,[a] Viraj A. Bhosale,[a] Ivana Císařová,[b] and Jan Veselý[a,]**

[a] *Department of Organic Chemistry, Faculty of Science, Charles University, Hlavova 2030, 128 43 Prague, Czech Republic.*

[b] *Department of Inorganic Chemistry, Faculty of Science, Charles University, Hlavova 2030, 128 43 Prague, Czech Republic.*

Corresponding author Email: jan.vesely@natur.cuni.cz.

SUPPORTING INFORMATION

General synthesis for substituted (2-nitrophenyl)methanols (2a-b): 2

General synthesis for substituted (2-nitrophenyl)methanols (2c-e): 2

General synthesis for substituted 1-(bromomethyl)-2-nitrobenzenes (4a-e): 3

Procedure for Synthesis of *N*-phenyloxetan-3-amine (S1): 4

General Procedure A for Synthesis of Starting Materials: Synthesis of Substituted Oxetanes (S2-6): 4

General Procedure B for Synthesis of Starting Materials: Synthesis of substituted oxetanes (S7-8): 5

General procedure for reduction of nitro group (S9-14): 5

General procedure for reduction of nitro group (S1) 6

Synthesis of substituted amines for enantiomeric desymmetrization reaction (1a-k): 6

Synthesis of substituted amines for enantiomeric desymmetrization reaction (1q-u): 7

General Procedure for Enantioselective Desymmetrization of Oxetane Derivatives (2a-y): 7

X-Ray Crystallography 8

NMR DATA 19

HPLC DATA 163

General synthesis for substituted (2-nitrophenyl)methanols (2a-b):

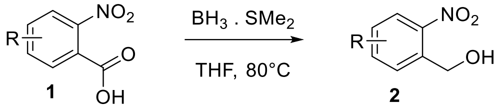

To a 2M solution of the substituted 2-nitrobenzoic acid in dried THF was added drop wise a 10 M solution of borane dimethylsulfide complex (1.1– 1.3 equiv). The resulting solution was heated to 80 °C. After 3 hours, a 3 M aqueous solution of hydrochloric acid was added drop wise into this reaction system until effervescence was no longer observed. The resulting mixture was then extracted with 3 × 30 mL of ethyl acetate. The combined organic phases were washed with a saturated aqueous solution of Na_2CO_3 followed by brine. The resulting organic phase was dried over Na_2SO_4 and filtered. The filtrate was concentrated in vacuo to afford the product. The product was used directly to the next reaction without any purification. All compounds are known from the literature and spectral data are with accordance with reported one. [1]

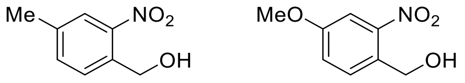

2a Time: 4 h
Yield: 95 %

2b Time: 4 h
Yield: 98 %

Figure 1: Synthesized substituted (2-nitrophenyl)methanols.

General synthesis for substituted (2-nitrophenyl)methanols (2c-e):

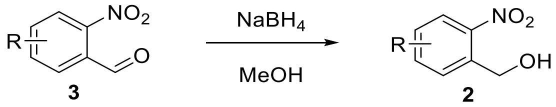

Corresponding (2-nitrophenyl)methanol was dissolved in MeOH (0.1 M) and cooled to 0°C. To that solution, $NaBH_4$ (2 equiv.) was added and stirred at 0°C until full conversion of starting material. After that the reaction was quenched with water and MeOH was evaporated. Then the mixture was extracted with EtOAc (with 1 × 30 mL). dried over Na_2SO_4, filtered, and evaporated on vacuo. Compound was used directly without any purification to the next reaction. The product was used directly to the next reaction without any purification. All compounds are known from the literature and spectral data are with accordance with reported one. [1]

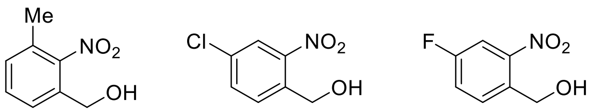

2c Time: 4 h
Yield: 92 %

2d Time: 6 h
Yield: 95 %

2e Time: 6 h
Yield: 97 %

Figure 2: Synthesized substituted (2-nitrophenyl)methanols.

General synthesis for substituted 1-(bromomethyl)-2-nitrobenzenes (4a-e):

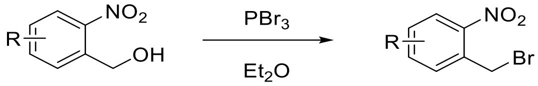

2a-e **4a-e**

To a solution of substituted (2-nitrophenyl)methanol in Et_2O (0.1 M) was added PBr_3 (2 equiv). After 3 hours, a saturated aqueous solution of $NaHCO_3$ was added dropwise to the reaction mixture until a neutral pH was obtained. The resulting mixture was extracted with 1 × 30 mL of ethyl acetate, and the resulting organic phase washed with brine. The resulting organic phase was dried over Na_2SO_4, filtered, and concentrated in vacuo to afford the product. The product was used directly to the next reaction without any purification. All compounds are known from the literature and spectral data are with accordance with reported one. [1]

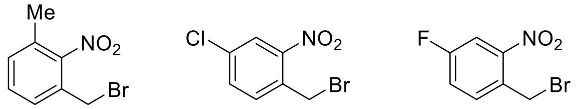

4a Time: 4 h
Yield: 92 %

4b Time: 6 h
Yield: 95 %

4c Time: 6 h
Yield: 97 %

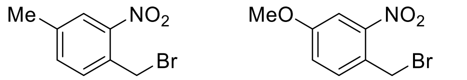

4d Time: 4 h
Yield: 95 %

4e Time: 4 h
Yield: 98 %

Figure 3: Synthesized substituted 1-(bromomethyl)-2-nitrobenzenes.

Literature: [1] Kong, C.; Jana, N.; Driver, T.G. *Org. Lett.* **2013**, *15*, 4, 824–827.

Procedure for Synthesis of *N*-phenyloxetan-3-amine (S1):

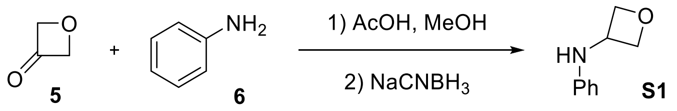

The round-bottomed flask charged with aniline (5.3 mmol, 1 equiv.), oxetan-3-one (13.4 mmol, 2.5 equiv.) and AcOH (10.7 mmol, 2 equiv.) was dissolved in MeOH (0.3 M) at 0°C. The reaction mixture was stirred for 3 hours and then $NaCNBH_3$ (10.7 mmol, 2 equiv.) was added at 0°C and stirred until full conversion of starting material (controlled by TLC). After completion of reaction the crude was quenched with sat. $NaHCO_3$, the MeOH was removed on a rotary evaporator under reduced pressure, extracted with EtOAc (3x), washed with Brine (1x), concentrated on rotary evaporator and purified by silica gel column chromatography using (Hexane:EtOAc, 5:1), gave corresponding 3-substitueted oxetane.

General Procedure A for Synthesis of Starting Materials: Synthesis of Substituted Oxetanes (S2-6):

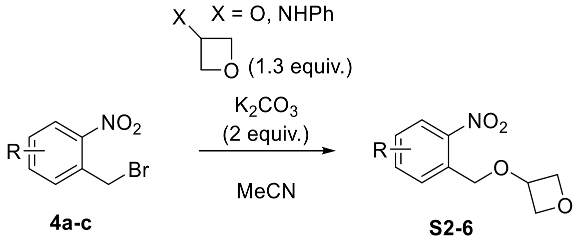

The round-bottomed flask charged with corresponding 1-(bromomethyl)-2-nitrobenzene (1 equiv.), K_2CO_3 (2 equiv.) was dissolved in acetonitrile. After that oxetan-3-ol (1.3 equiv.) was added to the reaction mixture and stirred after full conversion of starting material (controlled by TLC). The reaction mixture was filtered through the Celite, washed with excess of EtOAc. The solvent was removed on a rotary evaporator under reduced pressure, and purified by silica gel column chromatography using (Hexane:EtOAc, 5:1), gave corresponding aniline compounds

General Procedure B for Synthesis of Starting Materials: Synthesis of substituted oxetanes (S7-8):

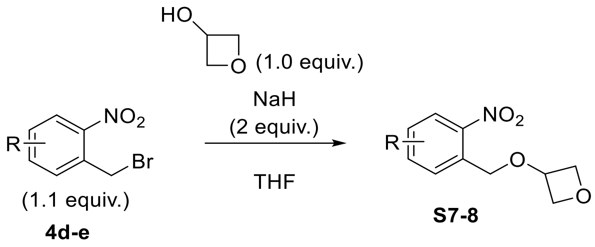

To a suspension of NaH (60% wt,1.2 equiv.) in THF (0.5 M) was added oxetan-3-ol (1.0 equiv.) dropwise at 0°C. The mixture was stirred at room temperature for 30 minutes, and a solution of benzyl bromide (1.2 equiv.) was added dropwise. The reaction mixture was stirred at 60 °C overnight and then allowed to cool to room temperature. Saturated aqueous NH_4Cl solution (10 mL) was added and the organic layer was separated. The aqueous layer was extracted with Et_2O (2 x mL). The combined organic layers were washed with brine, dried over $MgSO_4$, and concentrated under reduced pressure. The residue was purified by flash column chromatography (20-30% EtOAc in hexane) to give the desired product.

General procedure for reduction of nitro group (S9-14):

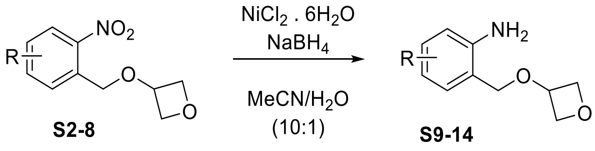

The round-bottomed flask charged with nitrobenzene (1 equiv.), $NiCl_2{\cdot}6H_2O$ (0.2 equiv.) and CH_3CN:H_2O (10:1) was cooled to 0 ºC. After 5 min stirring, $NaBH_4$ (4 equiv.) was added portionwise. A fine black precipitate immediately deposited. Then mixture continued to be stirred for next 15 min at r.t. and later quenched with of aq. NH_4Cl. The reaction mixture filtered through the celite, washed with excess MeOH. The MeOH was removed under high vacuum to afforded crude product. The residue obtained was dissolved in EtOAc, washed with water (3x). The organic layer was collected, dried over Na_2SO_4. The solvent was removed on a rotary evaporator under reduced pressure, and purified by silica gel column chromatography using (DCM: MeOH, 90:10), gave corresponding aniline compounds.

General procedure for reduction of nitro group (S1).

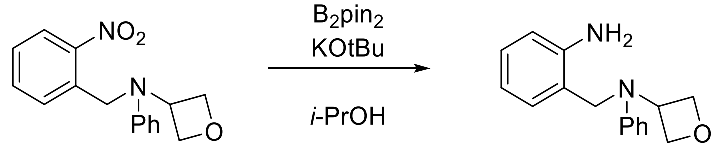

A dried glass reaction tube equipped with a magnetic stir bar was charged with aromatic nitro compounds (0.88 mmol, 1.0 equiv.), B_2pin_2 (2.7 mmol, 3.1 equiv.) and KOtBu (1.06 mmol, 1.2 equiv.), *i*PrOH (5.0 mL) was added and the mixture was then stirred in the preheated oil base at 110 °C for 2 h. The reaction progress was monitored by TLC. After cooling to room temperature, the crude production was diluted with ethyl acetate and then washed with saturated NaCl solution. The organic layers dried over anhydrous Na_2SO_4, concentrated in vacuo, and purified by flash column chromatograph to give the pure products.

Synthesis of substituted amines for enantiomeric desymmetrization reaction (1a-k):

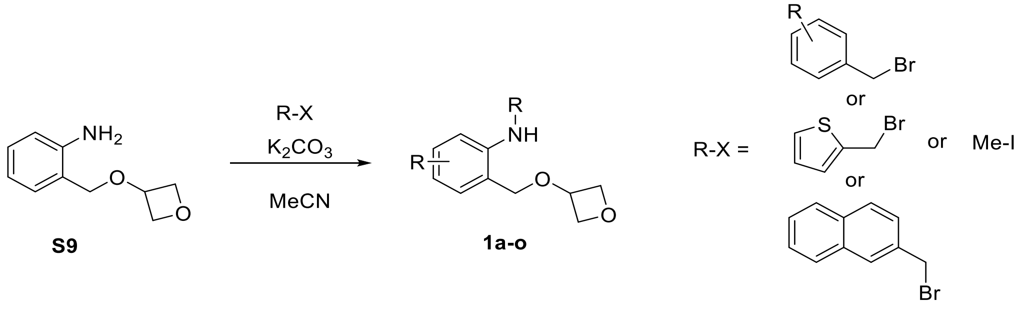

To a 5 mL vial equipped with magnetic stirrer was charge with amine 1 (1equiv.), K_2CO_3 (2 equiv.) and acetonitrile (3 mL) was added at room temperature. To the resulting solution corresponding corresponding substituted bromide or iodine (1.1 equiv.) was added at room temperature. The reaction was stirred for 5h to 24h and monitored by TLC. After full consumption of starting material, the reaction mixture was filtered through Celite, evaporated under reduced pressure, and the residue was purified by silica gel column chromatography (hexane: EtOAc = 5:1) to afford the desired product.

Synthesis of substituted amines for enantiomeric desymmetrization reaction (1q-u):

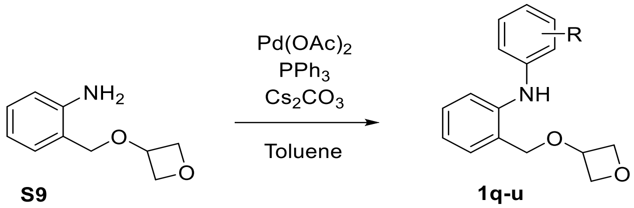

To a 5 mL vial equipped with magnetic stirrer was charge with amine (1 equiv.), $Pd(OAc)_2$ (10 mol %), PPh_3 (20 mol%), Cs_2CO_3 (2 equiv.) and toluene (3 mL) was added at room temperature. The resulting solution was reflux for 24h to 72h and monitored by TLC. After full consumption of starting material, the reaction mixture was filtered through Celite, evaporated under reduced pressure, and the residue was purified by silica gel column chromatography (hexane: EtOAc = 5:1) to afford the desired product.

General Procedure for Enantioselective Desymmetrization of Oxetane Derivatives (2a-y):

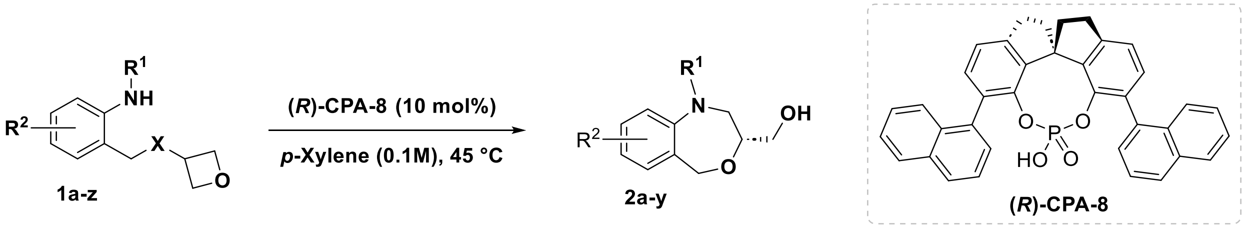

To a 1 mL vial equipped with magnetic stirrer was charge with oxetane derivative 1 (0.05 mmol, 1 equiv.), (*R*)-CPA-6 (0.005 mmol, 10 mol%) and *p*-xylene (0.5 mL, 0.1 M) was added at room temperature. The resulting solution was stirred at 45°C for 24h to 72h and monitored by TLC. After full consumption of starting material, the reaction mixture was evaporated under reduced pressure, and the residue was purified by silica gel column chromatography (hexane: Et_2O = 1:1) to afford the desired product.

X-Ray Crystallography

X-ray single crystal data for **2d** were collected on Bruker D8 VENTURE Kappa Duo PHOTONIII by IμS micro-focus sealed tube CuKα (λ= 1.54178 Å). The structure was solved by direct methods (XT[1]) and refined by full matrix least squares based on F^2 (SHELXL2019[2]). The hydrogen atoms on carbon were fixed into idealized positions (riding model) and assigned temperature factors either $H_{iso}(H) = 1.2\ U_{eq}$(pivot atom) or $H_{iso}(H) = 1.5\ U_{eq}$ (pivot atom) for methyl moiety. The hydrogen atoms in –OH moiety was found on difference Fourier map and refined under rigid-body assumption with assigned temperature factor $H_{iso}(H) = 1.5\ U_{eq}$(O(2a) or O(2b)). The determination of absolute structure was based of anomalous dispersion of nitrogen and oxygen atoms. Absolute structure parameter: -0.10 (6)[3].

Crystal data for **2d,** $C_{17}H_{18}N_2O_4$, $M_r = 314.33$; Monoclinic, $P2_1$ (No 4), $a = 8.3958$ (2) Å, $b = 6.9154$ (2) Å, $c = 26.4082$ (7) Å, $\beta = 91.595$ (1)°, $V = 1532.67$ (7) Å^3, Z = 4, $D_x = 1.362$ Mg m^{-3}, temperature of sample 120(2) K, colorless prism of dimensions 0.44 × 0.21 × 0.15 mm, multi-scan absorption correction (μ = 0.81 mm^{-1}) $T_{min} = 0.79$, $T_{max} = 0.89$; a total of 30775 measured reflections (θ_{max}= 77.4°), from which 6294 were unique ($R_{int} = 0.018$) and 6271 observed according to the $I > 2\sigma(I)$ criterion. The refinement converged (Δ/σ_{max}= 0.001) to $R = 0.032$ for observed reflections and $wR(F^2) = 0.093$, $GOF = 1.09$ for 415 parameters and all 6294 reflections. The final difference map displayed no peaks of chemical significance ($\Delta\rho_{max} = 0.24$, $\Delta\rho_{min}$ -0.17 e.Å^{-3}.

X-ray crystallographic data for **2d** have been deposited with the Cambridge Crystallographic Data Centre under deposition number CCDC 2285225 and can be obtained free of charge from the Centre via its website (https://www.ccdc.cam.ac.uk/structures/).

[1]. SHELXT: Sheldrick, G.M. (2015). *Acta Cryst.* **A71**, 3-8.

[2]. SHELXL: Sheldrick, G.M. (2015). *Acta Cryst.* **C71**, 3-8.

[3]. Parsons,S., Flack, H.D. and Wagner, T. (2013) *Acta Cryst.* **B69**, 249-259.

Fig.1. View on two symmetrically independent molecule A of **2d**, displaying *R* configuration on chiral carbons C(2a). The displacement ellipsoids at 30% probability level. The molecules A and B are differing only very slightly in the orientation of –NO_2 moiety

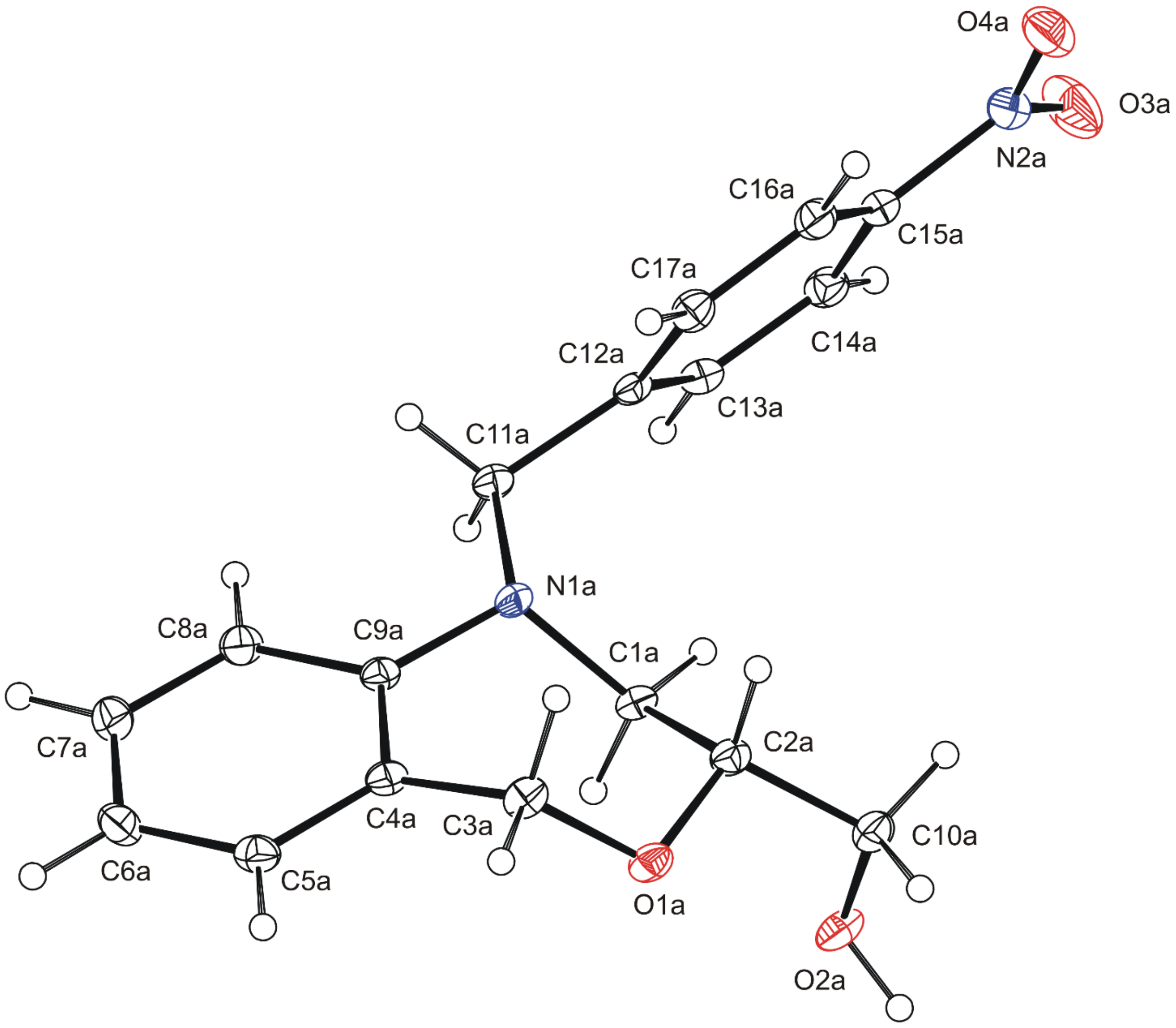

Crystal data, data collection and structure refinement details are summarized in Table 1. Computing details of compound (**2d**) Data collection: Bruker Instrument Service vV6.2.14; cell refinement: SAINT V8.40B (Bruker Nano, Inc., 2019); data reduction: SAINT V8.40B (Bruker Nano, Inc., 2019); program(s) used to solve structure: SHELXT 2018/2 (Sheldrick, 2018); program(s) used to refine structure: SHELXL2018/3 (Sheldrick, 2018).

Table 1. Crystal data

Crystal data

$C_{17}H_{18}N_2O_4$	$F(000) = 664$
$M_r = 314.33$	$D_x = 1.362$ Mg m^{-3}
Monoclinic, $P2_1$	Cu $K\alpha$ radiation, $\lambda = 1.54178$ Å
$a = 8.3958$ (2) Å	Cell parameters from 9813 reflections
$b = 6.9154$ (2) Å	$\theta = 3.4–77.2°$
$c = 26.4082$ (7) Å	$\mu = 0.81$ mm^{-1}
$\beta = 91.595$ (1)°	$T = 120$ K
$V = 1532.67$ (7) Å^3	Prism, colourless
$Z = 4$	0.44 × 0.21 × 0.15 mm

Data collection

Bruker D8 VENTURE Kappa Duo PHOTONIII CMOS diffractometer	6294 independent reflections
Radiation source: IμS micro-focus sealed tube	6271 reflections with $I > 2\sigma(I)$
Helios Cu multilayer optic monochromator	$R_{int} = 0.018$
ϕ and ω scans	$\theta_{max} = 77.4°$, $\theta_{min} = 3.4°$
Absorption correction: multi-scan Krause, L., Herbst-Irmer, R., Sheldrick, G. M., Stalke, D. (2015). "Comparison of silver and molybdenum microfocus X-ray sources for single-crystal structure determination" J. Appl. Cryst. 48, 3-10. doi:10.1107/S1600576714022985	$h = -10 \rightarrow 10$
$T_{min} = 0.79$, $T_{max} = 0.89$	$k = -8 \rightarrow 8$
30775 measured reflections	$l = -33 \rightarrow 33$

Refinement

Refinement on F^2	Secondary atom site location: difference Fourier map
Least-squares matrix: full	Hydrogen site location: mixed

$R[F^2 > 2\sigma(F^2)] = 0.032$	H-atom parameters constrained
$wR(F^2) = 0.093$	$w = 1/[\sigma^2(F_o^2) + (0.0634P)^2 + 0.1617P]$ where $P = (F_o^2 + 2F_c^2)/3$
$S = 1.09$	$(\Delta/\sigma)_{max} < 0.001$
6294 reflections	$\Delta\rangle_{max}$ = 0.24 e Å^{-3}
415 parameters	$\Delta\rangle_{min}$ = -0.17 e Å^{-3}
1 restraint	Absolute structure: Flack x determined using 2786 quotients [(I+)-(I-)]/[(I+)+(I-)] (Parsons, Flack and Wagner, Acta Cryst. B69 (2013) 249-259).
Primary atom site location: structure-invariant direct methods	Absolute structure parameter: -0.10 (6)

Special details

Geometry. All esds (except the esd in the dihedral angle between two l.s. planes) are estimated using the full covariance matrix. The cell esds are taken into account individually in the estimation of esds in distances, angles and torsion angles; correlations between esds in cell parameters are only used when they are defined by crystal symmetry. An approximate (isotropic) treatment of cell esds is used for estimating esds involving l.s. planes.

Fractional atomic coordinates and isotropic or equivalent isotropic displacement parameters (Å^2) for (cu_mn_622p_cu_vesely)

	x	*y*	*z*	$U_{iso}*/U_{eq}$
O1A	0.39031 (13)	0.66609 (18)	0.05674 (4)	0.0238 (2)
N1A	0.12185 (14)	0.80006 (19)	0.12641 (4)	0.0193 (3)
C1A	0.22707 (17)	0.9168 (2)	0.09425 (5)	0.0201 (3)
H1A1	0.237051	1.048088	0.108914	0.024*
H1A2	0.176078	0.929675	0.060159	0.024*
C2A	0.39328 (17)	0.8326 (2)	0.08866 (6)	0.0214 (3)
H2C	0.436779	0.795010	0.122899	0.026*
C3A	0.30968 (18)	0.5042 (2)	0.07878 (6)	0.0232 (3)
H3A1	0.340379	0.497213	0.115200	0.028*
H3A2	0.347427	0.384114	0.062582	0.028*
C4A	0.13084 (17)	0.5105 (2)	0.07384 (5)	0.0208 (3)
C5A	0.0527 (2)	0.3634 (2)	0.04682 (6)	0.0253 (3)
H5A	0.113433	0.274090	0.028000	0.030*
C6A	-0.1125 (2)	0.3450 (3)	0.04691 (6)	0.0298 (4)
H6A	-0.163997	0.243378	0.028619	0.036*
C7A	-0.20057 (19)	0.4764 (3)	0.07398 (6)	0.0293 (4)

H7A	-0.313043	0.462967	0.074980	0.035*
C8A	-0.12562 (18)	0.6285 (3)	0.09979 (6)	0.0243 (3)
H8A	-0.187896	0.719789	0.117436	0.029*
C9A	0.04075 (17)	0.6484 (2)	0.10001 (5)	0.0195 (3)
C10A	0.50418 (18)	0.9810 (3)	0.06561 (6)	0.0256 (3)
H10A	0.535611	1.078542	0.091449	0.031*
H10B	0.601906	0.916105	0.054146	0.031*
C11A	0.02001 (18)	0.9236 (2)	0.15712 (6)	0.0225 (3)
H11A	-0.050165	0.841243	0.177545	0.027*
H11B	-0.048512	1.004120	0.134455	0.027*
C12A	0.11778 (17)	1.0536 (2)	0.19204 (5)	0.0201 (3)
C13A	0.10019 (19)	1.2536 (3)	0.18926 (6)	0.0233 (3)
H13A	0.029320	1.308407	0.164529	0.028*
C14A	0.18508 (19)	1.3732 (2)	0.22221 (6)	0.0255 (3)
H14A	0.174559	1.509742	0.219969	0.031*
C15A	0.28554 (19)	1.2902 (3)	0.25847 (6)	0.0248 (3)
C16A	0.30787 (19)	1.0919 (3)	0.26184 (6)	0.0259 (3)
H16A	0.378658	1.038020	0.286730	0.031*
C17A	0.22455 (19)	0.9749 (2)	0.22807 (6)	0.0248 (3)
H17A	0.239935	0.838826	0.229302	0.030*
O2A	0.42594 (13)	1.0730 (2)	0.02405 (4)	0.0292 (3)
H2A	0.492680	1.105709	0.000314	0.044*
N2A	0.37152 (18)	1.4157 (2)	0.29484 (6)	0.0303 (3)
O4A	0.45162 (17)	1.3386 (2)	0.32873 (5)	0.0410 (3)
O3A	0.3610 (2)	1.5904 (2)	0.28963 (6)	0.0538 (4)
O1B	0.60718 (13)	-0.27077 (17)	0.44229 (4)	0.0240 (3)
N1B	0.87749 (14)	-0.1340 (2)	0.37334 (4)	0.0199 (3)
C1B	0.77154 (17)	-0.0184 (2)	0.40556 (5)	0.0204 (3)
H1B1	0.761733	0.113159	0.391037	0.024*
H1B2	0.822551	-0.005897	0.439658	0.024*
C2B	0.60504 (17)	-0.1014 (2)	0.41128 (5)	0.0221 (3)
H2D	0.560128	-0.135599	0.376970	0.027*
C3B	0.68672 (19)	-0.4313 (2)	0.41957 (6)	0.0251 (3)
H3B1	0.656813	-0.434920	0.383050	0.030*
H3B2	0.647763	-0.552248	0.434990	0.030*
C4B	0.86538 (18)	-0.4268 (2)	0.42498 (5)	0.0218 (3)
C5B	0.9422 (2)	-0.5742 (2)	0.45206 (6)	0.0269 (3)

H5B	0.880498	-0.663002	0.470713	0.032*
C6B	1.1073 (2)	-0.5945 (3)	0.45246 (6)	0.0308 (4)
H6B	1.157776	-0.696446	0.470910	0.037*
C7B	1.1968 (2)	-0.4636 (3)	0.42555 (6)	0.0307 (4)
H7B	1.309145	-0.478412	0.424564	0.037*
C8B	1.12303 (18)	-0.3104 (3)	0.39989 (6)	0.0256 (3)
H8B	1.186293	-0.219678	0.382395	0.031*
C9B	0.95680 (17)	-0.2877 (2)	0.39947 (5)	0.0202 (3)
C10B	0.49579 (18)	0.0450 (3)	0.43559 (6)	0.0254 (3)
H10C	0.463376	0.144742	0.410452	0.031*
H10D	0.398541	-0.020510	0.447205	0.031*
C11B	0.98285 (18)	-0.0100 (2)	0.34400 (6)	0.0231 (3)
H11C	1.053112	-0.092309	0.323598	0.028*
H11D	1.051247	0.066737	0.367610	0.028*
C12B	0.89063 (18)	0.1260 (3)	0.30911 (6)	0.0217 (3)
C13B	0.91240 (18)	0.3243 (2)	0.31293 (6)	0.0235 (3)
H13B	0.982732	0.374553	0.338481	0.028*
C14B	0.83292 (19)	0.4512 (2)	0.27993 (6)	0.0252 (3)
H14B	0.846406	0.587174	0.282823	0.030*
C15B	0.73330 (18)	0.3713 (3)	0.24269 (6)	0.0244 (3)
C16B	0.7082 (2)	0.1748 (3)	0.23797 (6)	0.0270 (3)
H16B	0.638687	0.124766	0.212149	0.032*
C17B	0.78654 (19)	0.0525 (2)	0.27173 (6)	0.0258 (3)
H17B	0.769382	-0.083007	0.269470	0.031*
O2B	0.57679 (13)	0.13322 (19)	0.47729 (4)	0.0296 (3)
H2B	0.514397	0.173137	0.500656	0.044*
N2B	0.64975 (17)	0.5032 (3)	0.20687 (5)	0.0299 (3)
O3B	0.67758 (18)	0.6767 (2)	0.20964 (5)	0.0392 (3)
O4B	0.55816 (17)	0.4303 (3)	0.17533 (6)	0.0459 (4)

Atomic displacement parameters (Å^2) for (cu_mn_622p_cu_vesely)

	U^{11}	U^{22}	U^{33}	U^{12}	U^{13}	U^{23}
O1A	0.0233 (5)	0.0240 (6)	0.0245 (5)	0.0031 (4)	0.0087 (4)	-0.0016 (4)
N1A	0.0177 (5)	0.0222 (6)	0.0182 (6)	0.0001 (5)	0.0052 (4)	-0.0011 (5)
C1A	0.0218 (7)	0.0209 (7)	0.0178 (6)	0.0019 (5)	0.0057 (5)	0.0015 (5)
C2A	0.0200 (7)	0.0243 (8)	0.0200 (6)	0.0018 (6)	0.0031 (5)	0.0002 (6)
C3A	0.0233 (7)	0.0202 (7)	0.0265 (7)	0.0047 (6)	0.0053 (5)	0.0021 (6)

C4A	0.0231 (7)	0.0227 (7)	0.0165 (6)	0.0015 (6)	0.0021 (5)	0.0031 (6)
C5A	0.0331 (8)	0.0234 (7)	0.0194 (7)	-0.0002 (6)	0.0022 (6)	0.0010 (6)
C6A	0.0348 (8)	0.0318 (9)	0.0225 (7)	-0.0068 (7)	-0.0047 (6)	-0.0024 (7)
C7A	0.0243 (7)	0.0374 (9)	0.0260 (7)	-0.0048 (7)	-0.0029 (6)	0.0015 (7)
C8A	0.0209 (7)	0.0305 (9)	0.0215 (7)	0.0029 (6)	0.0004 (5)	0.0007 (6)
C9A	0.0219 (7)	0.0215 (7)	0.0152 (6)	0.0003 (6)	0.0011 (5)	0.0026 (5)
C10A	0.0211 (7)	0.0306 (8)	0.0254 (7)	-0.0005 (6)	0.0048 (6)	0.0001 (6)
C11A	0.0210 (7)	0.0258 (8)	0.0211 (7)	0.0005 (6)	0.0065 (5)	-0.0043 (6)
C12A	0.0191 (6)	0.0238 (8)	0.0176 (6)	-0.0004 (6)	0.0073 (5)	-0.0011 (6)
C13A	0.0251 (7)	0.0267 (8)	0.0183 (7)	0.0010 (6)	0.0051 (5)	0.0018 (6)
C14A	0.0312 (8)	0.0213 (8)	0.0243 (7)	-0.0016 (6)	0.0060 (6)	0.0013 (6)
C15A	0.0245 (7)	0.0304 (9)	0.0197 (7)	-0.0058 (6)	0.0056 (6)	-0.0018 (6)
C16A	0.0235 (7)	0.0310 (8)	0.0232 (7)	-0.0014 (6)	0.0020 (6)	0.0033 (6)
C17A	0.0256 (7)	0.0225 (8)	0.0264 (7)	0.0006 (6)	0.0038 (6)	0.0026 (6)
O2A	0.0263 (5)	0.0357 (7)	0.0262 (6)	0.0005 (5)	0.0101 (4)	0.0085 (5)
N2A	0.0347 (8)	0.0324 (8)	0.0238 (6)	-0.0084 (6)	0.0020 (5)	-0.0016 (6)
O4A	0.0454 (7)	0.0464 (8)	0.0305 (6)	-0.0069 (7)	-0.0092 (5)	-0.0031 (6)
O3A	0.0829 (12)	0.0316 (8)	0.0460 (9)	-0.0167 (8)	-0.0175 (8)	-0.0019 (7)
O1B	0.0258 (5)	0.0230 (6)	0.0237 (5)	-0.0031 (4)	0.0097 (4)	0.0002 (4)
N1B	0.0195 (6)	0.0228 (6)	0.0178 (5)	-0.0009 (5)	0.0063 (4)	0.0008 (5)
C1B	0.0230 (7)	0.0210 (7)	0.0176 (6)	-0.0013 (6)	0.0059 (5)	-0.0006 (5)
C2B	0.0215 (7)	0.0269 (8)	0.0180 (6)	-0.0025 (6)	0.0030 (5)	0.0006 (6)
C3B	0.0263 (7)	0.0223 (8)	0.0270 (7)	-0.0061 (6)	0.0072 (6)	-0.0031 (6)
C4B	0.0262 (7)	0.0226 (8)	0.0168 (6)	-0.0016 (6)	0.0029 (5)	-0.0040 (6)
C5B	0.0383 (9)	0.0236 (8)	0.0188 (7)	-0.0010 (7)	0.0021 (6)	-0.0018 (6)
C6B	0.0388 (9)	0.0293 (9)	0.0239 (7)	0.0067 (7)	-0.0064 (6)	0.0010 (6)
C7B	0.0270 (8)	0.0361 (10)	0.0288 (8)	0.0027 (7)	-0.0050 (6)	-0.0021 (7)
C8B	0.0225 (7)	0.0304 (9)	0.0238 (7)	-0.0026 (6)	-0.0009 (6)	-0.0015 (6)
C9B	0.0238 (7)	0.0216 (7)	0.0153 (6)	-0.0021 (6)	0.0010 (5)	-0.0029 (5)
C10B	0.0220 (7)	0.0309 (8)	0.0237 (7)	0.0023 (6)	0.0042 (5)	0.0008 (6)
C11B	0.0219 (7)	0.0266 (8)	0.0212 (7)	-0.0004 (6)	0.0065 (5)	0.0045 (6)
C12B	0.0208 (6)	0.0273 (8)	0.0176 (7)	0.0001 (6)	0.0086 (5)	0.0018 (6)
C13B	0.0253 (7)	0.0273 (8)	0.0181 (6)	-0.0004 (6)	0.0045 (5)	-0.0003 (6)
C14B	0.0297 (7)	0.0221 (7)	0.0241 (7)	0.0009 (6)	0.0051 (6)	-0.0006 (6)
C15B	0.0226 (7)	0.0299 (8)	0.0210 (7)	0.0046 (6)	0.0049 (5)	0.0033 (6)
C16B	0.0226 (7)	0.0349 (9)	0.0235 (7)	-0.0008 (6)	0.0030 (6)	-0.0016 (7)
C17B	0.0260 (7)	0.0262 (9)	0.0255 (7)	-0.0024 (6)	0.0044 (6)	-0.0017 (6)

O2B	0.0266 (5)	0.0366 (7)	0.0261 (6)	0.0006 (5)	0.0099 (4)	-0.0083 (5)
N2B	0.0257 (6)	0.0375 (9)	0.0264 (7)	0.0028 (6)	0.0016 (5)	0.0060 (6)
O3B	0.0512 (8)	0.0290 (7)	0.0372 (7)	0.0043 (6)	-0.0030 (6)	0.0058 (6)
O4B	0.0412 (7)	0.0486 (9)	0.0468 (8)	-0.0045 (7)	-0.0186 (6)	0.0114 (7)

Geometric parameters (Å, º) for (cu_mn_622p_cu_vesely)

O1A—C2A	1.4268 (19)	O1B—C2B	1.4292 (19)
O1A—C3A	1.4397 (19)	O1B—C3B	1.4355 (19)
N1A—C9A	1.4222 (19)	N1B—C9B	1.423 (2)
N1A—C11A	1.4689 (18)	N1B—C11B	1.4685 (18)
N1A—C1A	1.4814 (18)	N1B—C1B	1.4821 (18)
C1A—C2A	1.5229 (19)	C1B—C2B	1.5224 (19)
C1A—H1A1	0.9900	C1B—H1B1	0.9900
C1A—H1A2	0.9900	C1B—H1B2	0.9900
C2A—C10A	1.524 (2)	C2B—C10B	1.520 (2)
C2A—H2C	1.0000	C2B—H2D	1.0000
C3A—C4A	1.504 (2)	C3B—C4B	1.503 (2)
C3A—H3A1	0.9900	C3B—H3B1	0.9900
C3A—H3A2	0.9900	C3B—H3B2	0.9900
C4A—C5A	1.396 (2)	C4B—C5B	1.393 (2)
C4A—C9A	1.410 (2)	C4B—C9B	1.413 (2)
C5A—C6A	1.393 (2)	C5B—C6B	1.393 (2)
C5A—H5A	0.9500	C5B—H5B	0.9500
C6A—C7A	1.384 (3)	C6B—C7B	1.385 (3)
C6A—H6A	0.9500	C6B—H6B	0.9500
C7A—C8A	1.394 (2)	C7B—C8B	1.394 (2)
C7A—H7A	0.9500	C7B—H7B	0.9500
C8A—C9A	1.403 (2)	C8B—C9B	1.404 (2)
C8A—H8A	0.9500	C8B—H8B	0.9500
C10A—O2A	1.414 (2)	C10B—O2B	1.416 (2)
C10A—H10A	0.9900	C10B—H10C	0.9900
C10A—H10B	0.9900	C10B—H10D	0.9900
C11A—C12A	1.513 (2)	C11B—C12B	1.514 (2)
C11A—H11A	0.9900	C11B—H11C	0.9900
C11A—H11B	0.9900	C11B—H11D	0.9900
C12A—C13A	1.393 (2)	C12B—C13B	1.387 (2)
C12A—C17A	1.399 (2)	C12B—C17B	1.396 (2)

C13A—C14A	1.384 (2)	C13B—C14B	1.394 (2)
C13A—H13A	0.9500	C13B—H13B	0.9500
C14A—C15A	1.383 (2)	C14B—C15B	1.388 (2)
C14A—H14A	0.9500	C14B—H14B	0.9500
C15A—C16A	1.387 (3)	C15B—C16B	1.381 (3)
C15A—N2A	1.469 (2)	C15B—N2B	1.477 (2)
C16A—C17A	1.380 (2)	C16B—C17B	1.382 (2)
C16A—H16A	0.9500	C16B—H16B	0.9500
C17A—H17A	0.9500	C17B—H17B	0.9500
O2A—H2A	0.8819	O2B—H2B	0.8657
N2A—O3A	1.219 (2)	N2B—O3B	1.224 (2)
N2A—O4A	1.226 (2)	N2B—O4B	1.226 (2)
C2A—O1A—C3A	112.93 (11)	C2B—O1B—C3B	113.13 (11)
C9A—N1A—C11A	114.92 (11)	C9B—N1B—C11B	114.30 (11)
C9A—N1A—C1A	113.93 (11)	C9B—N1B—C1B	113.88 (11)
C11A—N1A—C1A	111.43 (12)	C11B—N1B—C1B	111.59 (12)
N1A—C1A—C2A	114.20 (12)	N1B—C1B—C2B	114.90 (13)
N1A—C1A—H1A1	108.7	N1B—C1B—H1B1	108.5
C2A—C1A—H1A1	108.7	C2B—C1B—H1B1	108.5
N1A—C1A—H1A2	108.7	N1B—C1B—H1B2	108.5
C2A—C1A—H1A2	108.7	C2B—C1B—H1B2	108.5
H1A1—C1A—H1A2	107.6	H1B1—C1B—H1B2	107.5
O1A—C2A—C1A	111.38 (12)	O1B—C2B—C10B	107.57 (12)
O1A—C2A—C10A	107.96 (12)	O1B—C2B—C1B	111.64 (12)
C1A—C2A—C10A	110.67 (13)	C10B—C2B—C1B	110.94 (14)
O1A—C2A—H2C	108.9	O1B—C2B—H2D	108.9
C1A—C2A—H2C	108.9	C10B—C2B—H2D	108.9
C10A—C2A—H2C	108.9	C1B—C2B—H2D	108.9
O1A—C3A—C4A	114.94 (12)	O1B—C3B—C4B	114.76 (13)
O1A—C3A—H3A1	108.5	O1B—C3B—H3B1	108.6
C4A—C3A—H3A1	108.5	C4B—C3B—H3B1	108.6
O1A—C3A—H3A2	108.5	O1B—C3B—H3B2	108.6
C4A—C3A—H3A2	108.5	C4B—C3B—H3B2	108.6
H3A1—C3A—H3A2	107.5	H3B1—C3B—H3B2	107.6
C5A—C4A—C9A	119.52 (14)	C5B—C4B—C9B	119.53 (14)
C5A—C4A—C3A	118.47 (14)	C5B—C4B—C3B	118.70 (14)

C9A—C4A—C3A	121.68 (14)	C9B—C4B—C3B	121.51 (14)
C6A—C5A—C4A	121.31 (15)	C4B—C5B—C6B	121.60 (15)
C6A—C5A—H5A	119.3	C4B—C5B—H5B	119.2
C4A—C5A—H5A	119.3	C6B—C5B—H5B	119.2
C7A—C6A—C5A	119.17 (15)	C7B—C6B—C5B	118.95 (16)
C7A—C6A—H6A	120.4	C7B—C6B—H6B	120.5
C5A—C6A—H6A	120.4	C5B—C6B—H6B	120.5
C6A—C7A—C8A	120.52 (15)	C6B—C7B—C8B	120.43 (16)
C6A—C7A—H7A	119.7	C6B—C7B—H7B	119.8
C8A—C7A—H7A	119.7	C8B—C7B—H7B	119.8
C7A—C8A—C9A	120.79 (15)	C7B—C8B—C9B	121.11 (15)
C7A—C8A—H8A	119.6	C7B—C8B—H8B	119.4
C9A—C8A—H8A	119.6	C9B—C8B—H8B	119.4
C8A—C9A—C4A	118.62 (14)	C8B—C9B—C4B	118.25 (14)
C8A—C9A—N1A	122.50 (14)	C8B—C9B—N1B	122.61 (14)
C4A—C9A—N1A	118.87 (12)	C4B—C9B—N1B	119.12 (13)
O2A—C10A—C2A	109.70 (12)	O2B—C10B—C2B	109.44 (12)
O2A—C10A—H10A	109.7	O2B—C10B—H10C	109.8
C2A—C10A—H10A	109.7	C2B—C10B—H10C	109.8
O2A—C10A—H10B	109.7	O2B—C10B—H10D	109.8
C2A—C10A—H10B	109.7	C2B—C10B—H10D	109.8
H10A—C10A—H10B	108.2	H10C—C10B—H10D	108.2
N1A—C11A—C12A	111.58 (12)	N1B—C11B—C12B	112.23 (12)
N1A—C11A—H11A	109.3	N1B—C11B—H11C	109.2
C12A—C11A—H11A	109.3	C12B—C11B—H11C	109.2
N1A—C11A—H11B	109.3	N1B—C11B—H11D	109.2
C12A—C11A—H11B	109.3	C12B—C11B—H11D	109.2
H11A—C11A—H11B	108.0	H11C—C11B—H11D	107.9
C13A—C12A—C17A	119.16 (15)	C13B—C12B—C17B	119.33 (16)
C13A—C12A—C11A	120.19 (14)	C13B—C12B—C11B	120.38 (14)
C17A—C12A—C11A	120.65 (15)	C17B—C12B—C11B	120.27 (15)
C14A—C13A—C12A	120.56 (15)	C12B—C13B—C14B	121.15 (15)
C14A—C13A—H13A	119.7	C12B—C13B—H13B	119.4
C12A—C13A—H13A	119.7	C14B—C13B—H13B	119.4
C15A—C14A—C13A	118.75 (15)	C15B—C14B—C13B	117.46 (16)
C15A—C14A—H14A	120.6	C15B—C14B—H14B	121.3
C13A—C14A—H14A	120.6	C13B—C14B—H14B	121.3

C14A—C15A—C16A	122.24 (15)	C16B—C15B—C14B	122.93 (16)
C14A—C15A—N2A	119.12 (16)	C16B—C15B—N2B	118.78 (15)
C16A—C15A—N2A	118.64 (15)	C14B—C15B—N2B	118.29 (16)
C17A—C16A—C15A	118.26 (15)	C15B—C16B—C17B	118.35 (16)
C17A—C16A—H16A	120.9	C15B—C16B—H16B	120.8
C15A—C16A—H16A	120.9	C17B—C16B—H16B	120.8
C16A—C17A—C12A	120.98 (16)	C16B—C17B—C12B	120.76 (16)
C16A—C17A—H17A	119.5	C16B—C17B—H17B	119.6
C12A—C17A—H17A	119.5	C12B—C17B—H17B	119.6
C10A—O2A—H2A	112.1	C10B—O2B—H2B	113.9
O3A—N2A—O4A	123.33 (17)	O3B—N2B—O4B	123.97 (16)
O3A—N2A—C15A	118.62 (16)	O3B—N2B—C15B	118.72 (15)
O4A—N2A—C15A	118.04 (16)	O4B—N2B—C15B	117.29 (16)

Hydrogen-bond geometry (Å, º) for (cu_mn_622p_cu_vesely)

D—H···*A*	*D*—H	H···*A*	*D*···*A*	*D*—H···*A*
O2*A*—H2*A*···O1*A*^{i}	0.88	1.87	2.7441 (15)	172
O2*B*—H2*B*···O1*B*^{ii}	0.87	1.88	2.7422 (15)	171

Symmetry codes: (i) -*x*+1, *y*+1/2, -*z*; (ii) -*x*+1, *y*+1/2, -*z*+1.

Document origin: *publCIF* [Westrip, S. P. (2010). *J. Apply. Cryst.*, **43**, 920-925].

NMR DATA

1H NMR: (400 MHz, CDCl3): N-Phenyloxetan-3-amine (S1)

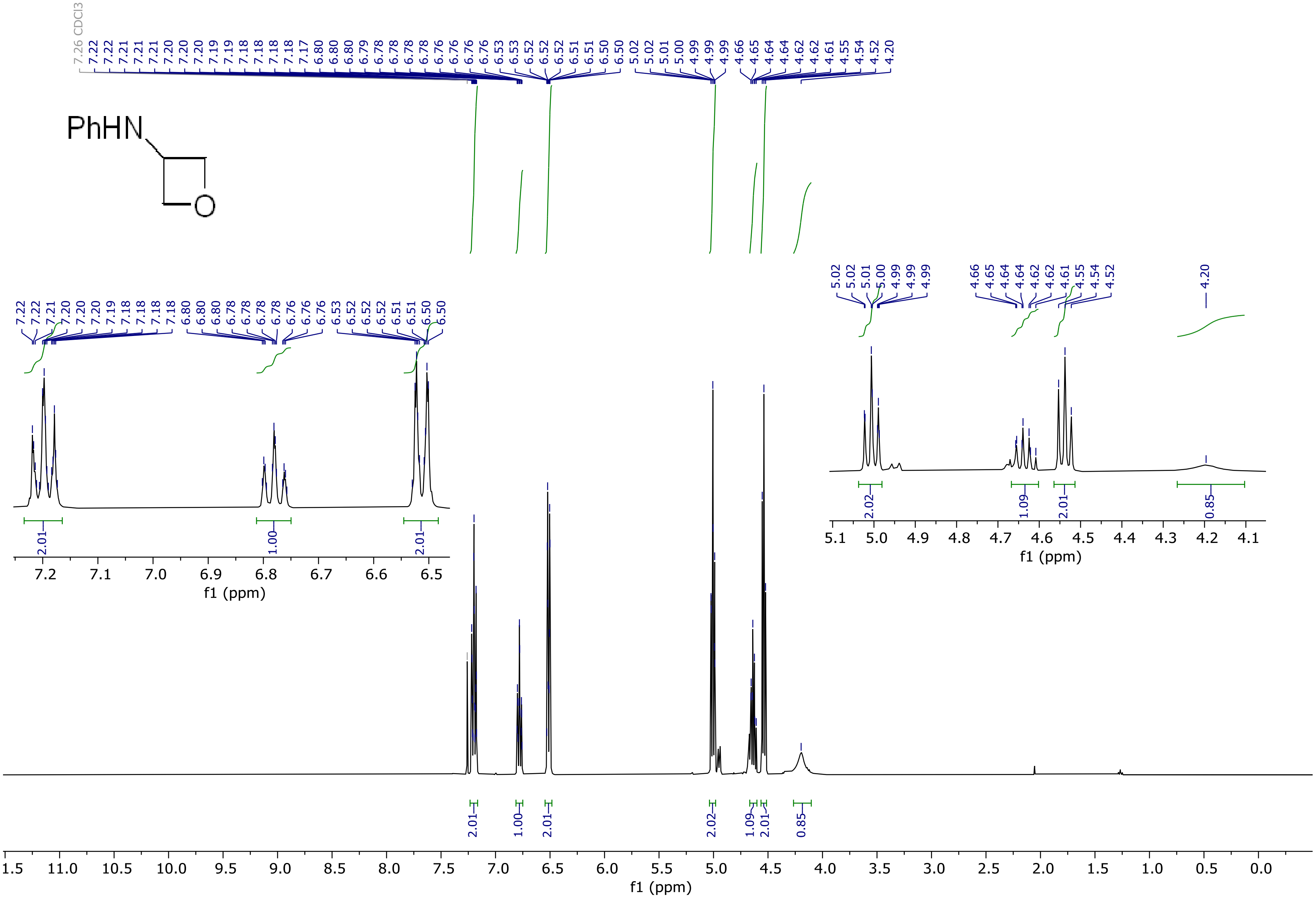

13C{1H}NMR (101 MHz, CDCl3): N-Phenyloxetan-3-amine (S1)

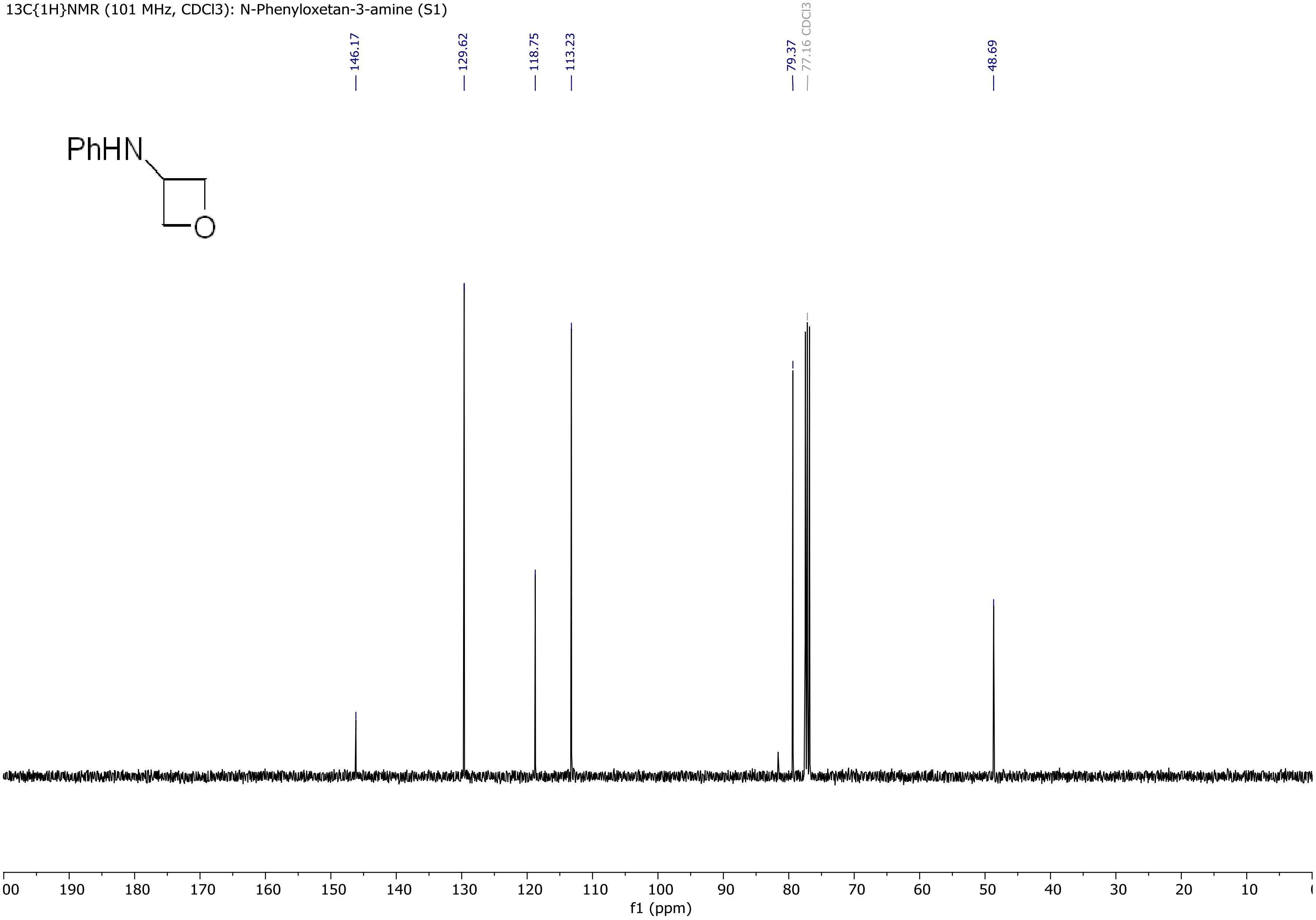

1H NMR: (400 MHz, CDCl3): 3-((2-Nitrobenzyl)oxy)oxetane (S2)

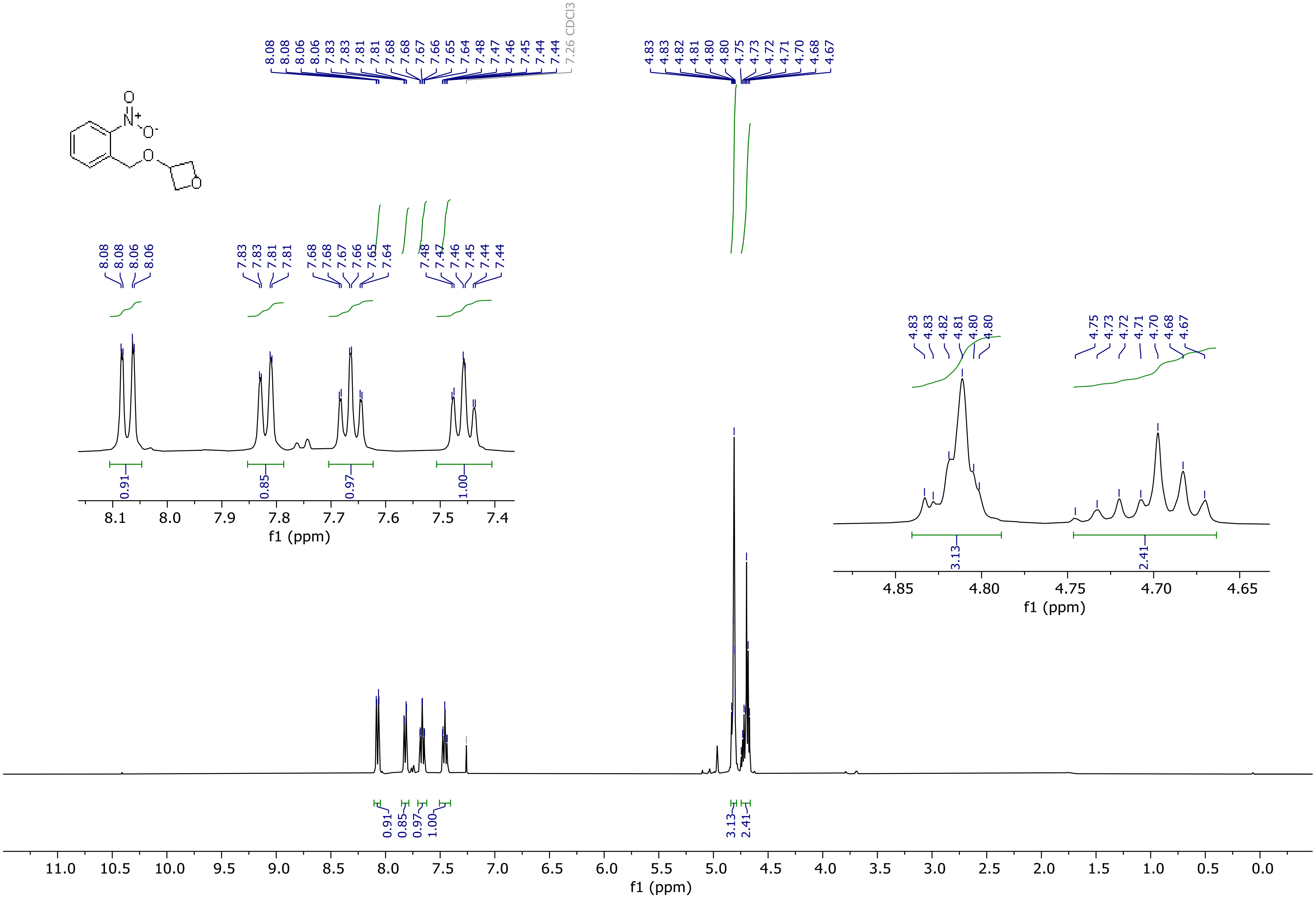

13C{1H}NMR (101 MHz, CDCl3): 3-((2-Nitrobenzyl)oxy)oxetane (S2)

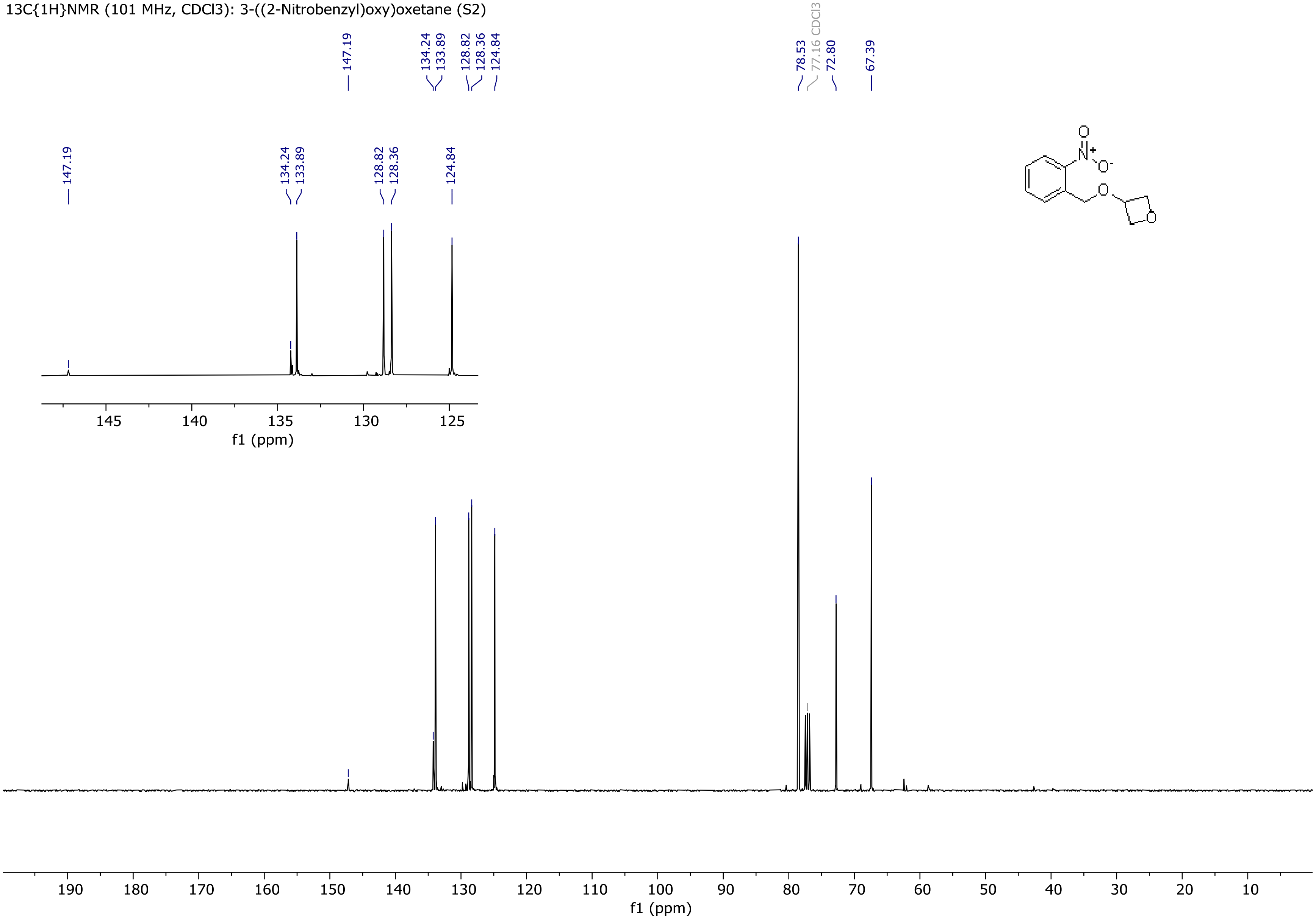

1H NMR: (400 MHz, CDCl3): 3-((3-Methyl-2-nitrobenzyl)oxy)oxetane (S3)

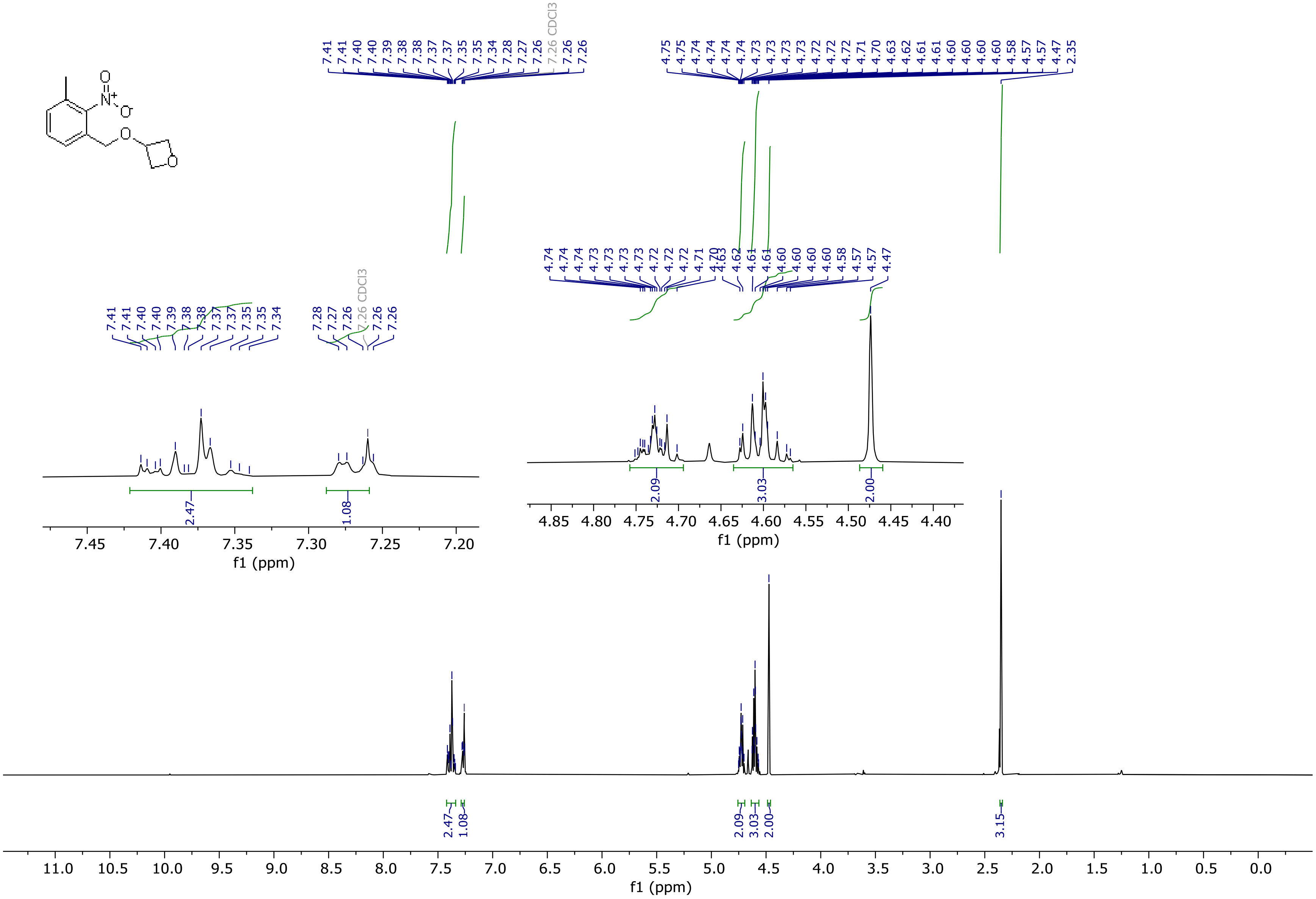

13C{1H}NMR (101 MHz, CDCl3): 3-((3-Methyl-2-nitrobenzyl)oxy)oxetane (S3)

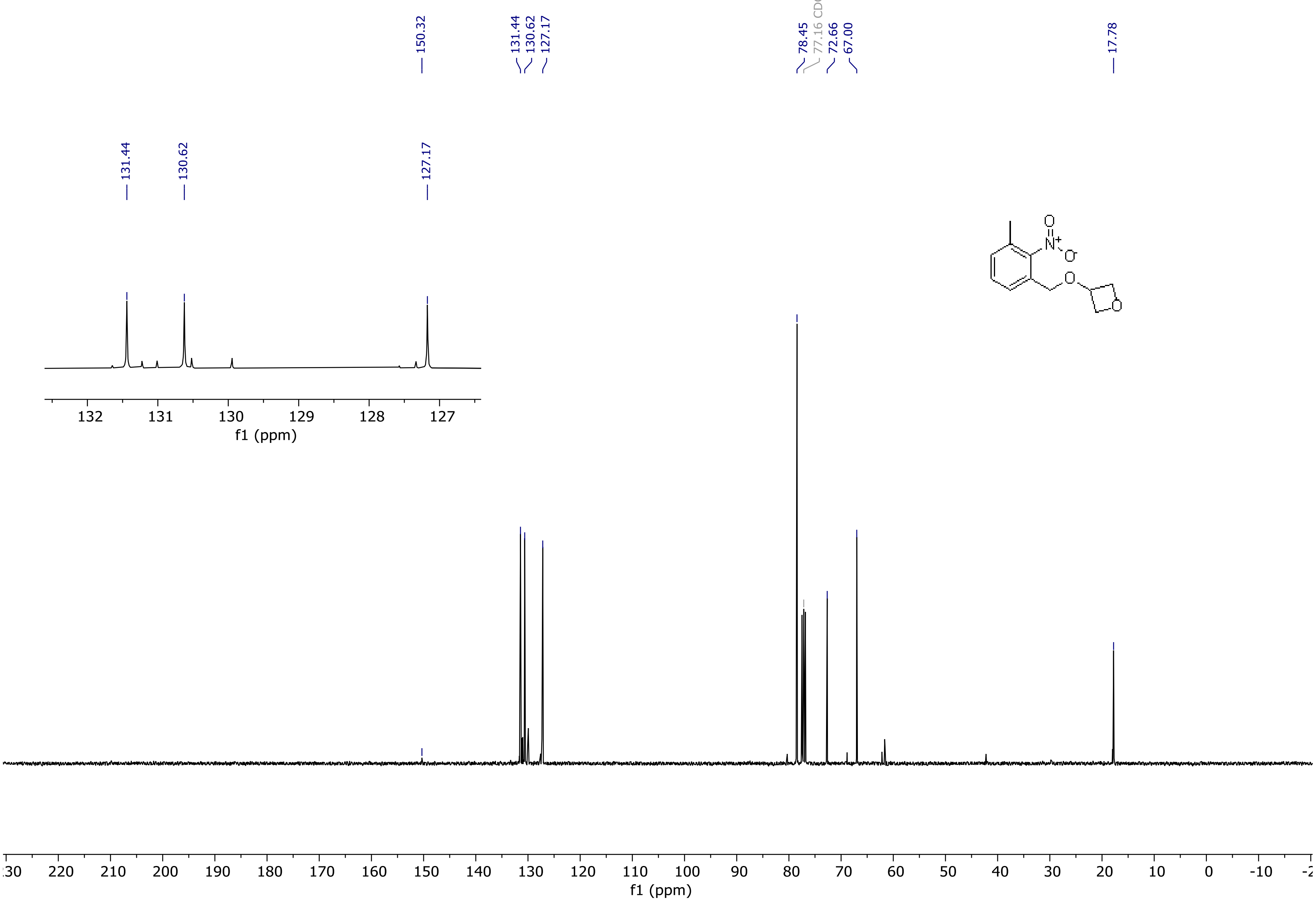

1H NMR: (400 MHz, CDCl3): 3-((4-Chloro-2-nitrobenzyl)oxy)oxetane (S4)

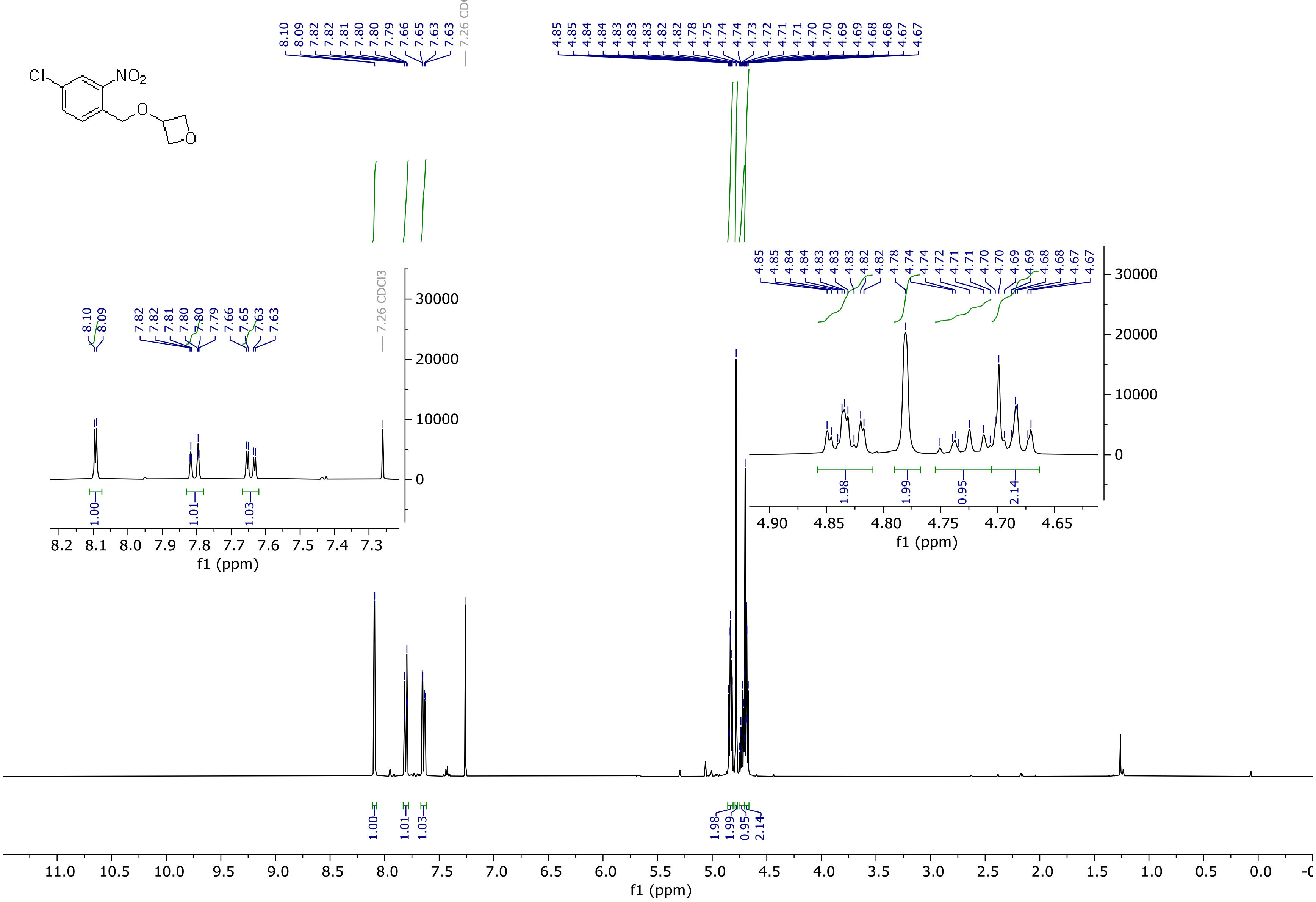

13C{1H}NMR (101 MHz, CDCl3): 3-((4-Chloro-2-nitrobenzyl)oxy)oxetane (S4)

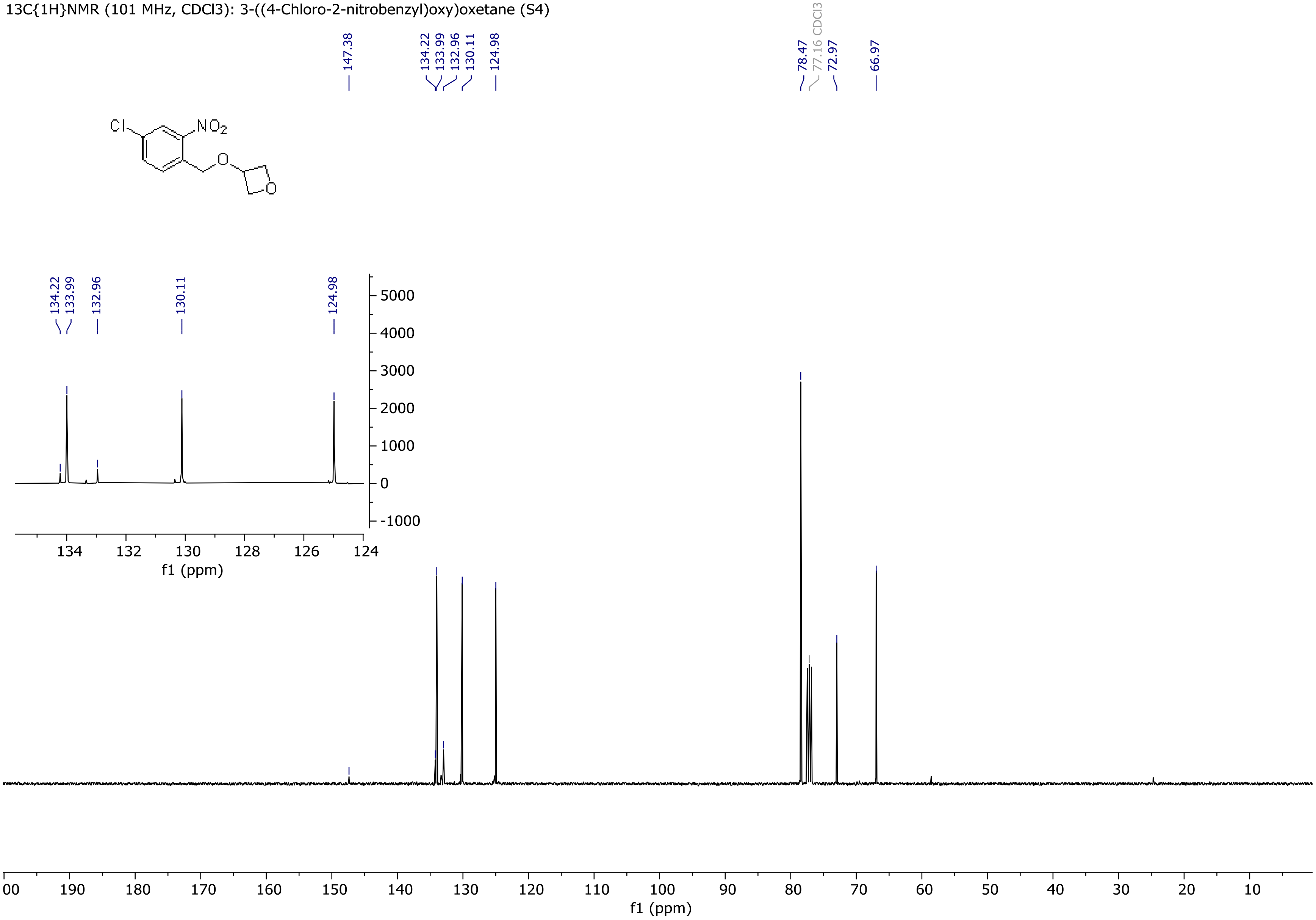

1H NMR: (400 MHz, CDCl3): 3-((4-Fluoro-2-nitrobenzyl)oxy)oxetane (S5)

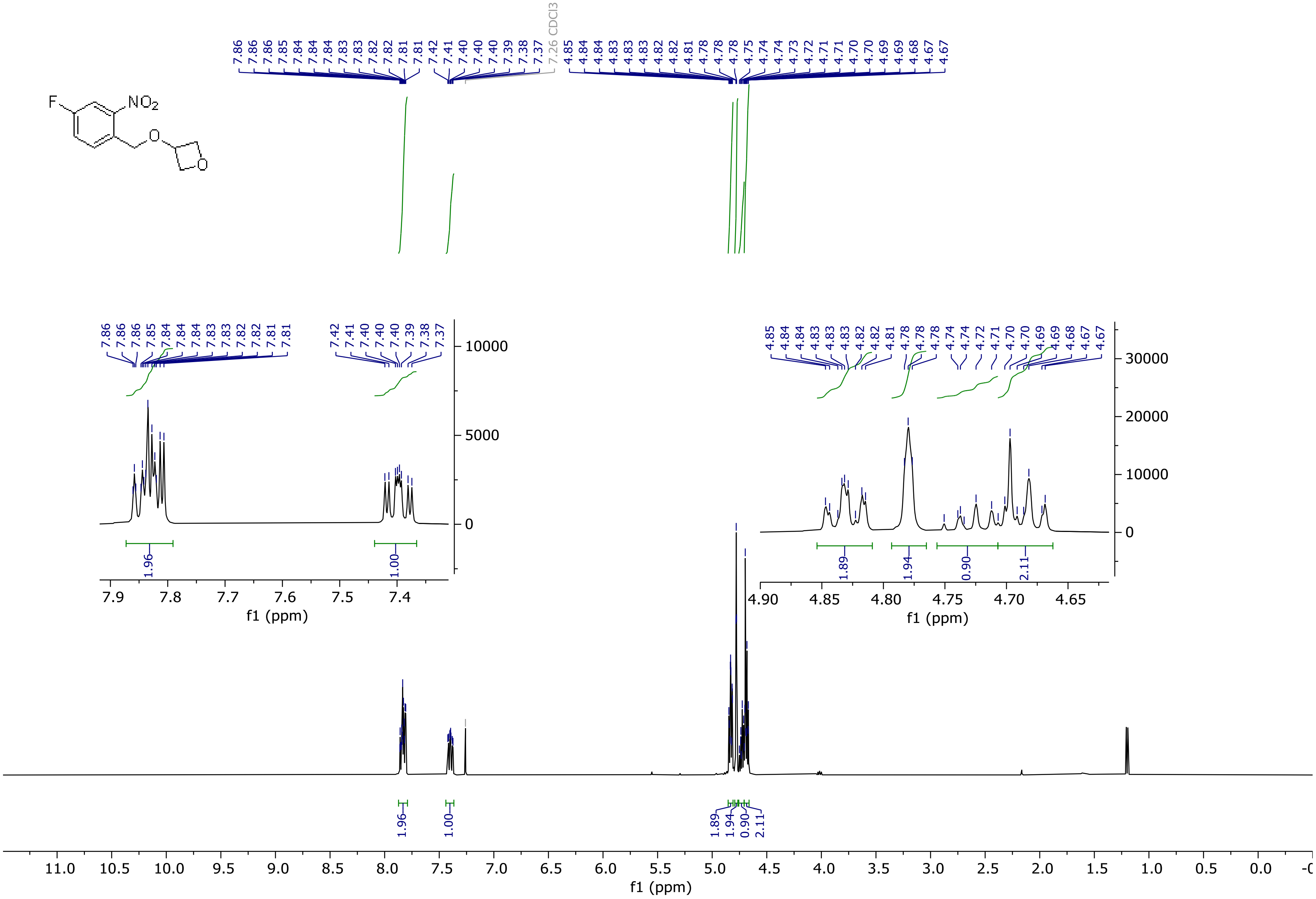

13C{1H}NMR (101 MHz, CDCl3): 3-((4-Fluoro-2-nitrobenzyl)oxy)oxetane (S5)

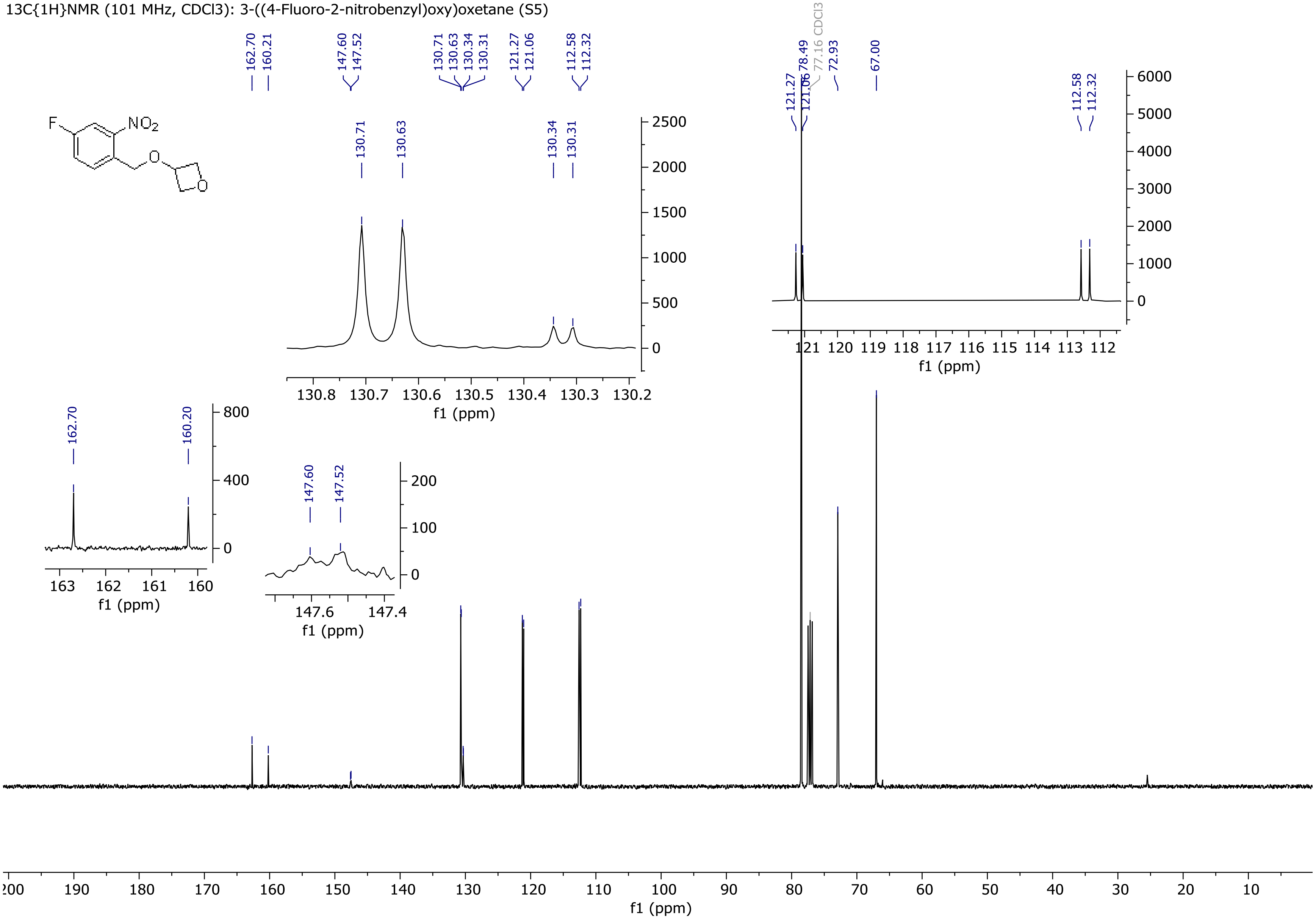

19F NMR (376 MHz, CDCl3): 3-((4-Fluoro-2-nitrobenzyl)oxy)oxetane (S5)

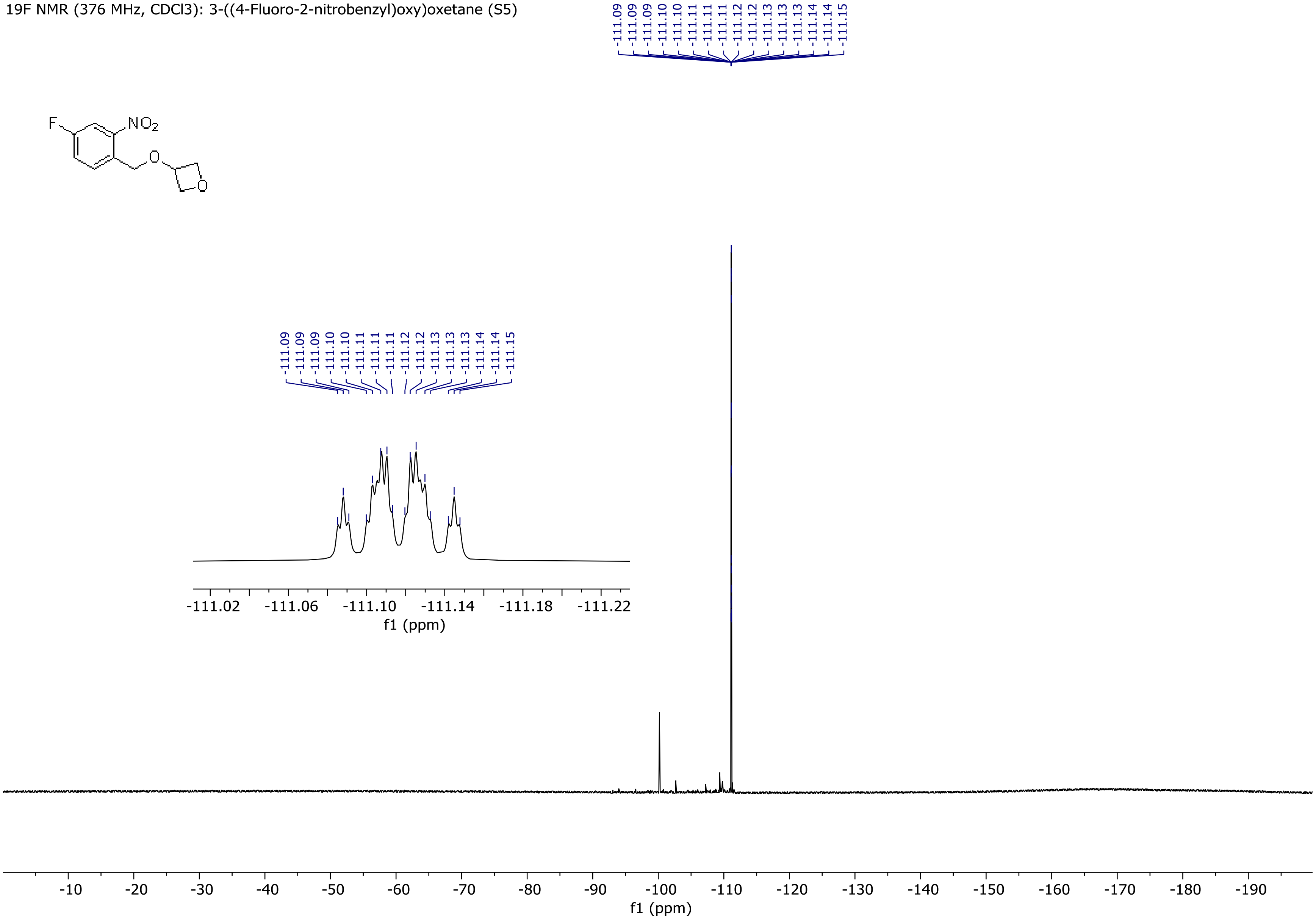

1H NMR: (400 MHz, CDCl3): N-(2-Nitrobenzyl)-N-phenyloxetan-3-amine (S6)

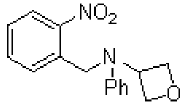

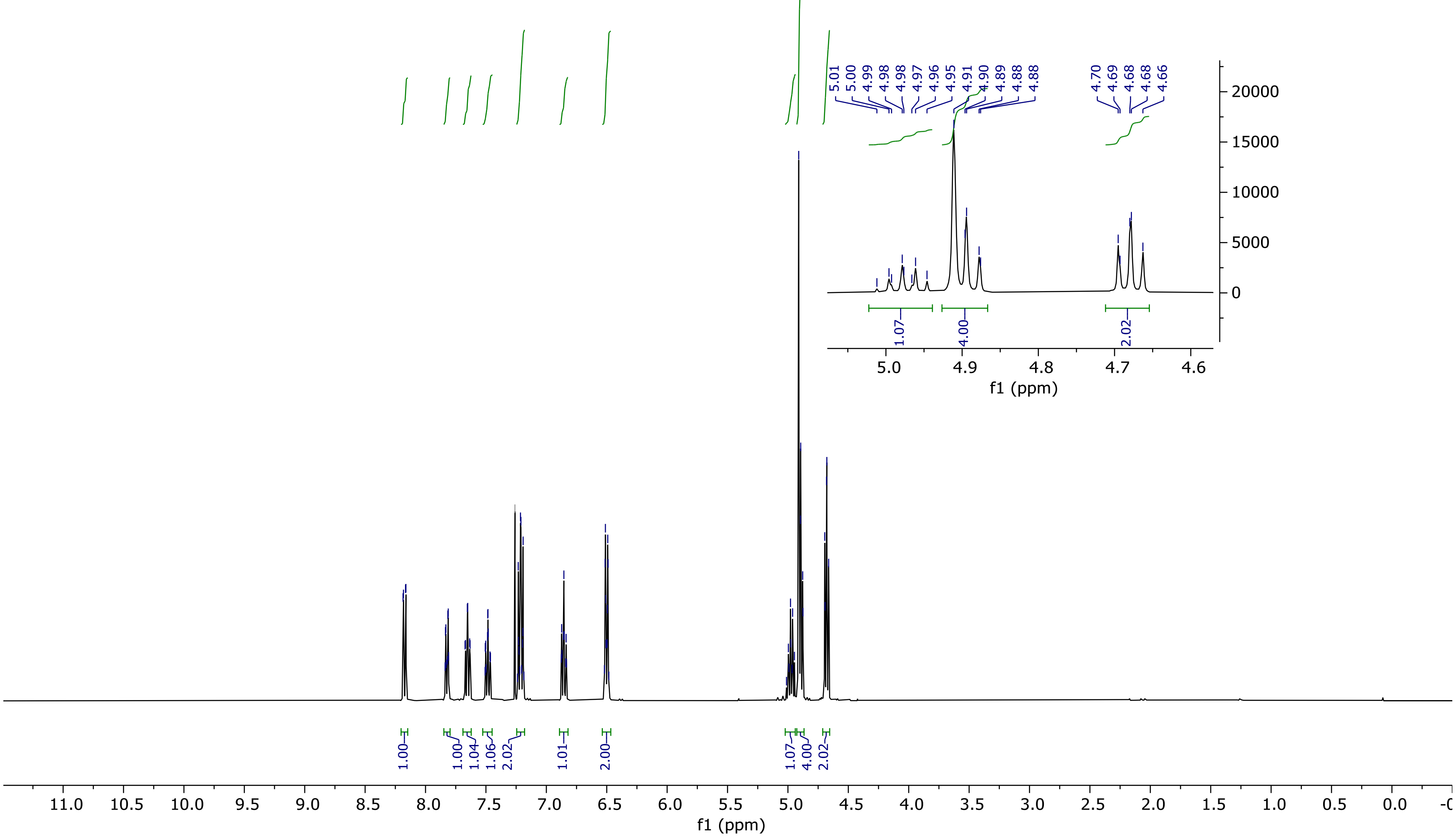

13C{1H}NMR (101 MHz, CDCl3): N-(2-Nitrobenzyl)-N-phenyloxetan-3-amine (S6)

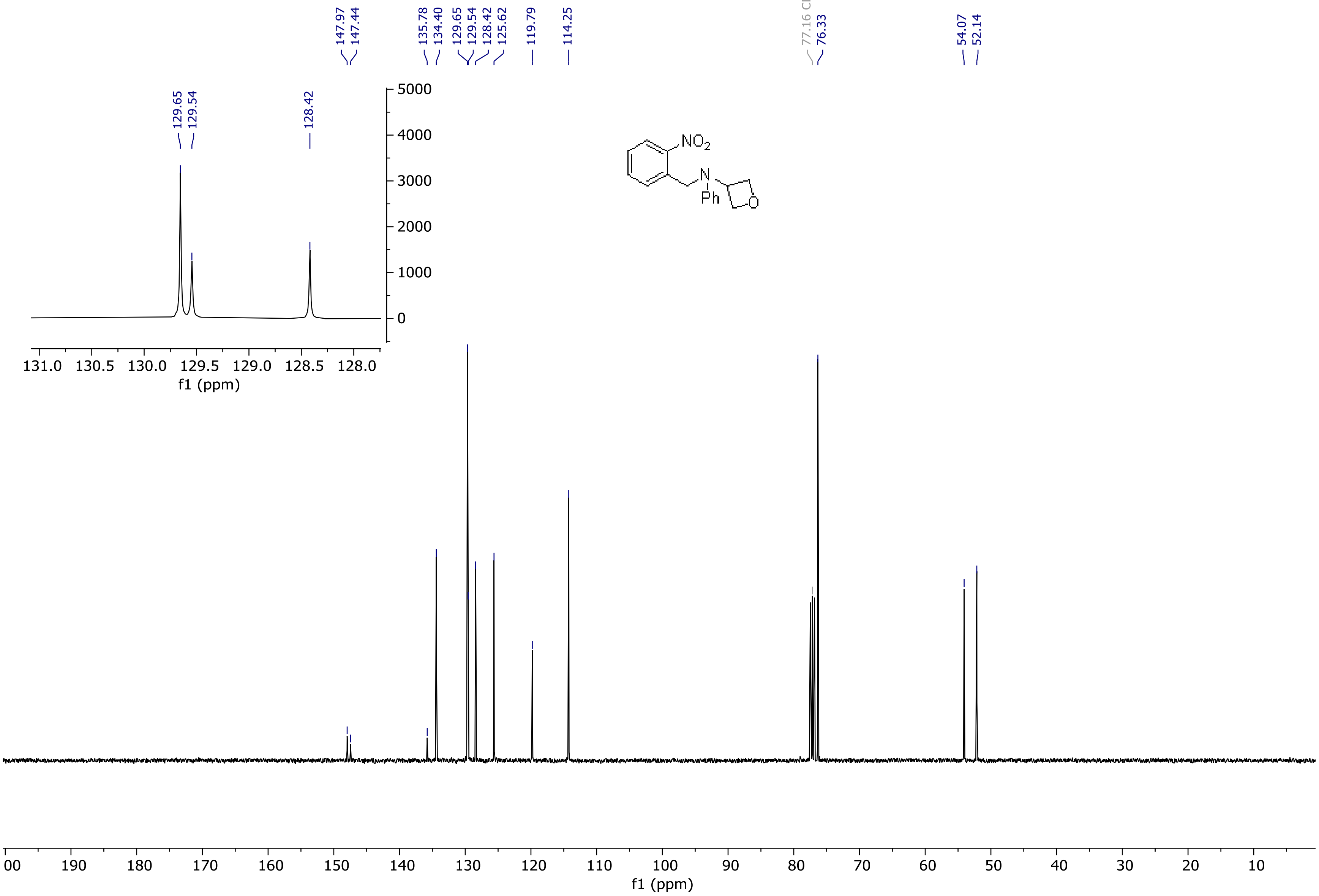

1H NMR (400 MHz, CDCl3): 3-((4-methyl-2-nitrobenzyl)oxy)oxetane (S7)

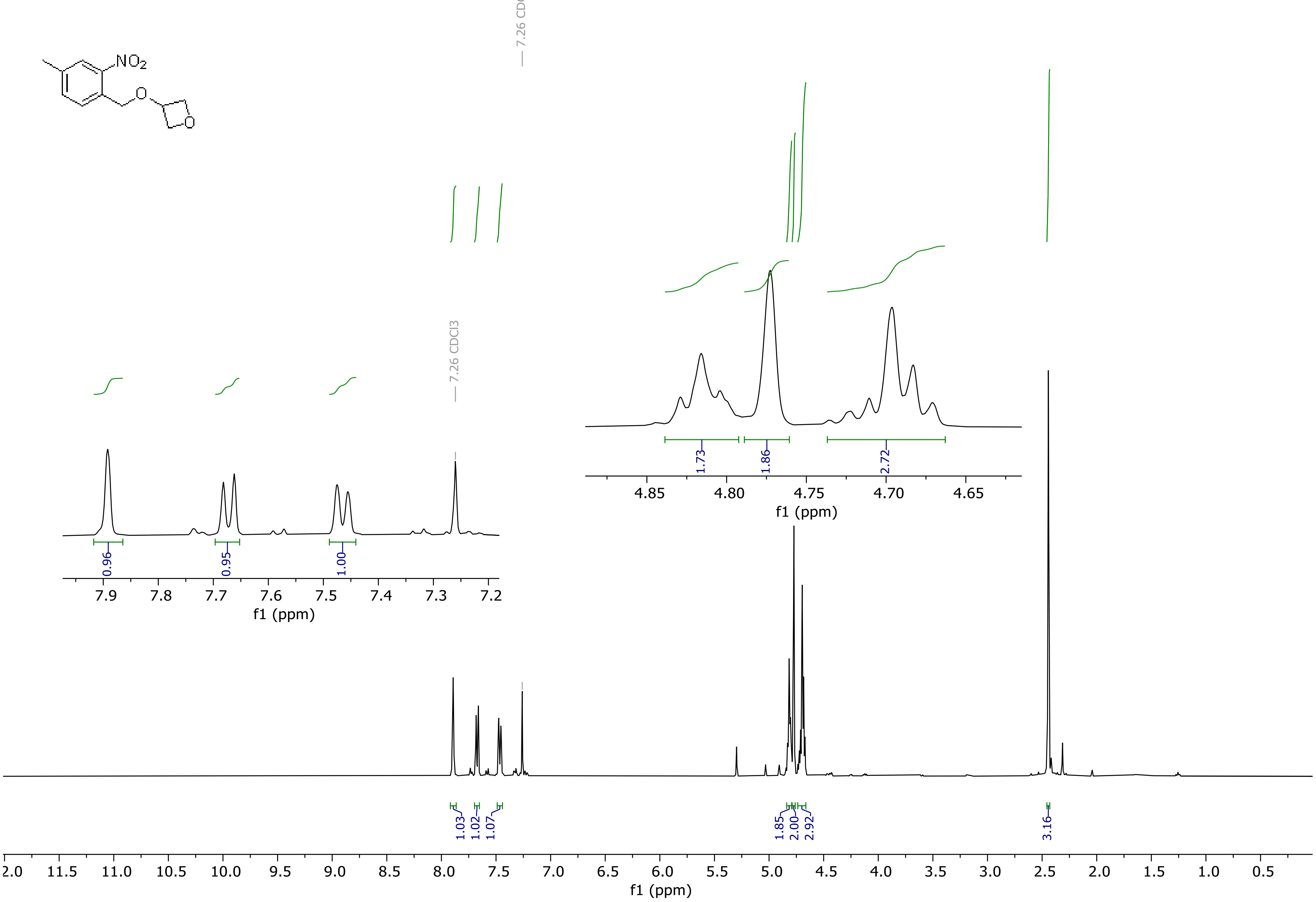

13C{1H}NMR (101 MHz, CDCl3): 3-((4-methyl-2-nitrobenzyl)oxy)oxetane (S7)

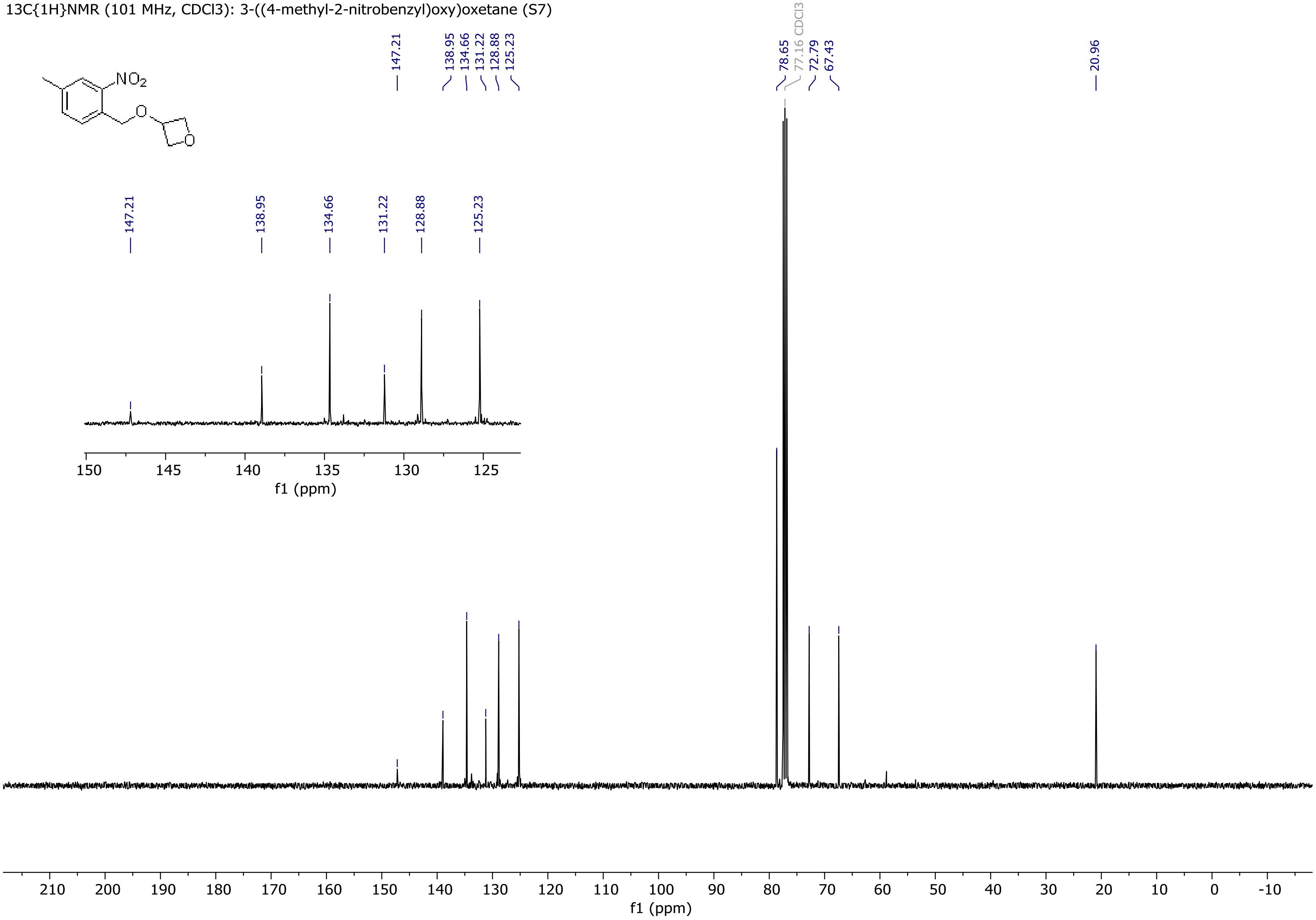

1H NMR (400 MHz, CDCl3): 3-((4-methoxy-2-nitrobenzyl)oxy)oxetane (S8)

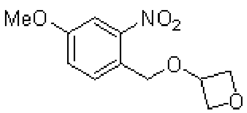

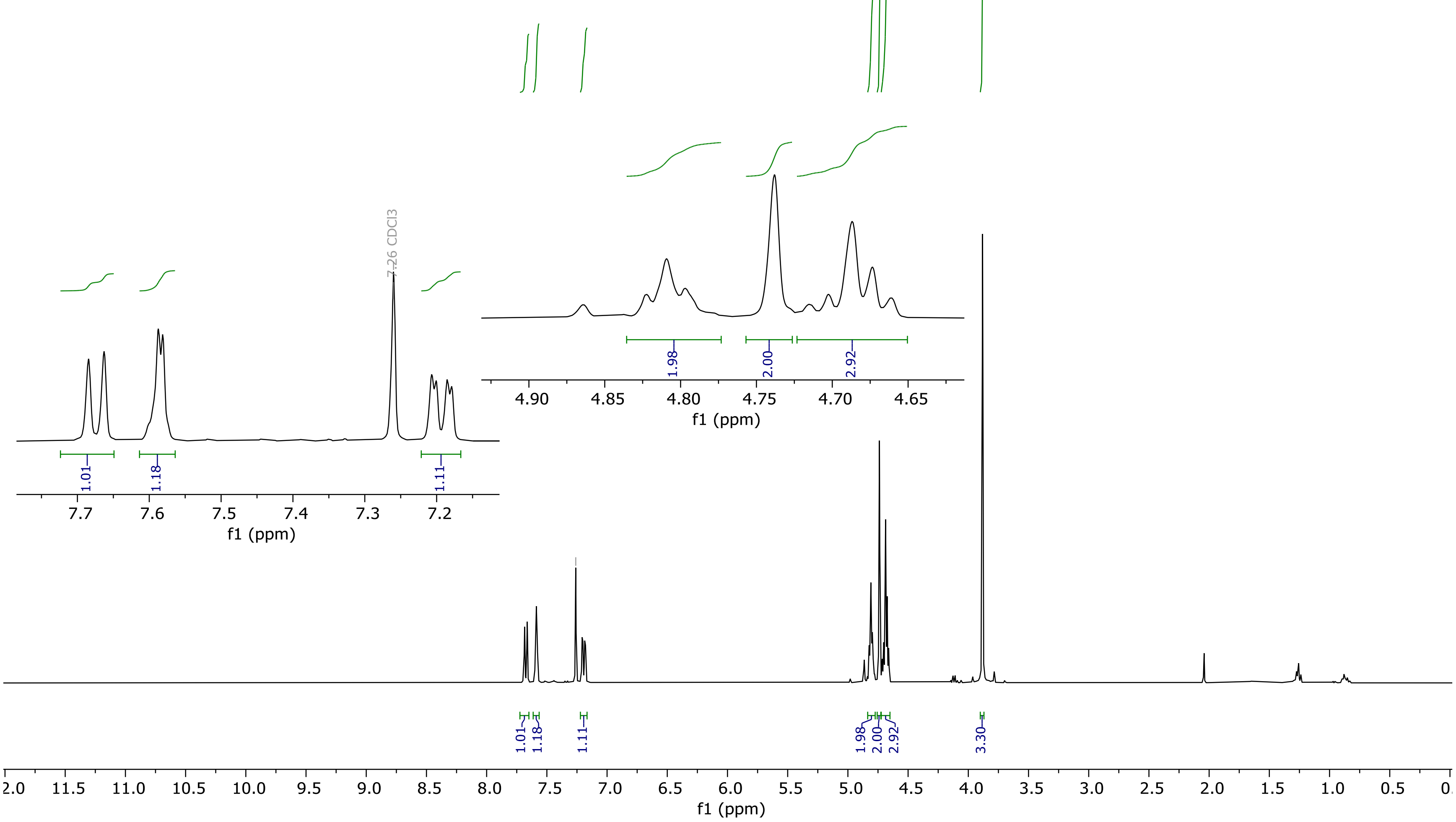

13C{1H}NMR (101 MHz, CDCl3): 3-((4-methoxy-2-nitrobenzyl)oxy)oxetane (S8)

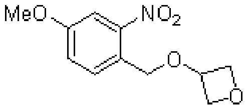

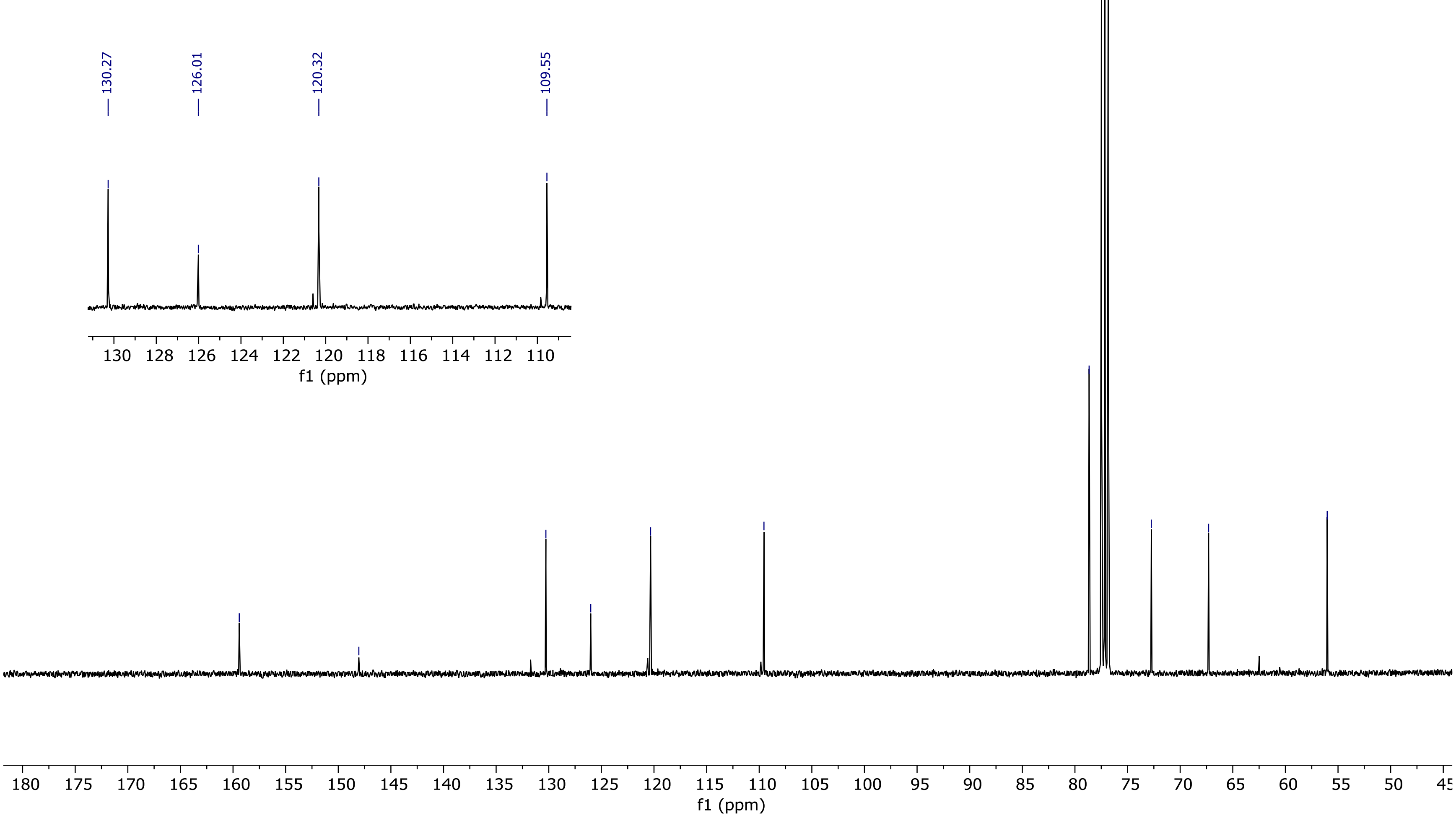

1H NMR: (400 MHz, CDCl3): 2-((Oxetan-3-yloxy)methyl)aniline (S9)

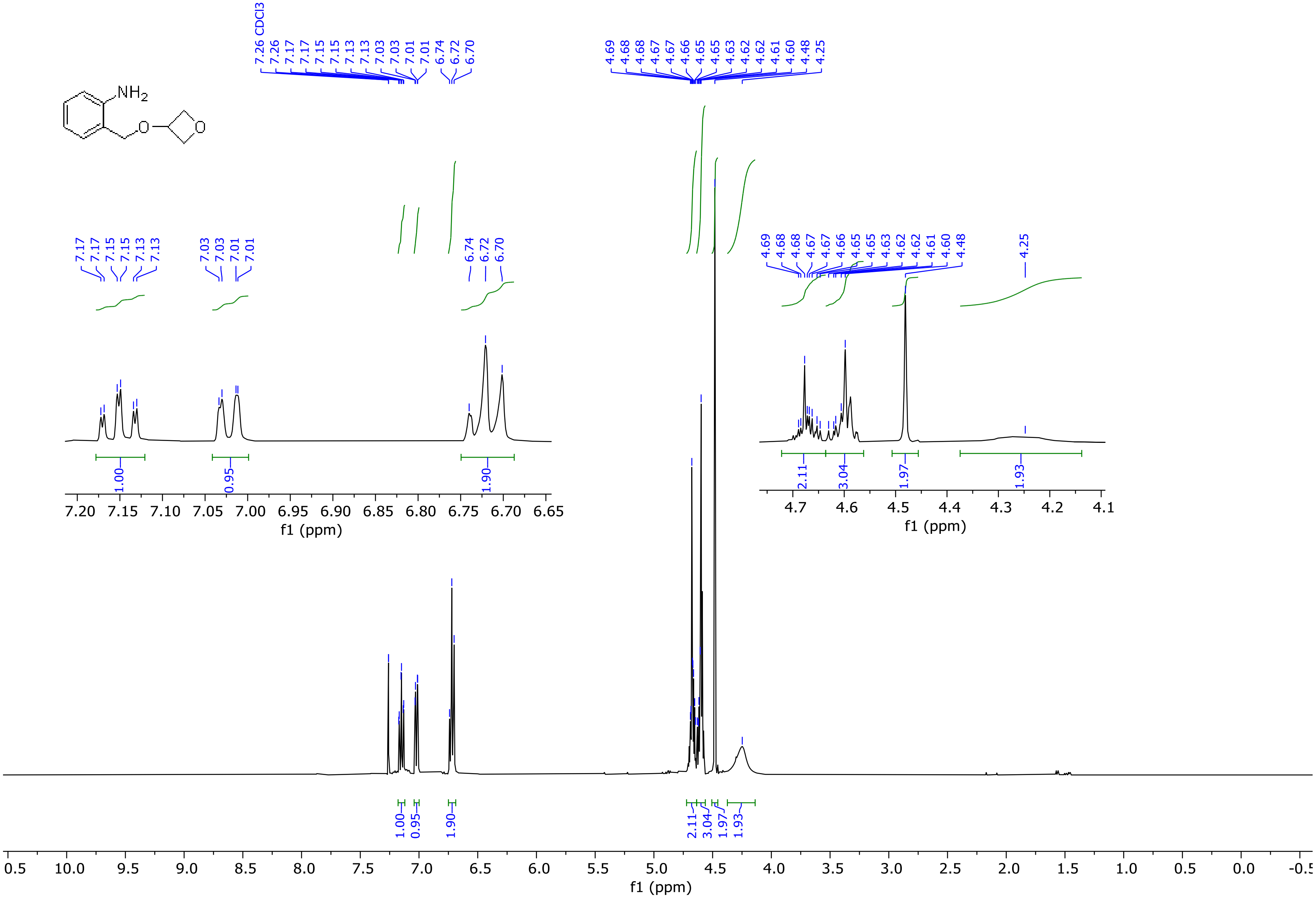

13C{1H}NMR (101 MHz, CDCl3): 2-((Oxetan-3-yloxy)methyl)aniline (S9)

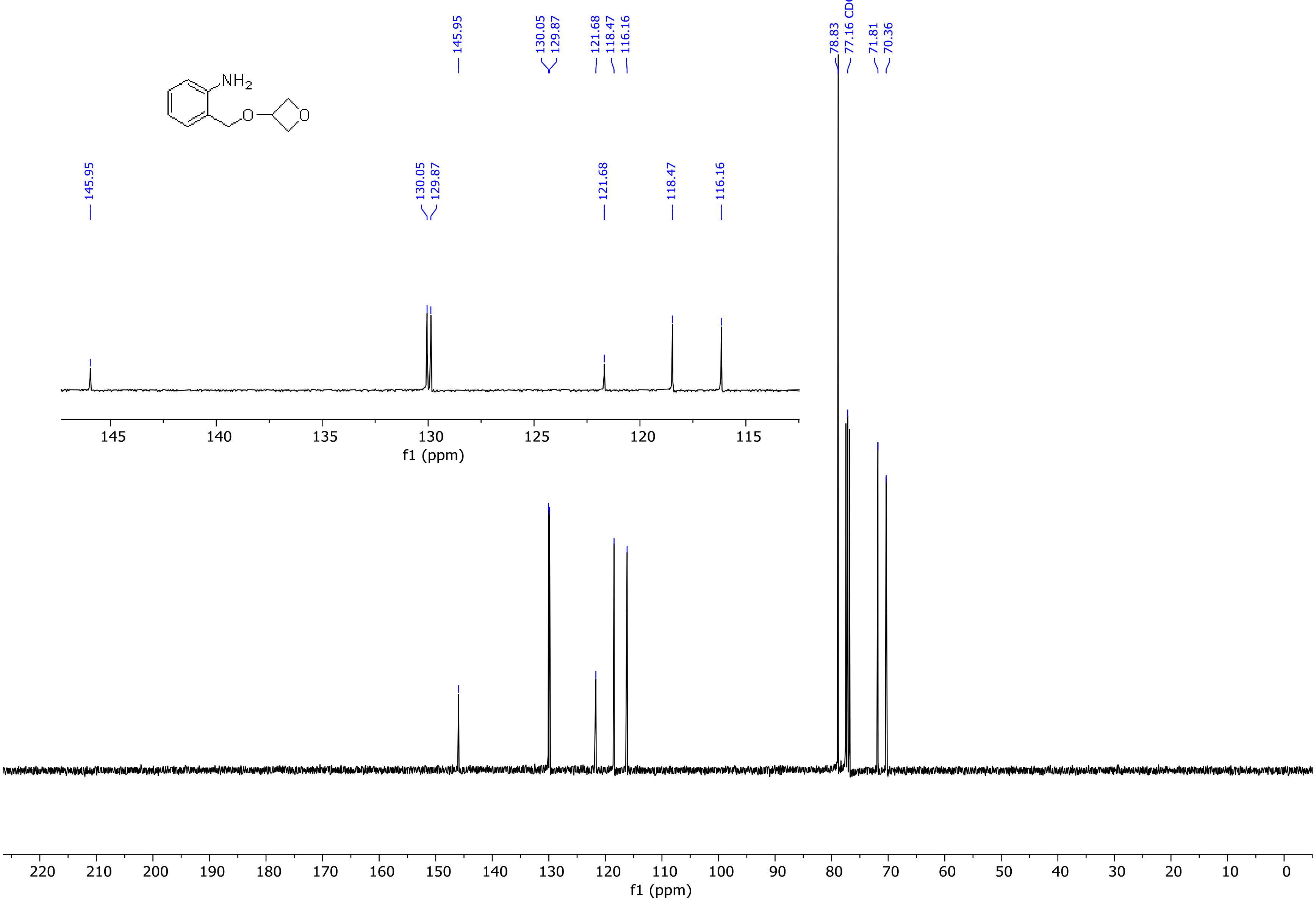

1H NMR: (400 MHz, CDCl3): 2-Methyl-6-((oxetan-3-yloxy)methyl)aniline (S10)

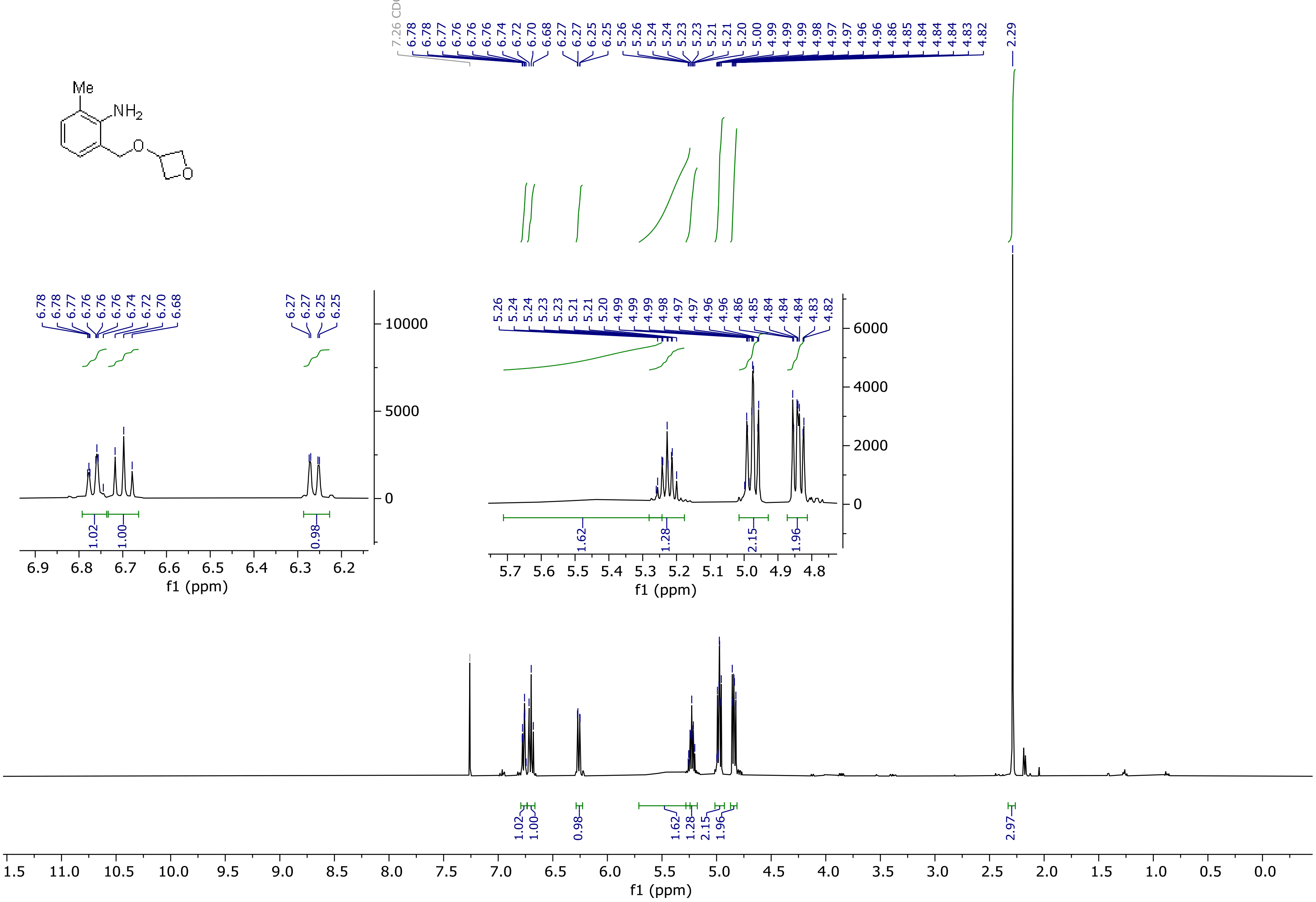

13C{1H}NMR (101 MHz, CDCl3): 2-Methyl-6-((oxetan-3-yloxy)methyl)aniline (S10)

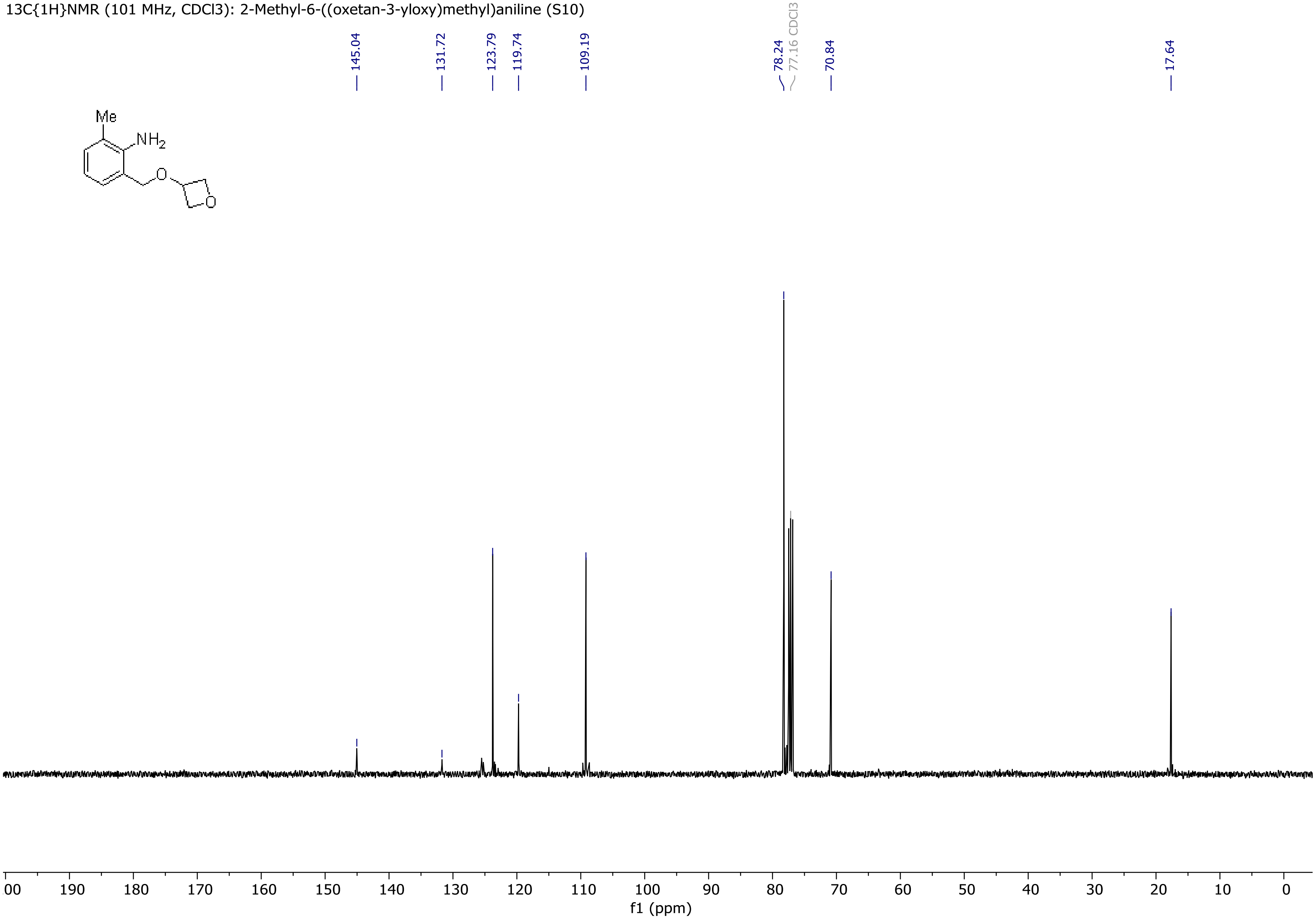

1H NMR: (400 MHz, CDCl3): 5-Chloro-2-((oxetan-3-yloxy)methyl)aniline (S11)

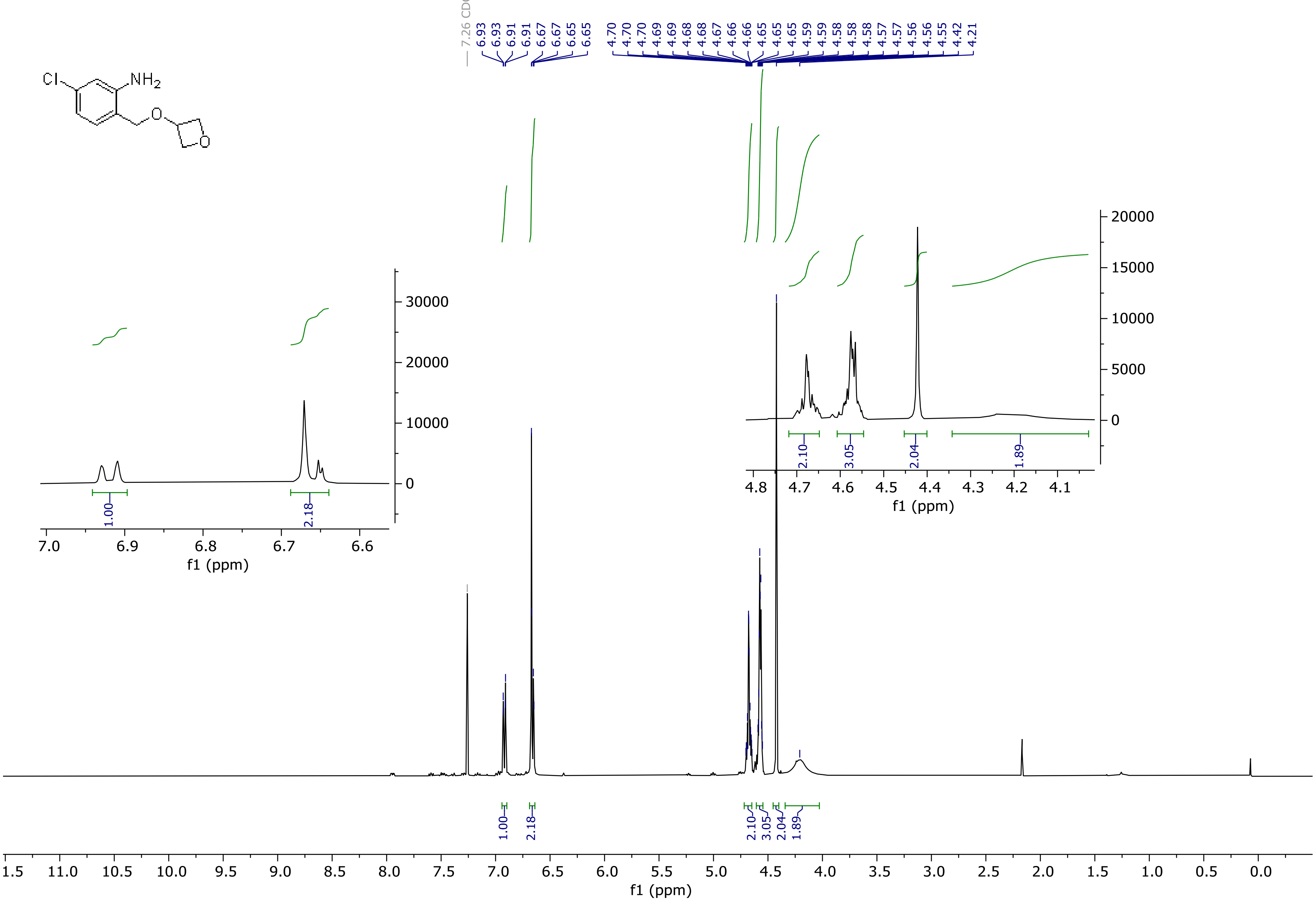

13C{1H}NMR (101 MHz, CDCl3): 5-Chloro-2-((oxetan-3-yloxy)methyl)aniline (S11)

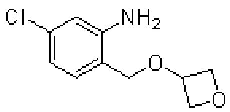

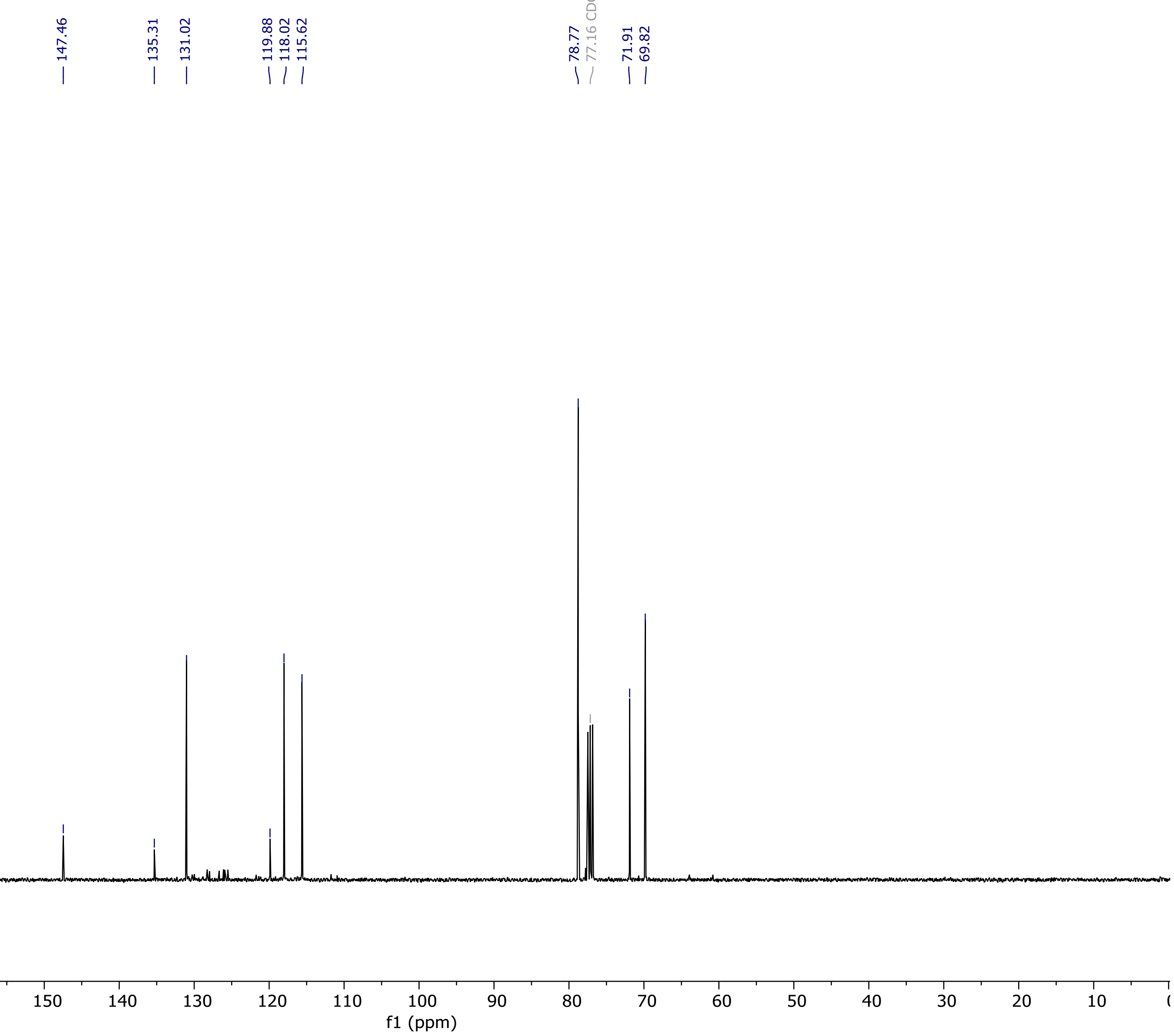

1H NMR: (400 MHz, CDCl3): 5-Fluoro-2-((oxetan-3-yloxy)methyl)aniline (S12)

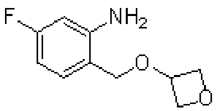

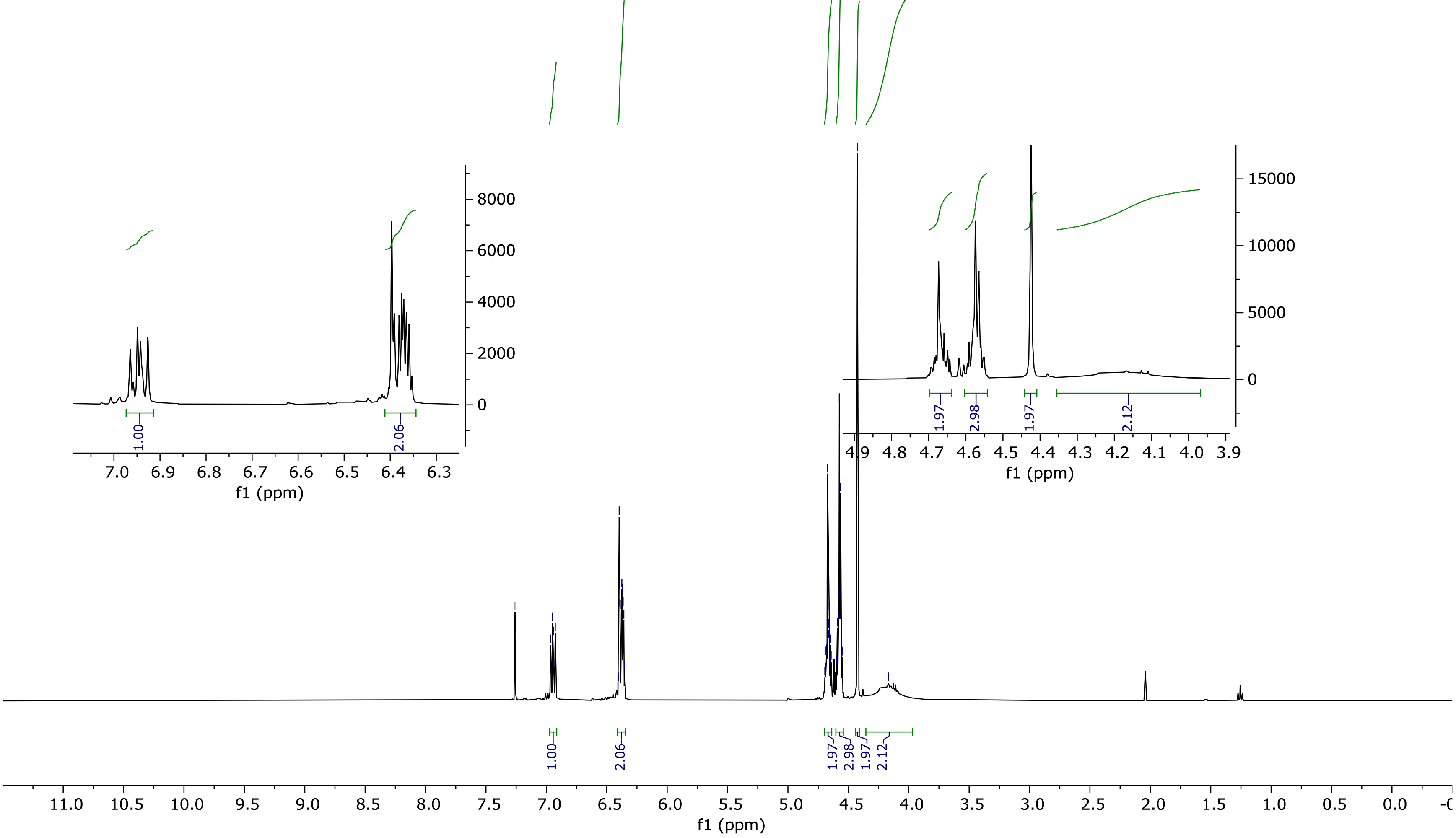

13C{1H}NMR (101 MHz, CDCl3): 5-Fluoro-2-((oxetan-3-yloxy)methyl)aniline (S12)

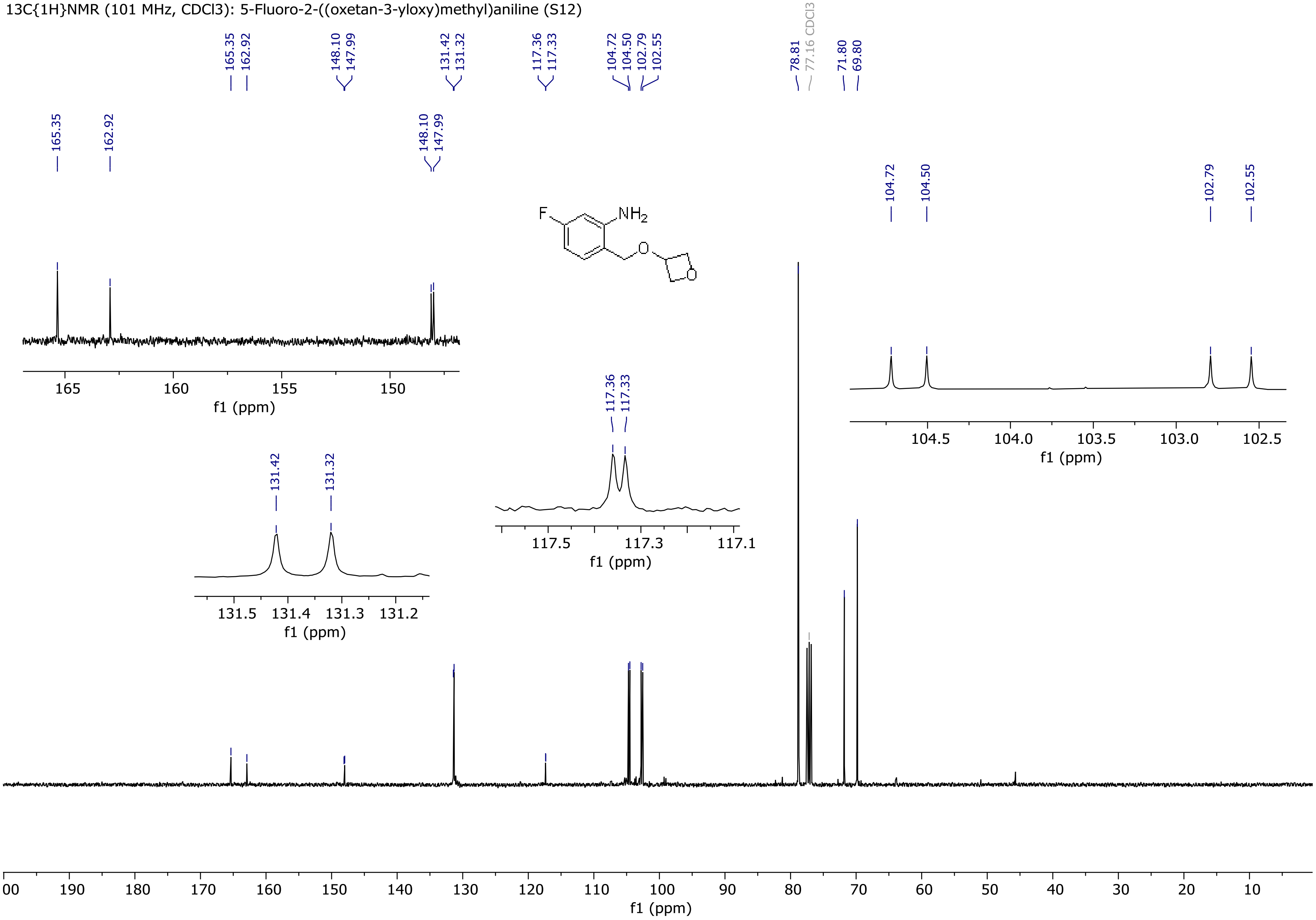

19F NMR (376 MHz, CDCl3): 5-Fluoro-2-((oxetan-3-yloxy)methyl)aniline (S12)

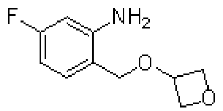

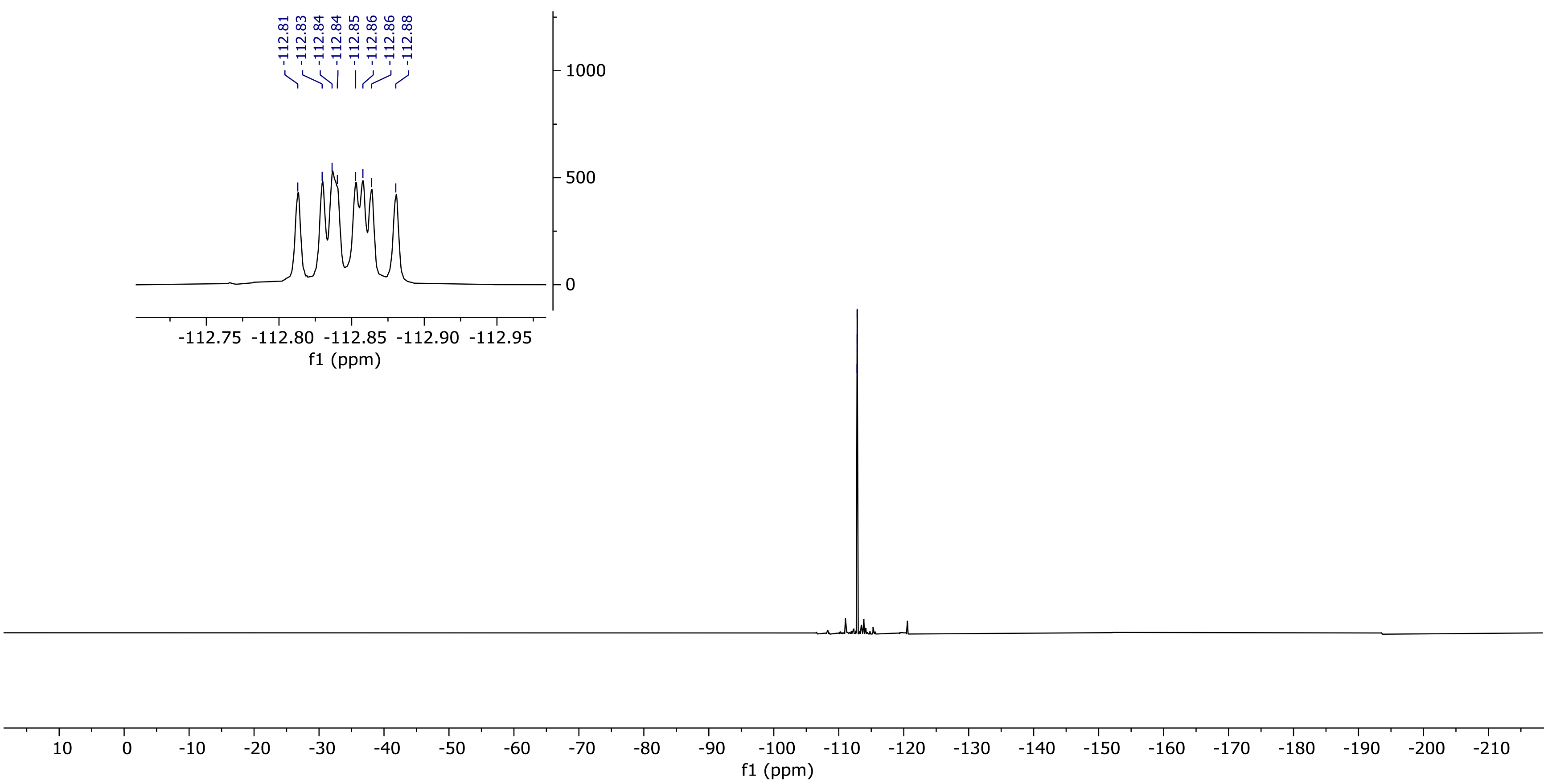

1H NMR (400 MHz, CDCl3): 5-methyl-2-((oxetan-3-yloxy)methyl)aniline (S13)

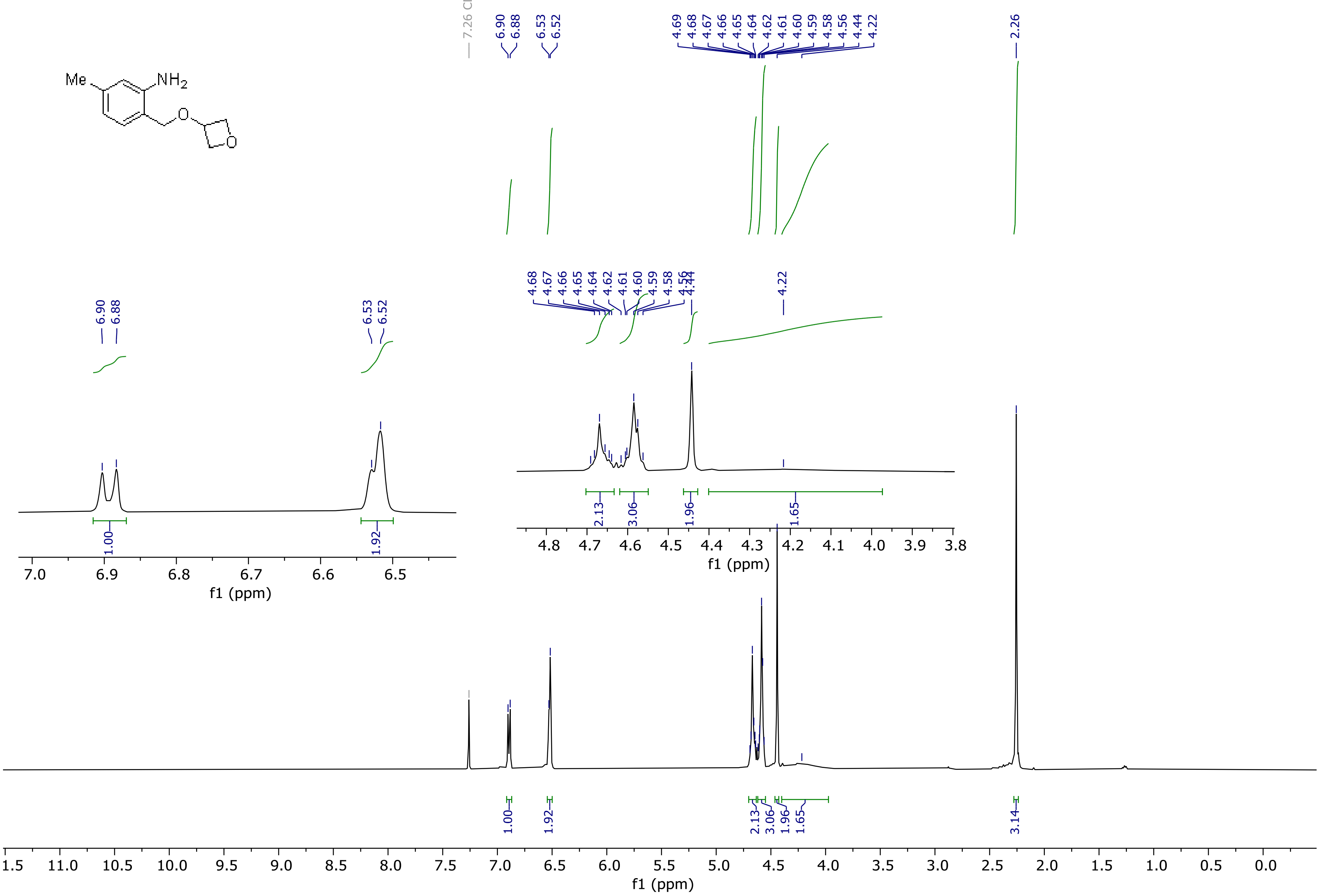

13C{1H}NMR (101 MHz, CDCl3): 5-methyl-2-((oxetan-3-yloxy)methyl)aniline (S13)

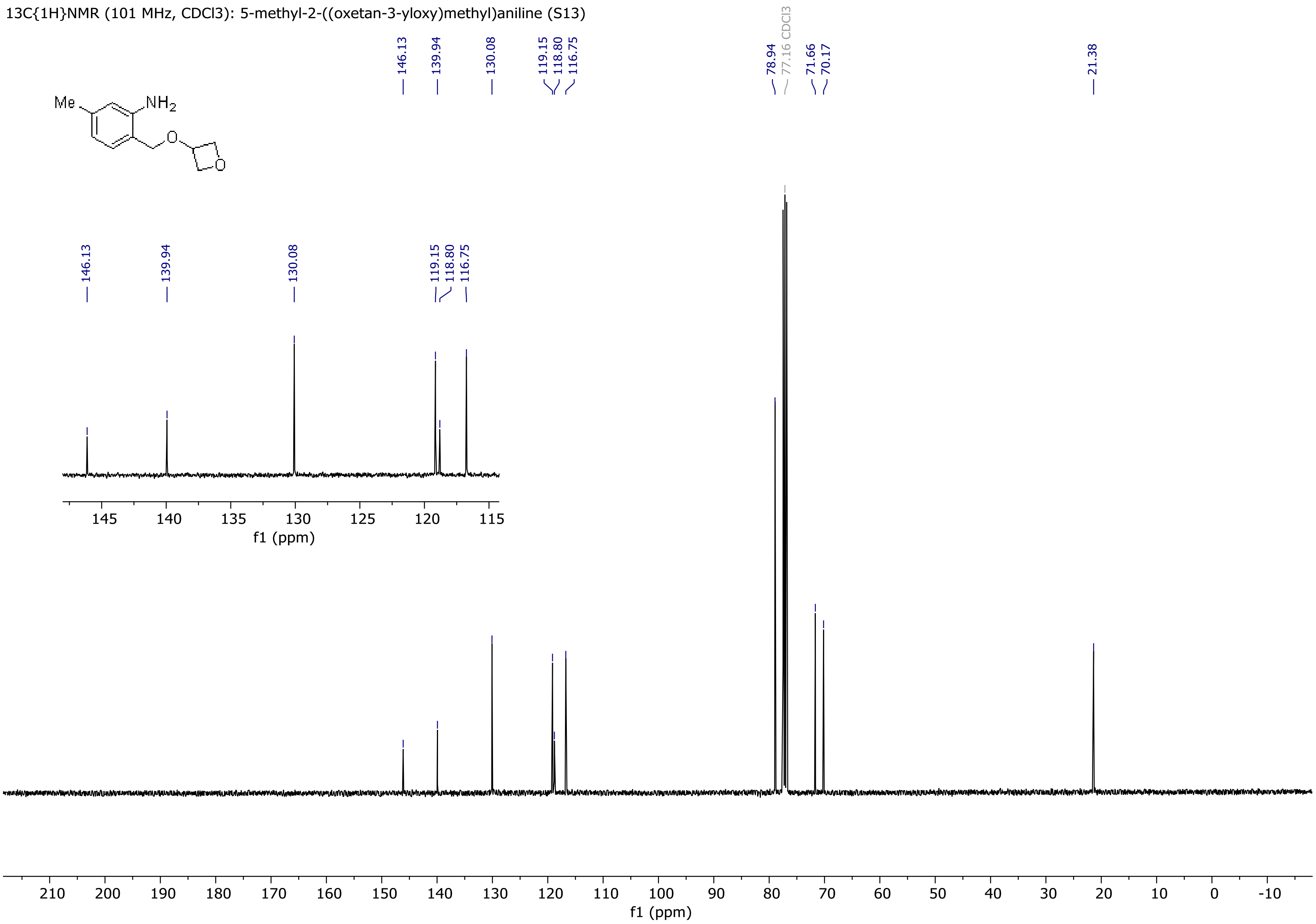

1H NMR (400 MHz, CDCl3): 5-Methoxy-2-((oxetan-3-yloxy)methyl)aniline (S14)

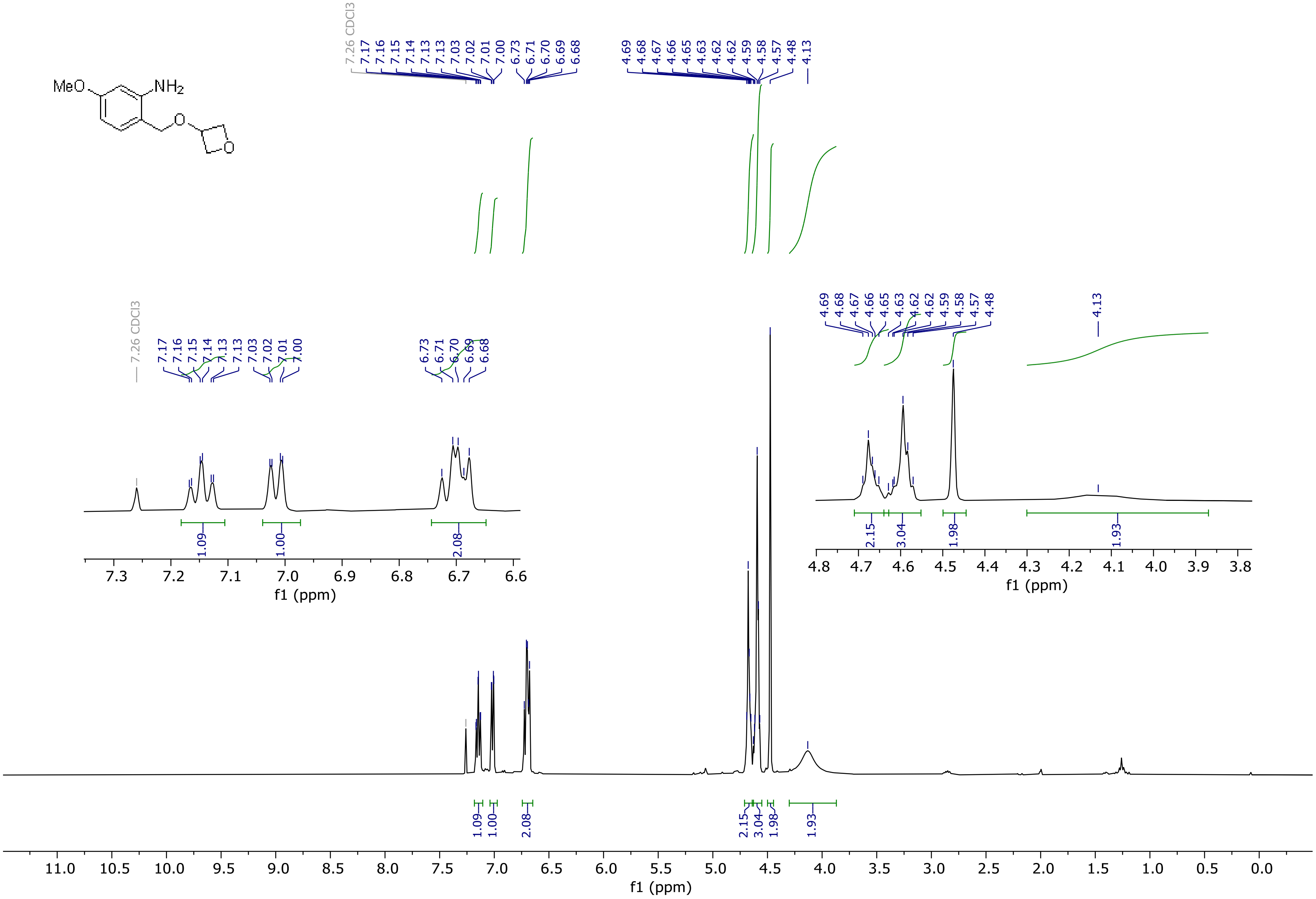

13C{1H}NMR (101 MHz, CDCl3): 5-Methoxy-2-((oxetan-3-yloxy)methyl)aniline (S14)

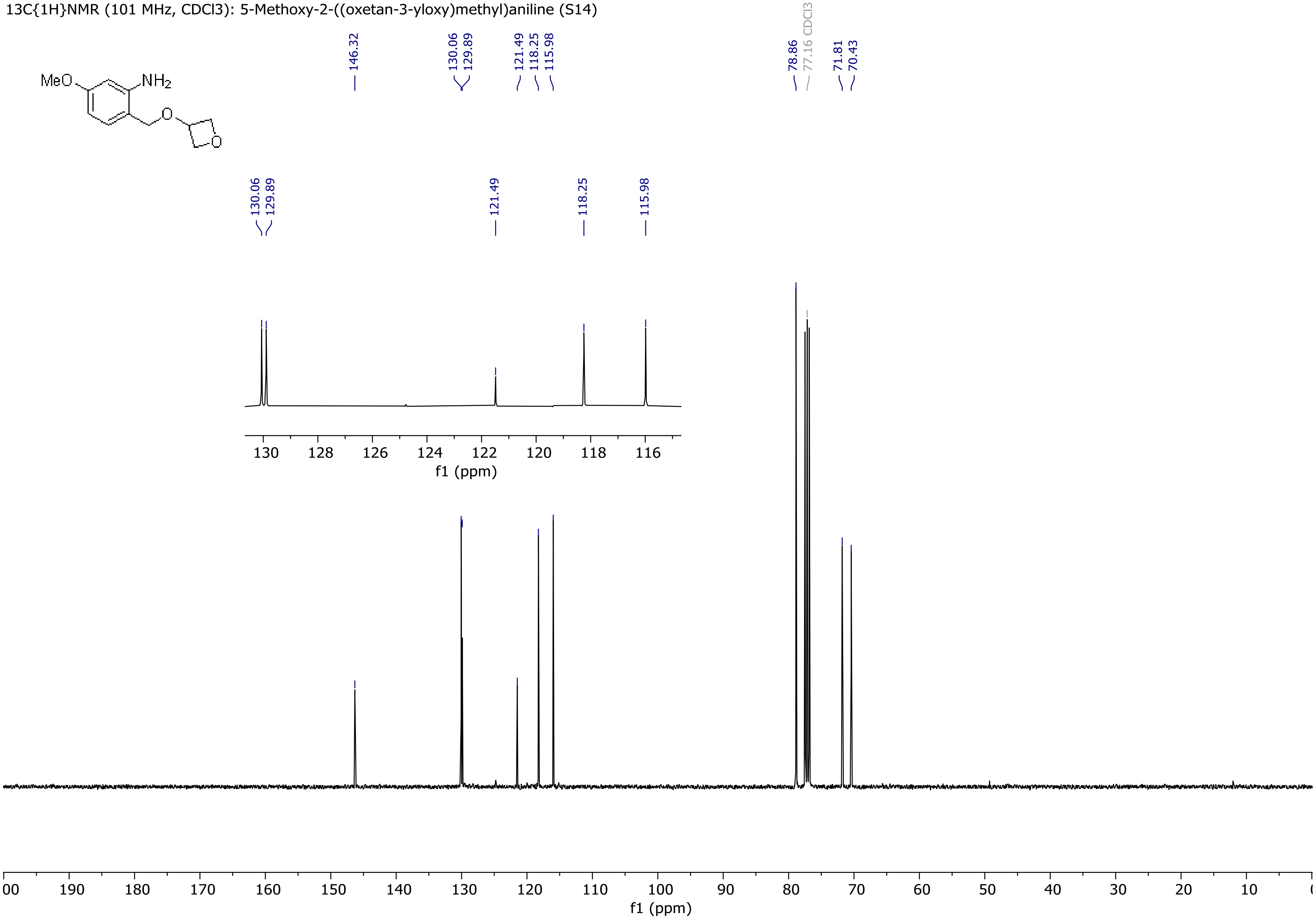

1H NMR: (400 MHz, CDCl3): N-(2-Aminobenzyl)-N-phenyloxetan-3-amine (S15)

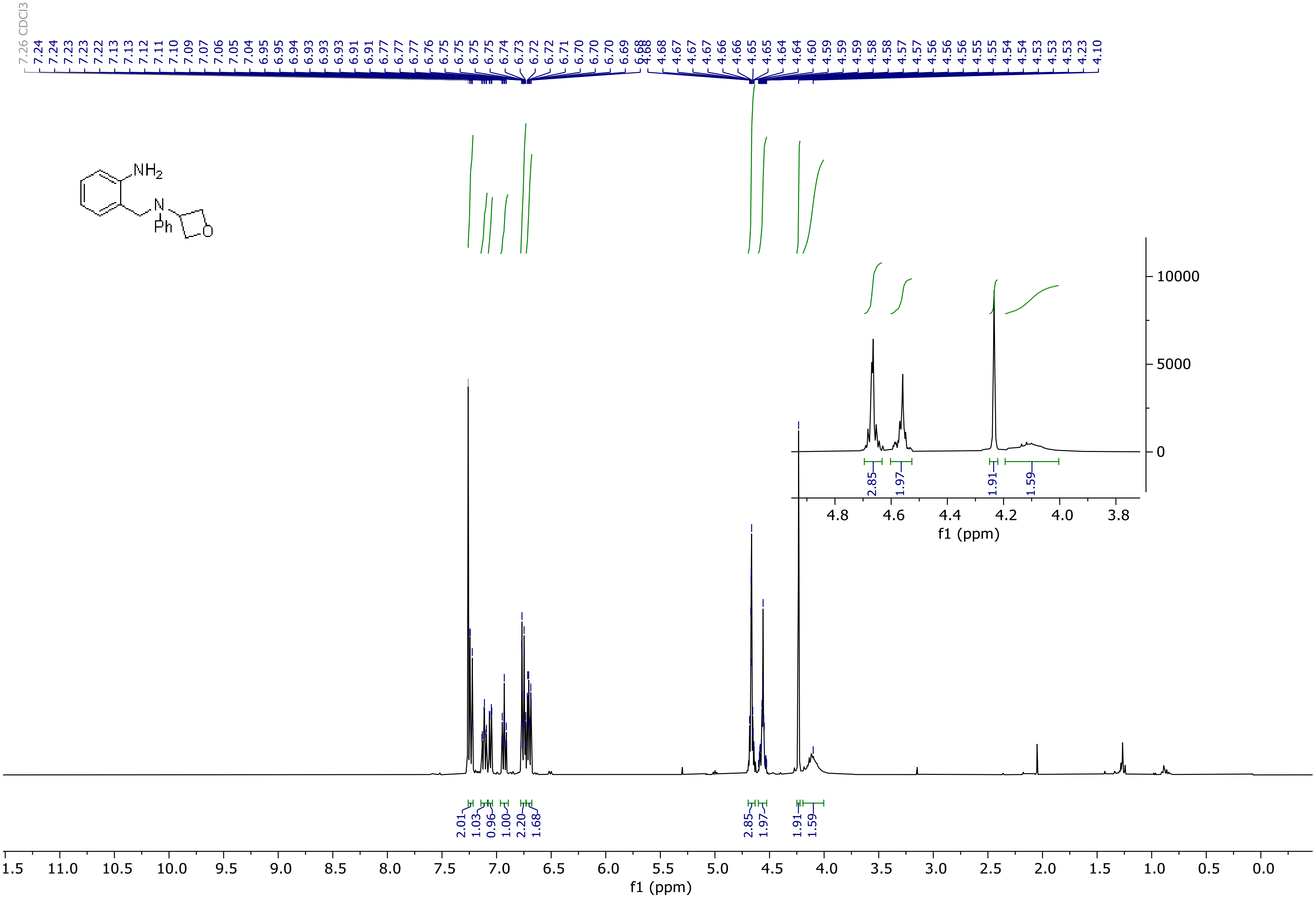

13C{1H}NMR (101 MHz, CDCl3): N-(2-Aminobenzyl)-N-phenyloxetan-3-amine (S15)

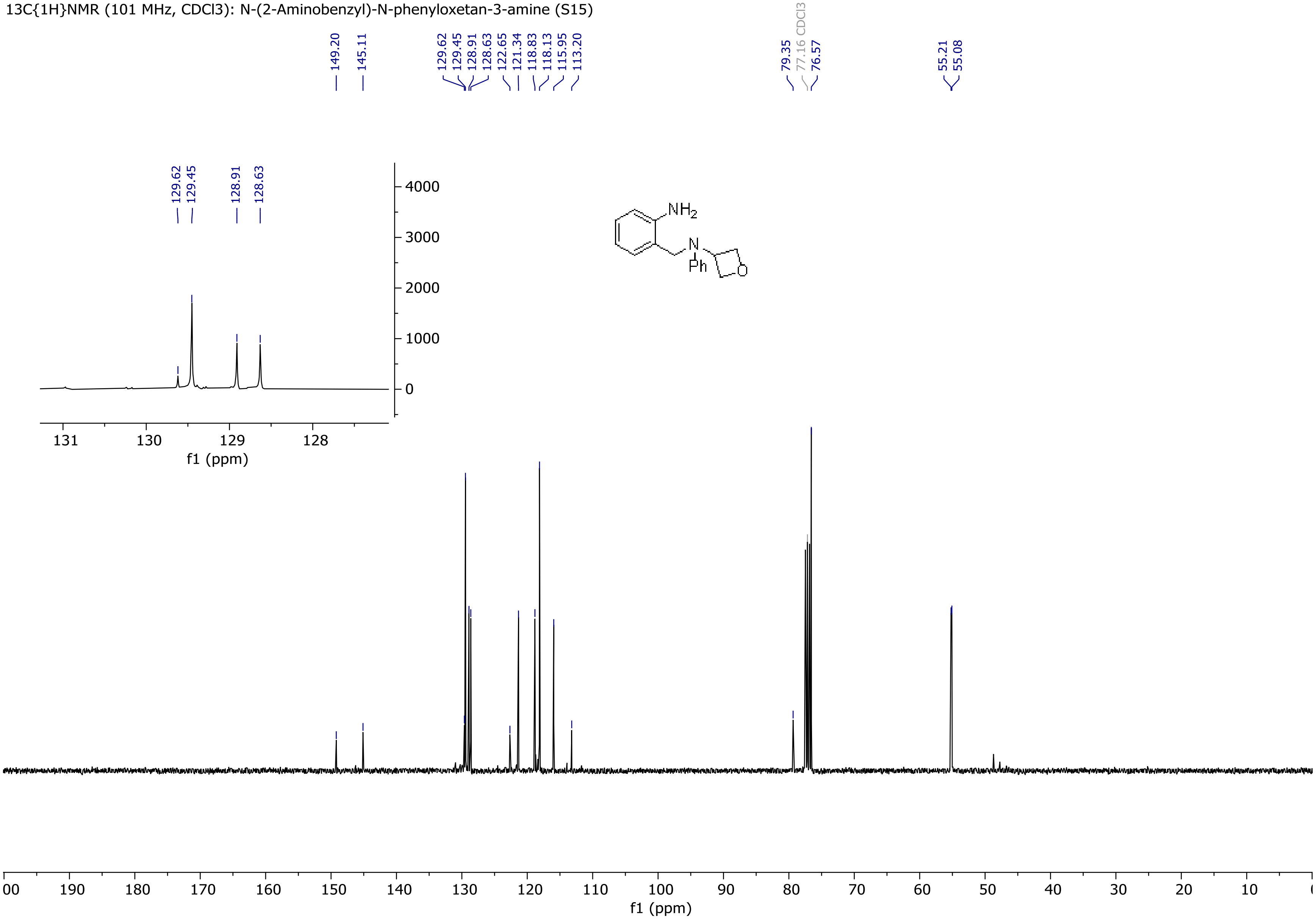

1H NMR: (400 MHz, CDCl3): N-Benzyl-2-((oxetan-3-yloxy)methyl)aniline (1a)

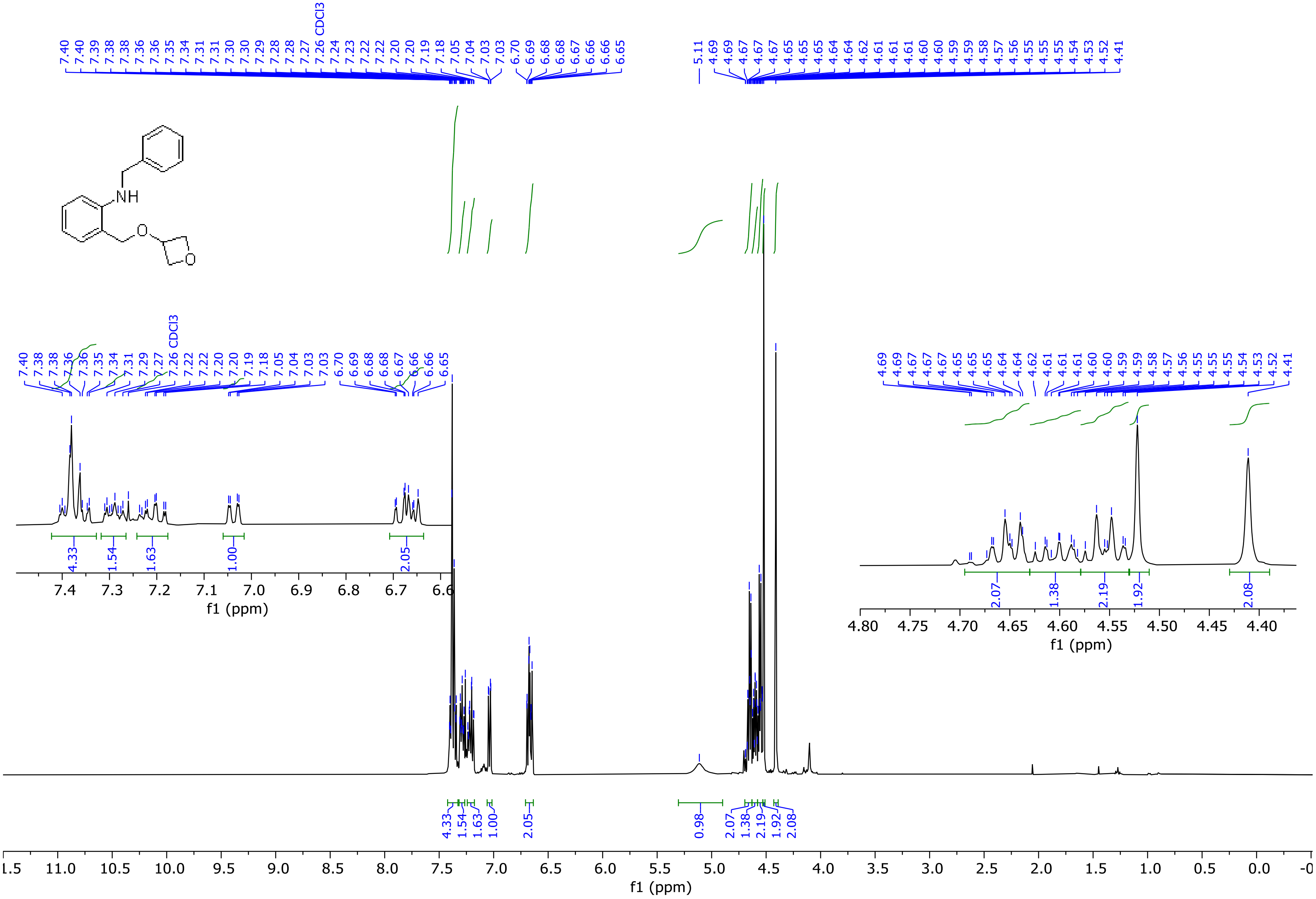

13C{1H}NMR (101 MHz, CDCl3): N-Benzyl-2-((oxetan-3-yloxy)methyl)aniline (1a)

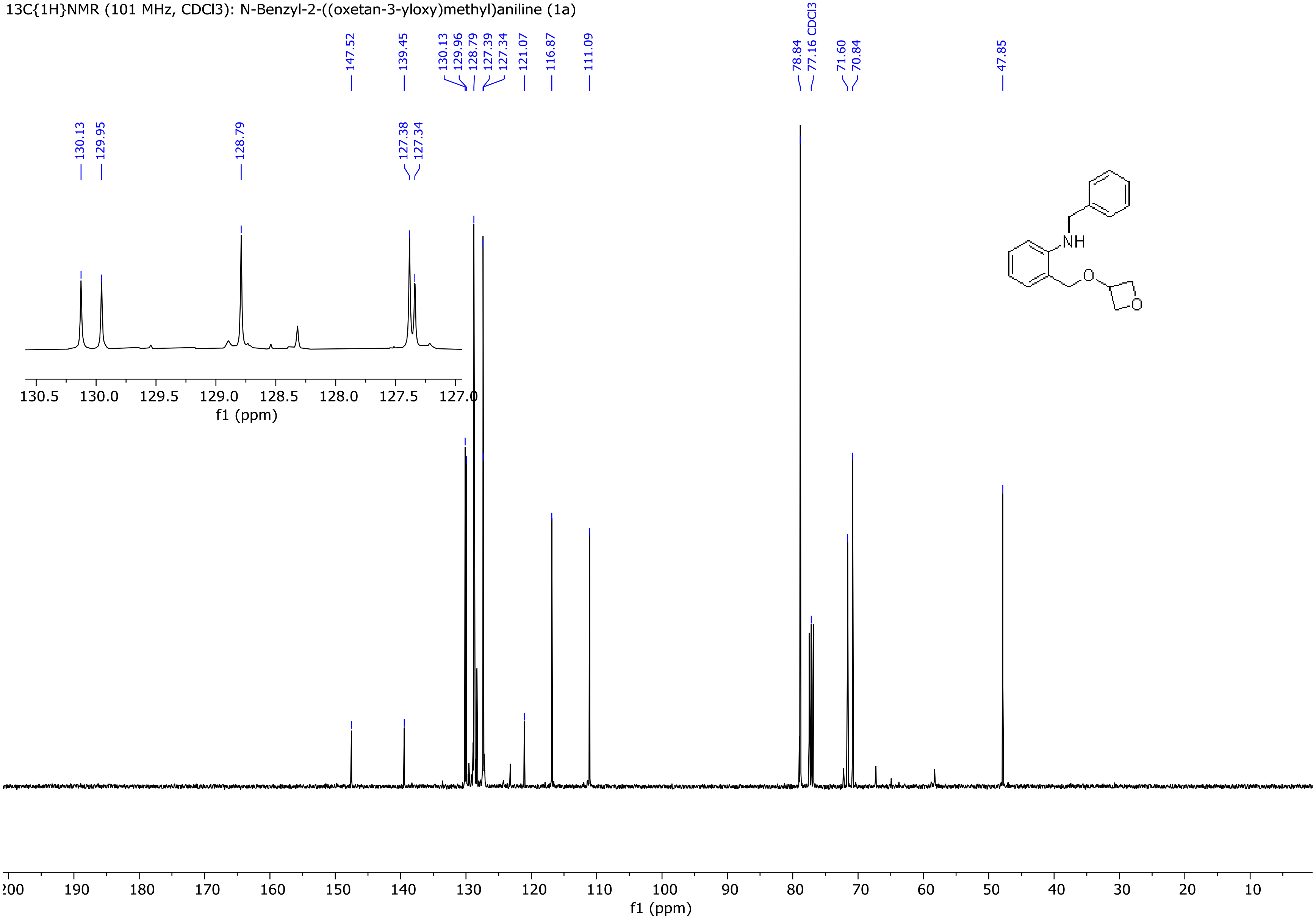

1H NMR: (400 MHz, CDCl3): N-(4-Methoxybenzyl)-2-((oxetan-3-yloxy)methyl)aniline (1b)

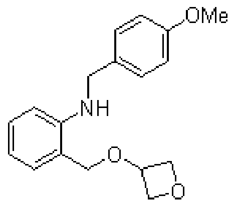

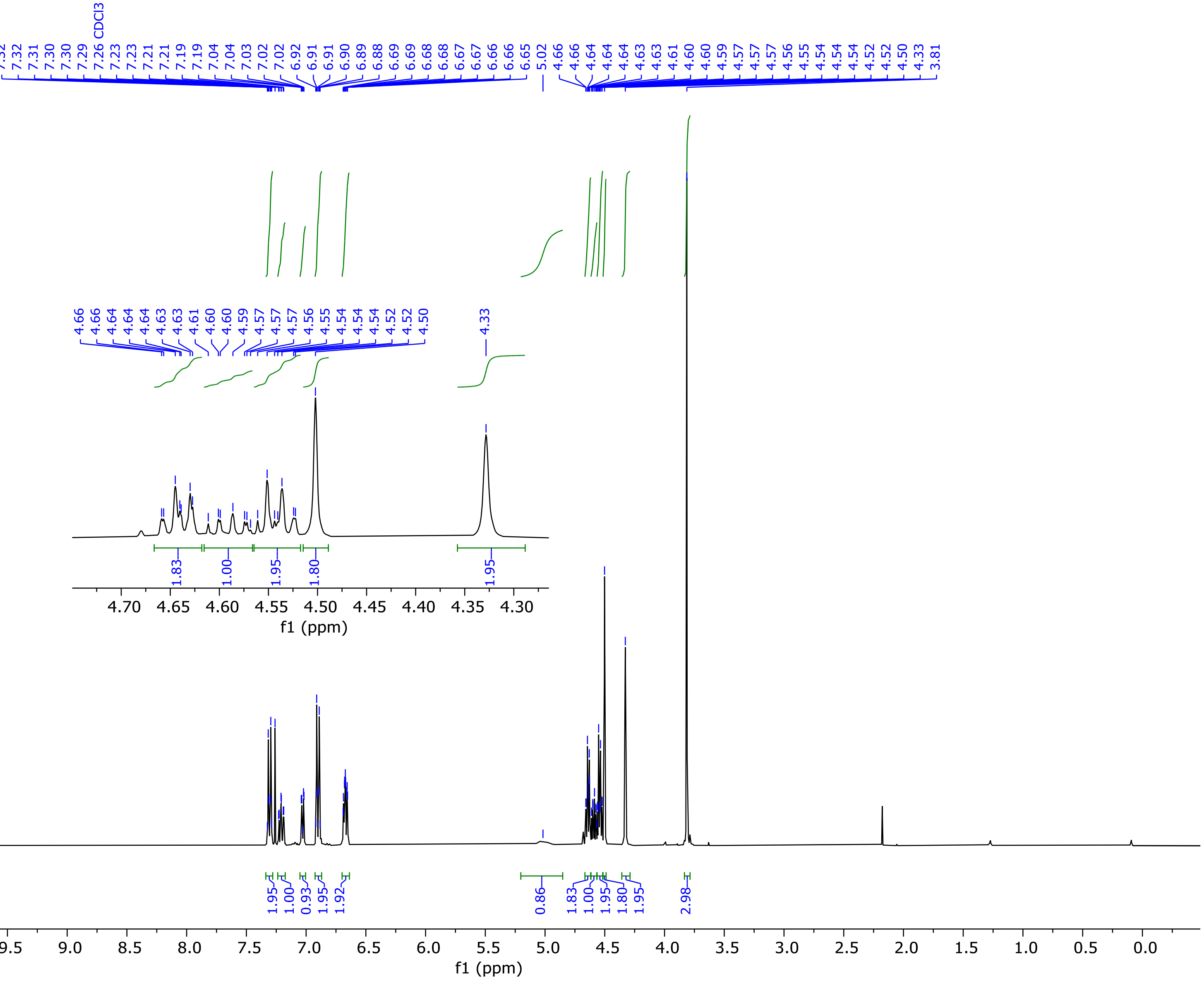

13C{1H}NMR (101 MHz, CDCl3): N-(4-Methoxybenzyl)-2-((oxetan-3-yloxy)methyl)aniline (1b)

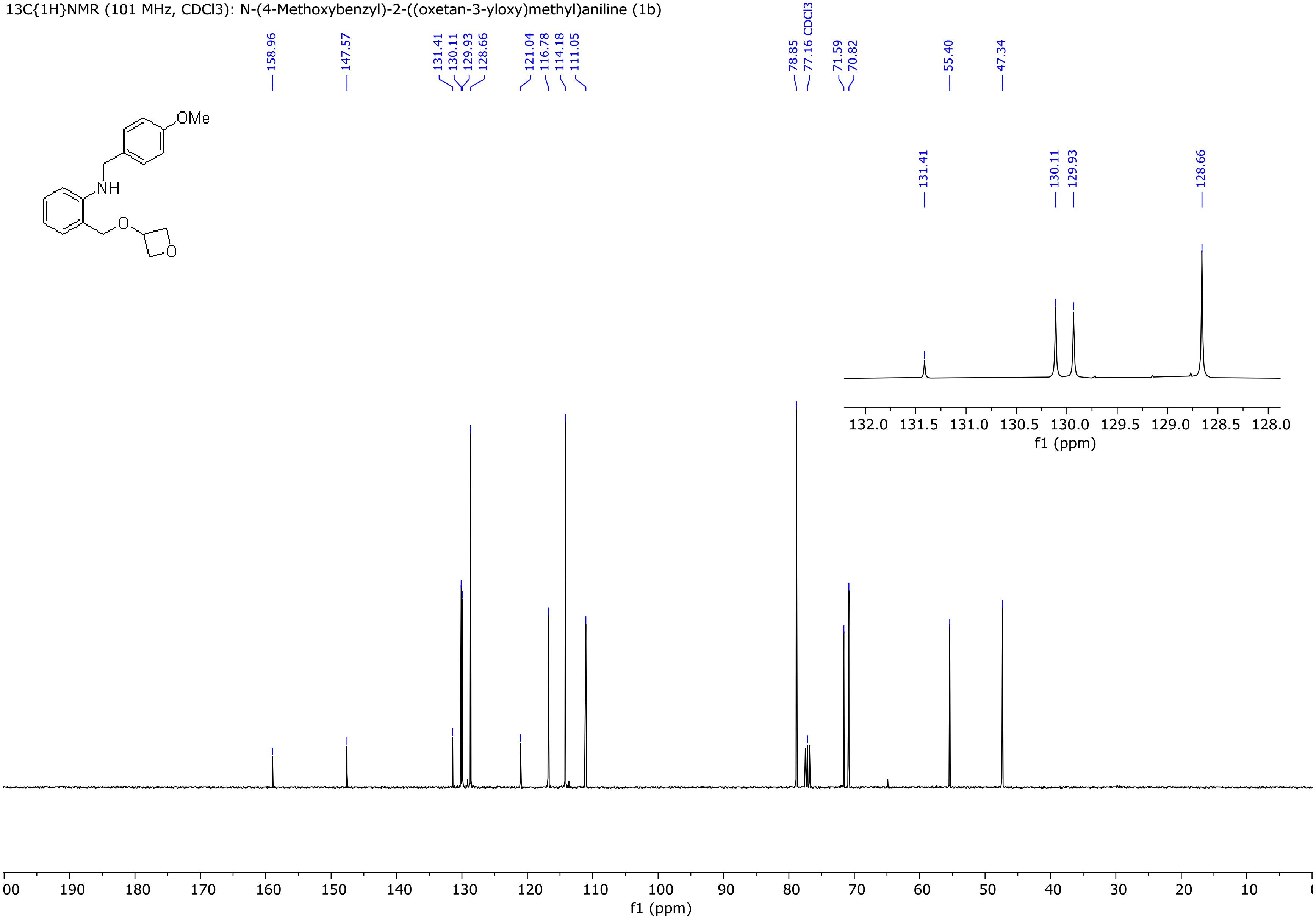

1H NMR: (400 MHz, CDCl3): N-(4-Methylbenzyl)-2-((oxetan-3-yloxy)methyl)aniline (1c)

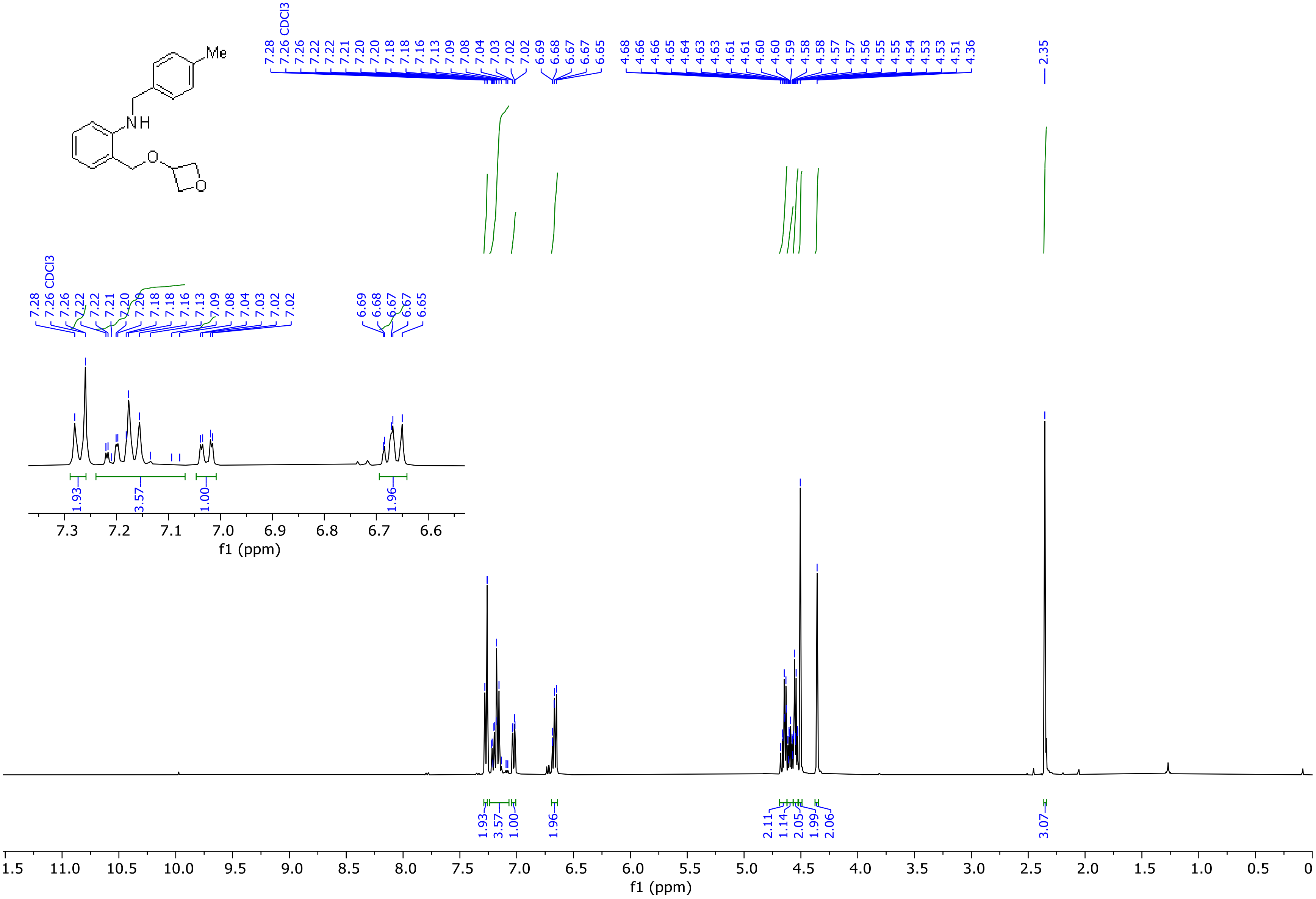

13C{1H}NMR (101 MHz, CDCl3): N-(4-Methylbenzyl)-2-((oxetan-3-yloxy)methyl)aniline (1c)

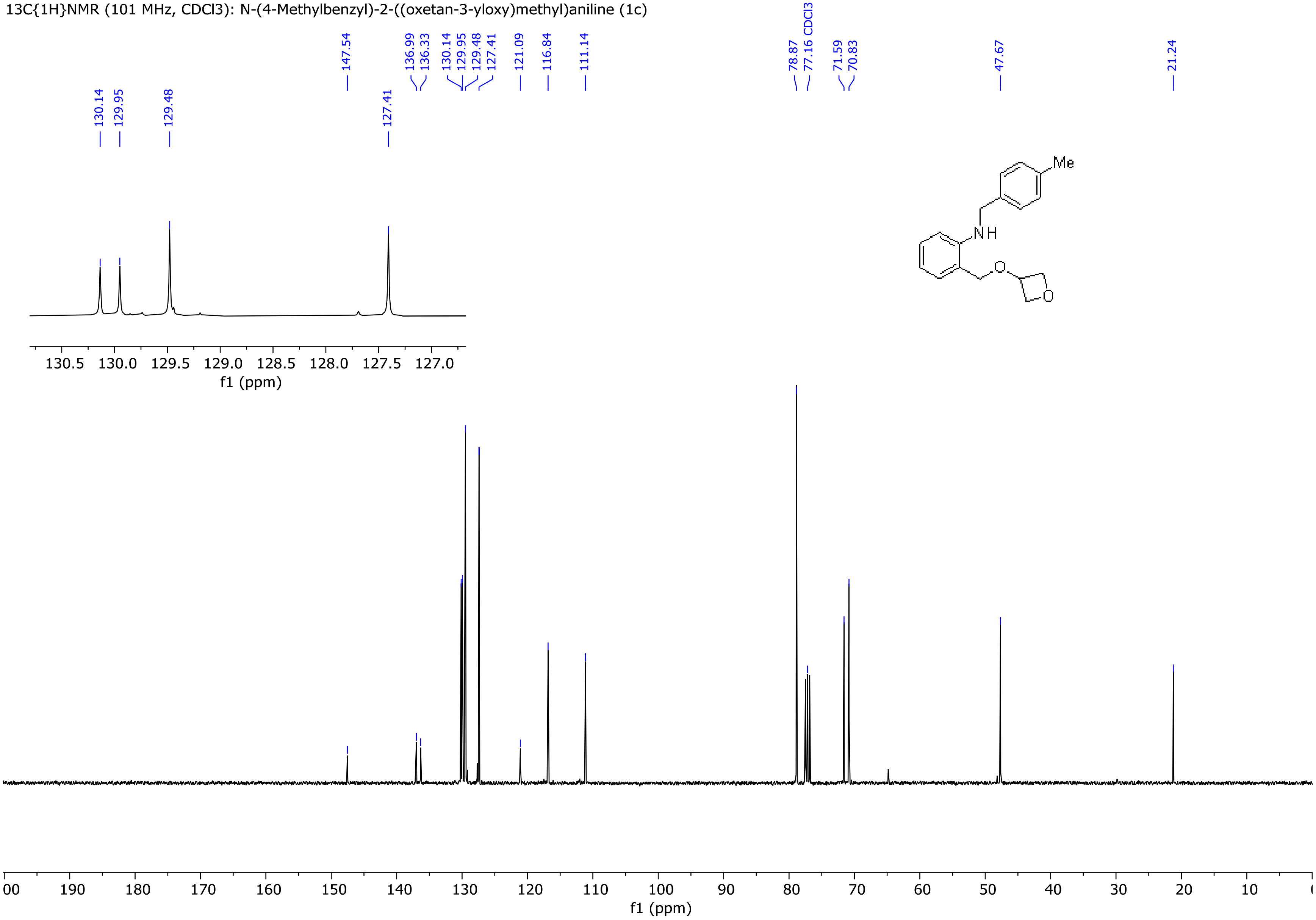

1H NMR: (400 MHz, CDCl3): N-(4-Nitrobenzyl)-2-((oxetan-3-yloxy)methyl)aniline (1d)

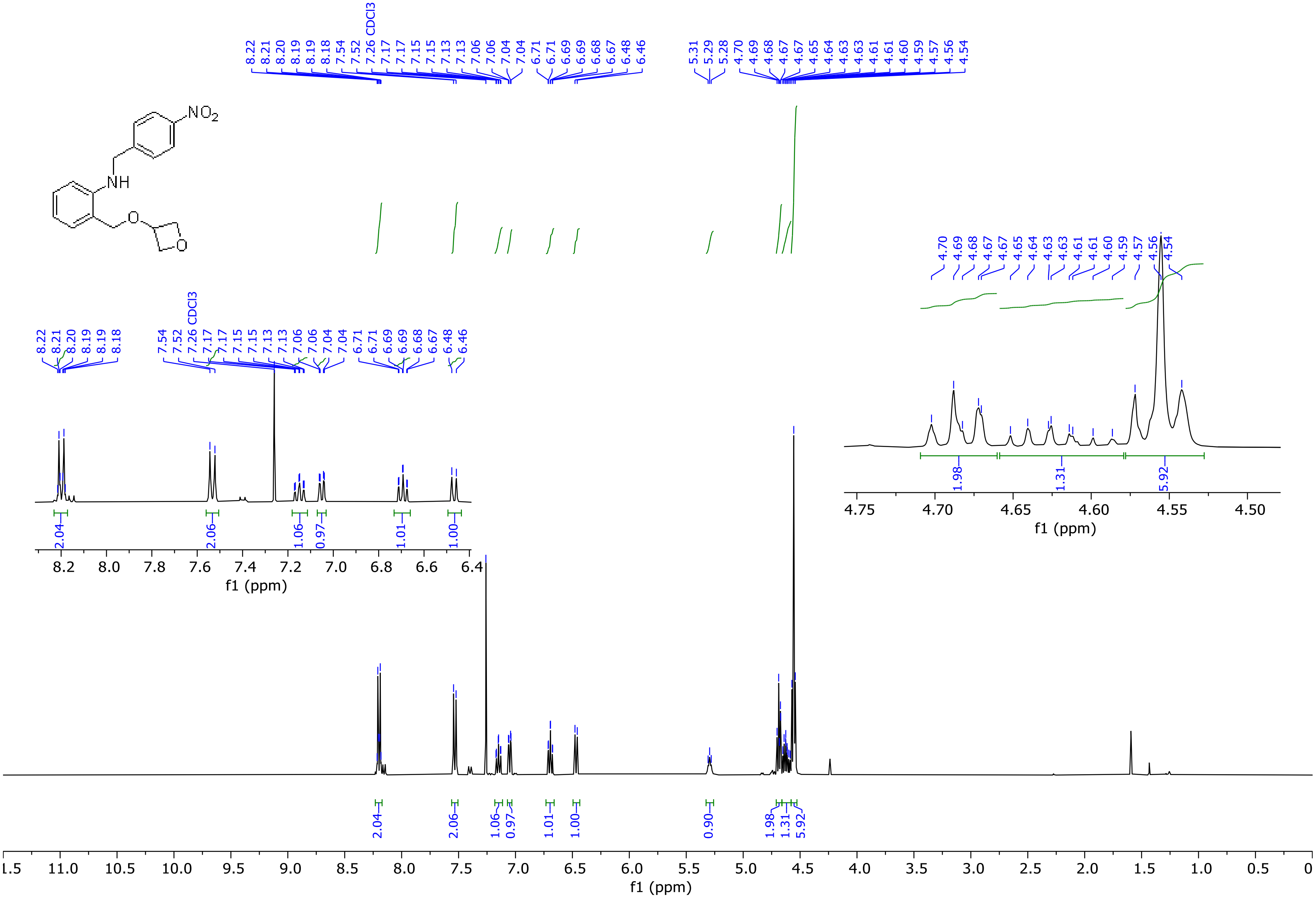

13C{1H}NMR (101 MHz, CDCl3): N-(4-Nitrobenzyl)-2-((oxetan-3-yloxy)methyl)aniline (1d)

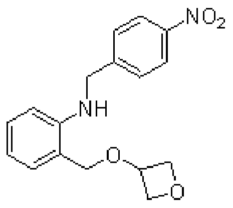

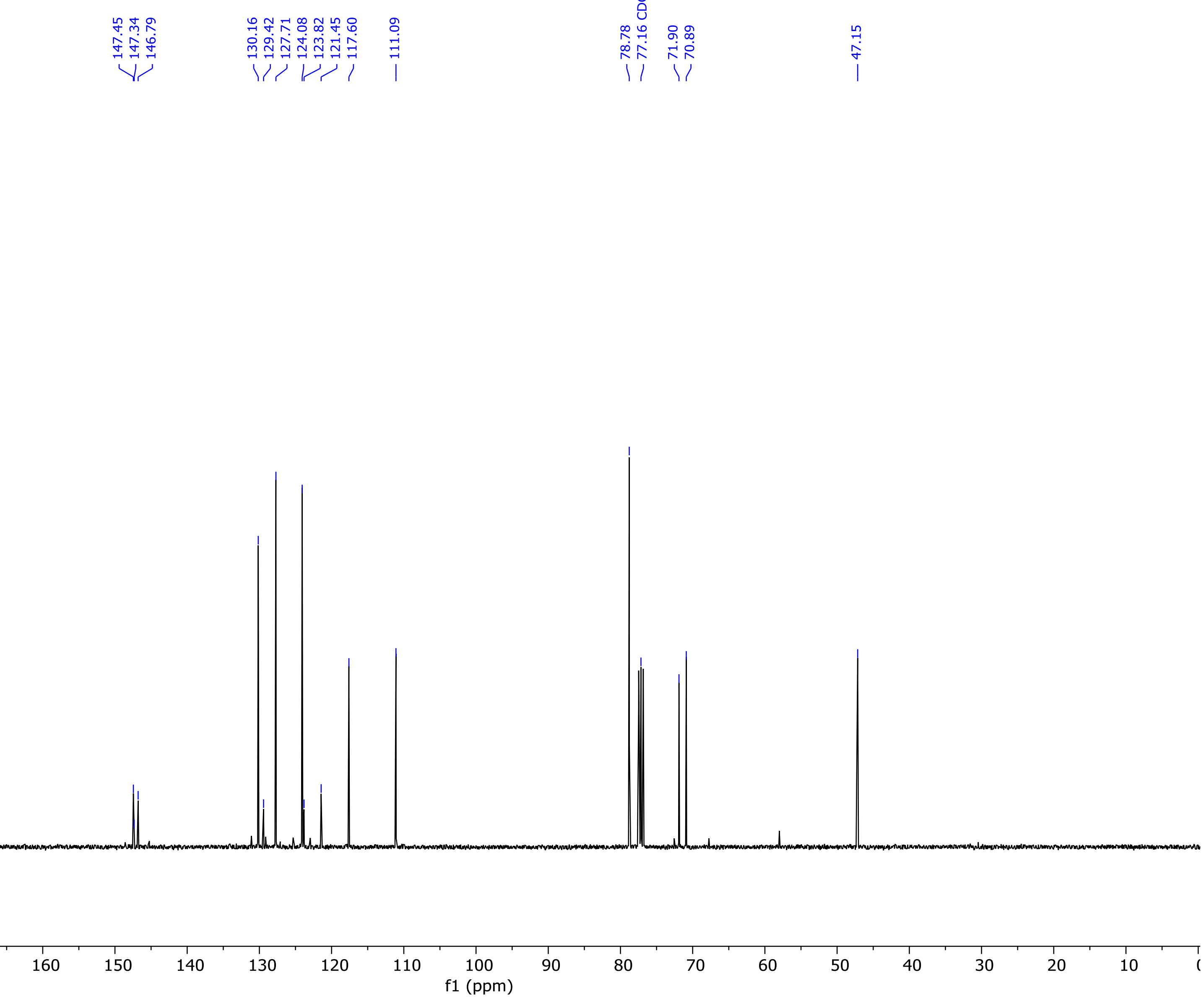

1H NMR: (400 MHz, CDCl3): 4-(((2-((Oxetan-3-yloxy)methyl)phenyl)amino)methyl)benzonitrile (1e)

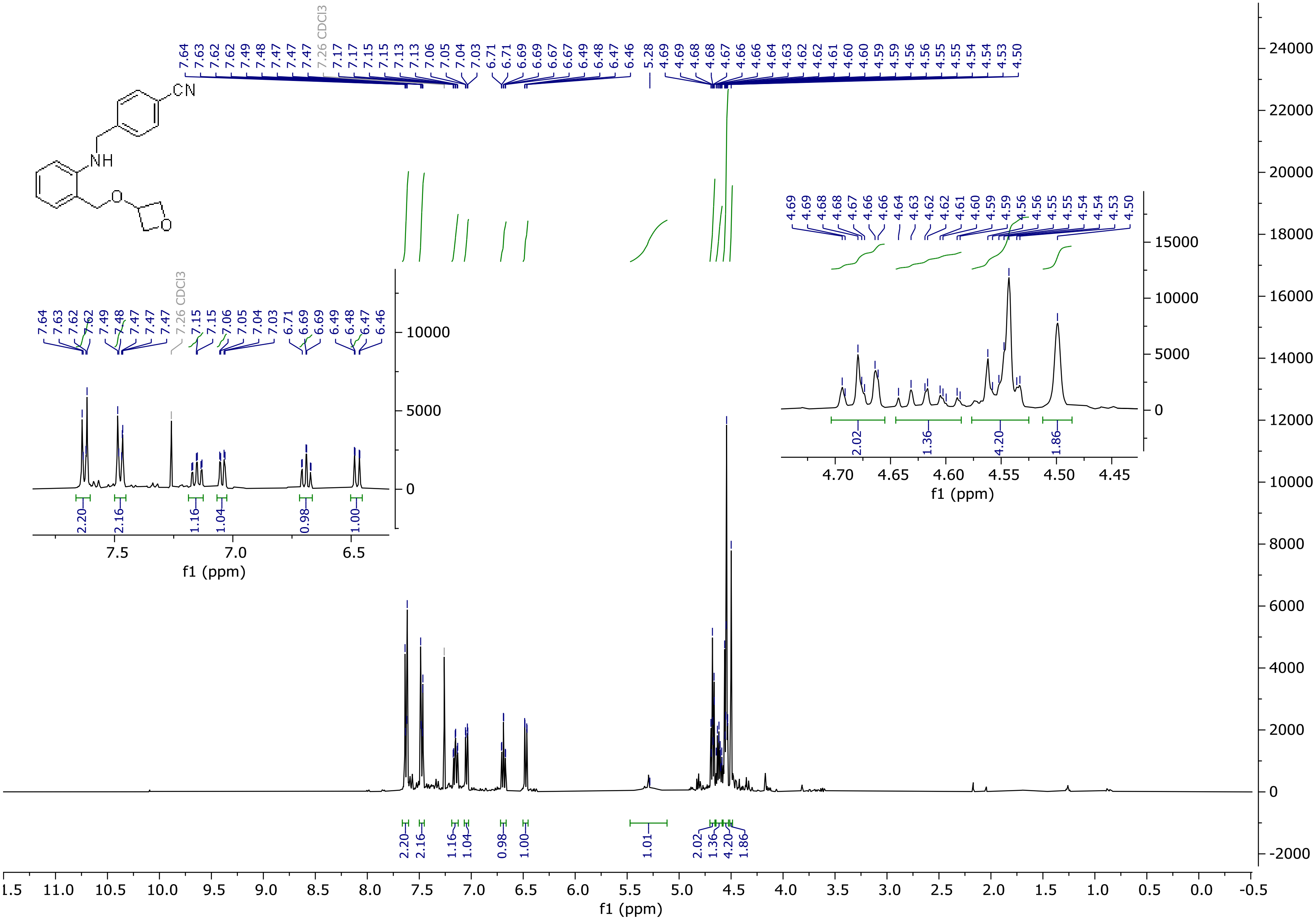

13C{1H}NMR (101 MHz, CDCl3): 4-(((2-((Oxetan-3-yloxy)methyl)phenyl)amino)methyl)benzonitrile (1e)

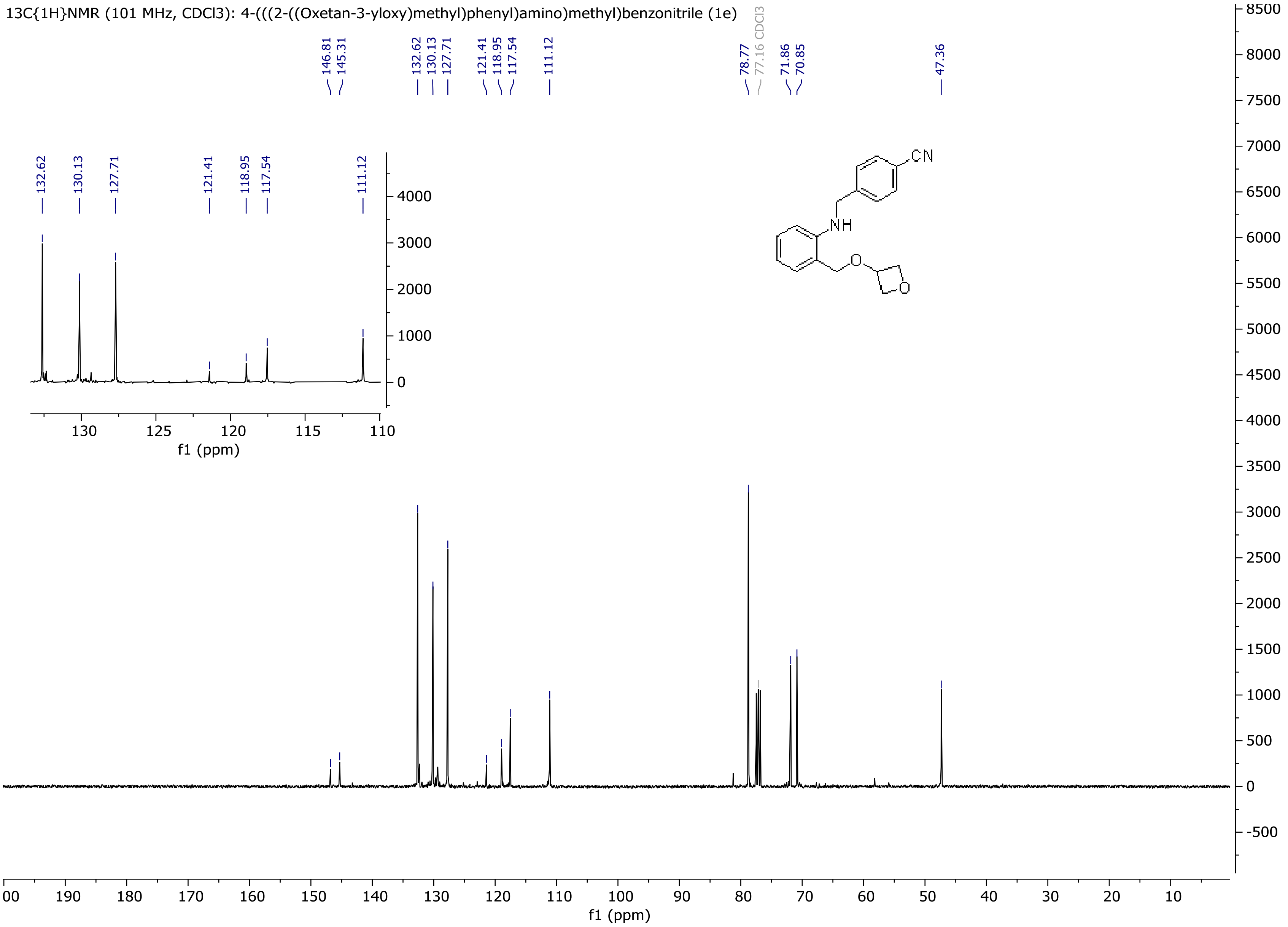

1H NMR: (400 MHz, CDCl3): 2-((Oxetan-3-yloxy)methyl)-N-(4-(trifluoromethyl)benzyl)aniline (1f)

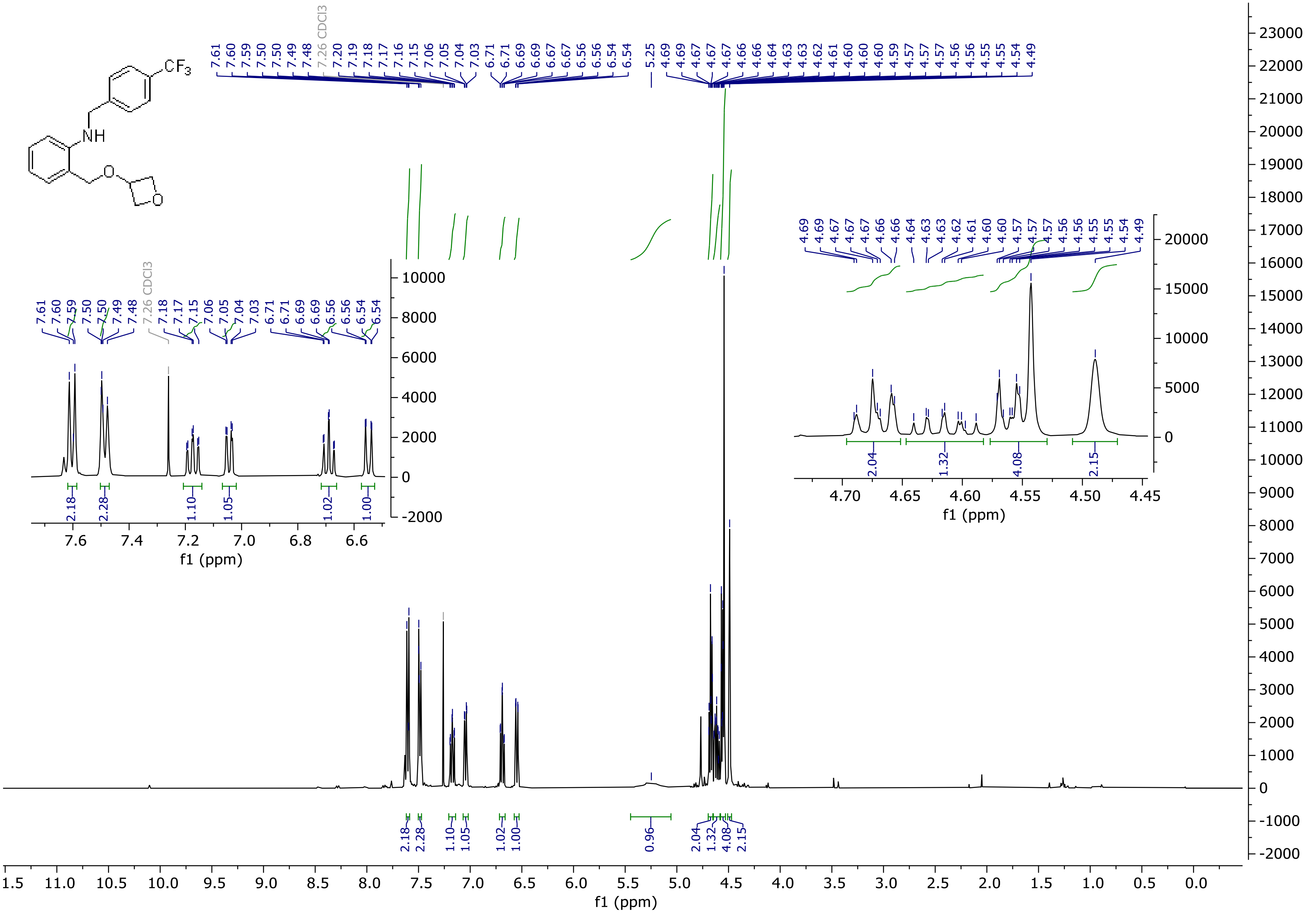

13C{1H}NMR (101 MHz, CDCl3): 2-((Oxetan-3-yloxy)methyl)-N-(4-(trifluoromethyl)benzyl)aniline (1f)

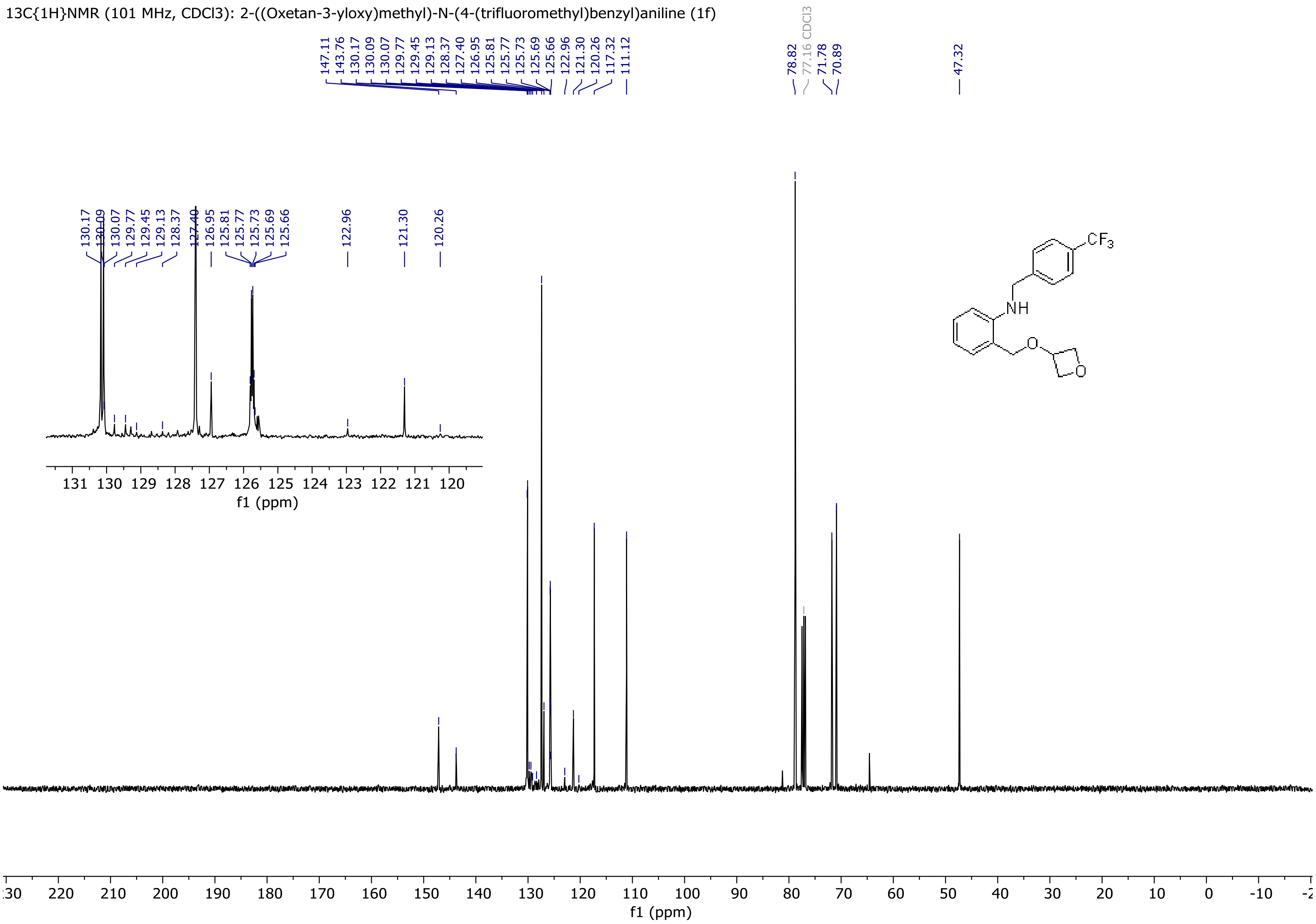

19F NMR (376 MHz, CDCl3): 2-((Oxetan-3-yloxy)methyl)-N-(4-(trifluoromethyl)benzyl)aniline (1f)

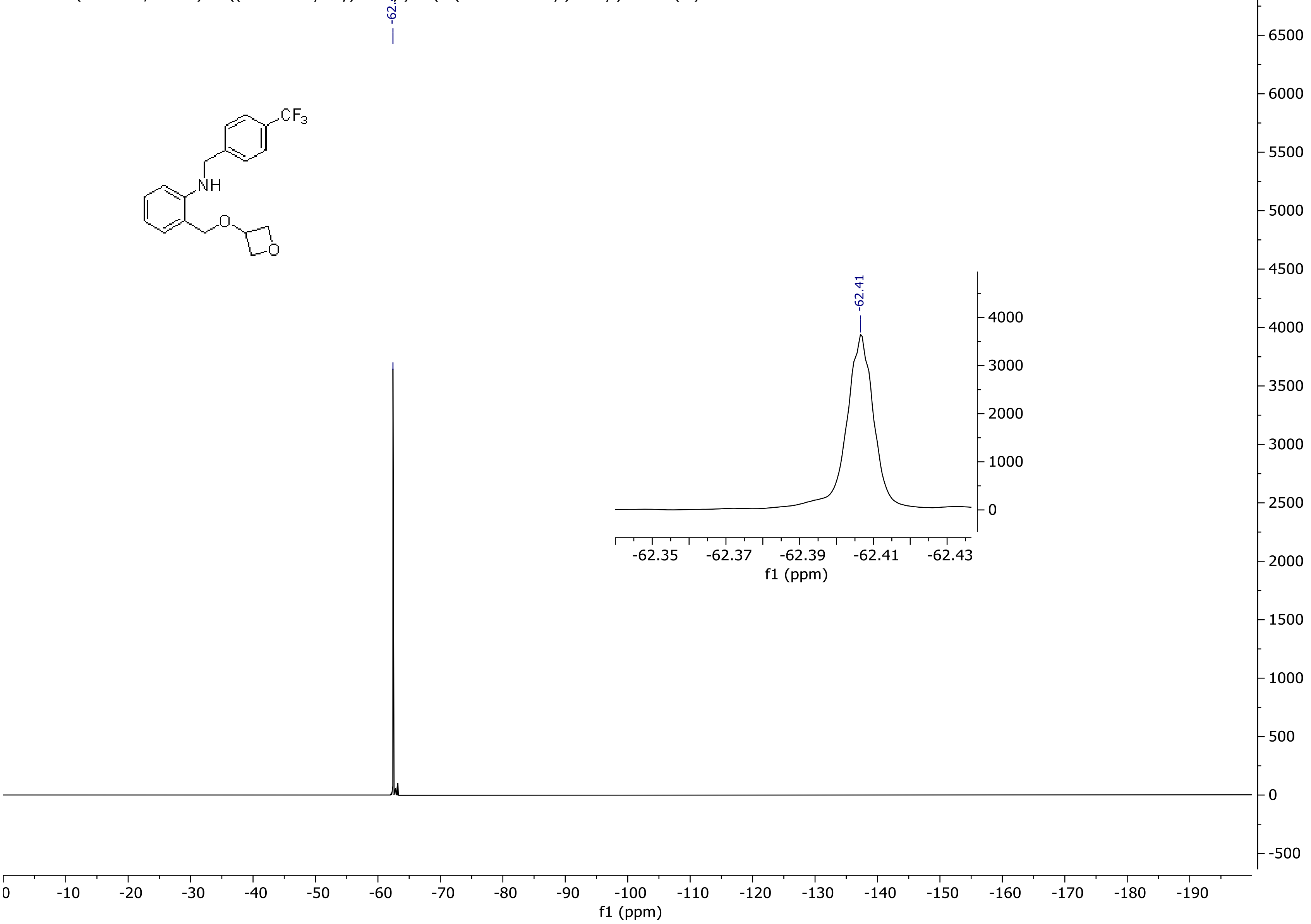

1H NMR: (400 MHz, CDCl3): N-(4-Fluorobenzyl)-2-((oxetan-3-yloxy)methyl)aniline (1g)

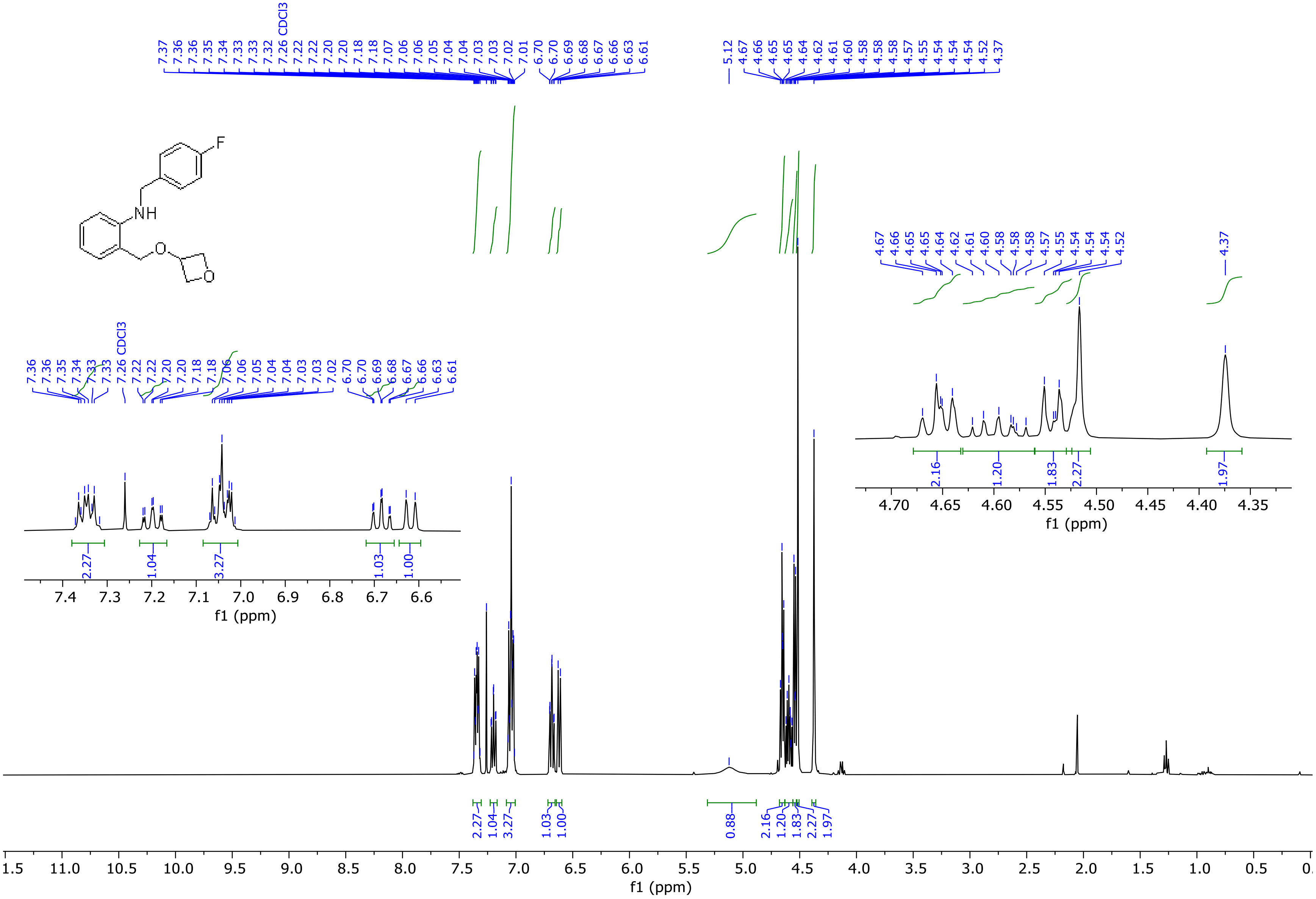

13C{1H}NMR (101 MHz, CDCl3): N-(4-Fluorobenzyl)-2-((oxetan-3-yloxy)methyl)aniline (1g)

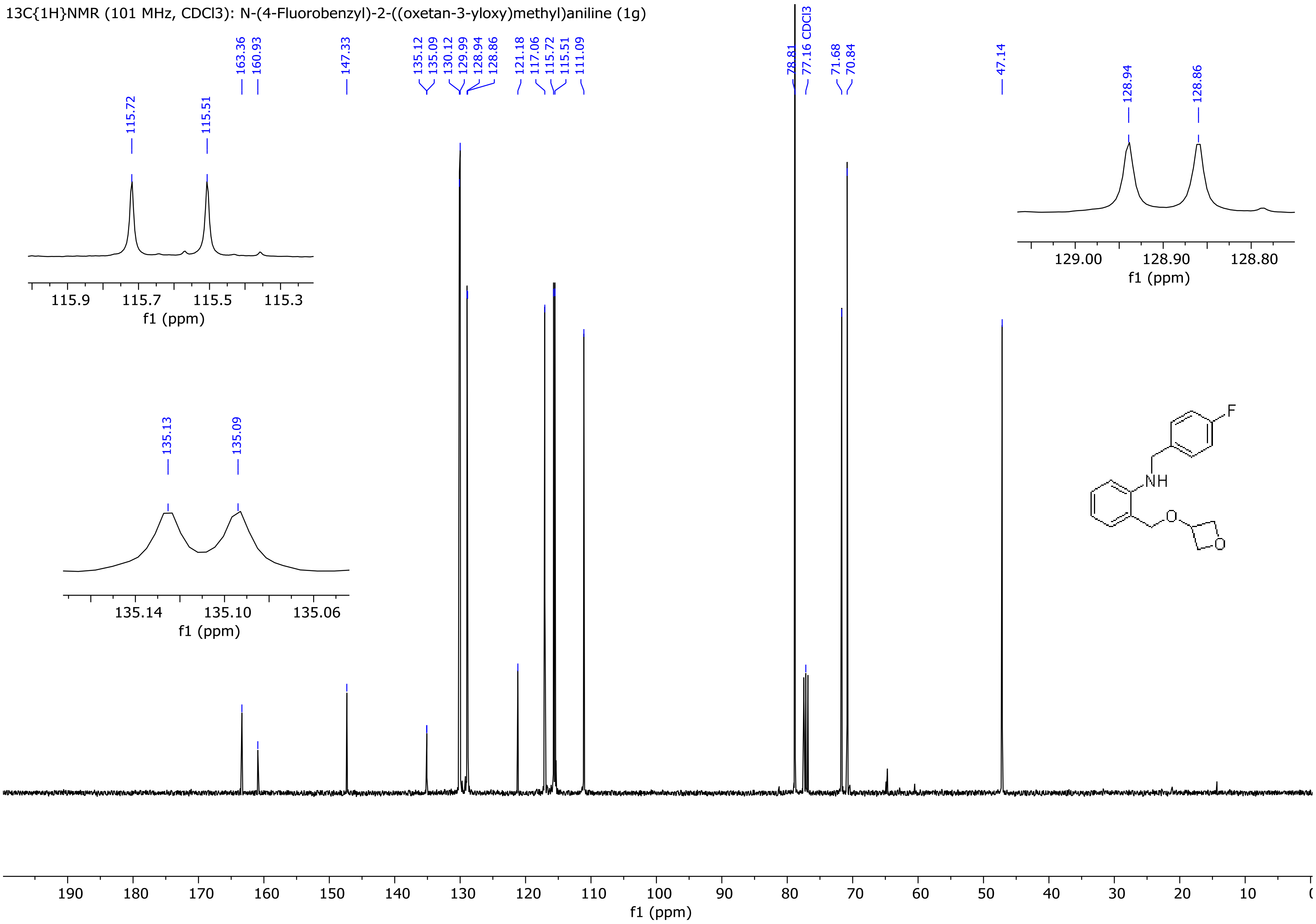

19F NMR (376 MHz, CDCl3): N-(4-Fluorobenzyl)-2-((oxetan-3-yloxy)methyl)aniline (1g)

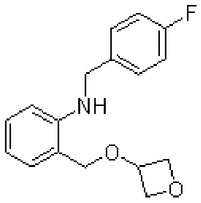

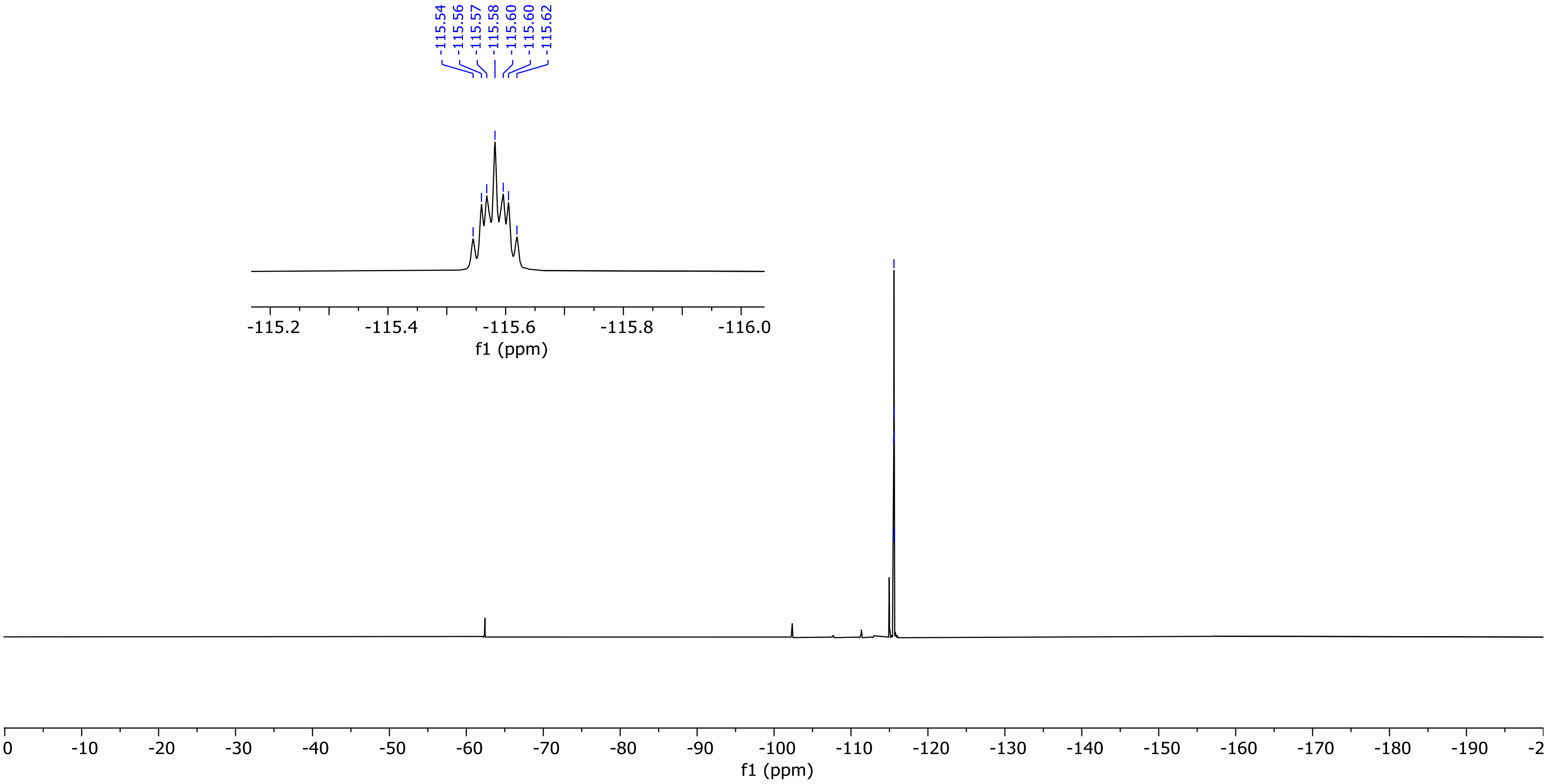

1H NMR: (400 MHz, CDCl3): N-(4-Bromobenzyl)-2-((oxetan-3-yloxy)methyl)aniline (1h)

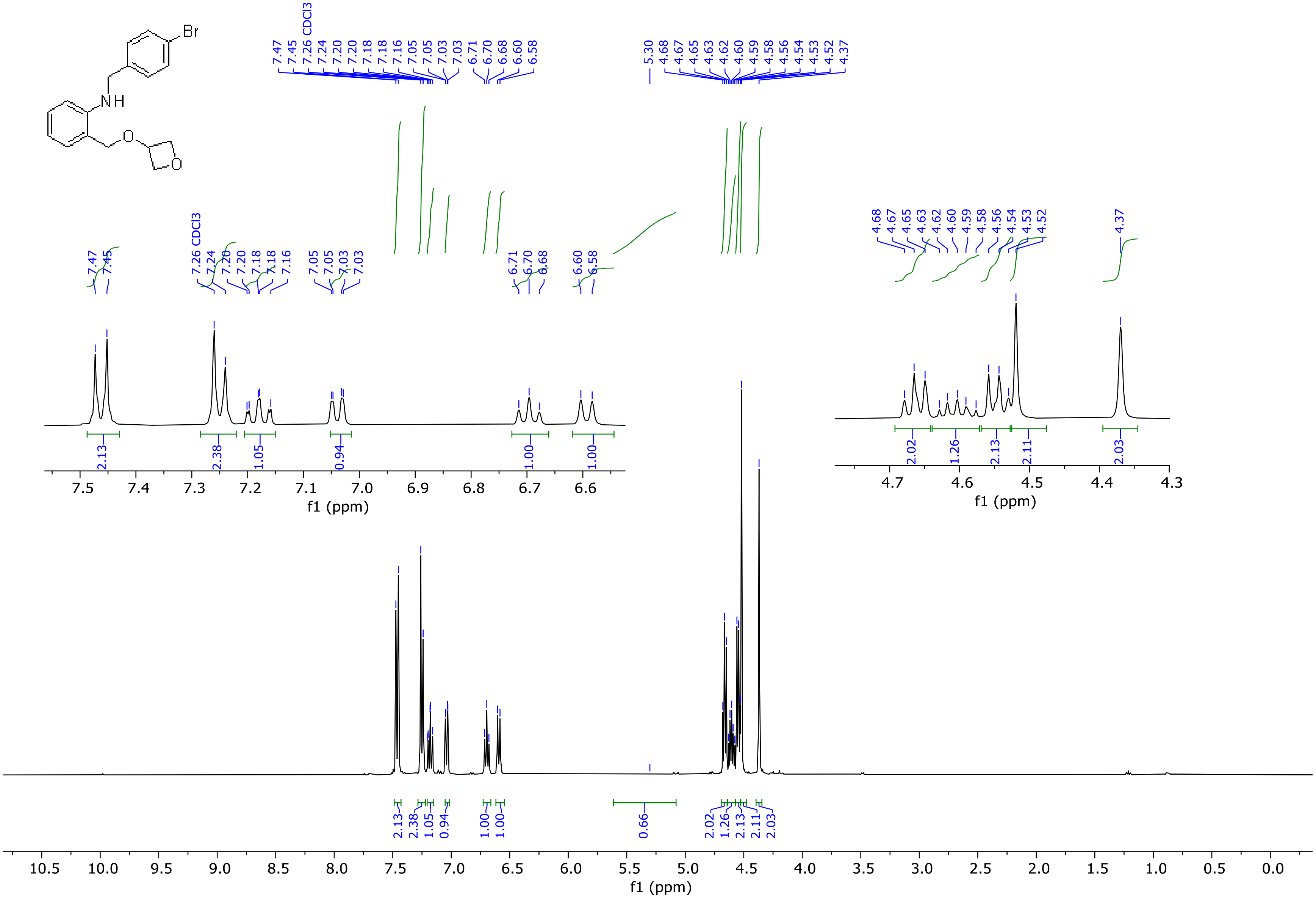

13C{1H}NMR (101 MHz, CDCl3): N-(4-Bromobenzyl)-2-((oxetan-3-yloxy)methyl)aniline (1h)

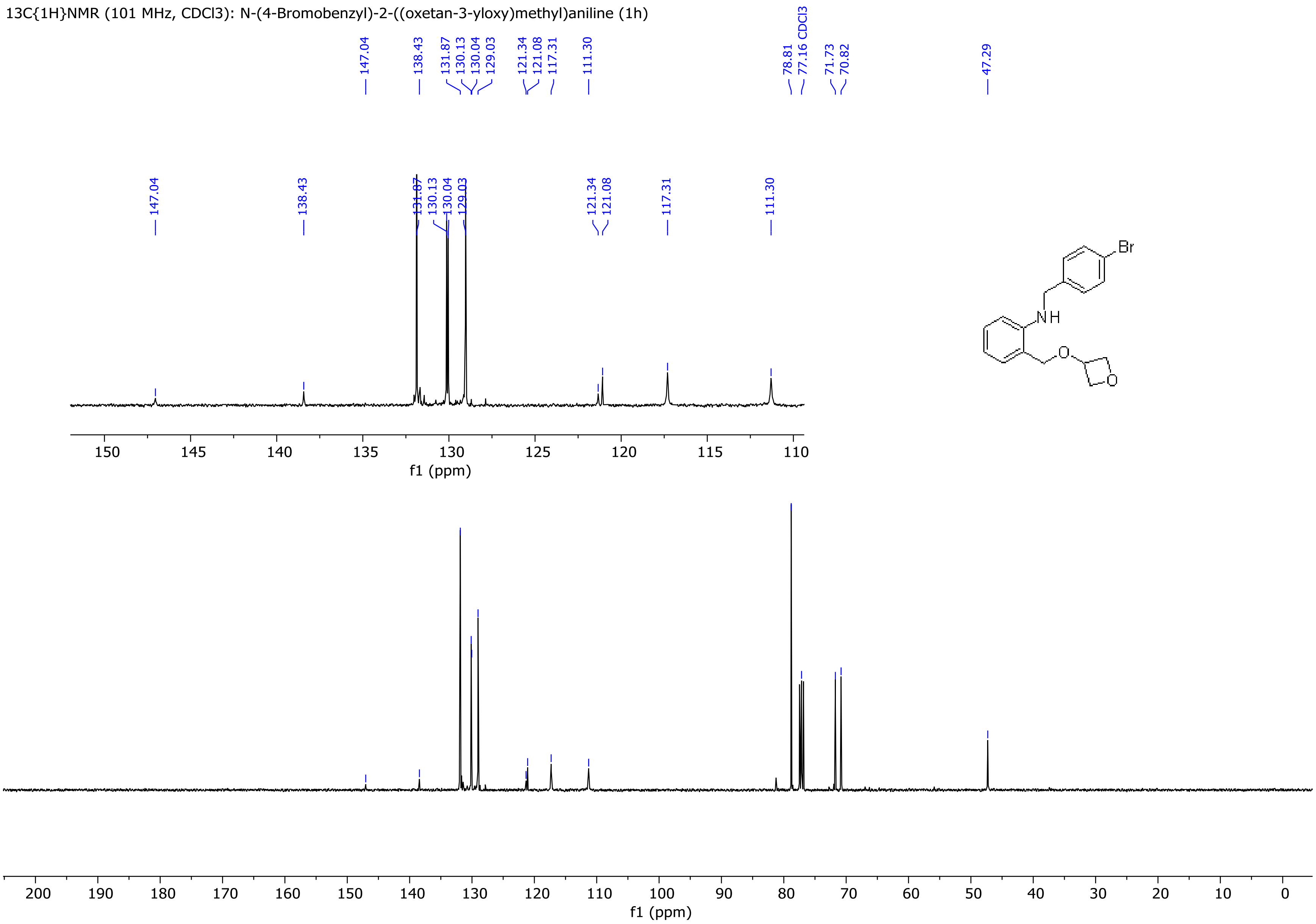

1H NMR: (400 MHz, CDCl3): N-(3-Bromobenzyl)-2-((oxetan-3-yloxy)methyl)aniline (1i)

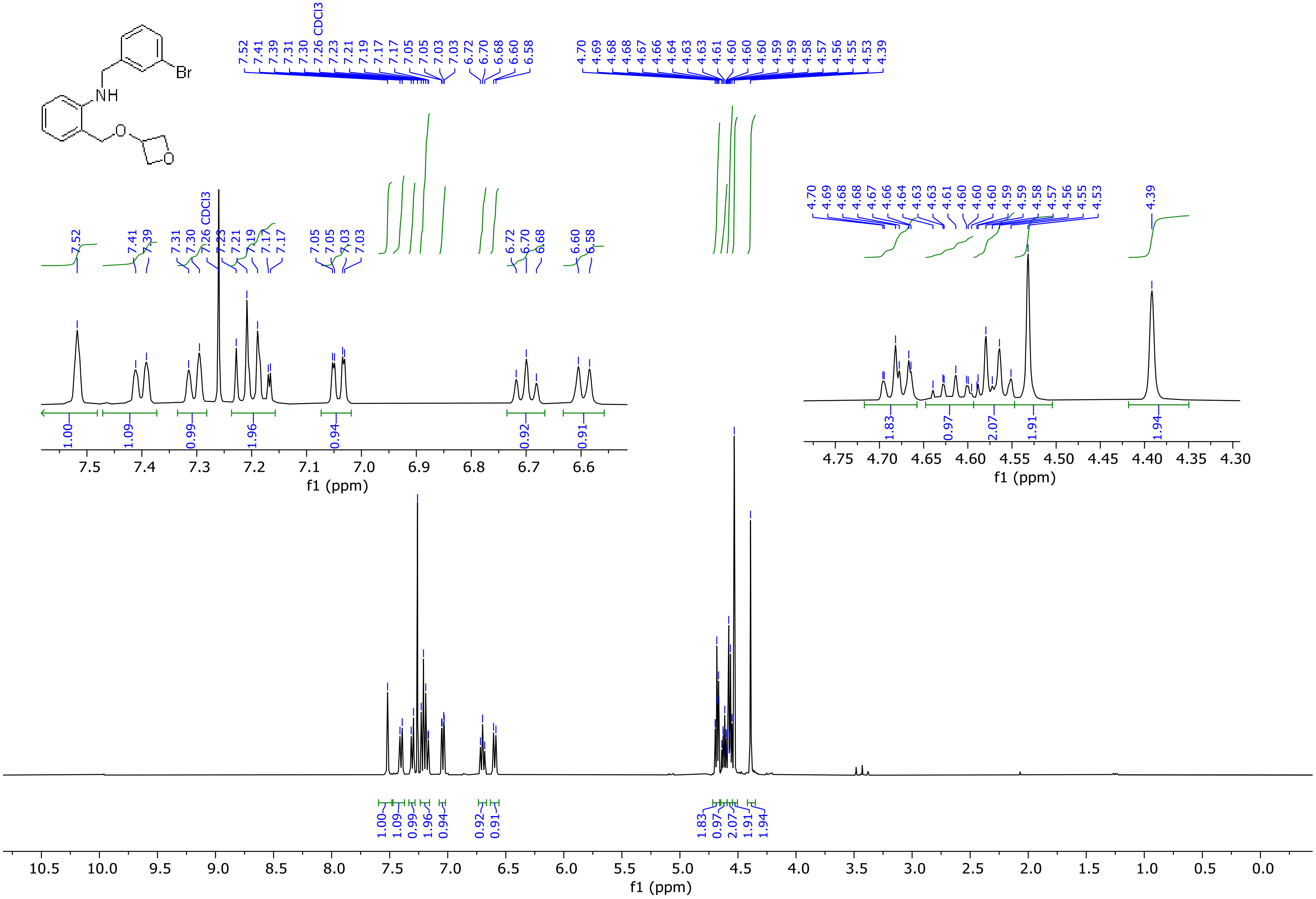

13C{1H}NMR (101 MHz, CDCl3): N-(3-Bromobenzyl)-2-((oxetan-3-yloxy)methyl)aniline (1i)

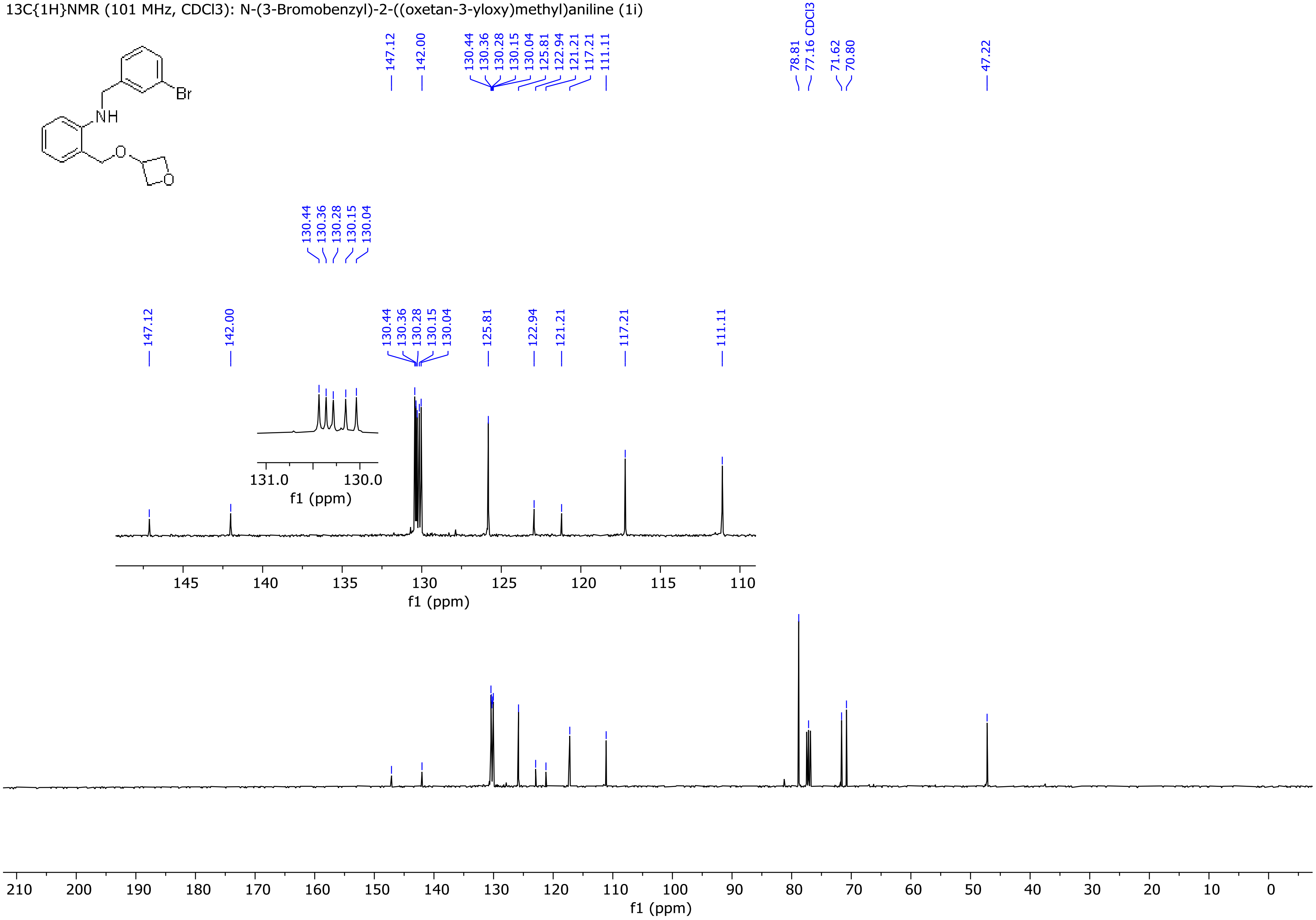

1H NMR: (400 MHz, CDCl3): N-(2-Bromobenzyl)-2-((oxetan-3-yloxy)methyl)aniline (1j)

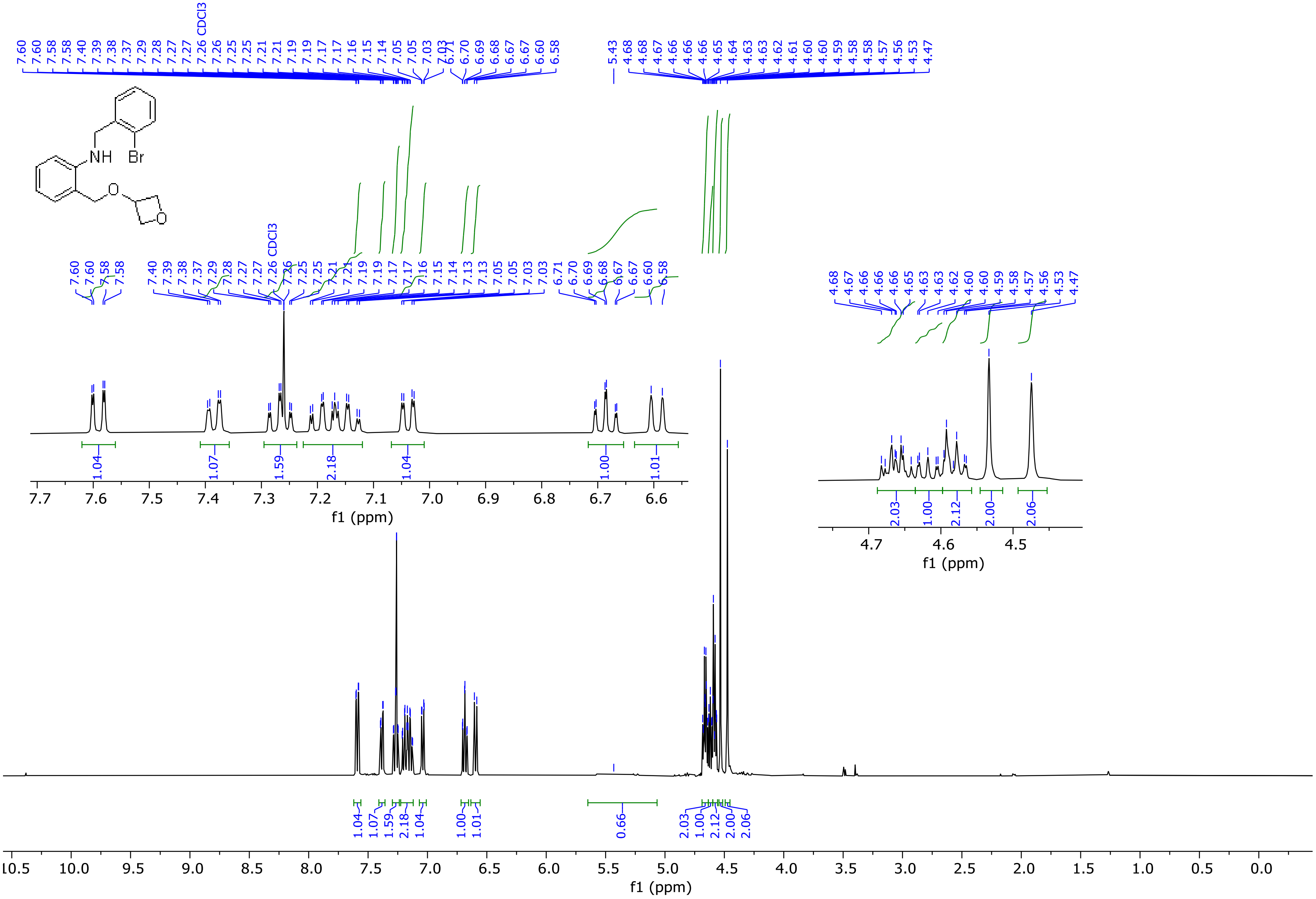

13C{1H}NMR (101 MHz, CDCl3): N-(2-Bromobenzyl)-2-((oxetan-3-yloxy)methyl)aniline (1j)

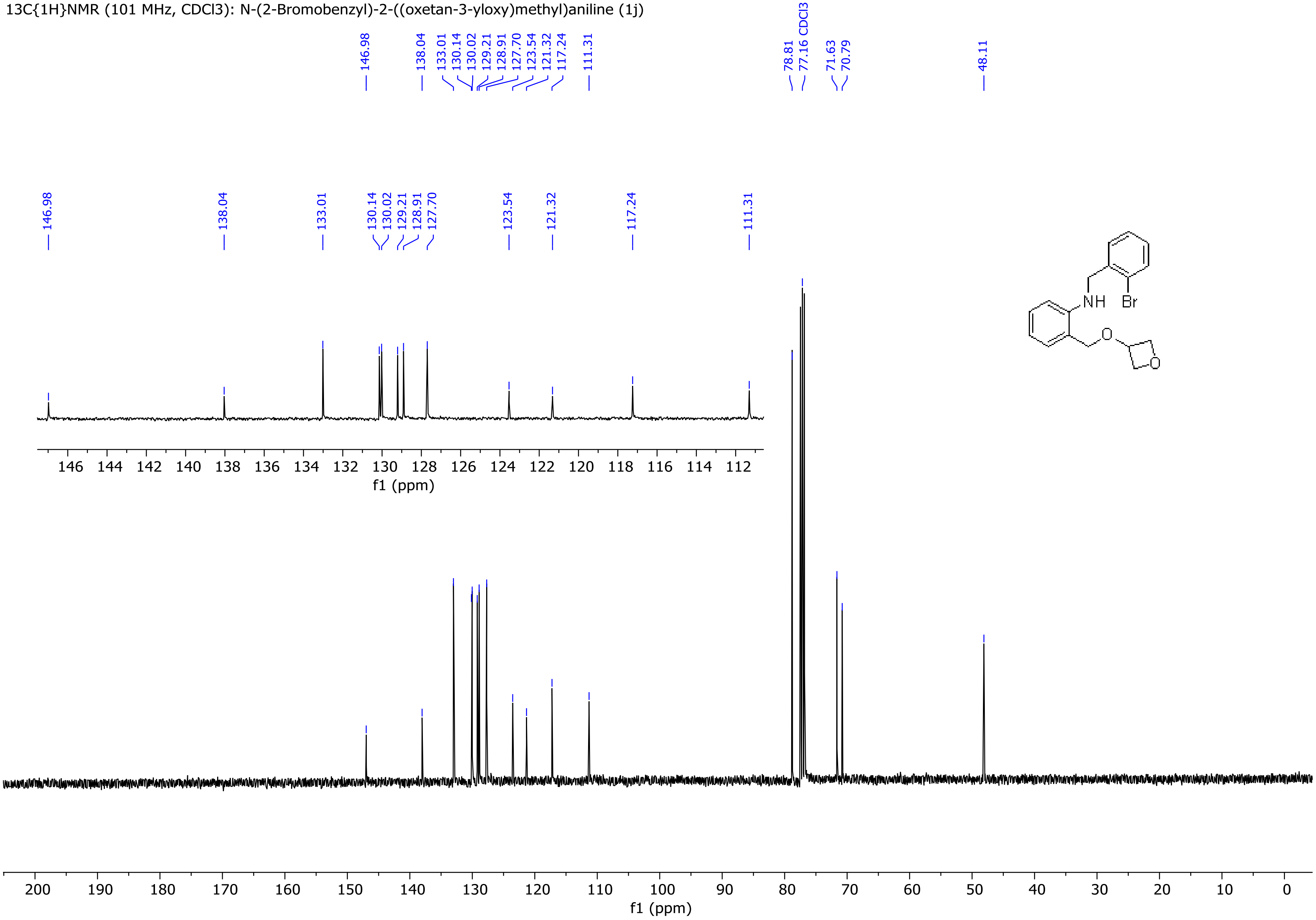

1H NMR: (400 MHz, CDCl3): N-(2-Nitrobenzyl)-2-((oxetan-3-yloxy)methyl)aniline (1k)

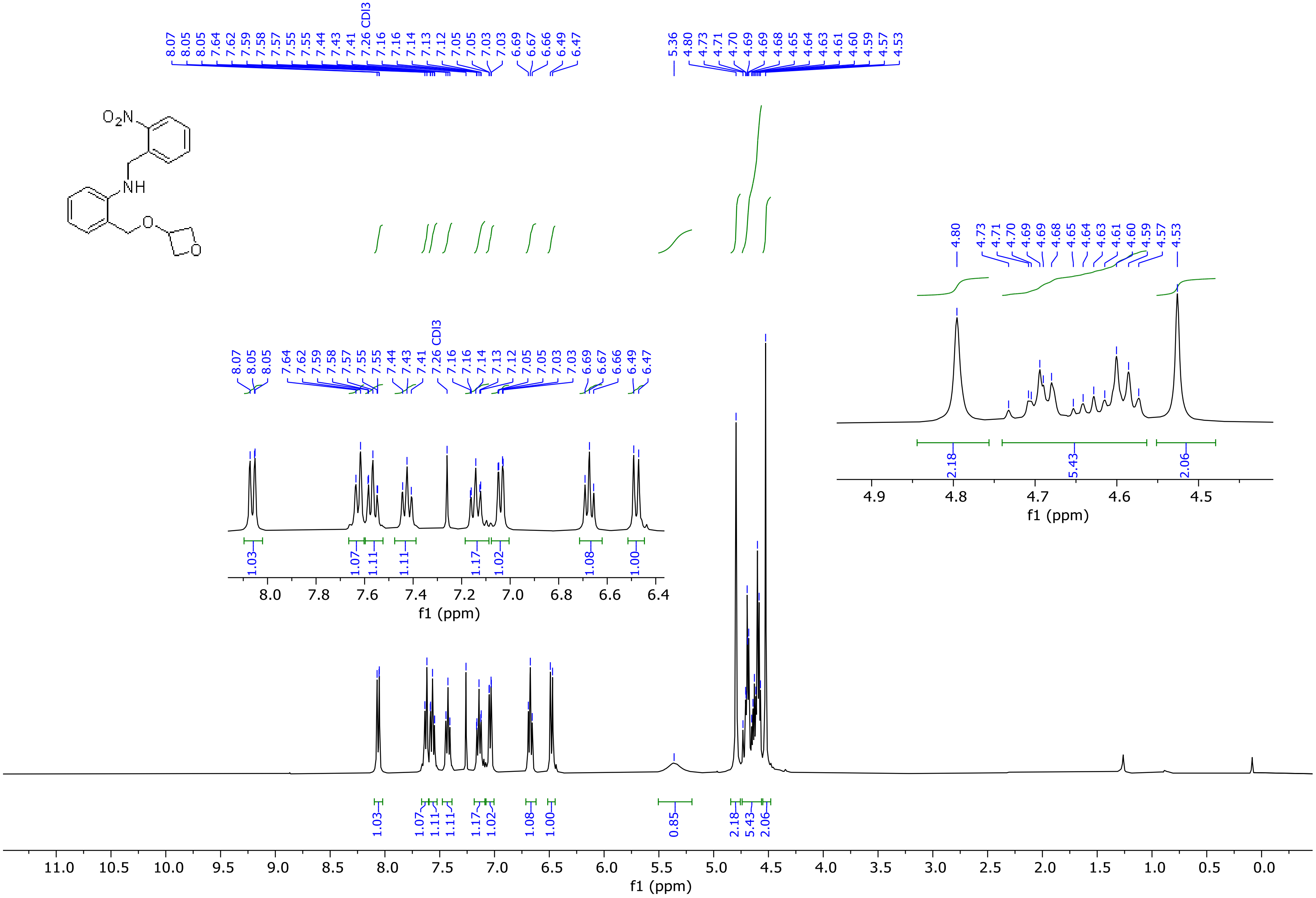

13C{1H}NMR (101 MHz, CDCl3): N-(2-Nitrobenzyl)-2-((oxetan-3-yloxy)methyl)aniline (1k)

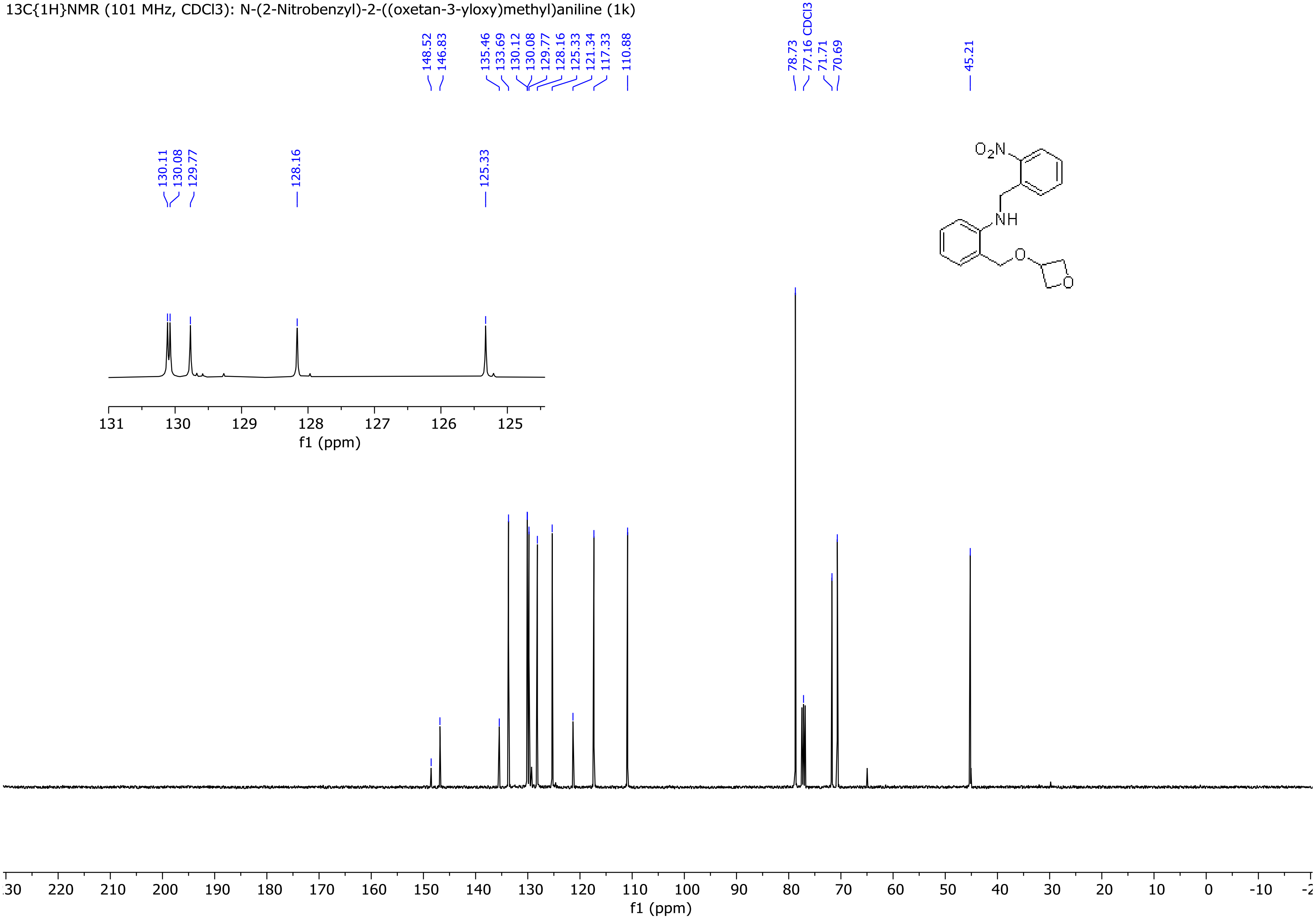

1H NMR: (400 MHz, CDCl3): N-(Naphthalen-2-ylmethyl)-2-((oxetan-3-yloxy)methyl)aniline (1l)

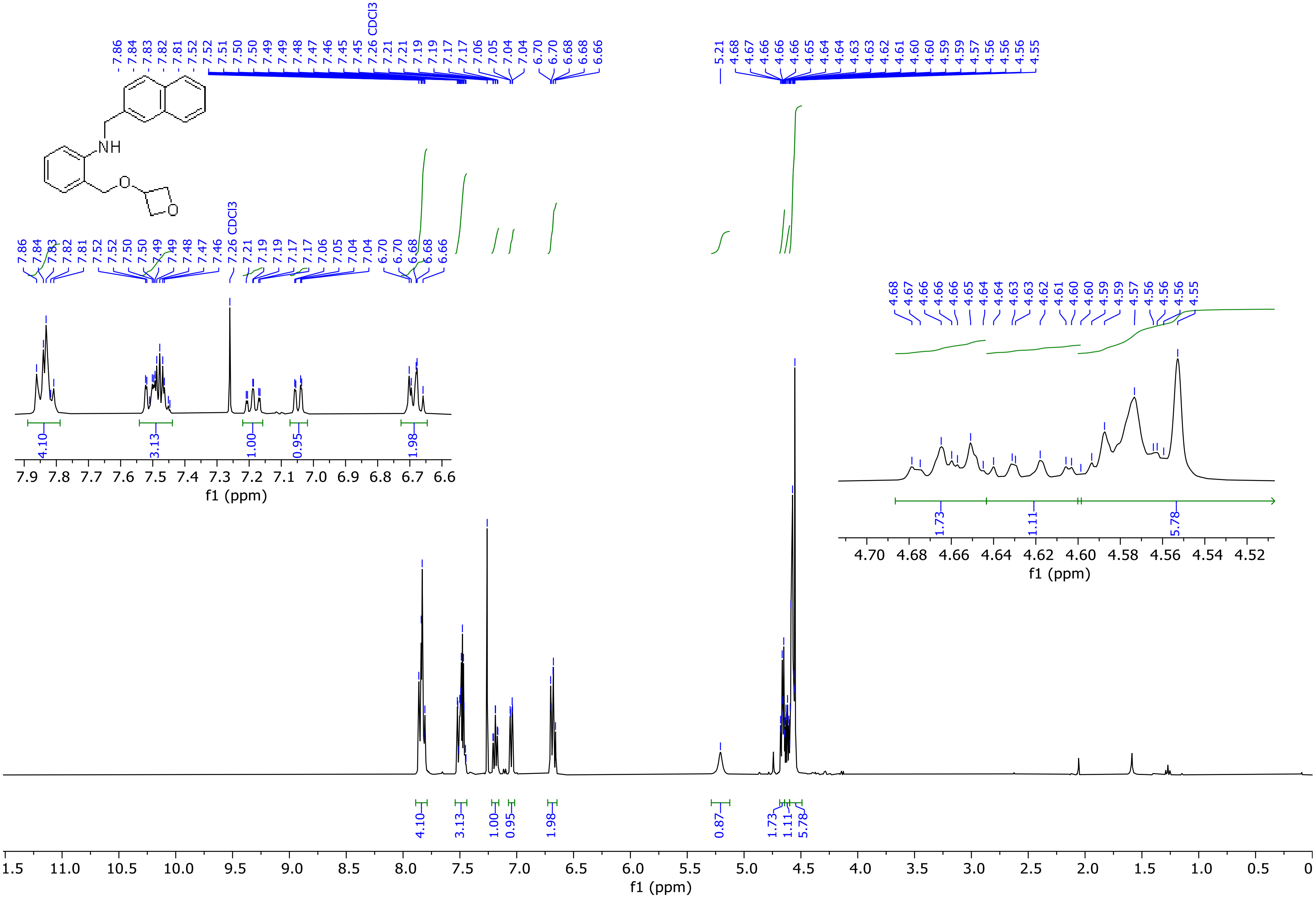

13C{1H}NMR (101 MHz, CDCl3): N-(Naphthalen-2-ylmethyl)-2-((oxetan-3-yloxy)methyl)aniline (1l)

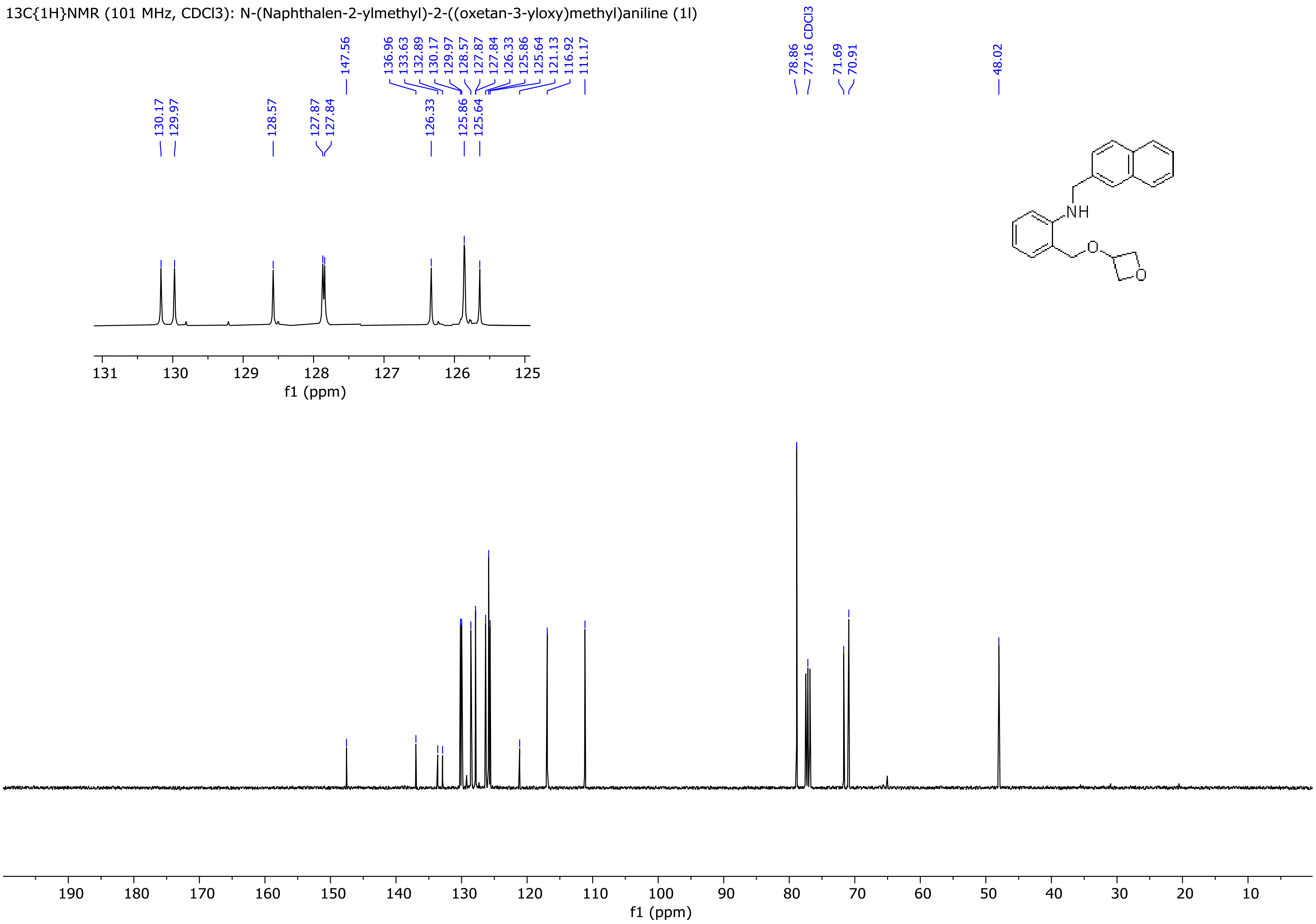

1H NMR: (400 MHz, CDCl3): 2-((Oxetan-3-yloxy)methyl)-N-(thiophen-2-ylmethyl)aniline (1m)

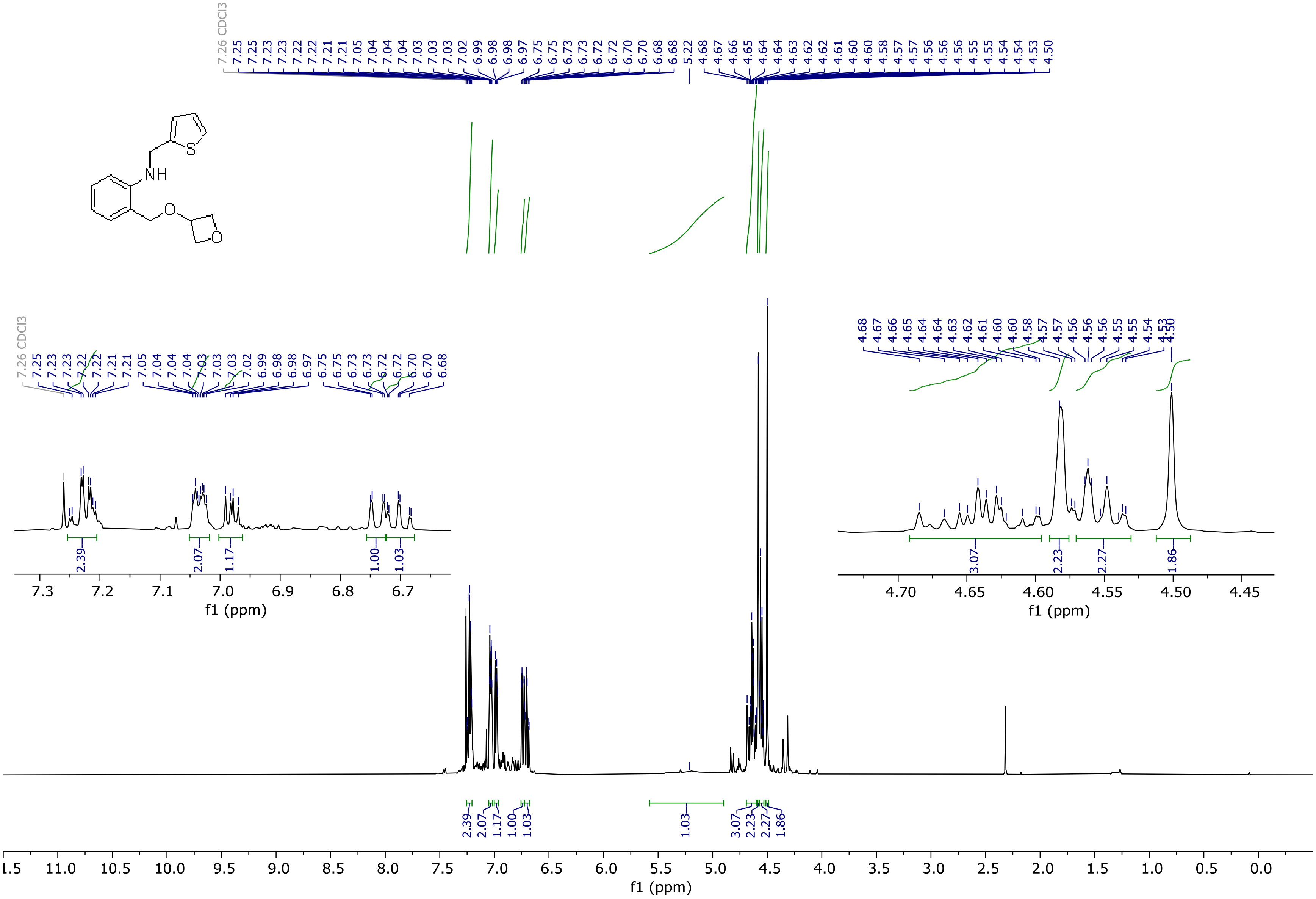

13C{1H}NMR (101 MHz, CDCl3): 2-((Oxetan-3-yloxy)methyl)-N-(thiophen-2-ylmethyl)aniline (1m)

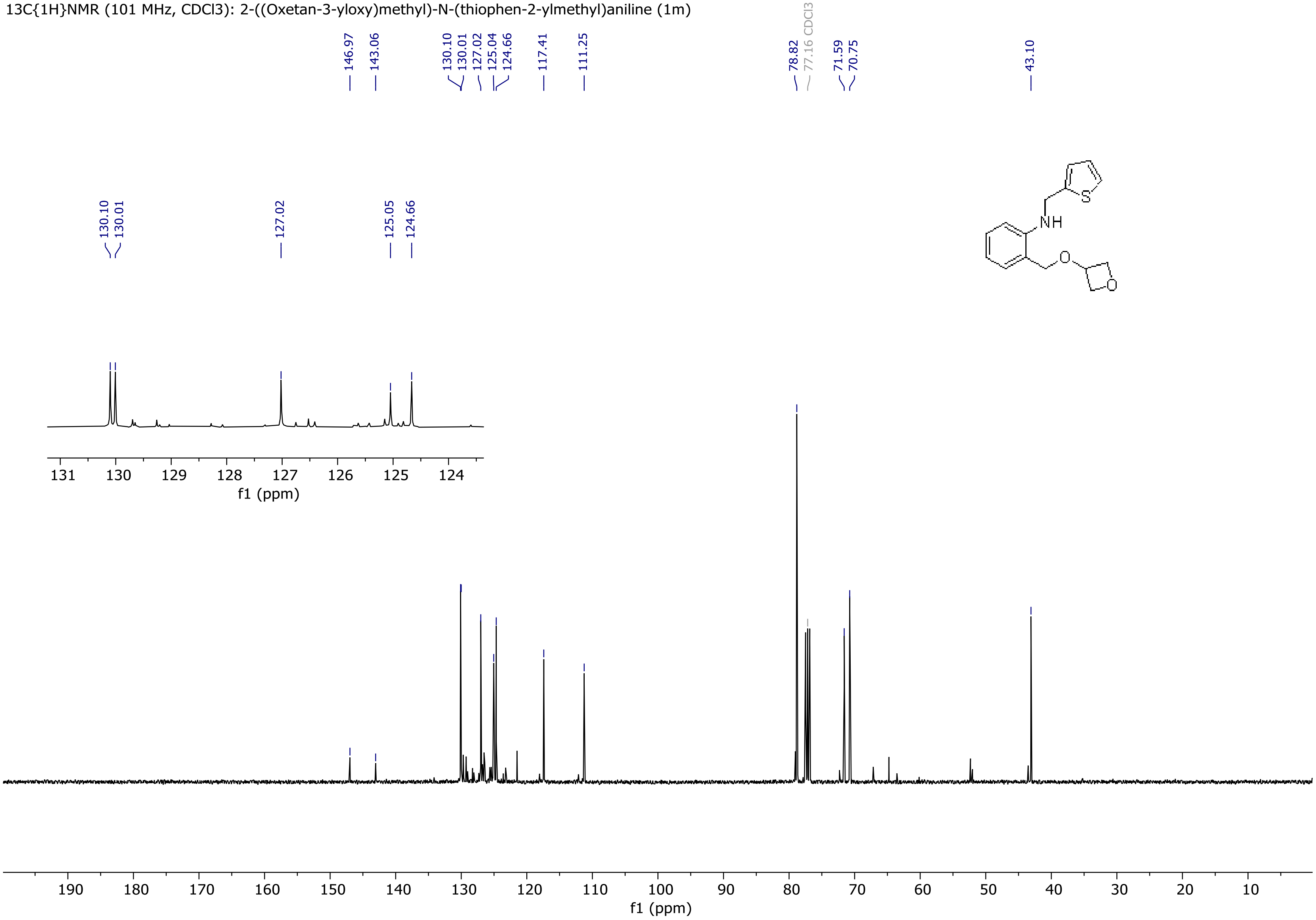

1H NMR: (400 MHz, CDCl3): N-Allyl-2-((oxetan-3-yloxy)methyl)aniline (1n)

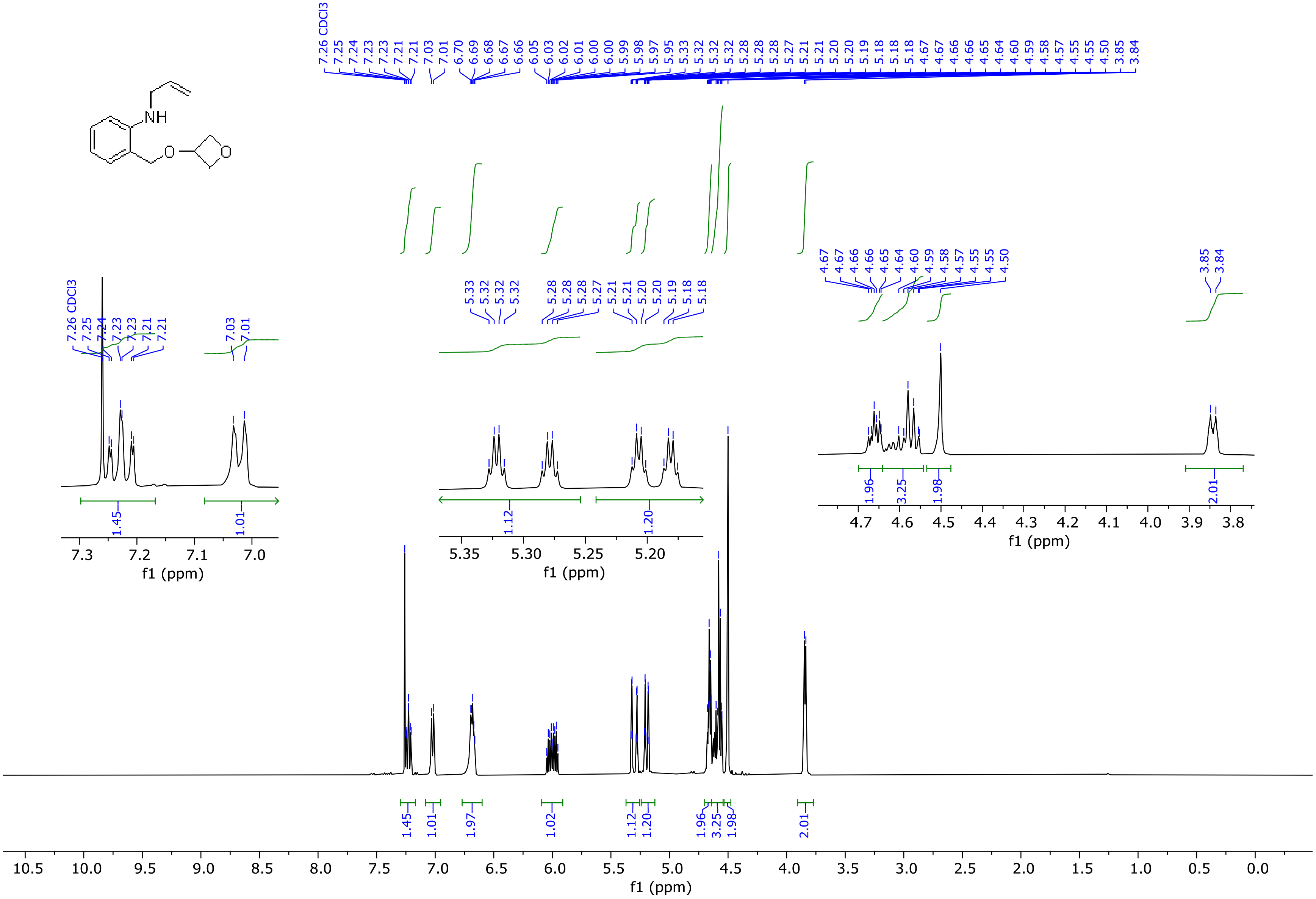

13C{1H}NMR (101 MHz, CDCl3): N-Allyl-2-((oxetan-3-yloxy)methyl)aniline (1n)

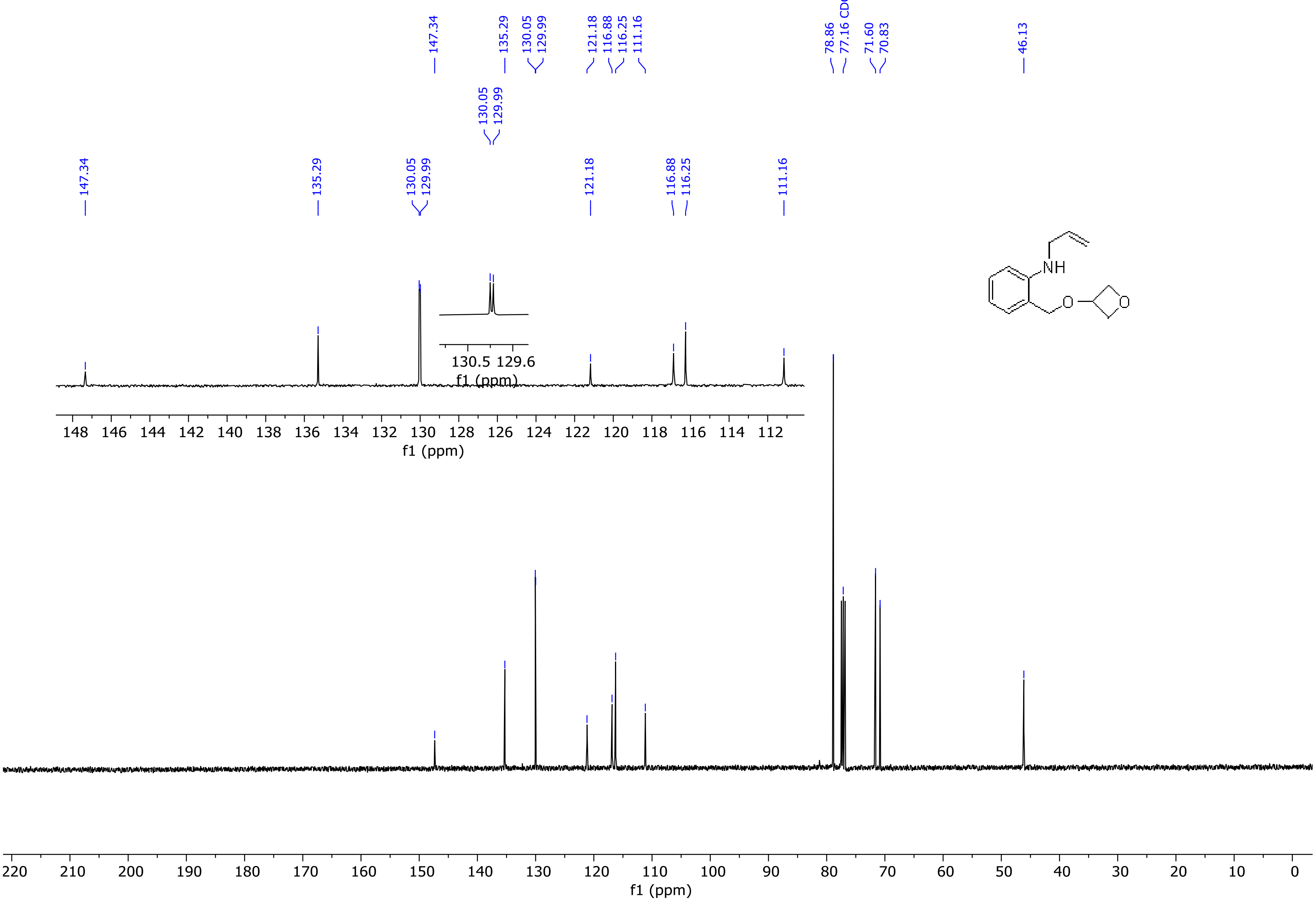

1H NMR: (400 MHz, CDCl3): N-Methyl-2-((oxetan-3-yloxy)methyl)aniline (1o)

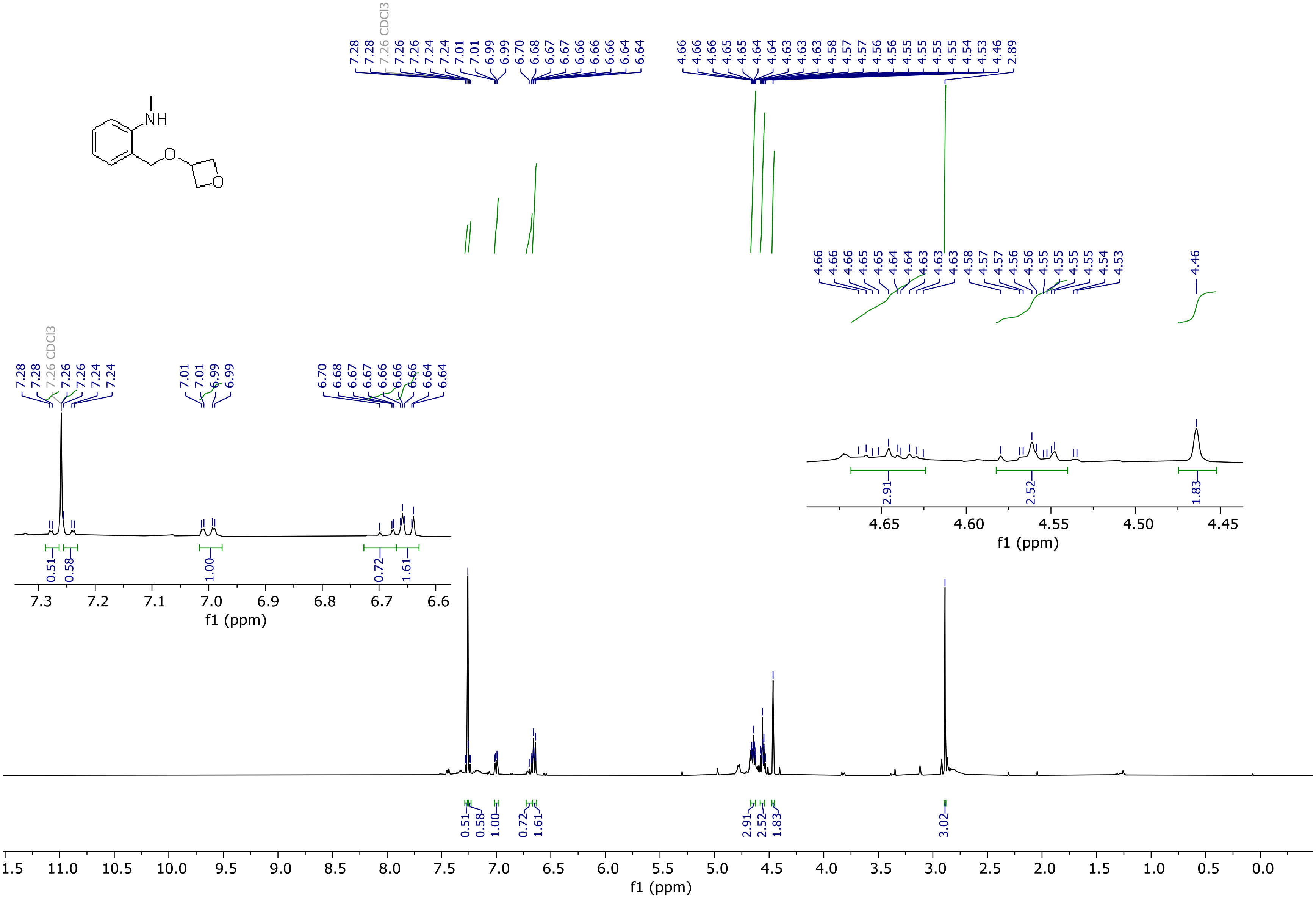

13C{1H}NMR (101 MHz, CDCl3): N-Methyl-2-((oxetan-3-yloxy)methyl)aniline (1o)

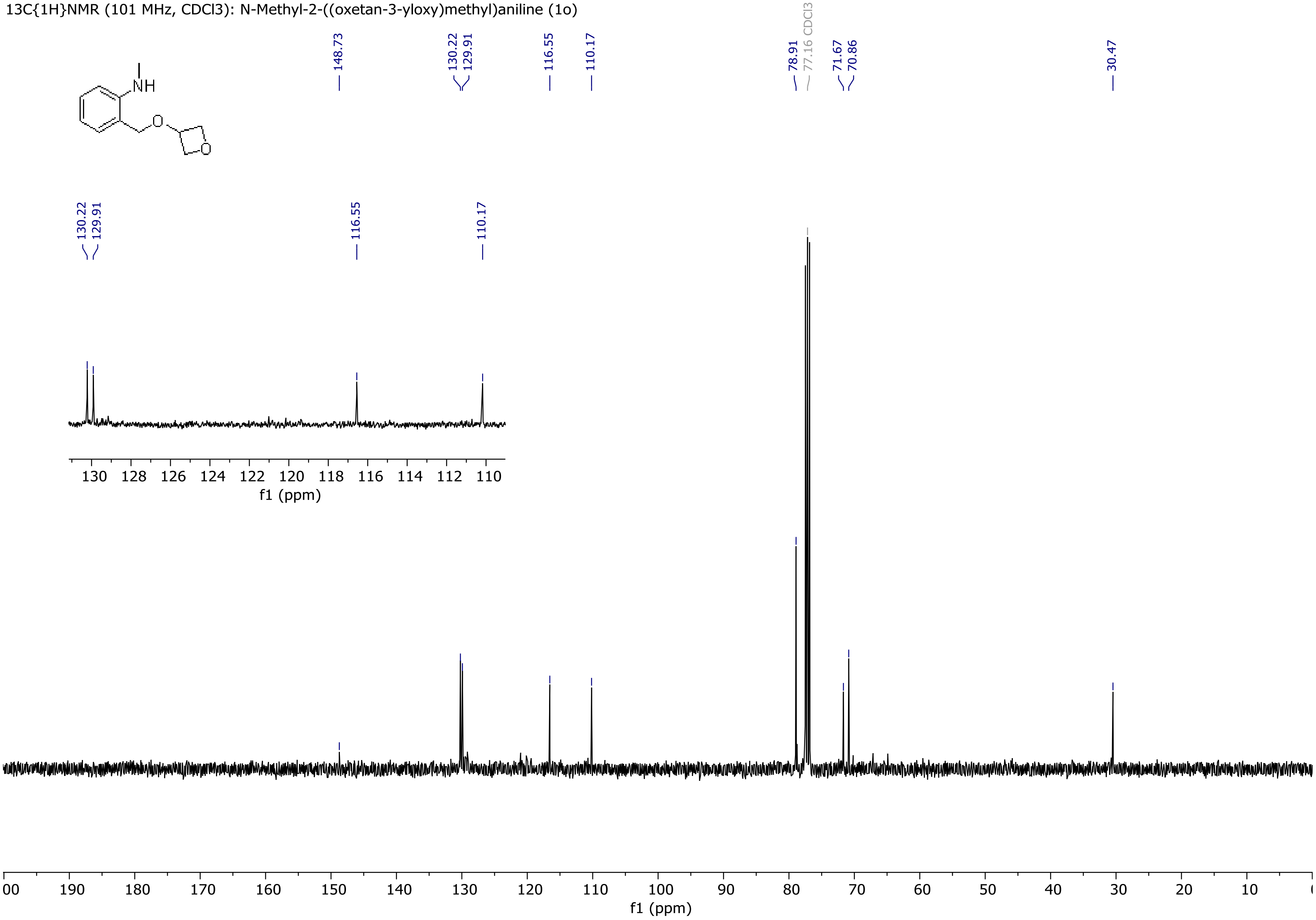

1H NMR: (400 MHz, CDCl3): tert-Butyl (2-((oxetan-3-yloxy)methyl)phenyl)carbamate (1p)

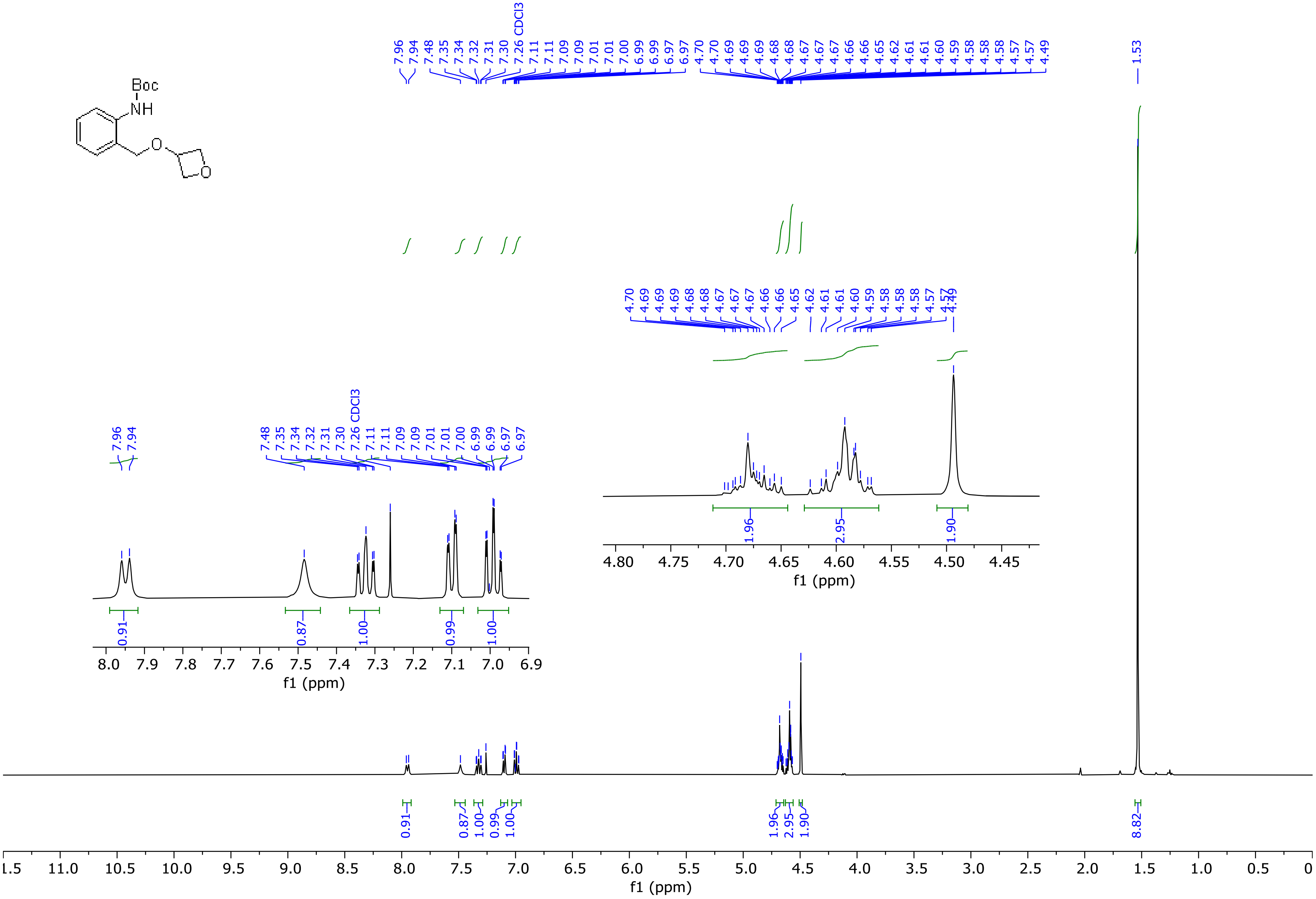

13C{1H}NMR (101 MHz, CDCl3): tert-Butyl (2-((oxetan-3-yloxy)methyl)phenyl)carbamate (1p)

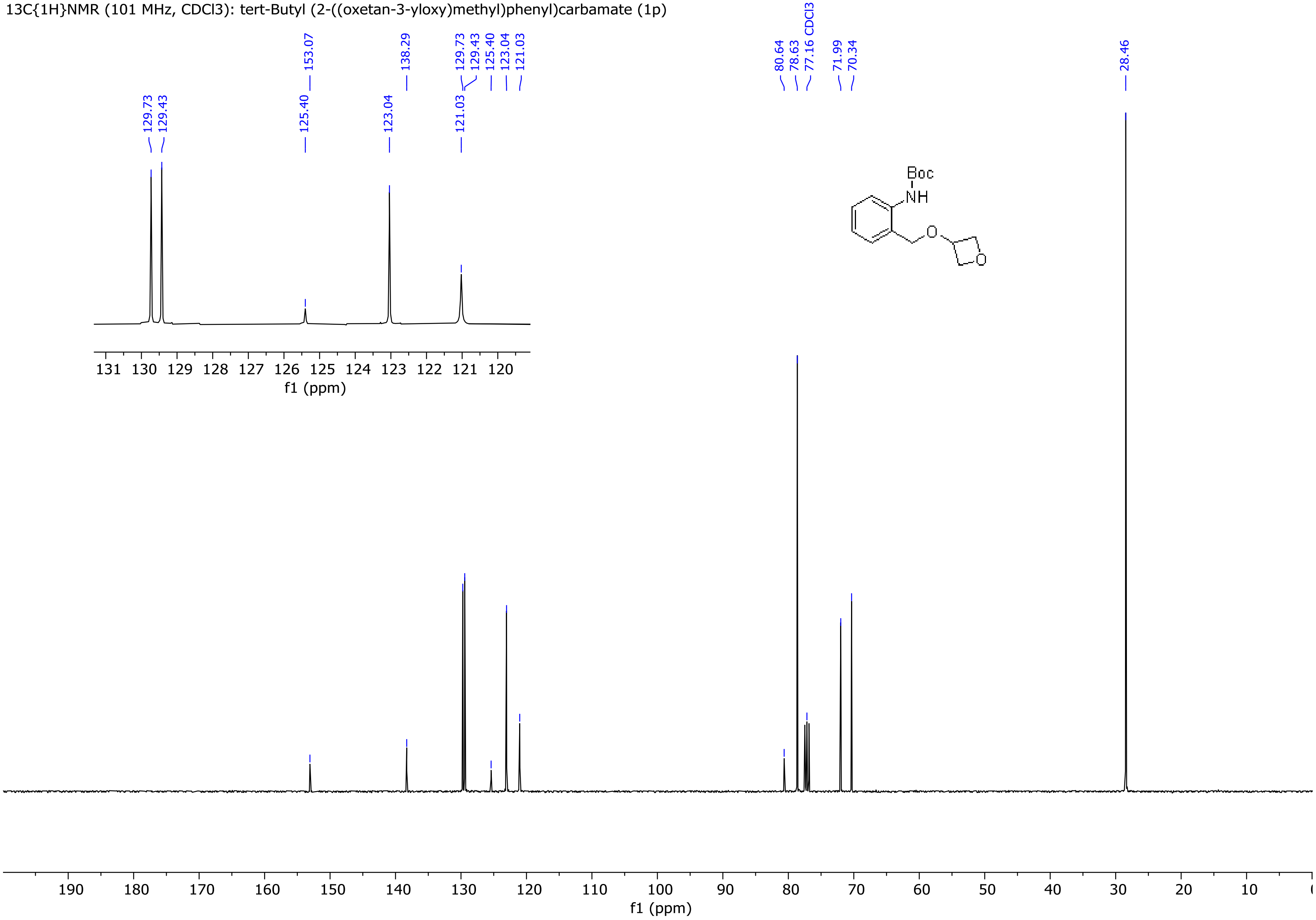

1H NMR: (400 MHz, CDCl3): N-Benzyl-2-methyl-6-((oxetan-3-yloxy)methyl)aniline (1v)

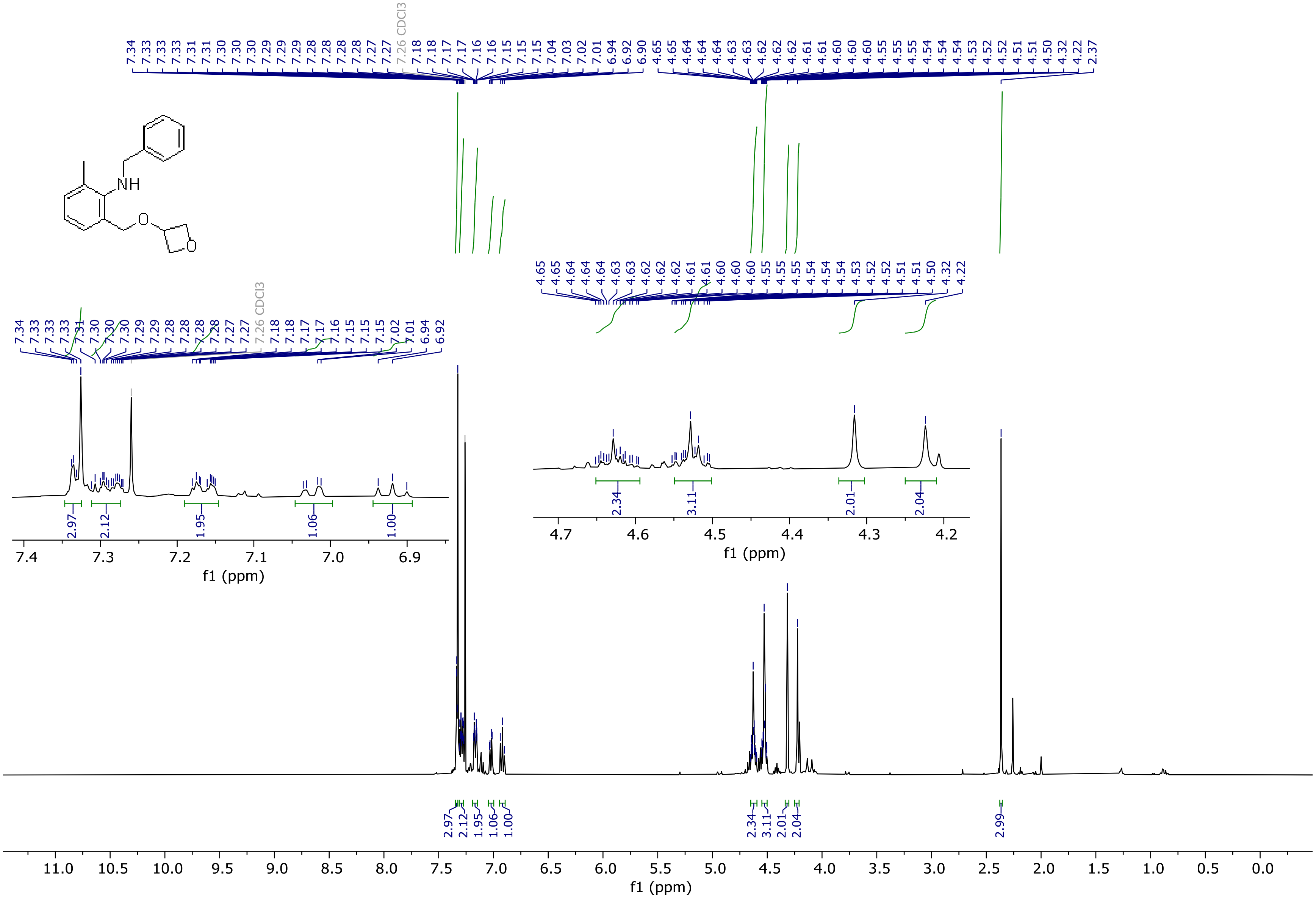

13C{1H}NMR (101 MHz, CDCl3): N-Benzyl-2-methyl-6-((oxetan-3-yloxy)methyl)aniline (1v)

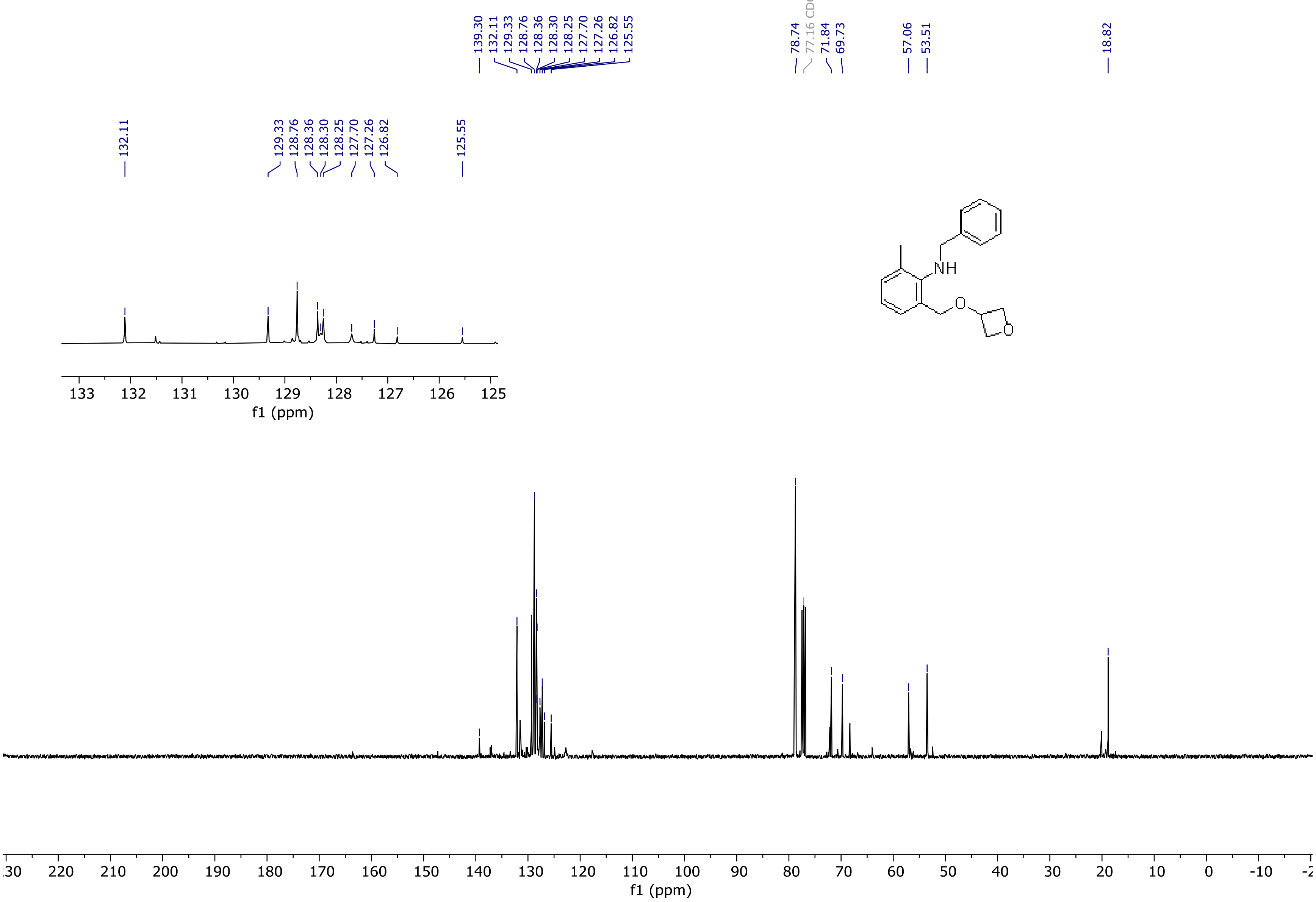

1H NMR: (400 MHz, CDCl3): N-Benzyl-5-chloro-2-((oxetan-3-yloxy)methyl)aniline (1w)

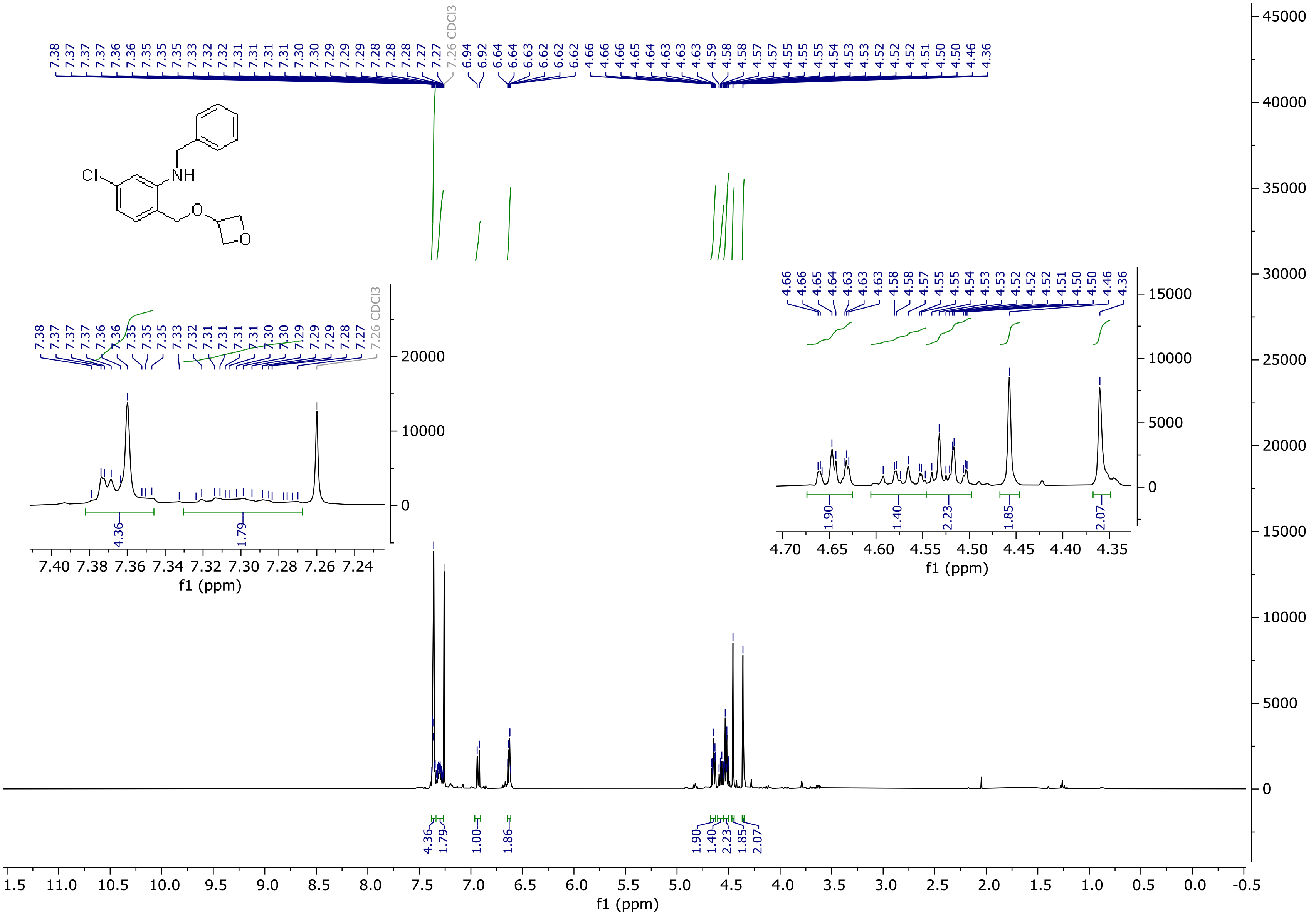

13C{1H}NMR (101 MHz, CDCl3): N-Benzyl-5-chloro-2-((oxetan-3-yloxy)methyl)aniline (1w)

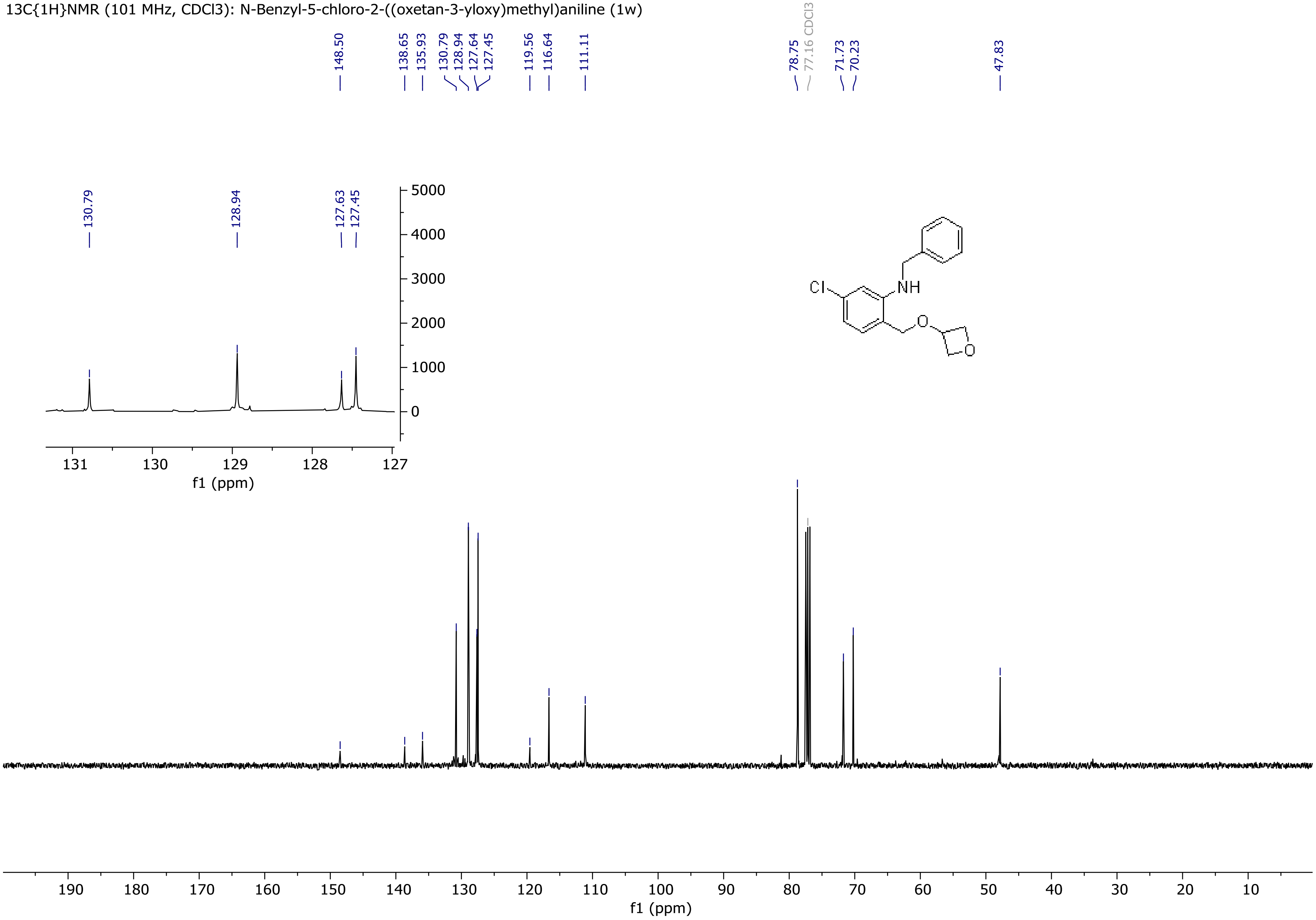

1H NMR: (400 MHz, CDCl3): N-Benzyl-5-fluoro-2-((oxetan-3-yloxy)methyl)aniline (1x)

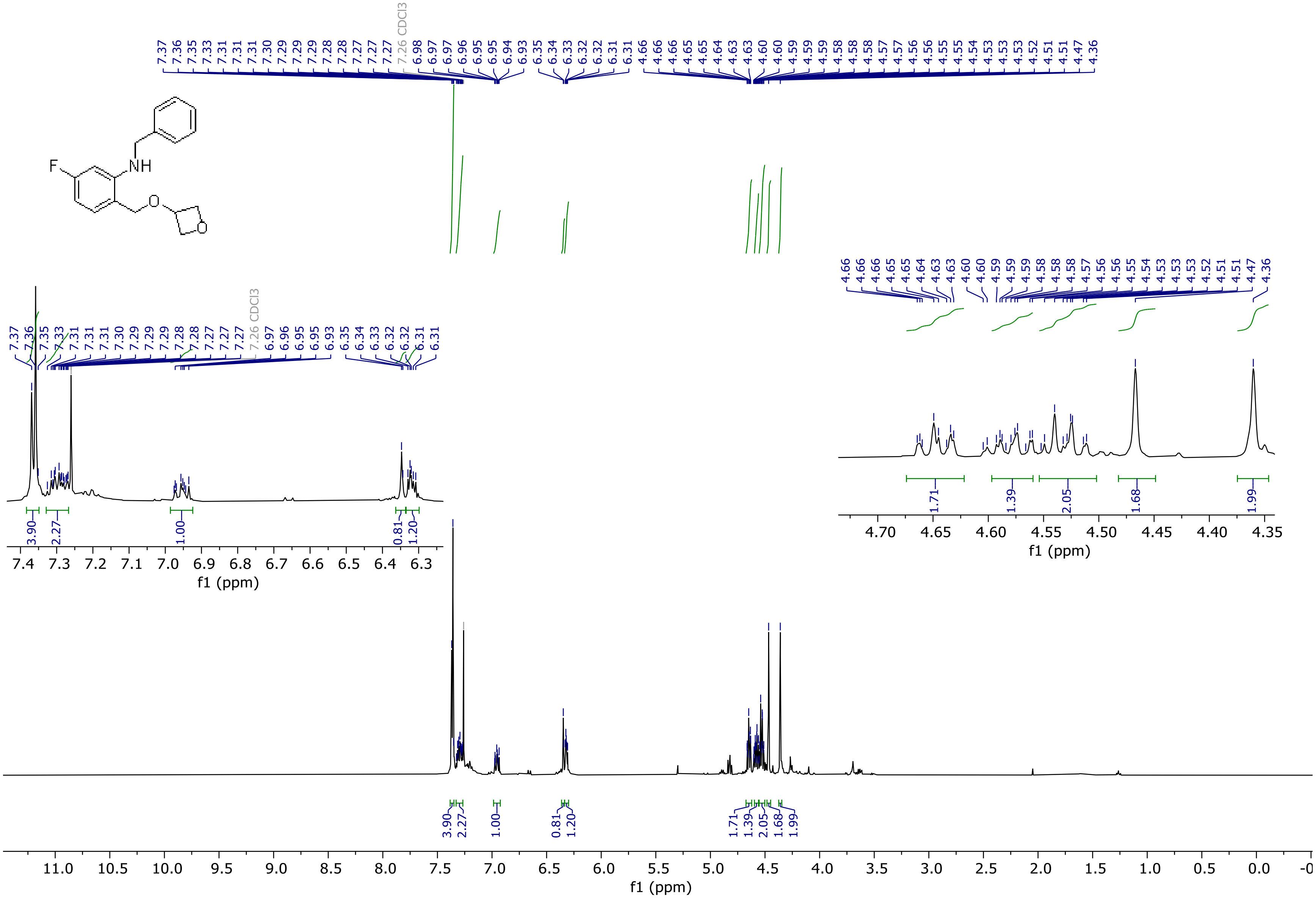

13C{1H}NMR (101 MHz, CDCl3): N-Benzyl-5-fluoro-2-((oxetan-3-yloxy)methyl)aniline (1x)

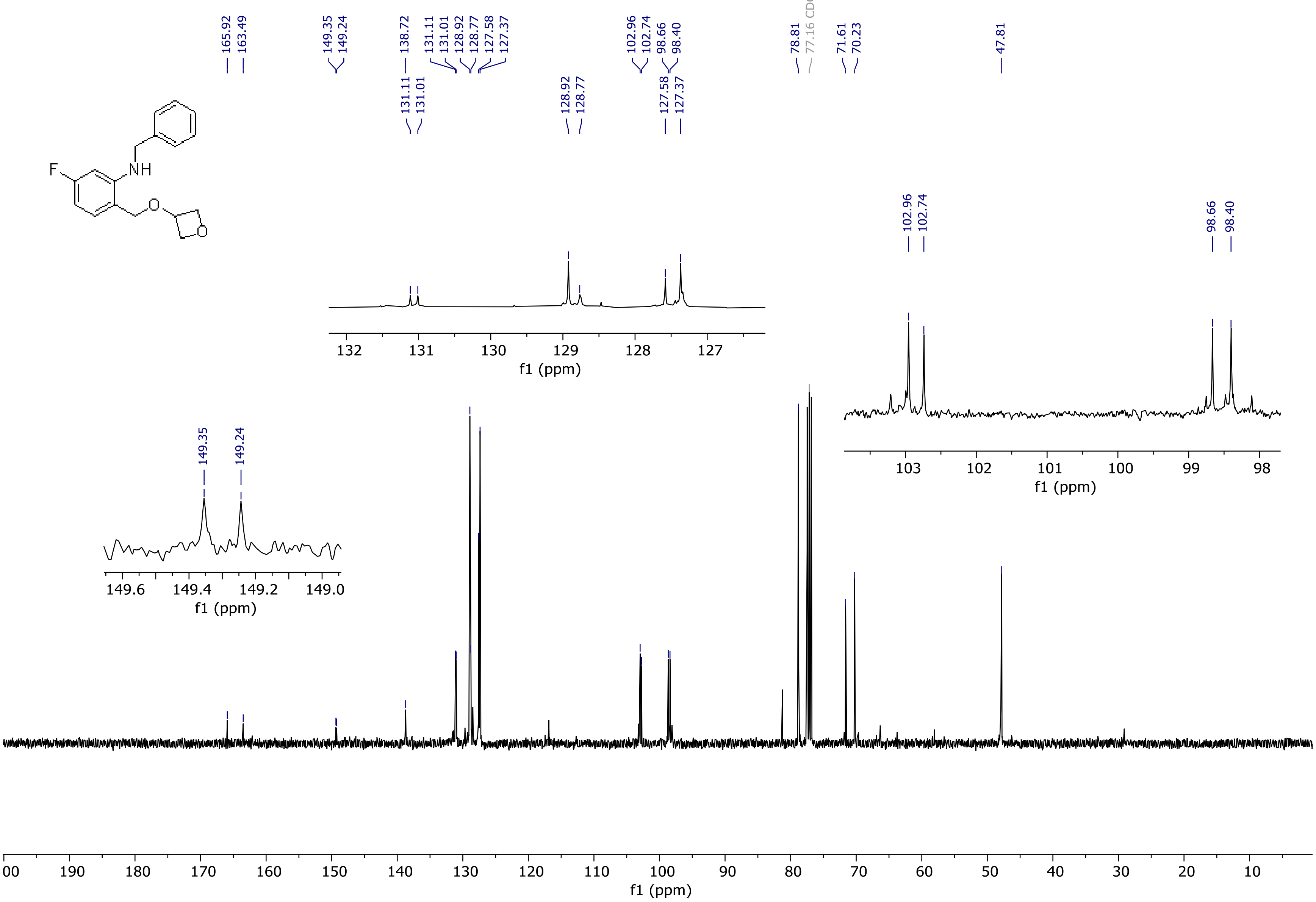

19F NMR (376 MHz, CDCl3): N-Benzyl-5-fluoro-2-((oxetan-3-yloxy)methyl)aniline (1x)

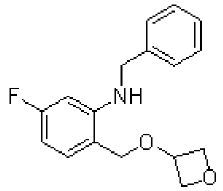

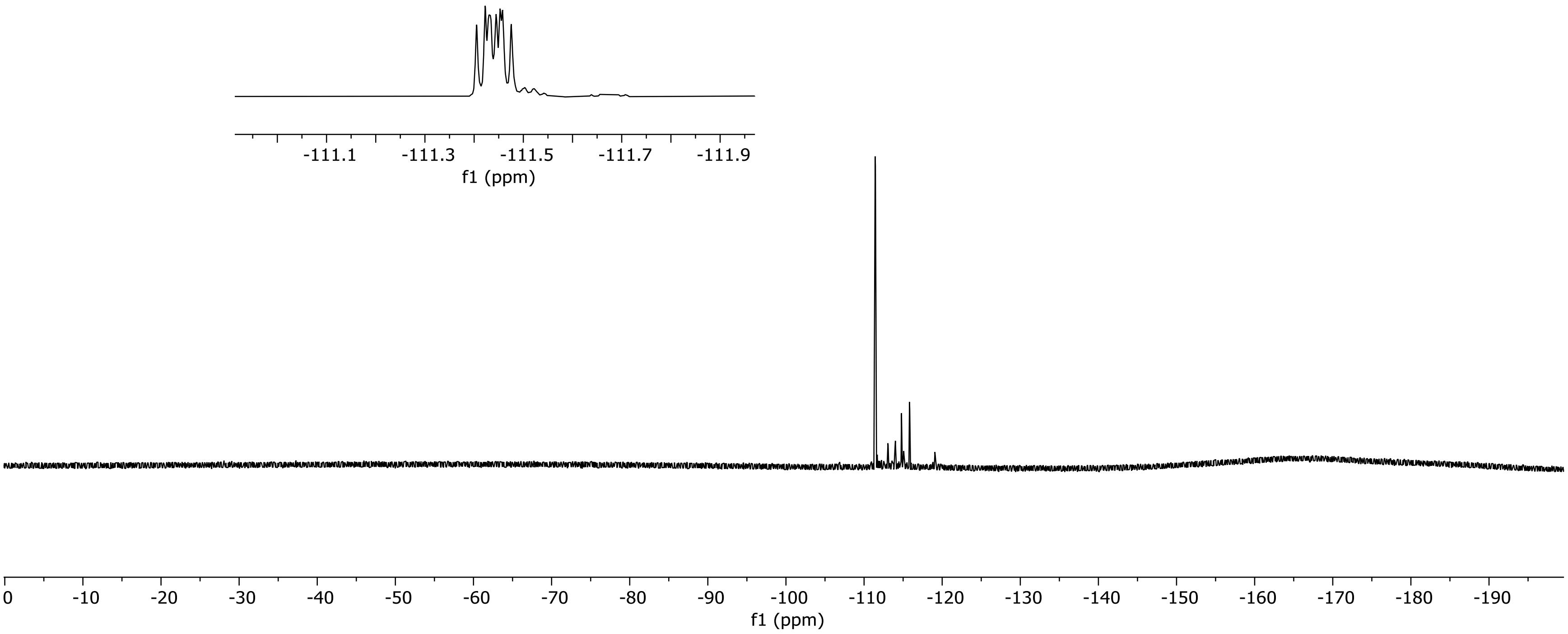

1H NMR (400 MHz, CDCl3): N-benzyl-5-methyl-2-((oxetan-3-yloxy)methyl)aniline (1y)

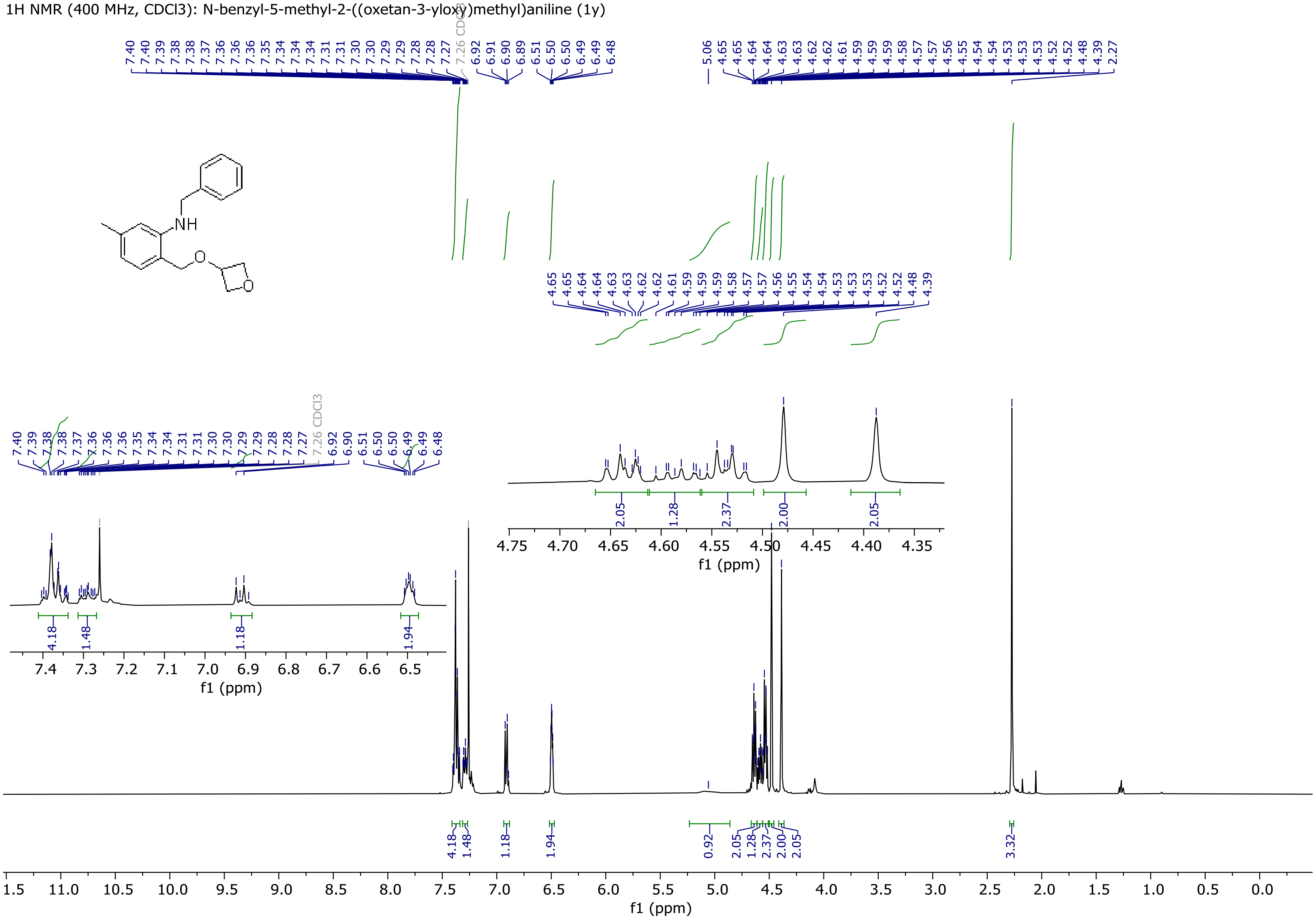

13C{1H}NMR (101 MHz, CDCl3): N-benzyl-5-methyl-2-((oxetan-3-yloxy)methyl)aniline (1y)

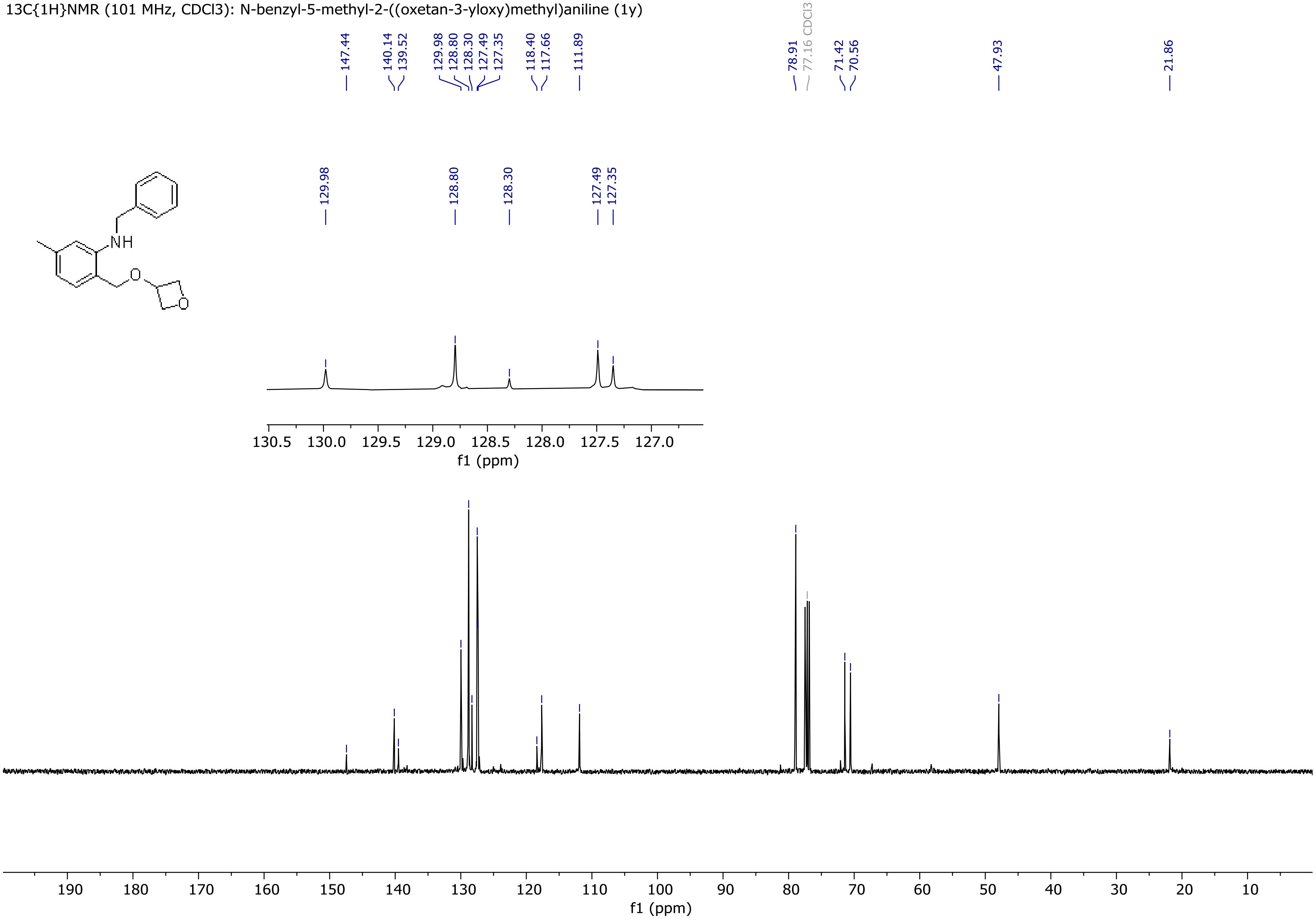

1H NMR (400 MHz, CDCl3): N-benzyl-5-methoxy-2-((oxetan-3-yloxy)methyl)aniline (1z)

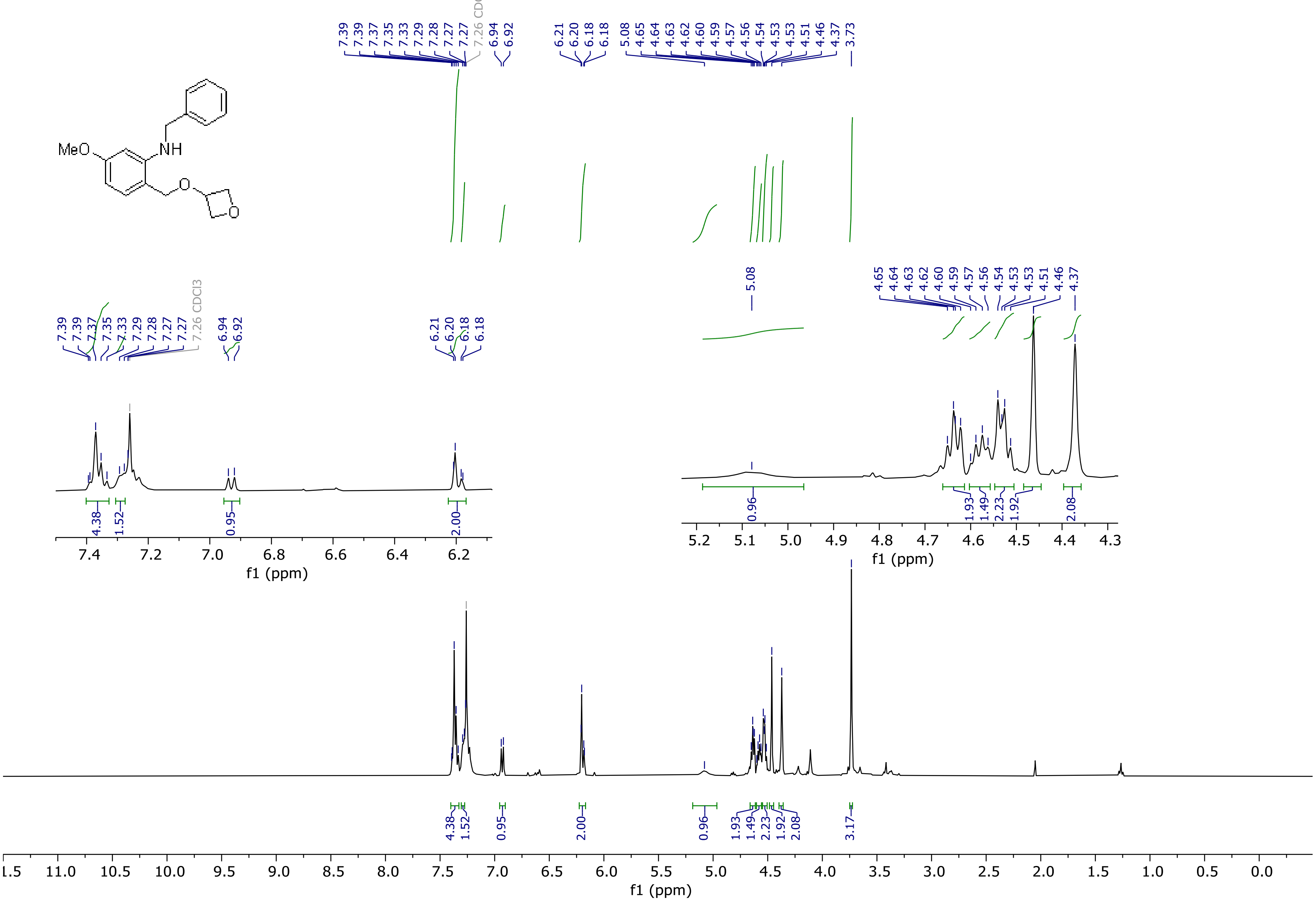

13C{1H}NMR (101 MHz, CDCl3): N-benzyl-5-methoxy-2-((oxetan-3-yloxy)methyl)aniline (1z)

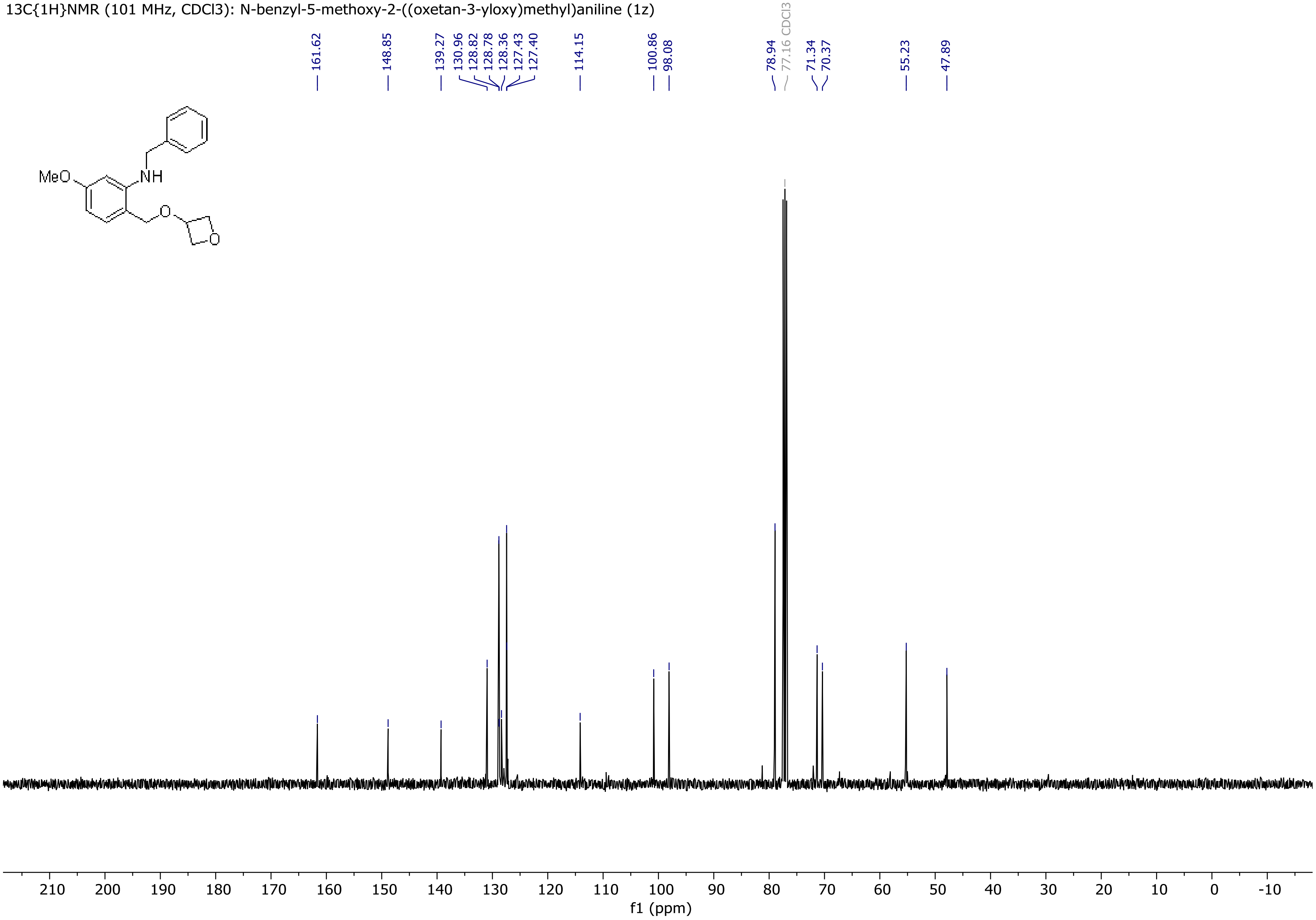

1H NMR: (400 MHz, CDCl3): 2-((Oxetan-3-yloxy)methyl)-N-phenylaniline (1q)

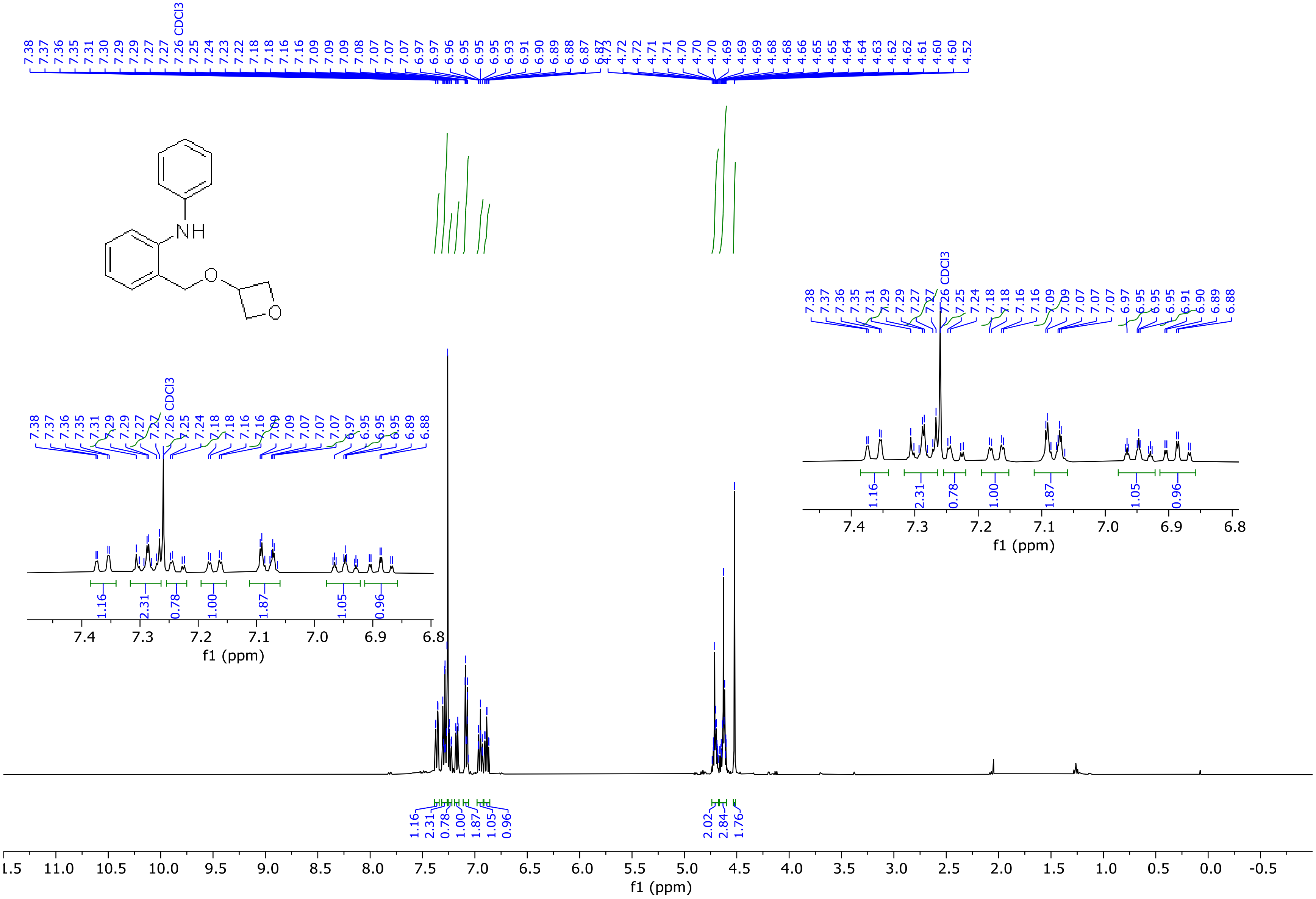

13C{1H}NMR (101 MHz, CDCl3): 2-((Oxetan-3-yloxy)methyl)-N-phenylaniline (1q)

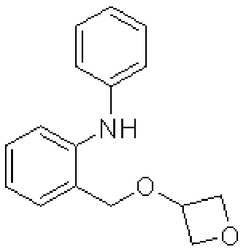

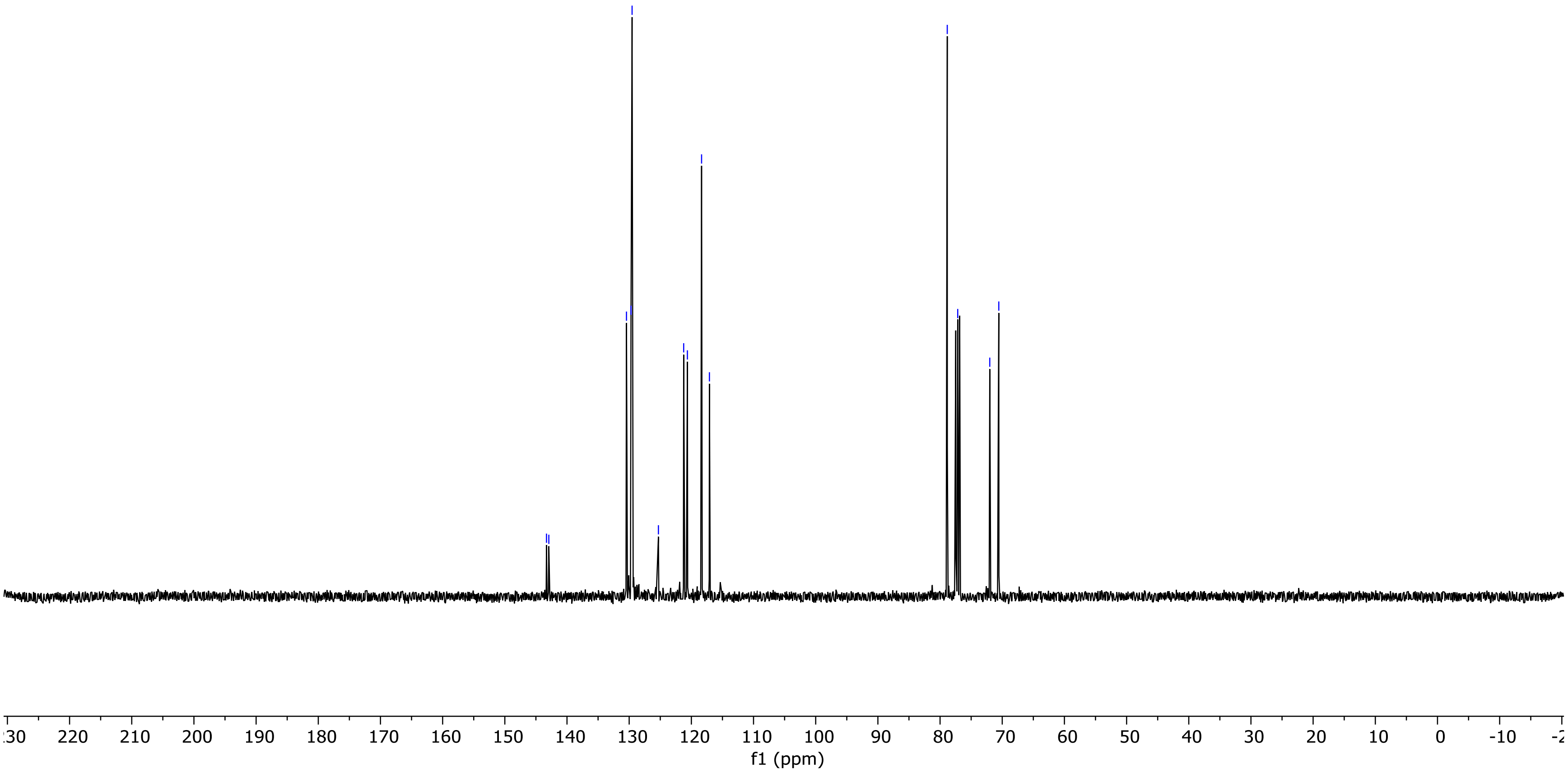

1H NMR: (400 MHz, CDCl3): N-(2-((Oxetan-3-yloxy)methyl)phenyl)naphthalen-1-amine (1r)

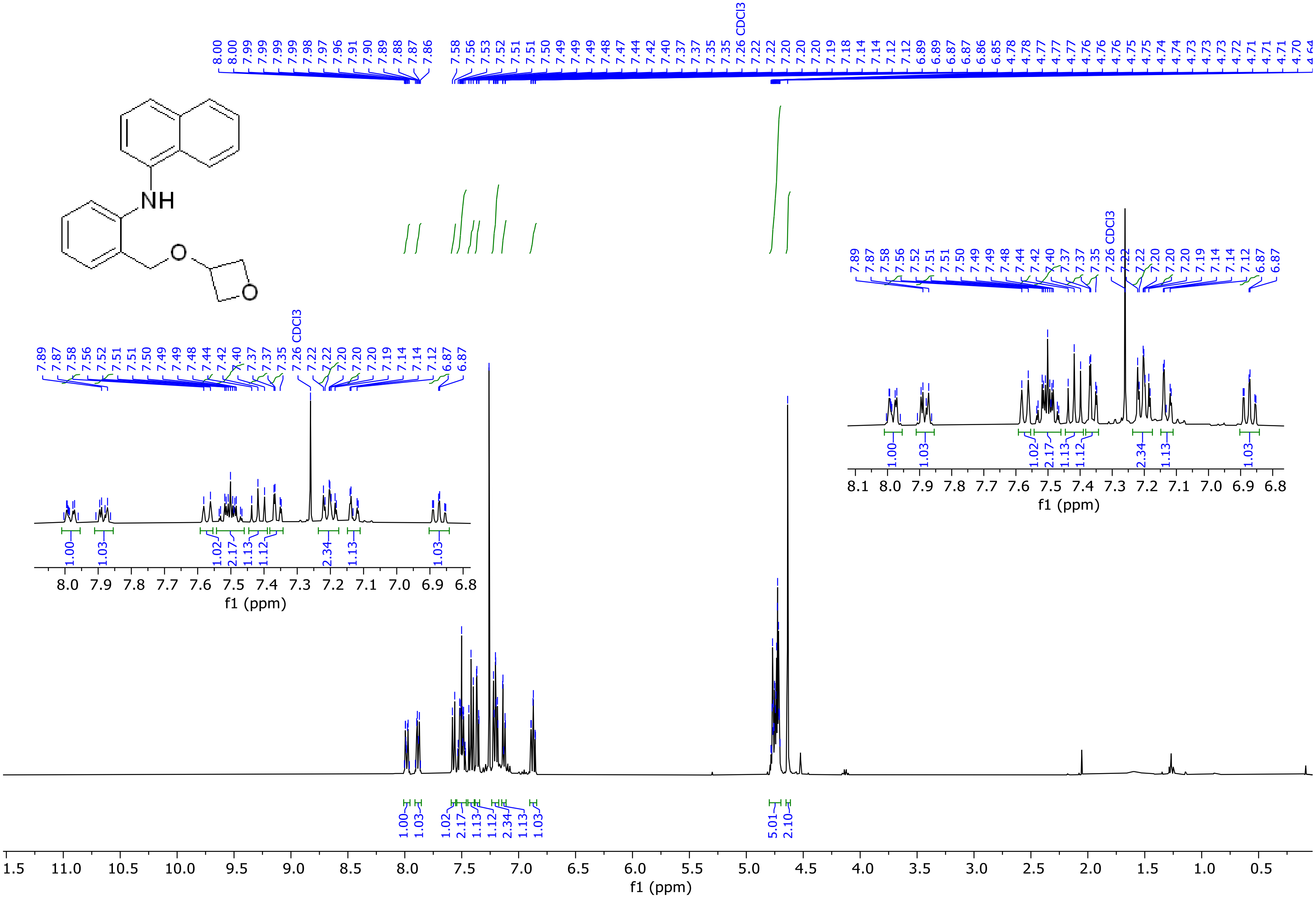

13C{1H}NMR (101 MHz, CDCl3): N-(2-((Oxetan-3-yloxy)methyl)phenyl)naphthalen-1-amine (1r)

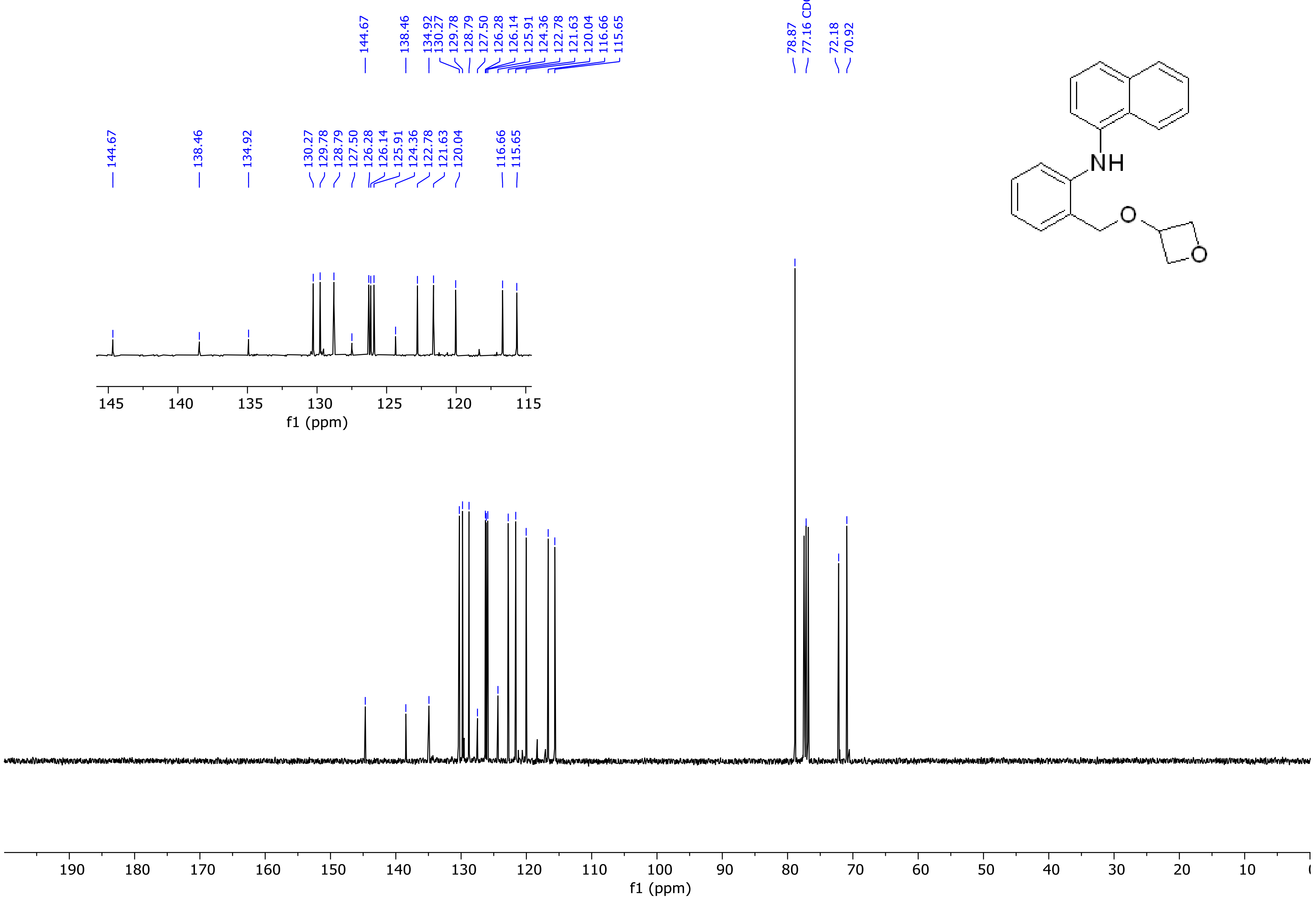

1H NMR: (400 MHz, CDCl3): N-2-((Oxetan-3-yloxy)methyl)-(4-(trifluoromethyl)phenyl)aniline (1s)

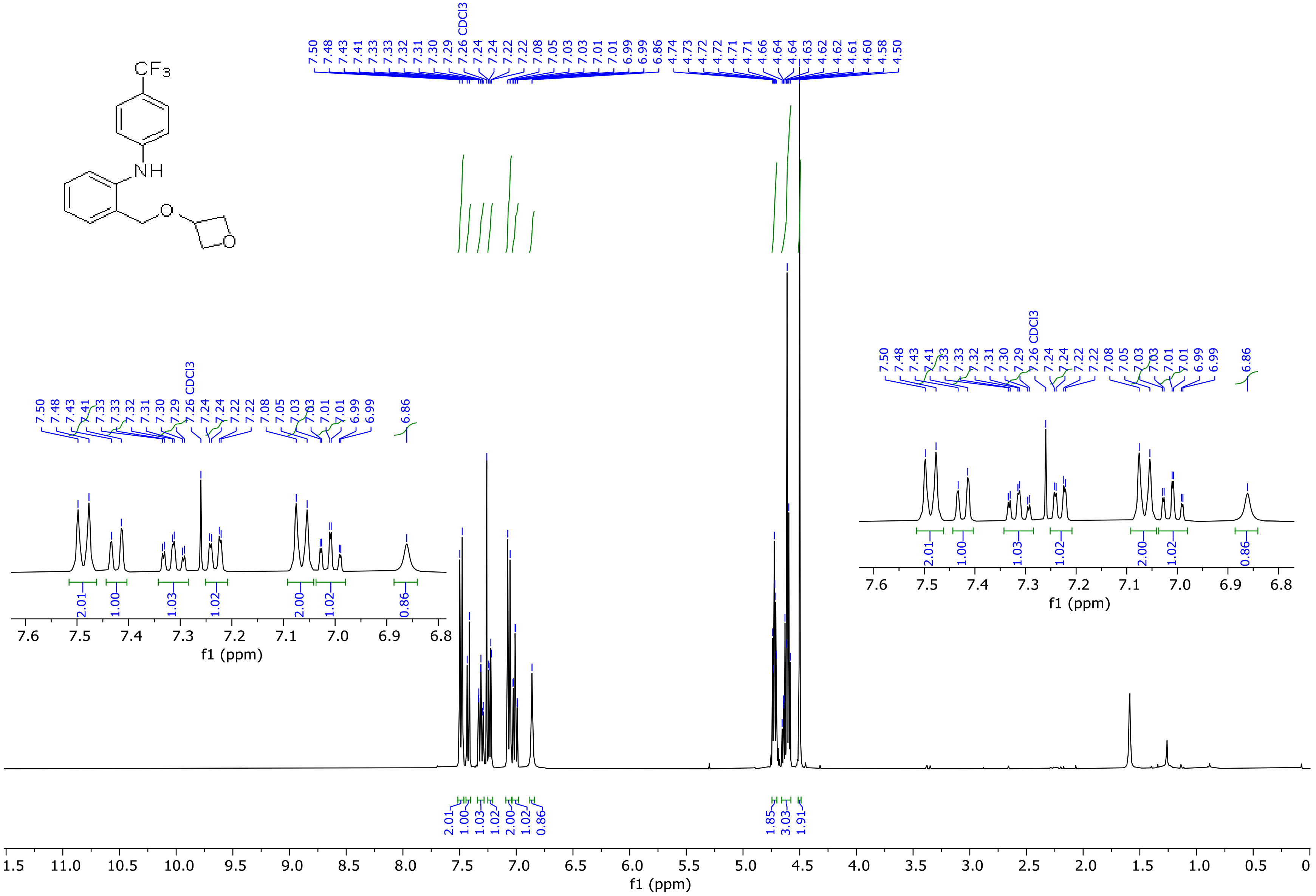

13C{1H}NMR (101 MHz, CDCl3): N-2-((Oxetan-3-yloxy)methyl)-(4-(trifluoromethyl)phenyl)aniline (1s)

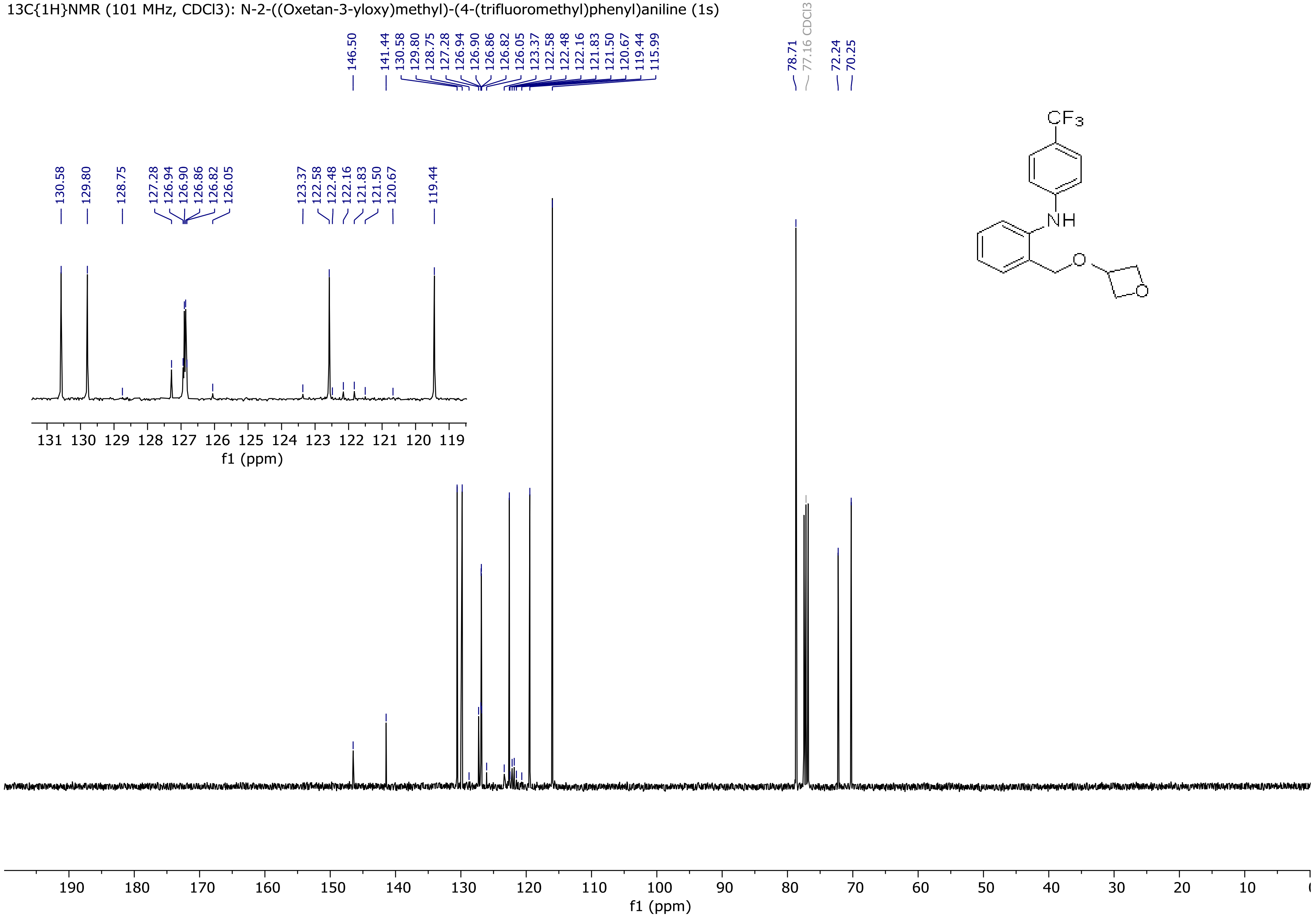

19F NMR (376 MHz, CDCl3): N-2-((Oxetan-3-yloxy)methyl)-(4-(trifluoromethyl)phenyl)aniline (1s)

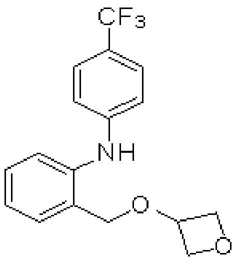

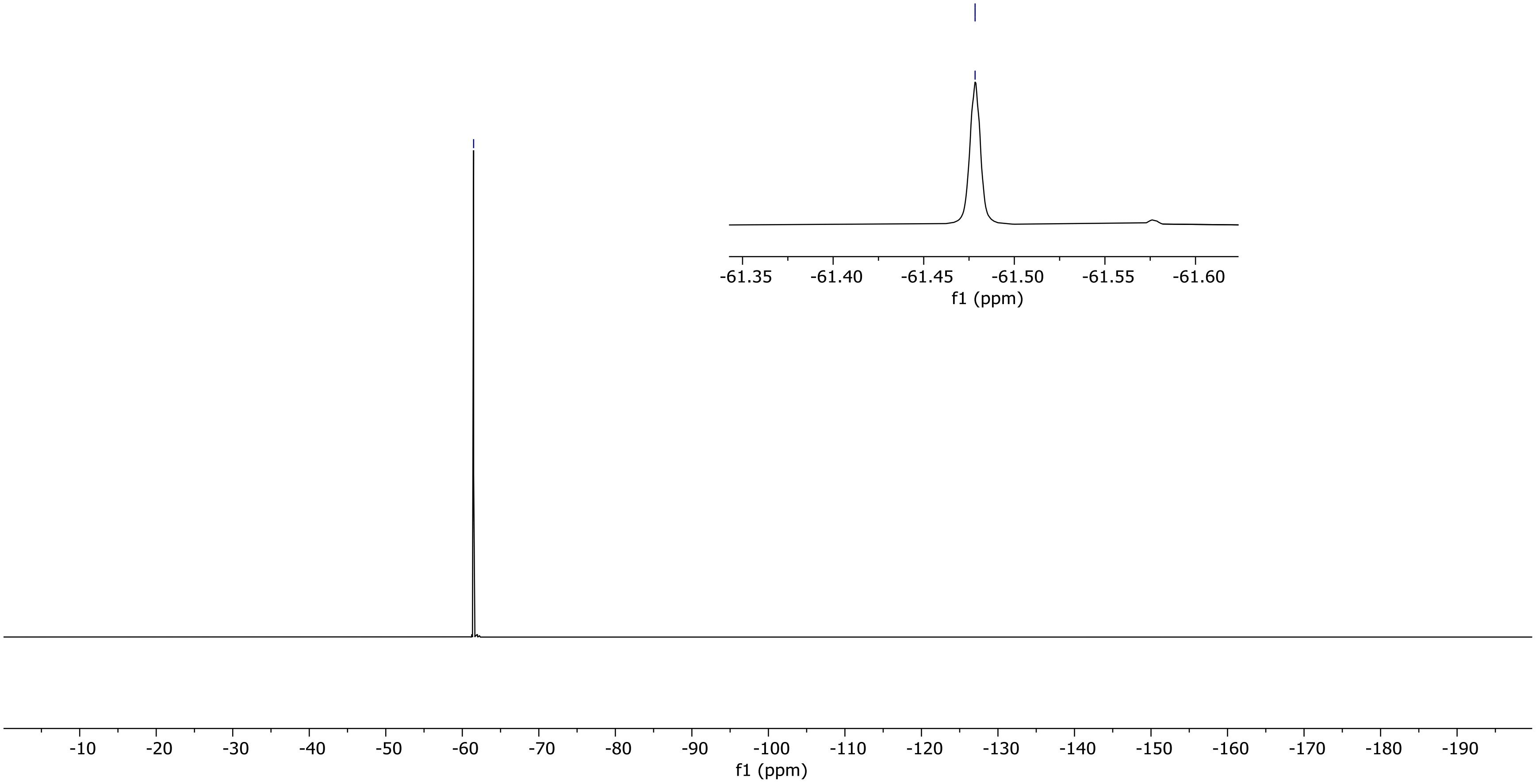

1H NMR: (400 MHz, CDCl3): N-(4-Methoxyphenyl)-2-((oxetan-3-yloxy)methyl)aniline (1t)

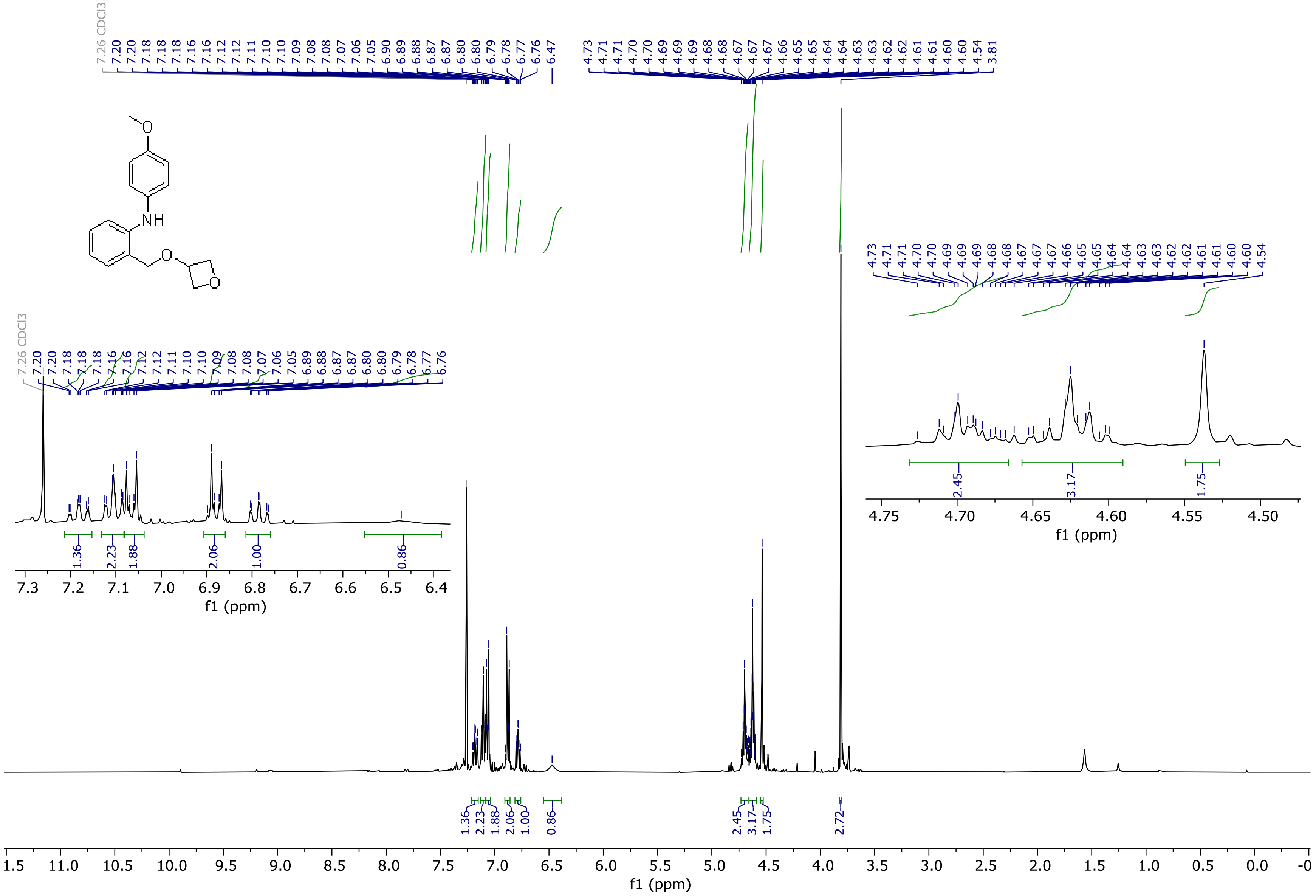

13C{1H}NMR (101 MHz, CDCl3): N-(4-Methoxyphenyl)-2-((oxetan-3-yloxy)methyl)aniline (1t)

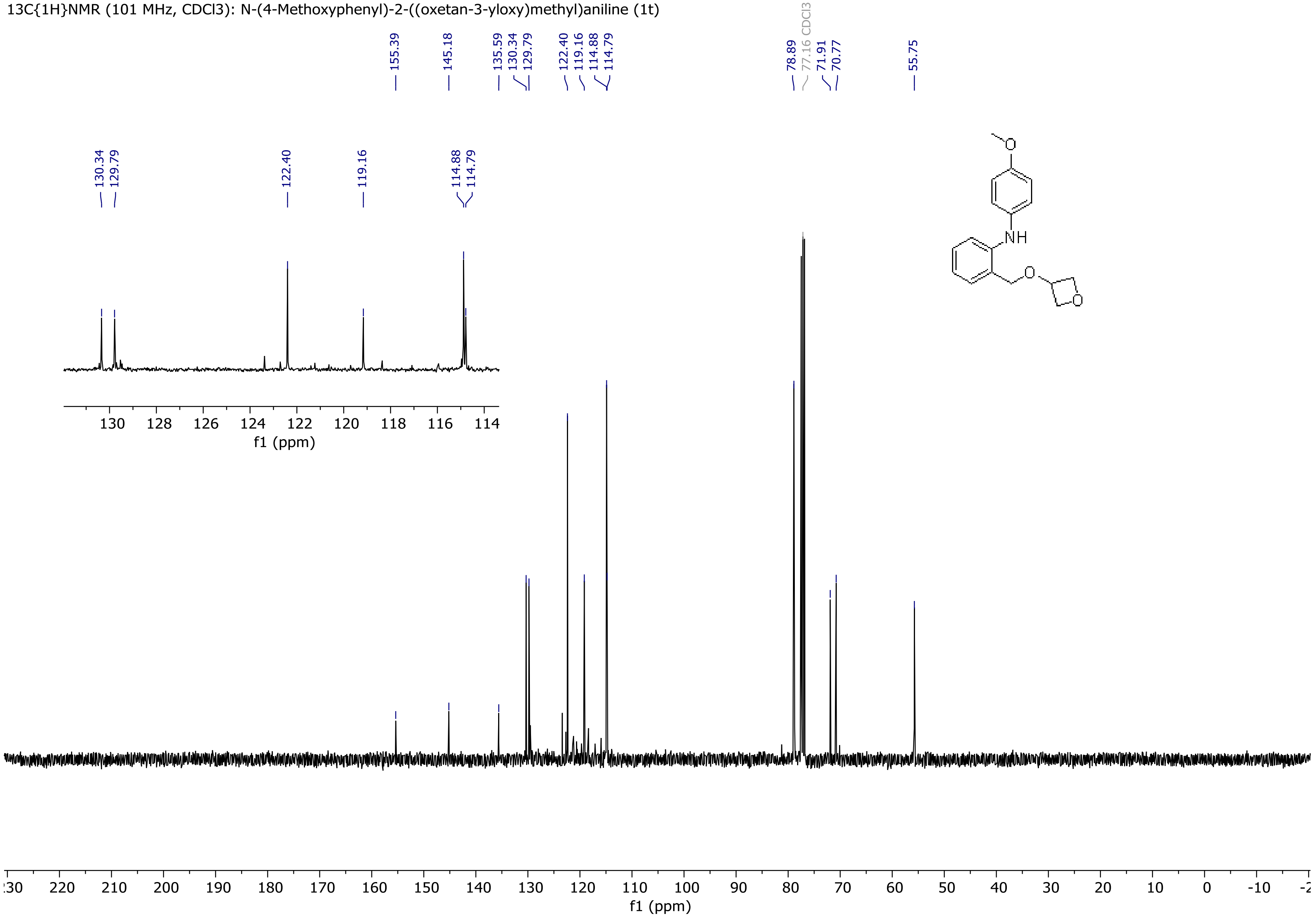

1H NMR (400 MHz, CDCl3): N-(4-bromophenyl)-2-((oxetan-3-yloxy)methyl)aniline (1u)

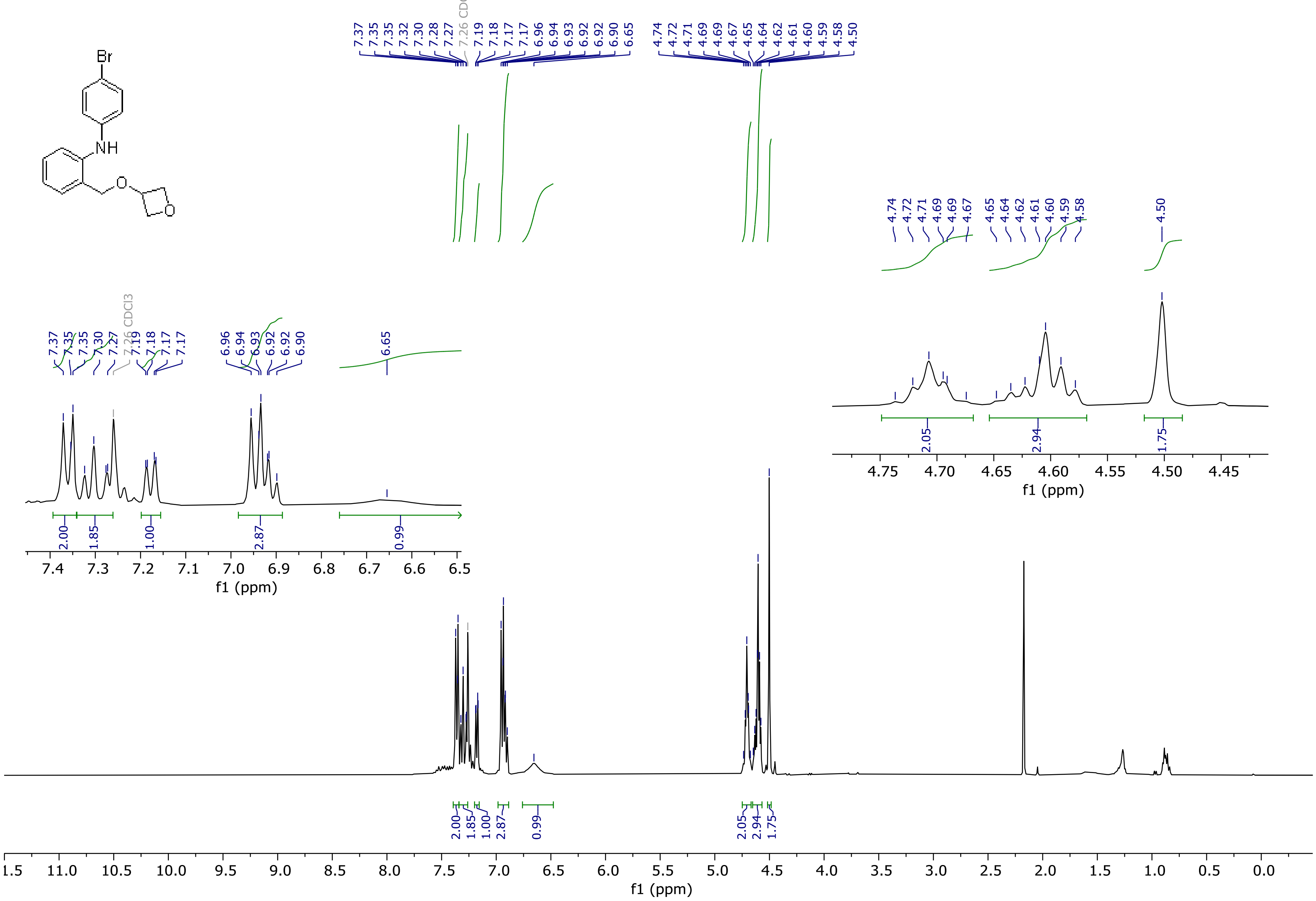

13C{1H}NMR (101 MHz, CDCl3): N-(4-bromophenyl)-2-((oxetan-3-yloxy)methyl)aniline (1u)

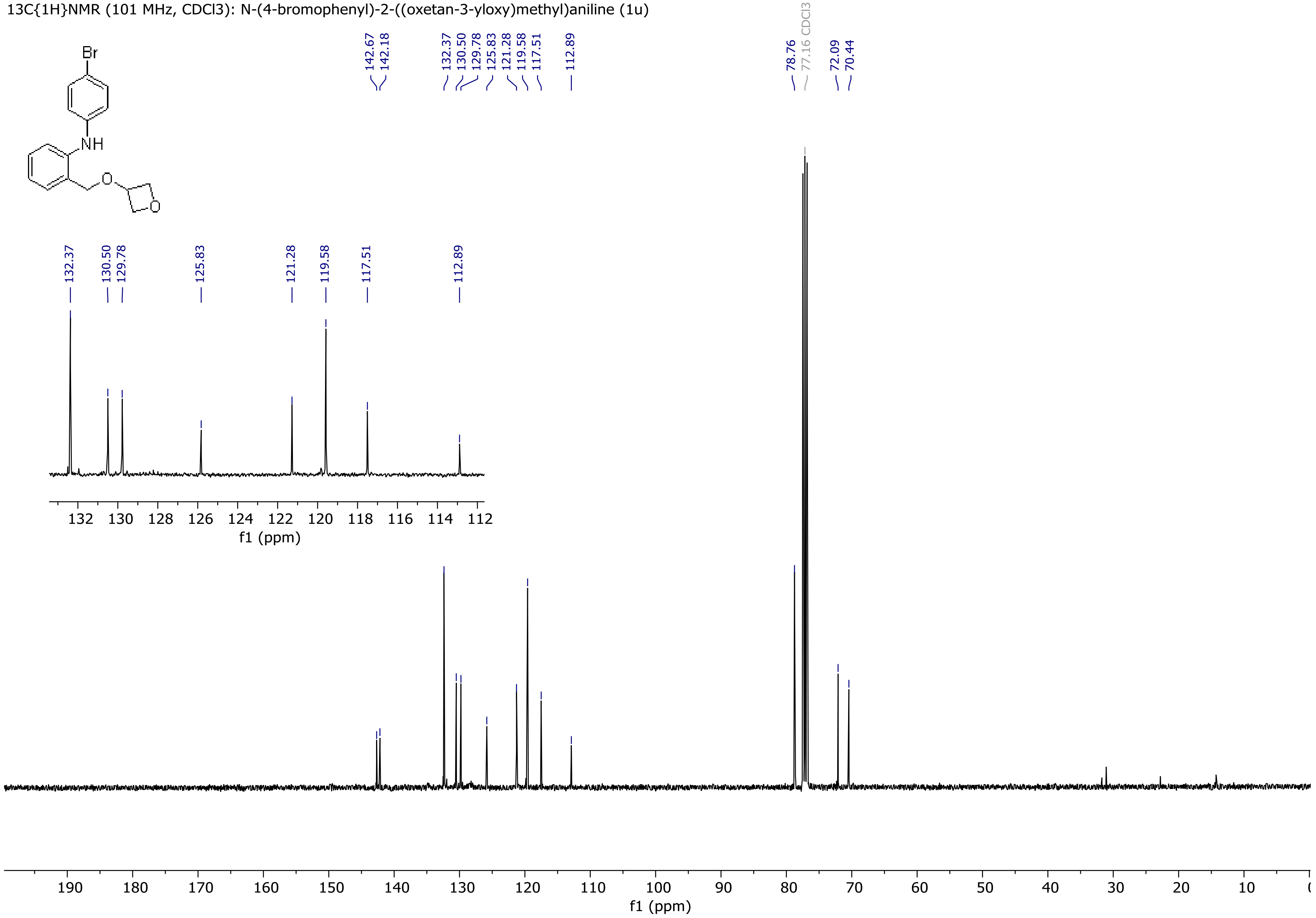

1H NMR: (400 MHz, CDCl3): (R)-(1-Benzyl-1,2,3,5-tetrahydrobenzo[e][1,4]oxazepin-3-yl)methanol (2a)

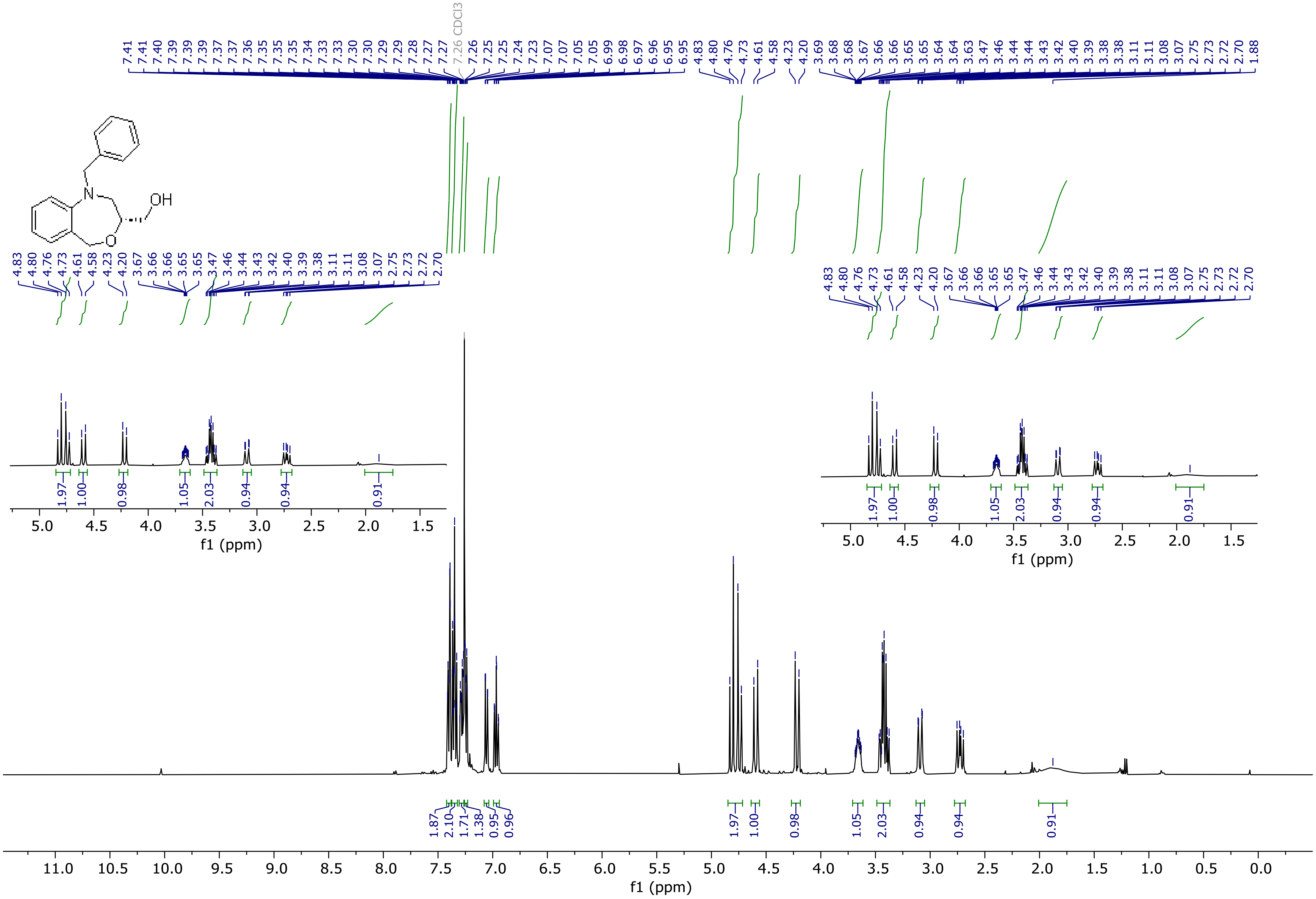

13C{1H}NMR (101 MHz, CDCl3): (R)-(1-Benzyl-1,2,3,5-tetrahydrobenzo[e][1,4]oxazepin-3-yl)methanol (2a)

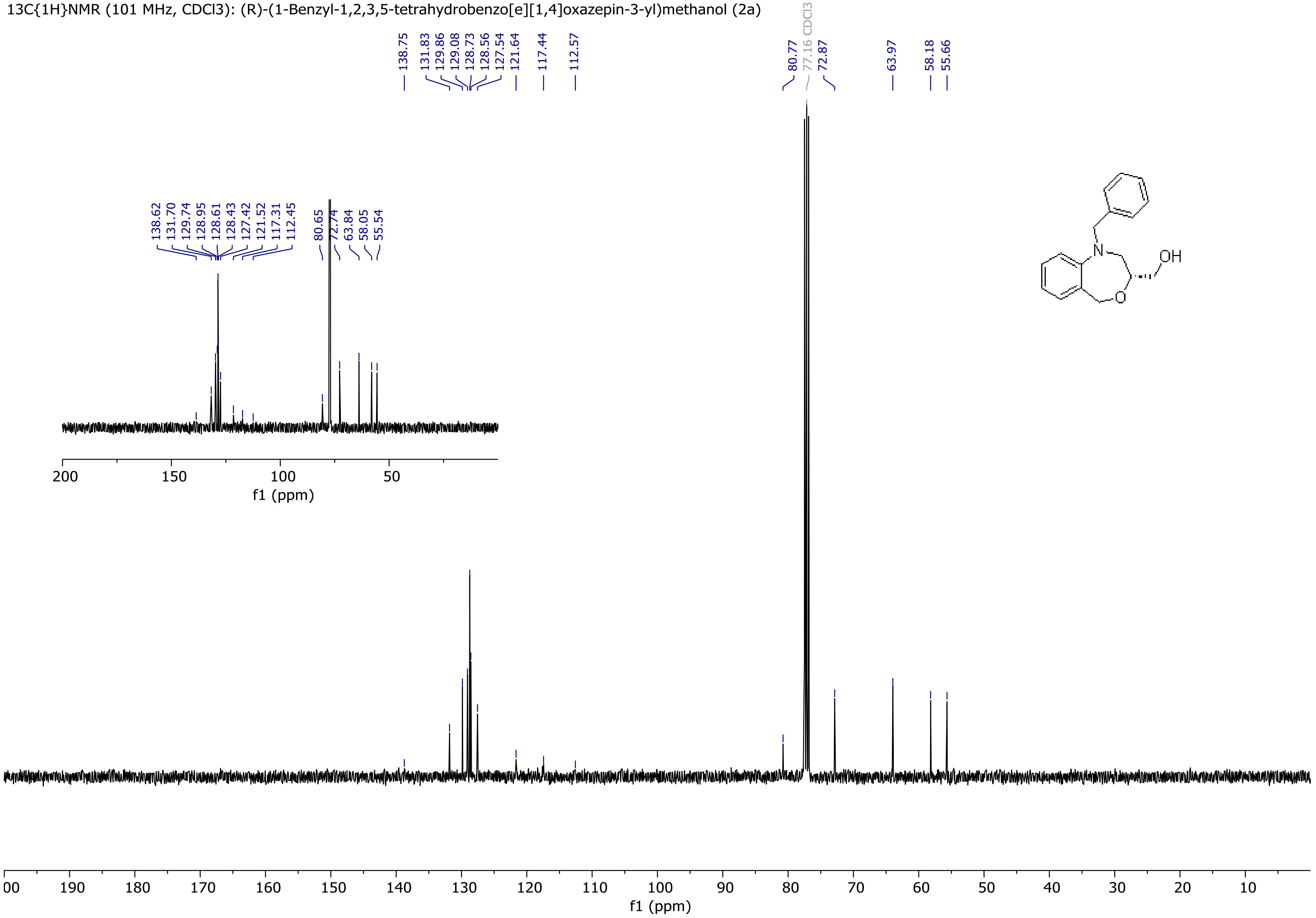

1H NMR: (400 MHz, CDCl3): (R)-(1-(4-Methoxybenzyl)-1,2,3,5-tetrahydrobenzo[e][1,4]oxazepin-3-yl)methanol (2b)

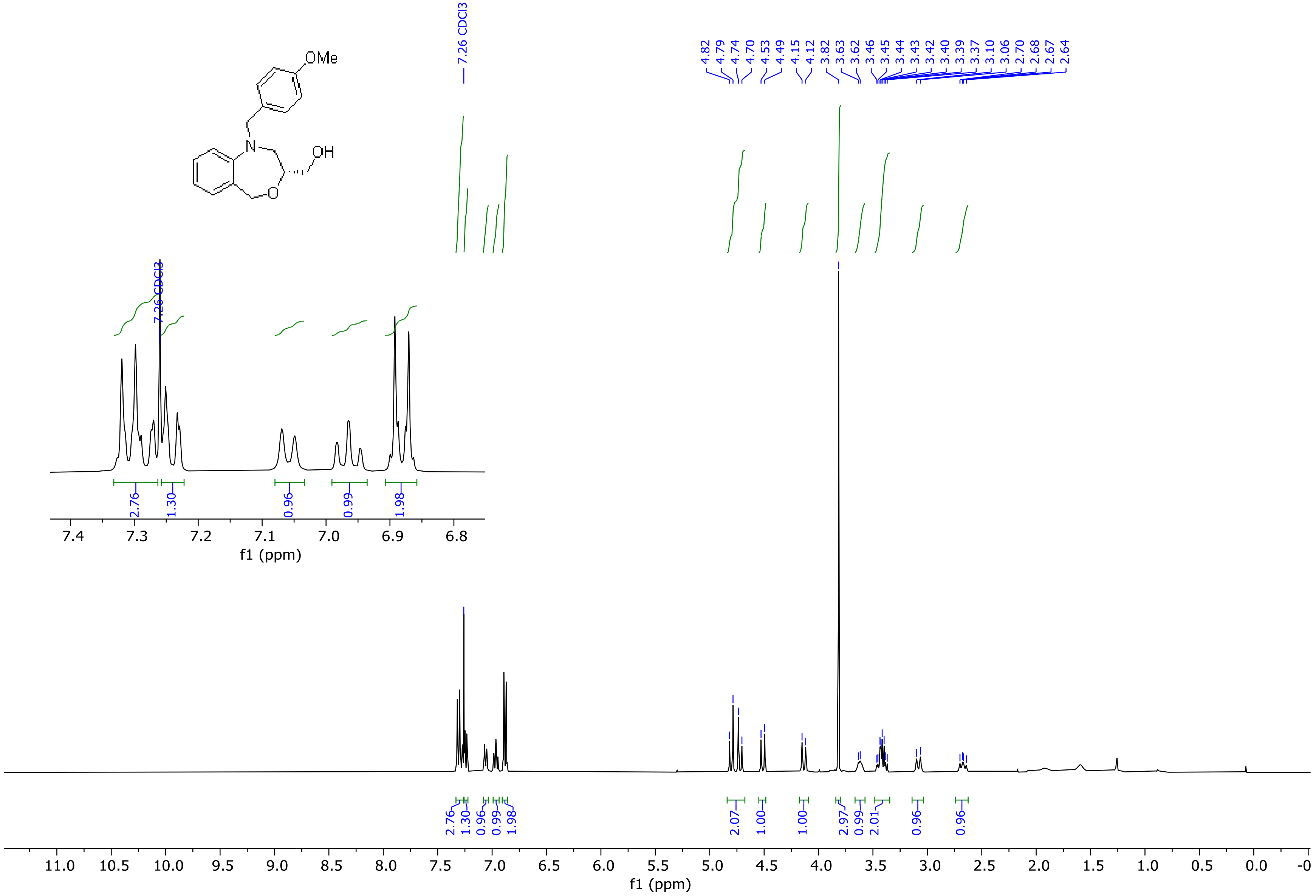

13C{1H}NMR (101 MHz, CDCl3): (R)-(1-(4-Methoxybenzyl)-1,2,3,5-tetrahydrobenzo[e][1,4]oxazepin-3-yl)methanol (2b)

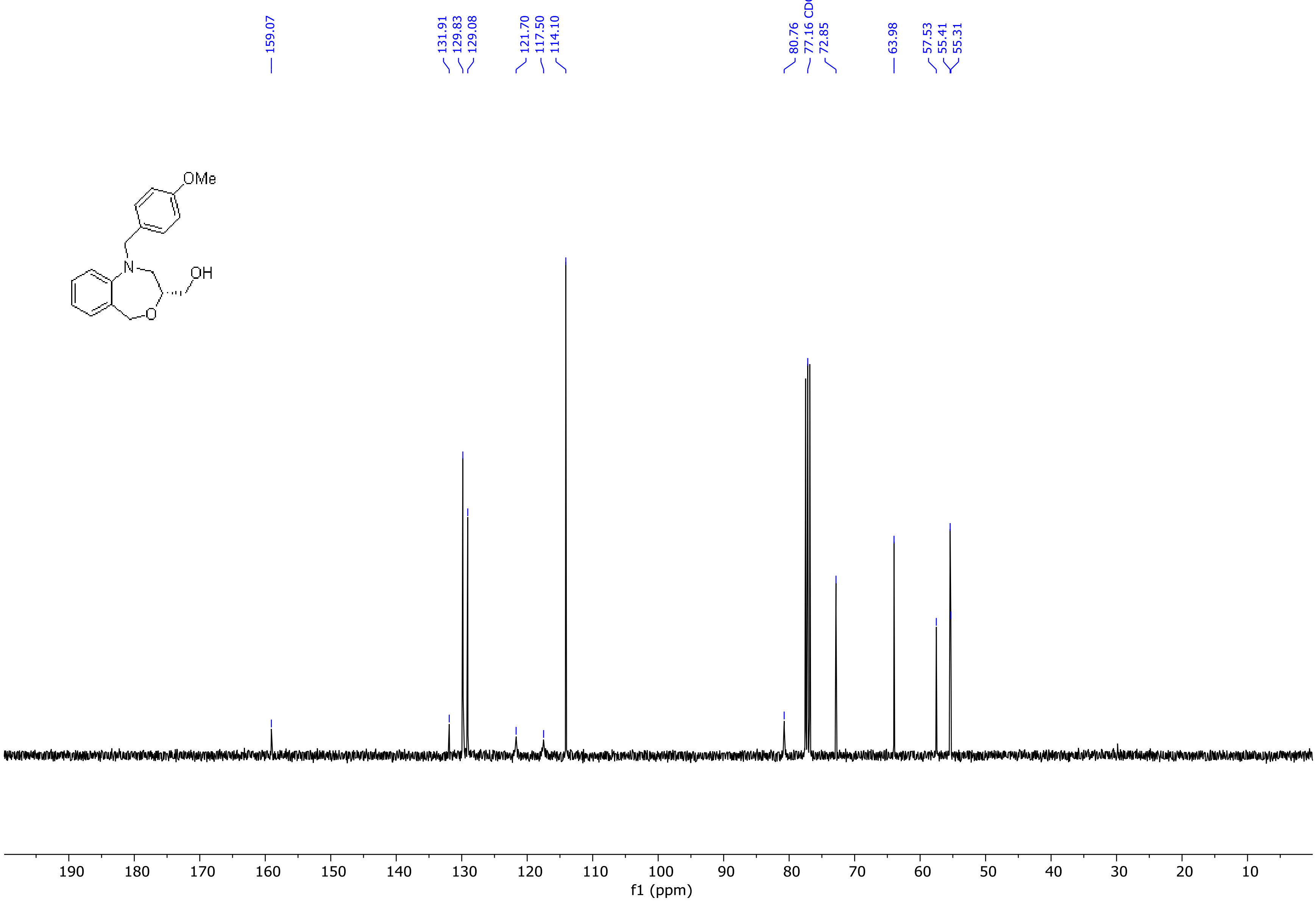

1H NMR: (400 MHz, CDCl3): (R)-(1-(4-Methylbenzyl)-1,2,3,5-tetrahydrobenzo[e][1,4]oxazepin-3-yl)methanol (2c)

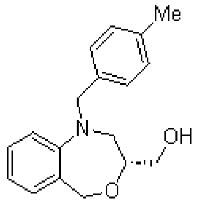

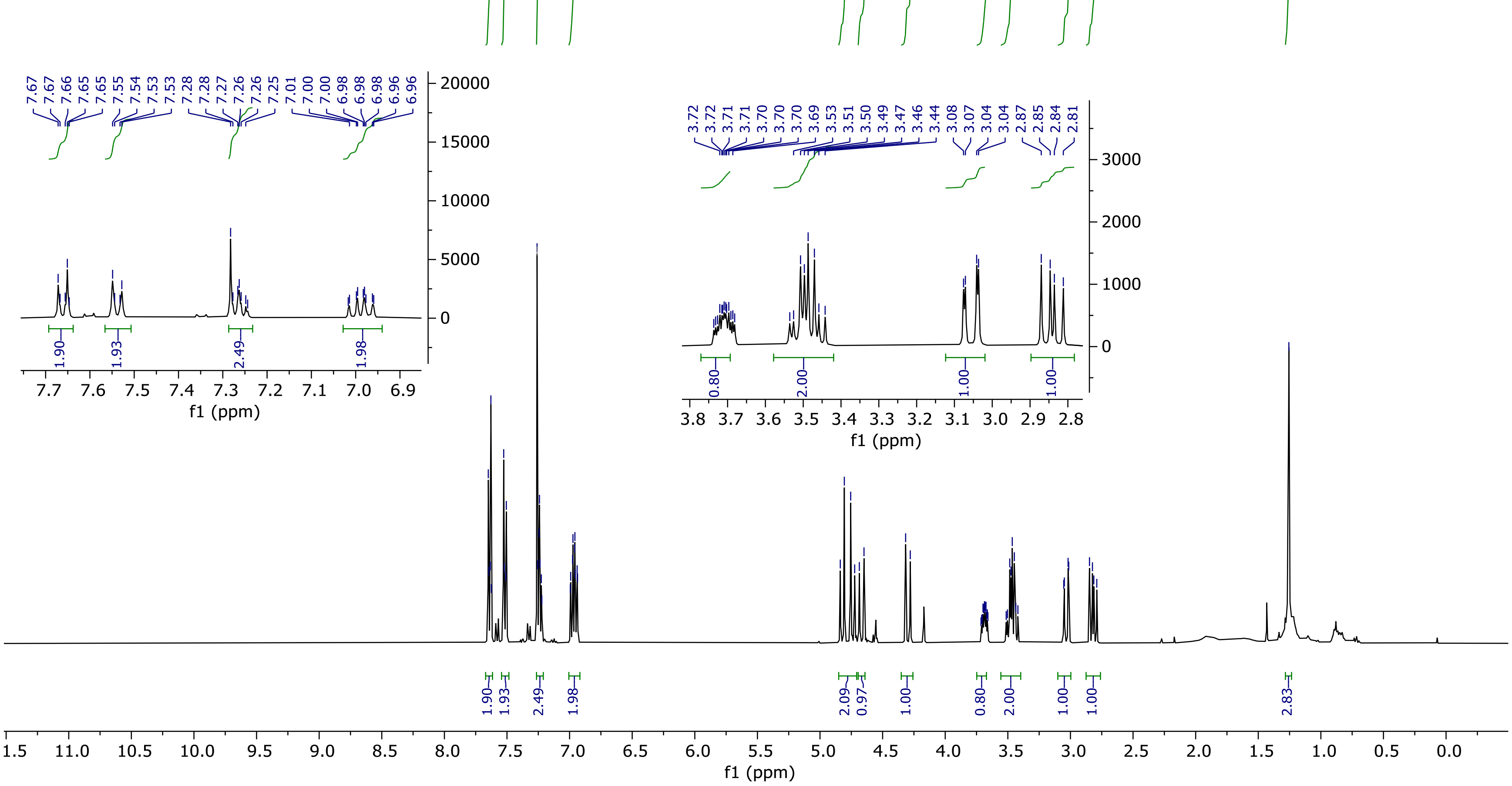

13C{1H}NMR (101 MHz, CDCl3): (R)-(1-(4-Methylbenzyl)-1,2,3,5-tetrahydrobenzo[e][1,4]oxazepin-3-yl)methanol (2c)

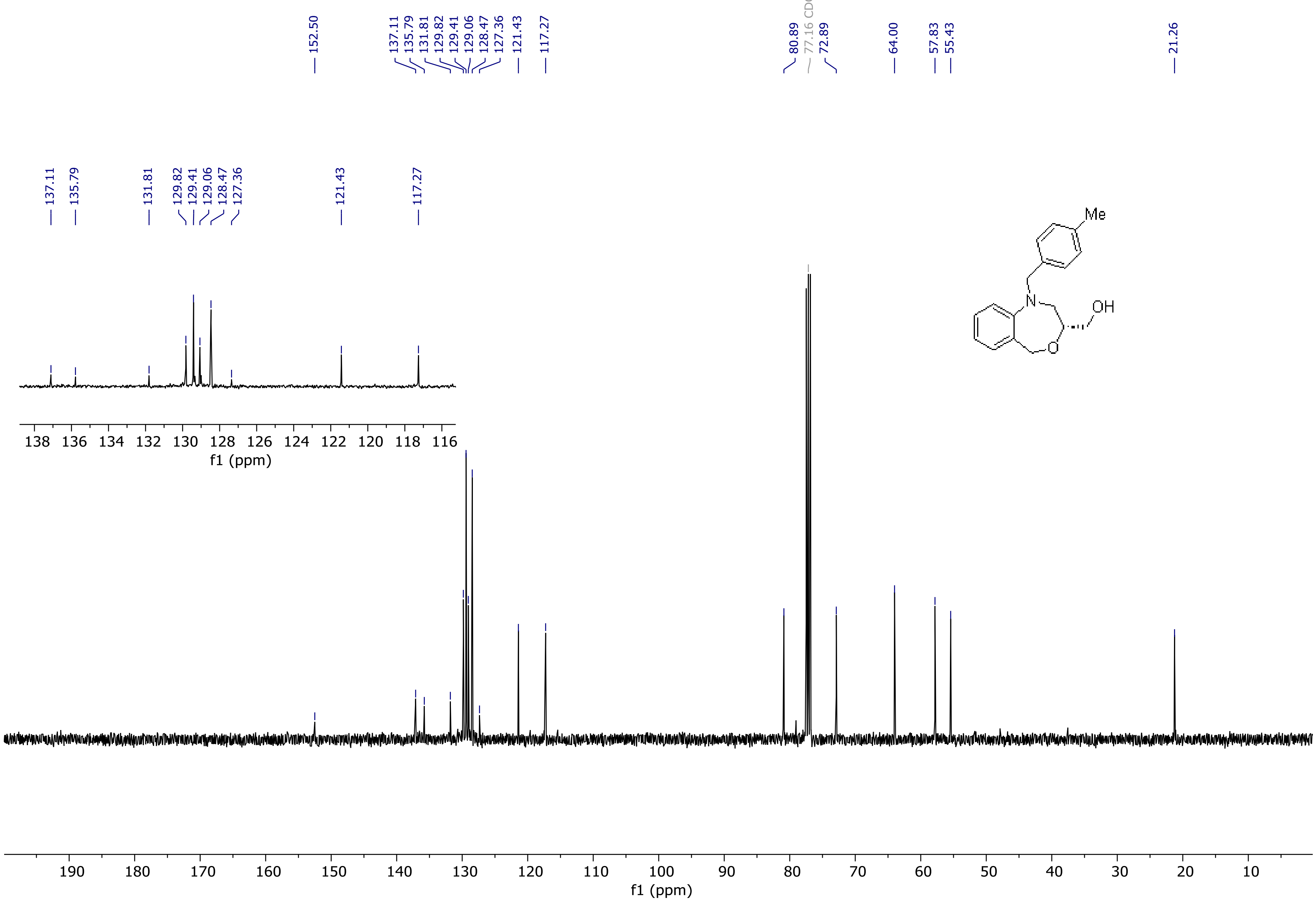

1H NMR: (400 MHz, CDCl3): (R)-(1-(4-Nitrobenzyl)-1,2,3,5-tetrahydrobenzo[e][1,4]oxazepin-3-yl)methanol (2d)

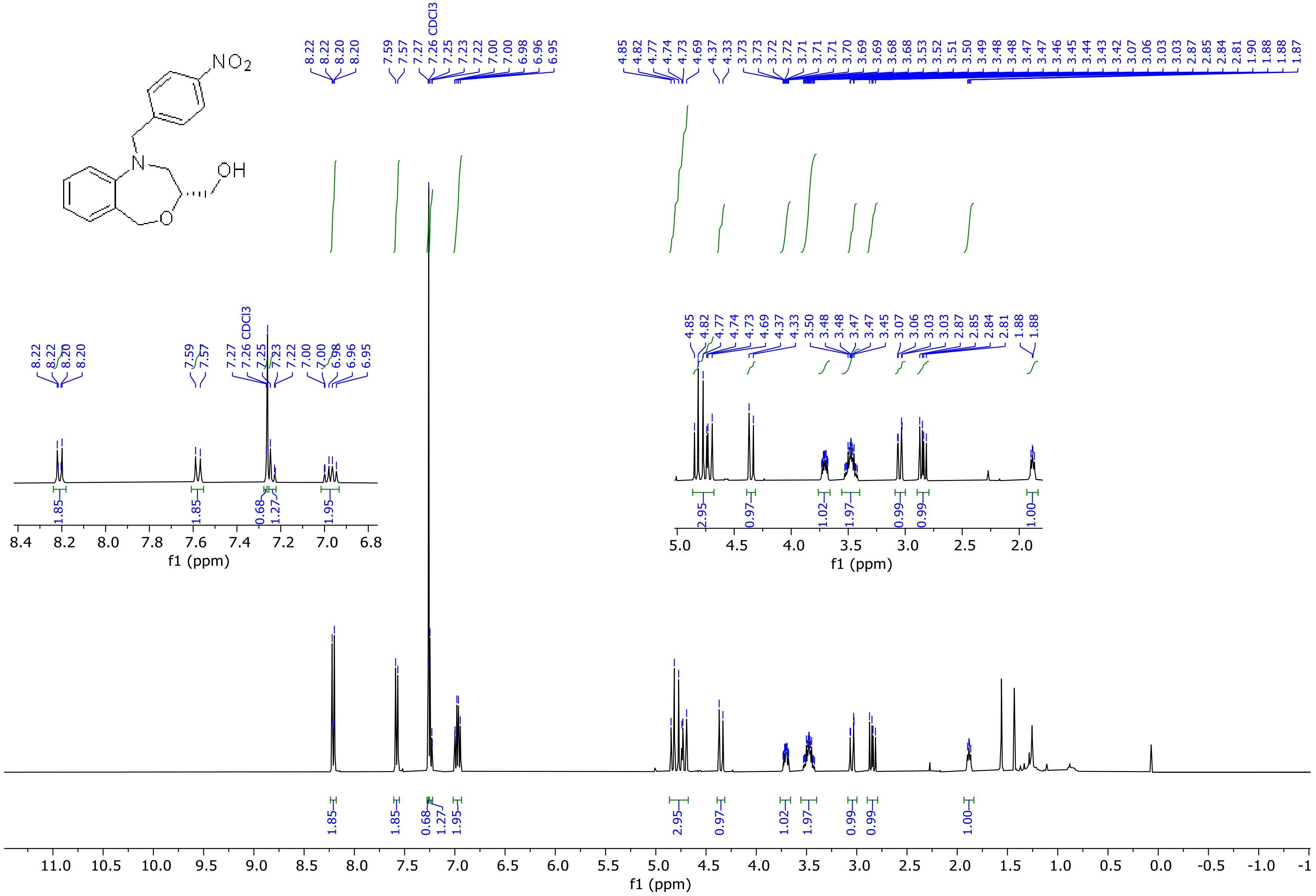

13C{1H}NMR (101 MHz, CDCl3): (R)-(1-(4-Nitrobenzyl)-1,2,3,5-tetrahydrobenzo[e][1,4]oxazepin-3-yl)methanol (9d)

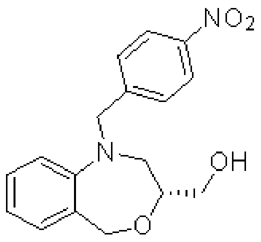

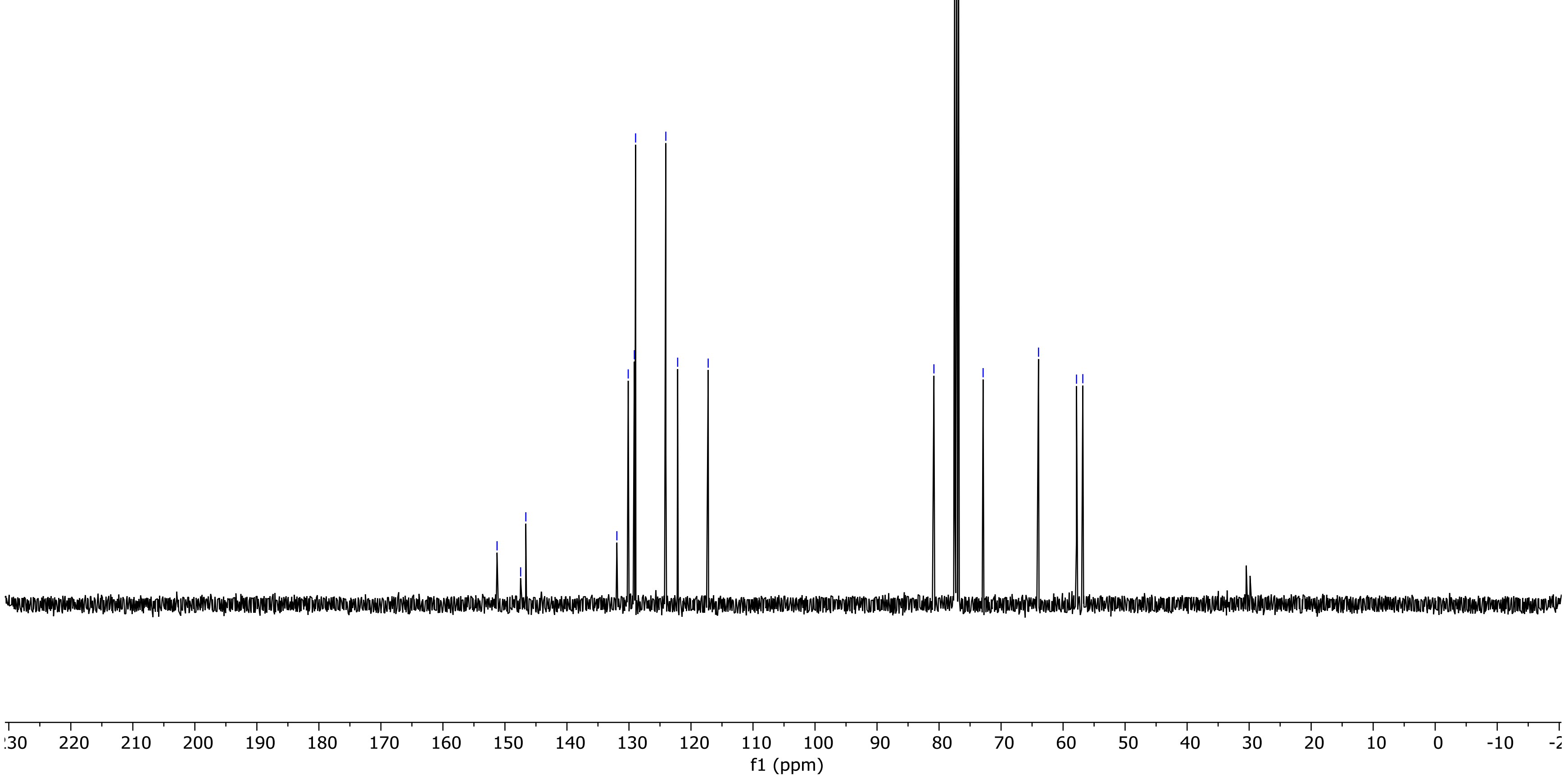

1H NMR: (400 MHz, CDCl3): (R)-4-((3-(Hydroxymethyl)-2,3-dihydrobenzo[e][1,4]oxazepin-1(5H)-yl)methyl)benzonitrile (2e)

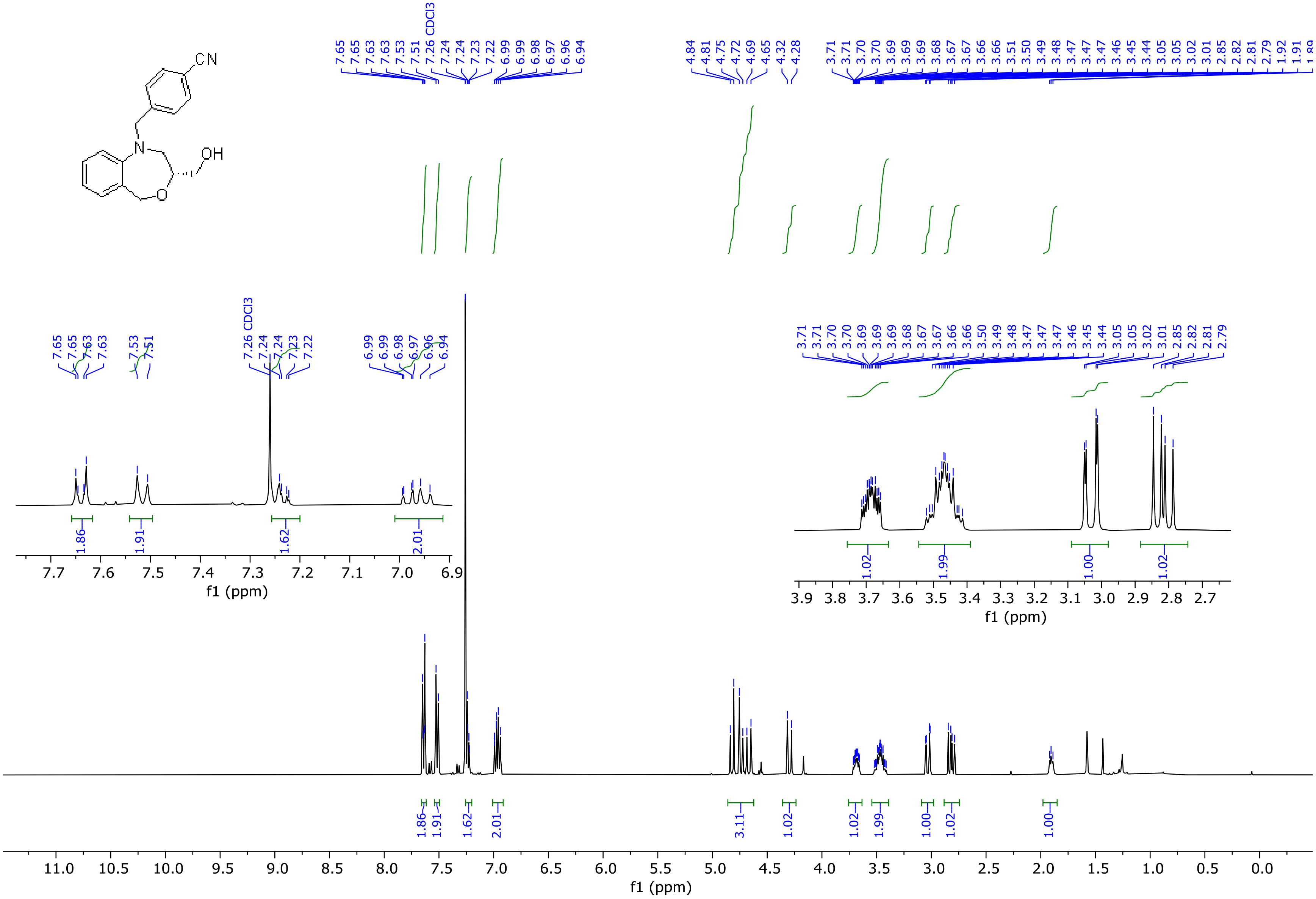

13C{1H}NMR (101 MHz, CDCl3): (R)-4-((3-(Hydroxymethyl)-2,3-dihydrobenzo[e][1,4]oxazepin-1(5H)-yl)methyl)benzonitrile (2e)

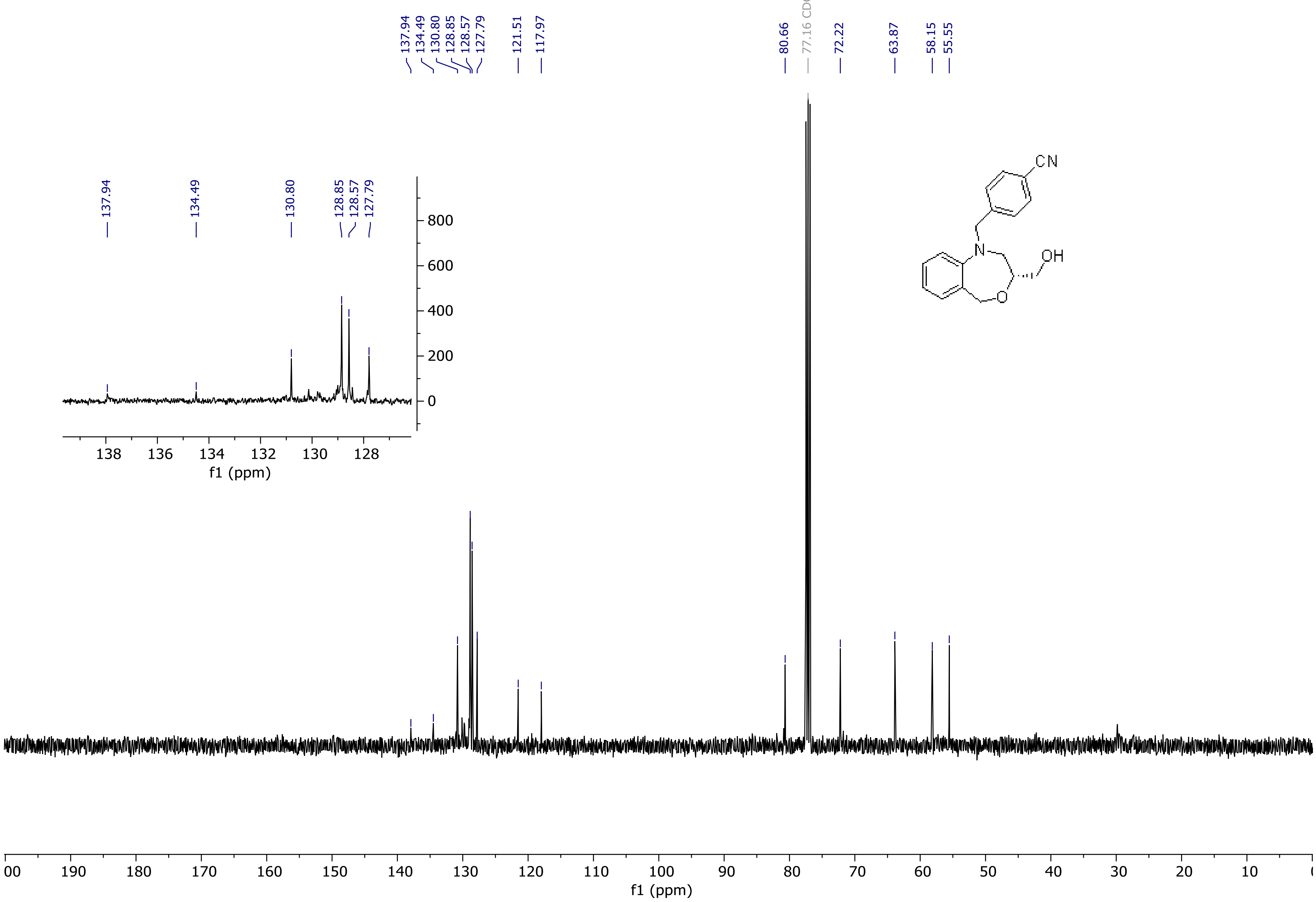

1H NMR: (400 MHz, CDCl3): (R)-(1-(4-(Trifluoromethyl)benzyl)-1,2,3,5-tetrahydrobenzo[e][1,4]oxazepin-3-yl)methanol (2f)

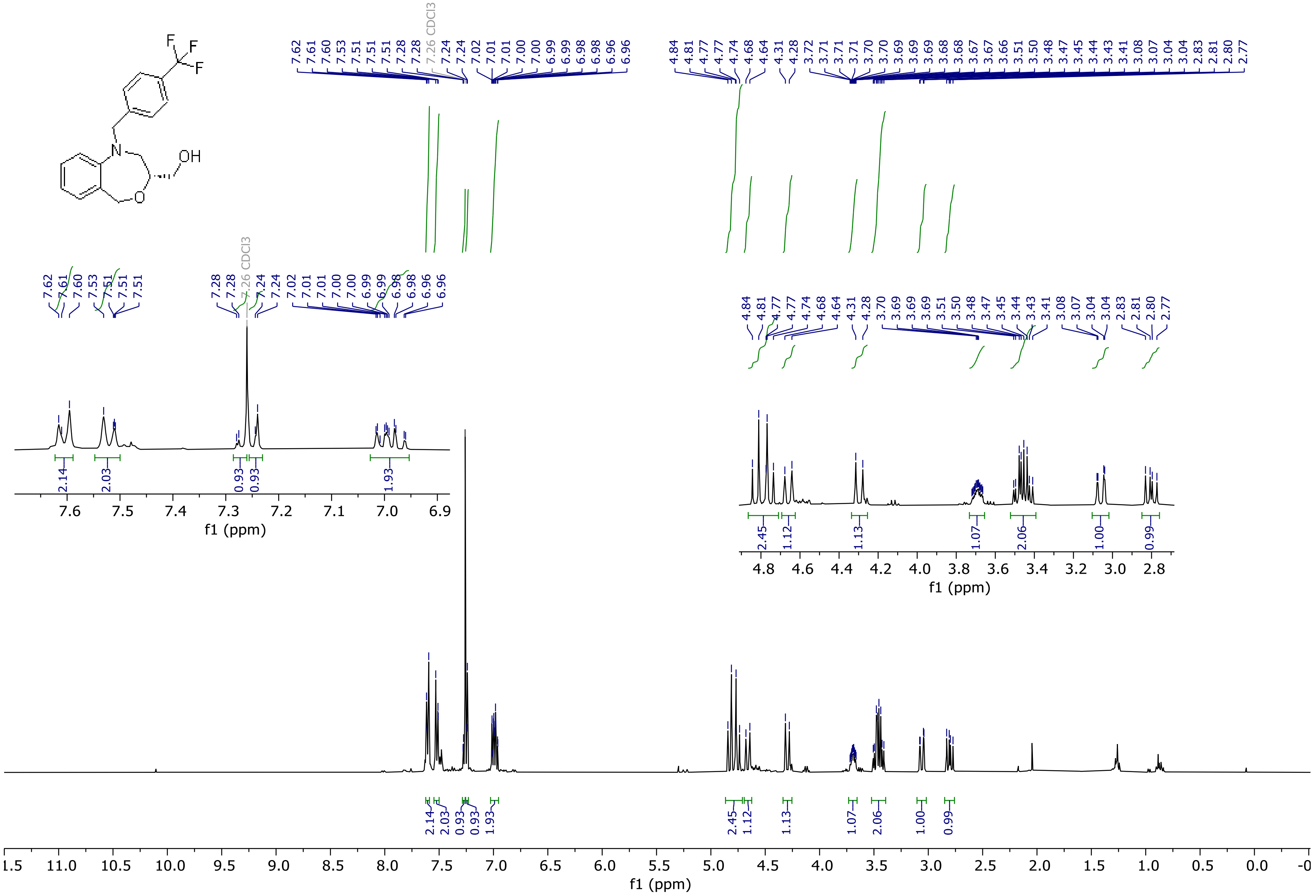

13C{1H}NMR (101 MHz, CDCl3): (R)-(1-(4-(Trifluoromethyl)benzyl)-1,2,3,5-tetrahydrobenzo[e][1,4]oxazepin-3-yl)methanol (2f)

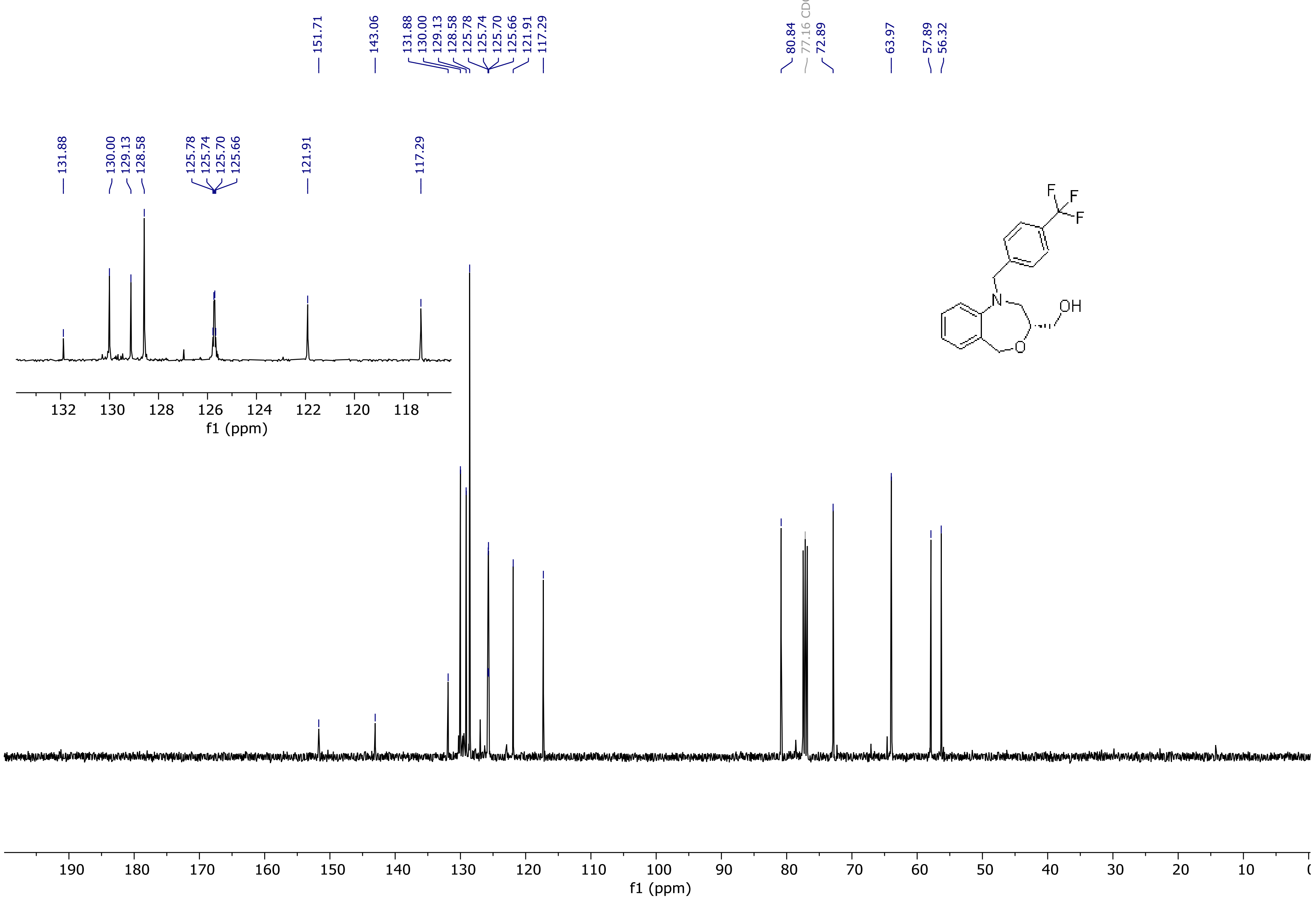

19F NMR (376 MHz, CDCl3): (R)-(1-(4-(Trifluoromethyl)benzyl)-1,2,3,5-tetrahydrobenzo[e][1,4]oxazepin-3-yl)methanol (2f)

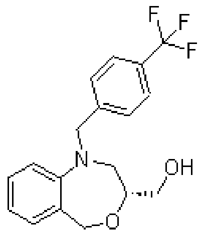

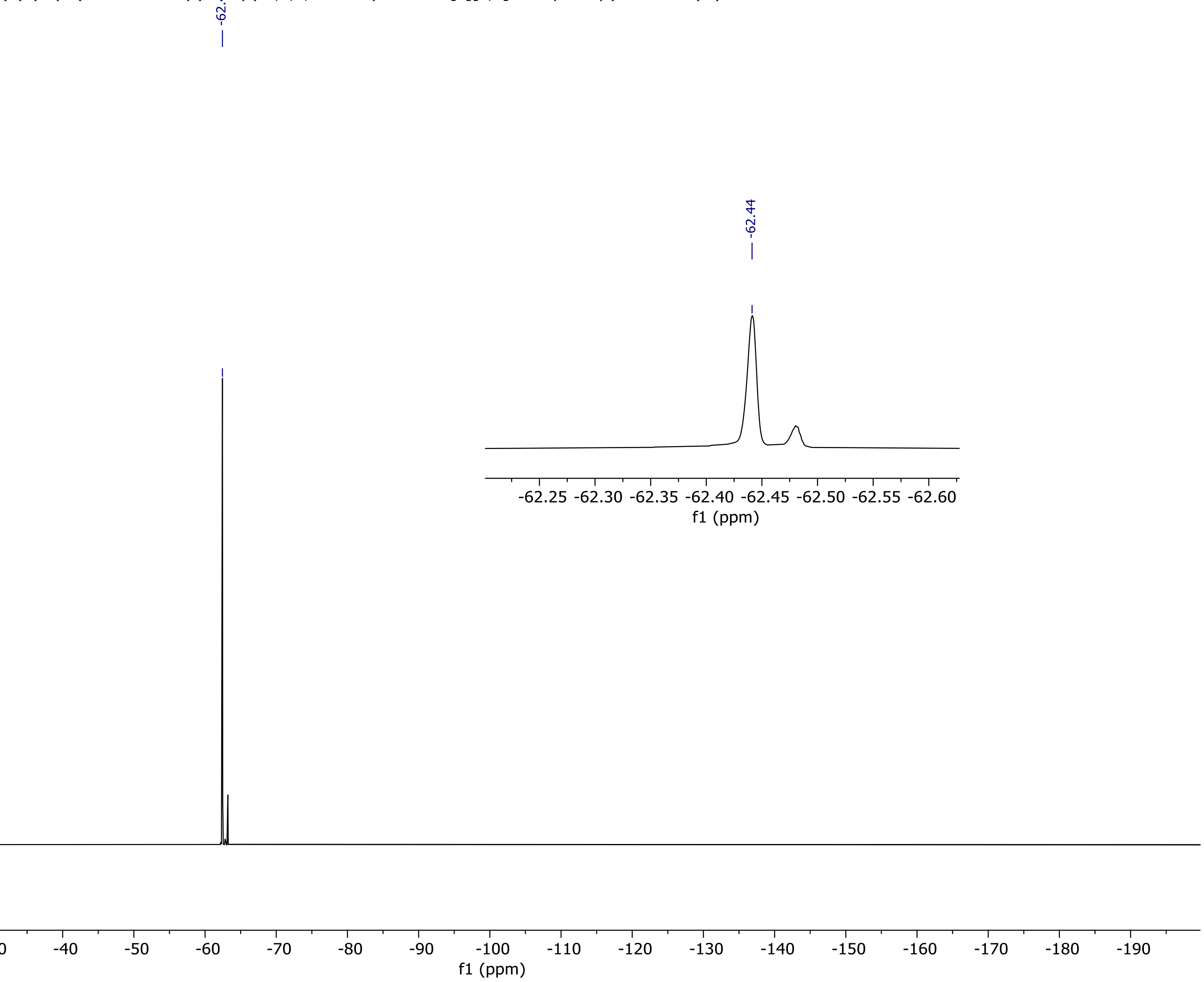

1H NMR: (400 MHz, CDCl3): (R)-(1-(4-Fluorobenzyl)-1,2,3,5-tetrahydrobenzo[e][1,4]oxazepin-3-yl)methanol (2g)

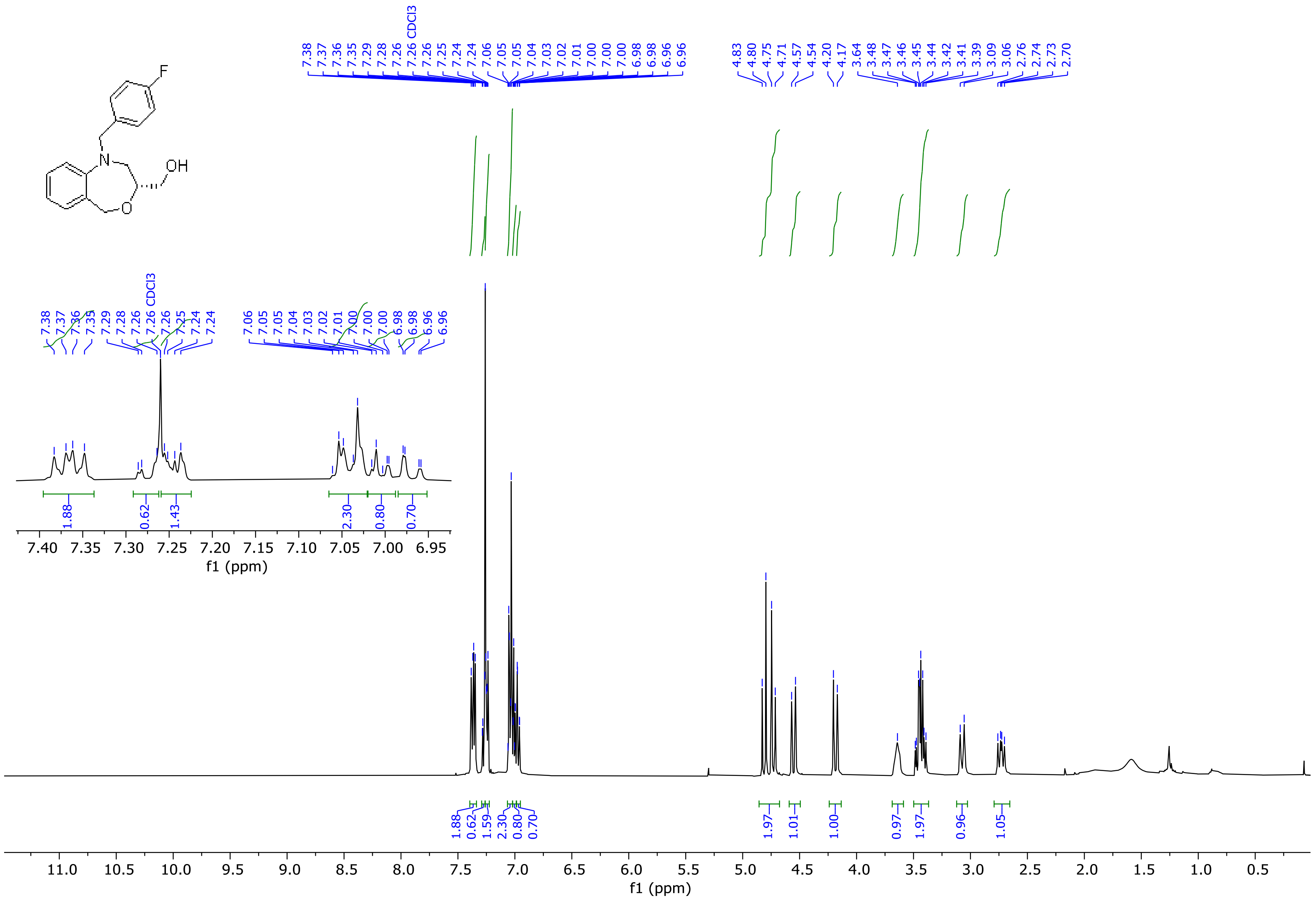

13C{1H}NMR (101 MHz, CDCl3): (R)-(1-(4-Fluorobenzyl)-1,2,3,5-tetrahydrobenzo[e][1,4]oxazepin-3-yl)methanol (2g)

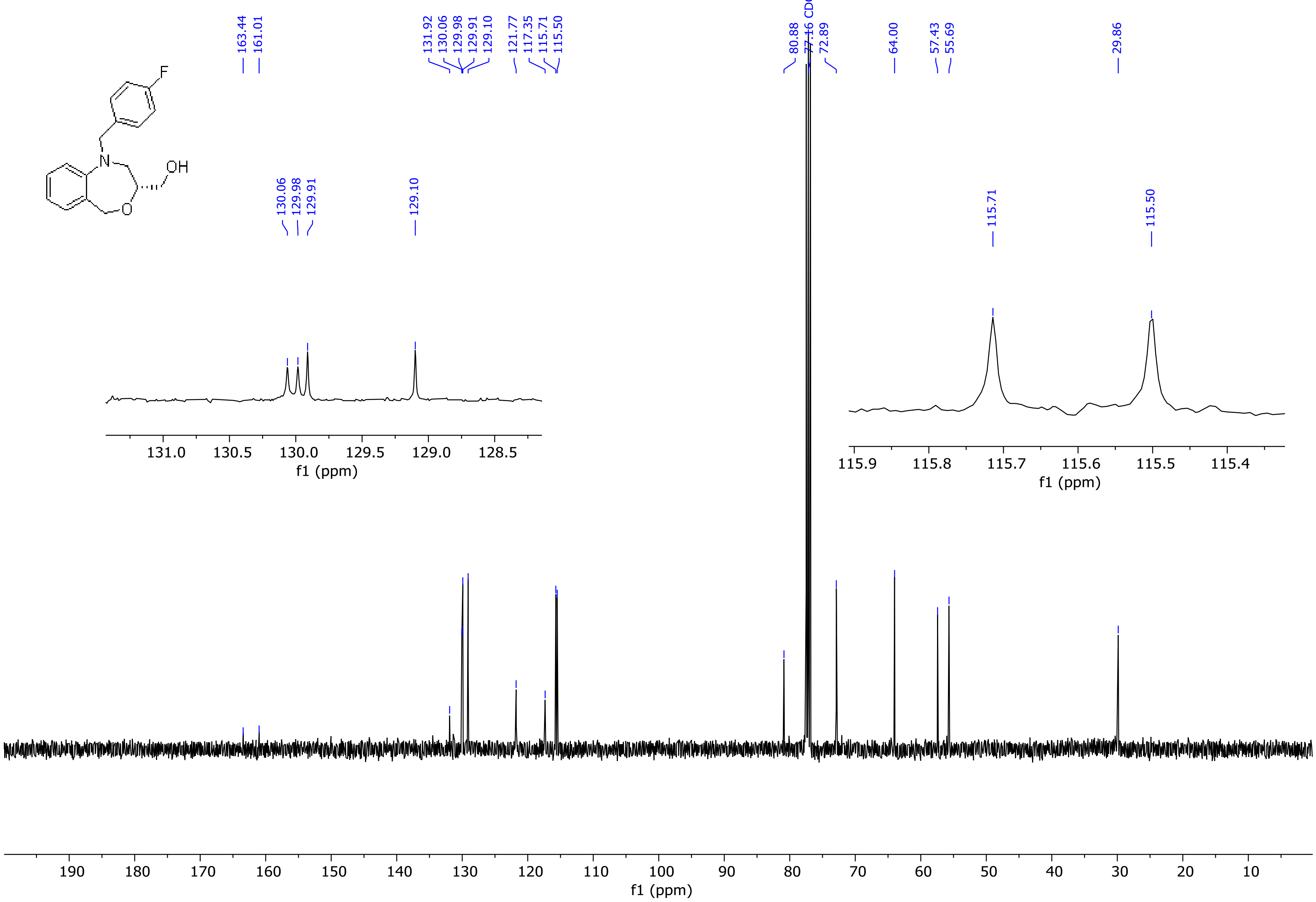

19F NMR (376 MHz, CDCl3): (R)-(1-(4-Fluorobenzyl)-1,2,3,5-tetrahydrobenzo[e][1,4]oxazepin-3-yl)methanol (2g)

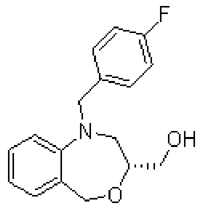

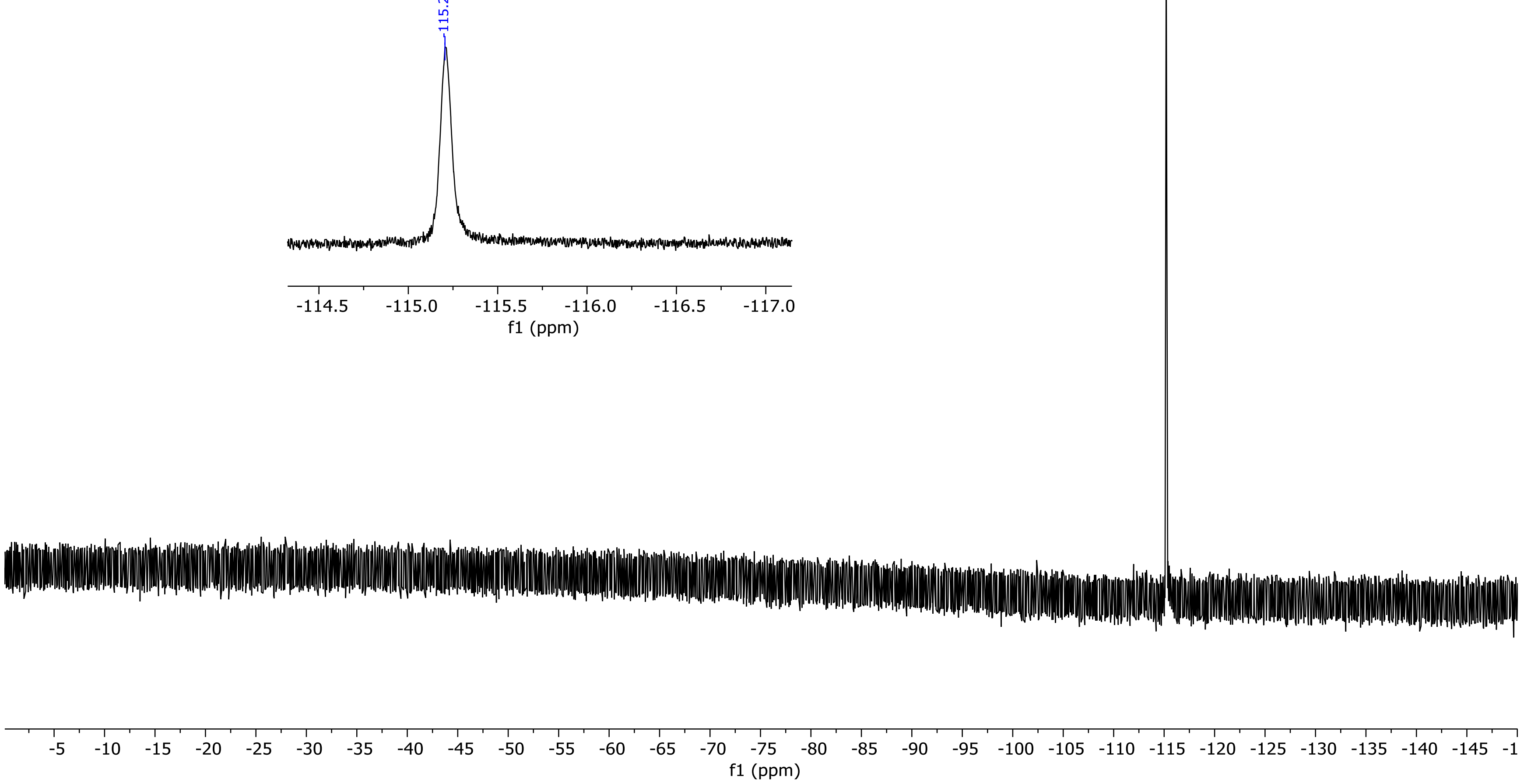

1H NMR: (400 MHz, CDCl3): (R)-(1-(4-Bromobenzyl)-1,2,3,5-tetrahydrobenzo[e][1,4]oxazepin-3-yl)methanol (2h)

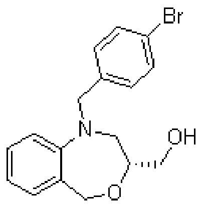

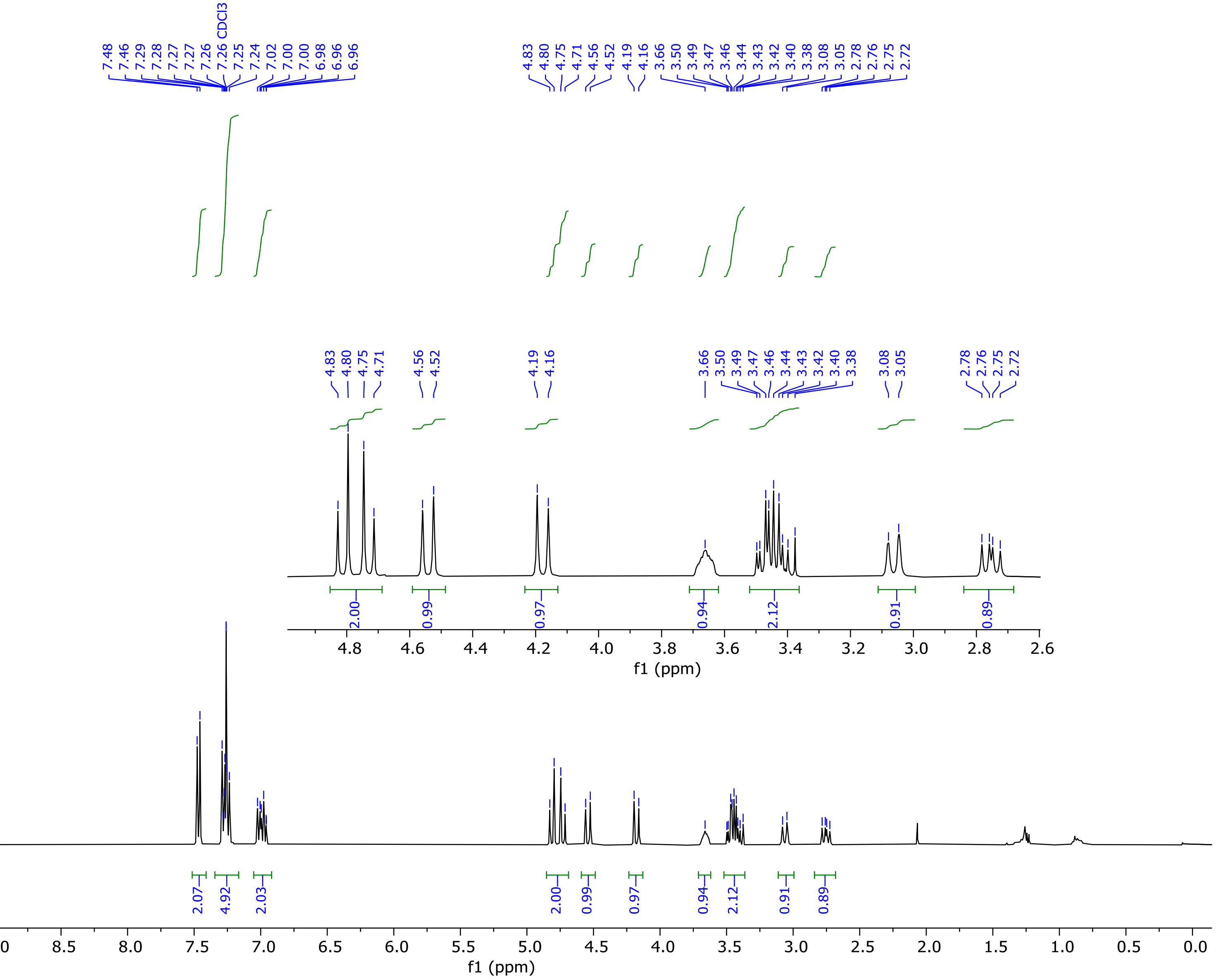

13C{1H}NMR (101 MHz, CDCl3): (R)-(1-(4-Bromobenzyl)-1,2,3,5-tetrahydrobenzo[e][1,4]oxazepin-3-yl)methanol (2h)

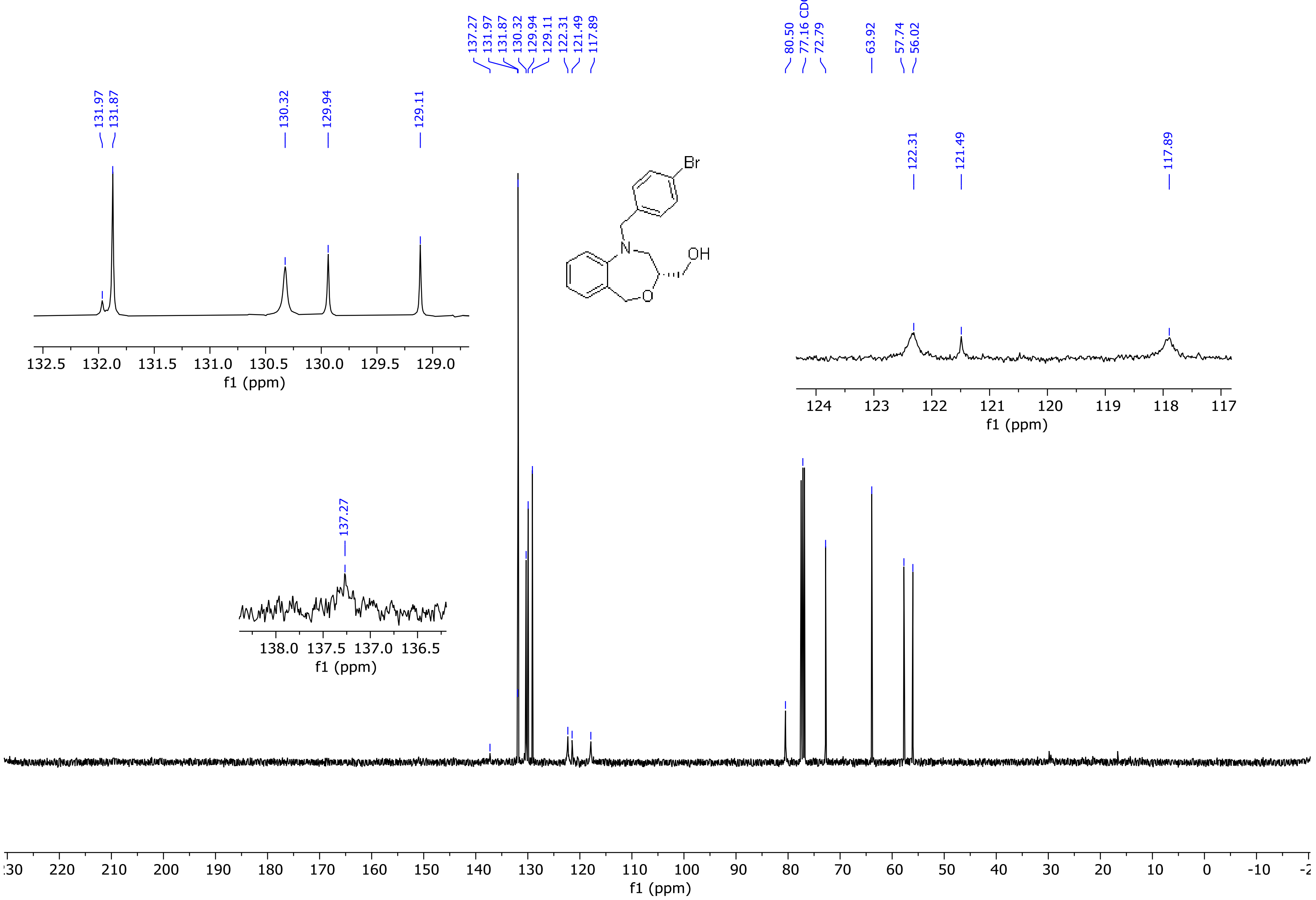

1H NMR: (400 MHz, CDCl3): (R)-(1-(3-Bromobenzyl)-1,2,3,5-tetrahydrobenzo[e][1,4]oxazepin-3-yl)methanol (2i)

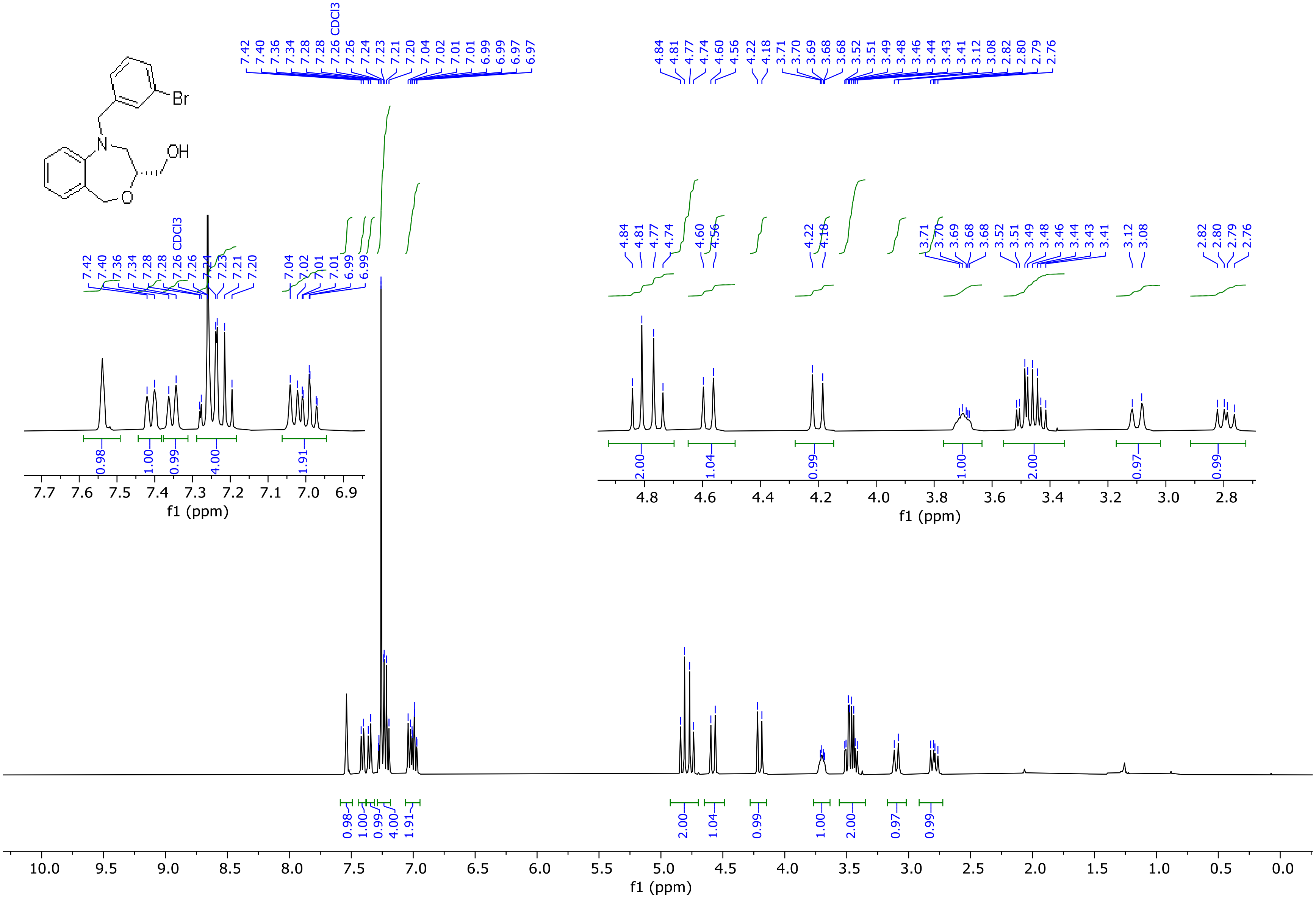

13C{1H}NMR (101 MHz, CDCl3): (R)-(1-(3-Bromobenzyl)-1,2,3,5-tetrahydrobenzo[e][1,4]oxazepin-3-yl)methanol (2i)

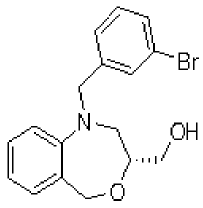

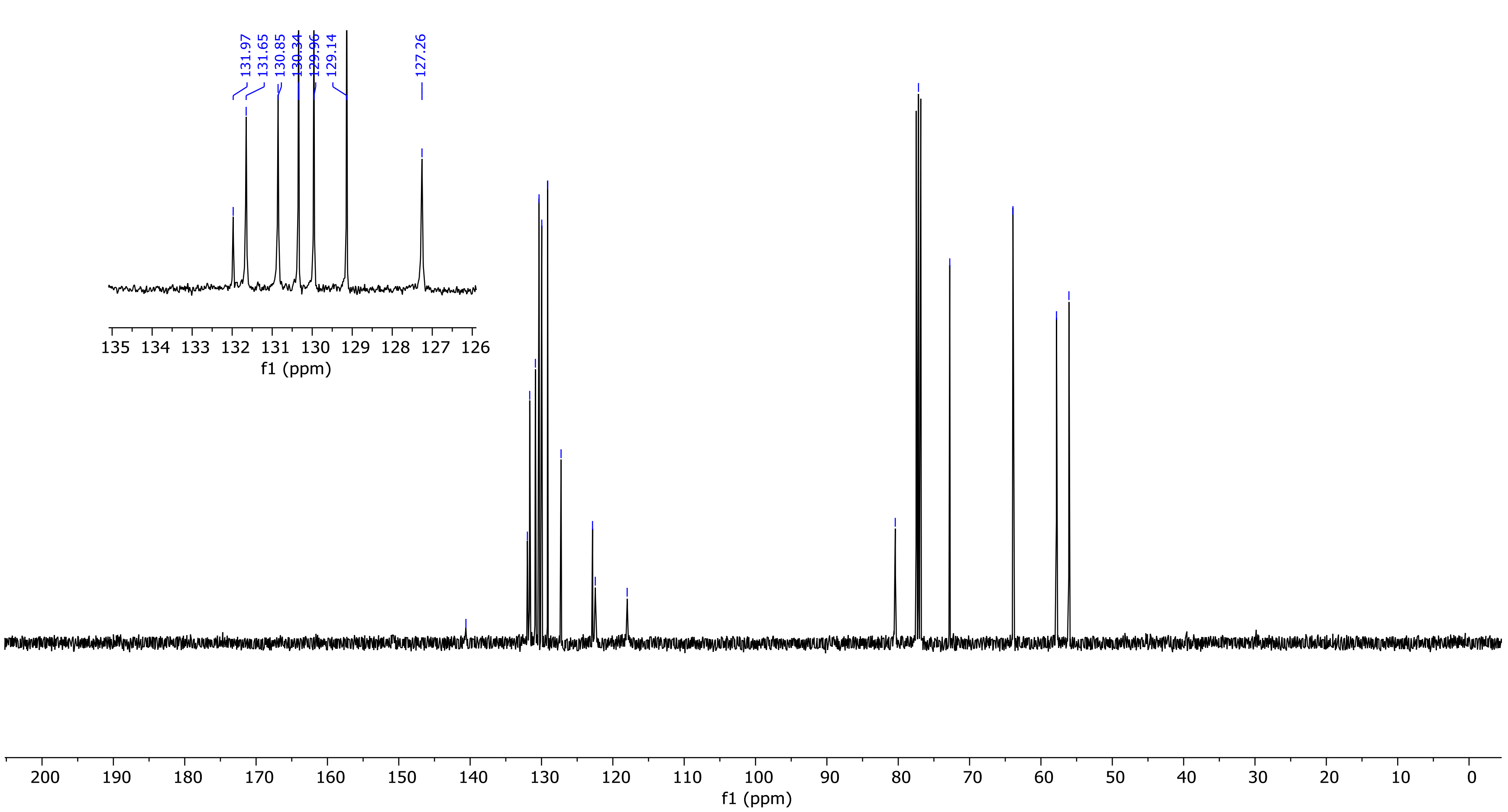

1H NMR: (400 MHz, CDCl3): (R)-(1-(2-Bromobenzyl)-1,2,3,5-tetrahydrobenzo[e][1,4]oxazepin-3-yl)methanol (2j)

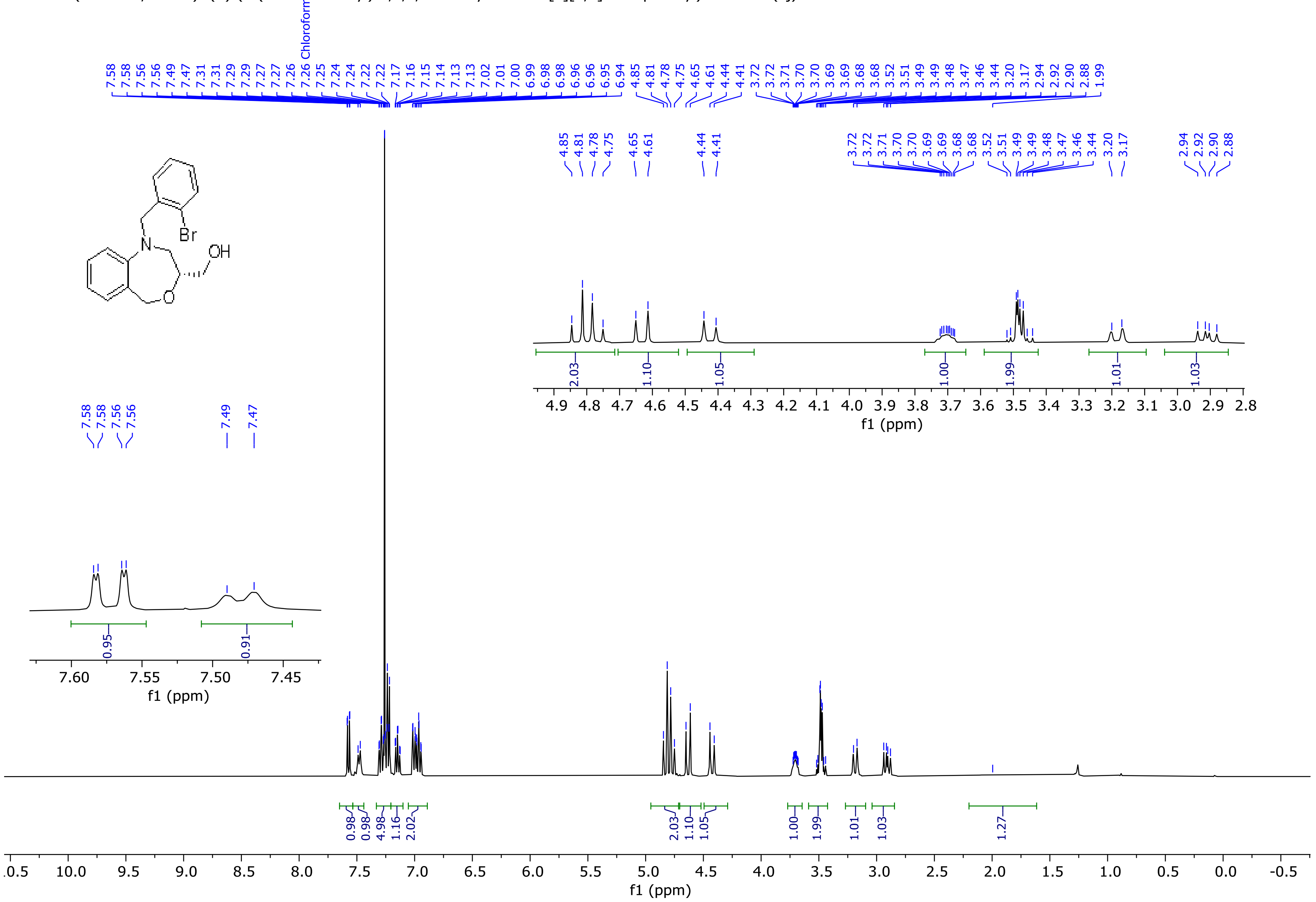

13C{1H}NMR (101 MHz, CDCl3): (R)-(1-(2-Bromobenzyl)-1,2,3,5-tetrahydrobenzo[e][1,4]oxazepin-3-yl)methanol (2f)

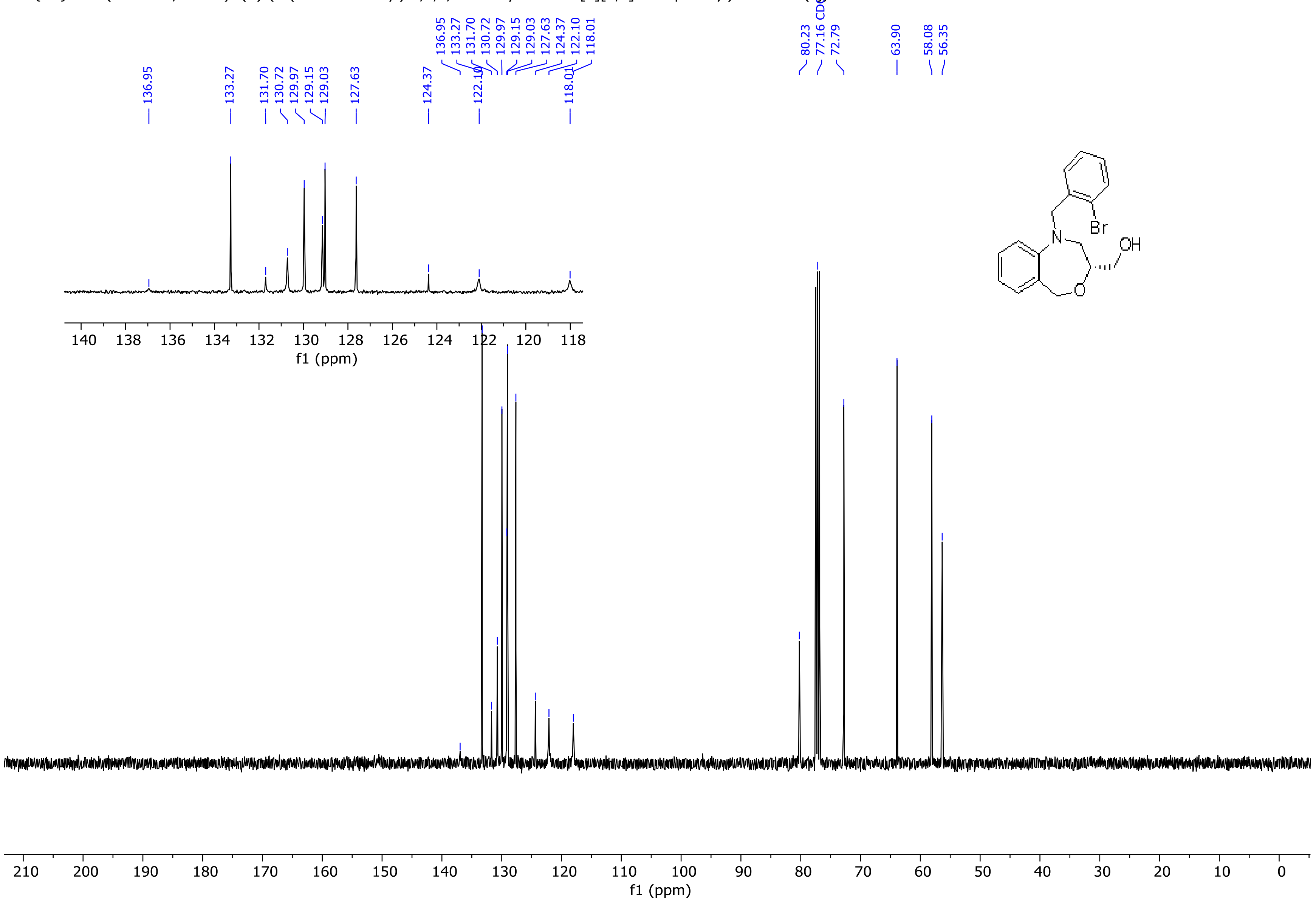

1H NMR: (400 MHz, CDCl3): (R)-(1-(2-Nitrobenzyl)-1,2,3,5-tetrahydrobenzo[e][1,4]oxazepin-3-yl)methanol (2k)

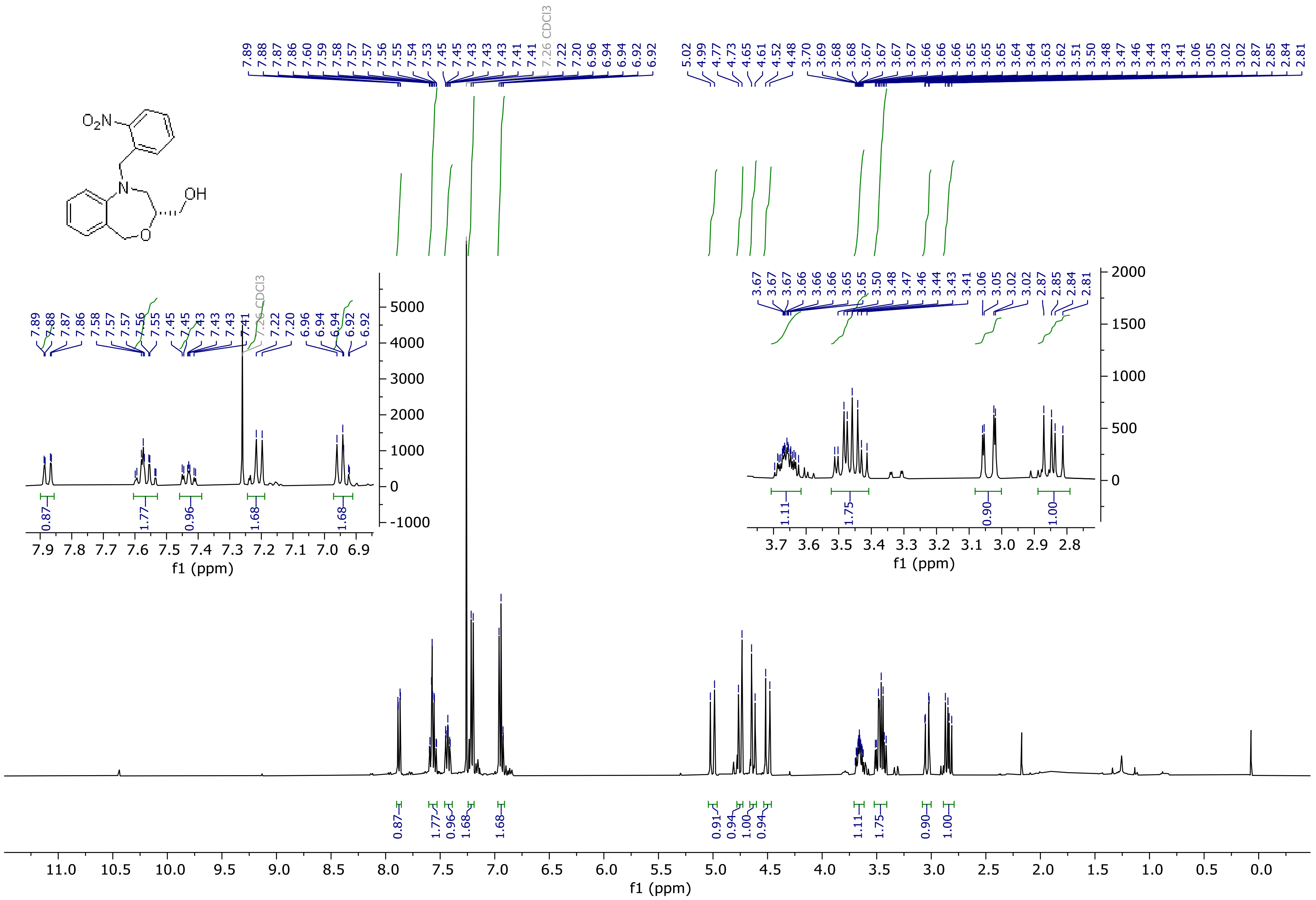

13C{1H}NMR (101 MHz, CDCl3): (R)-(1-(2-Nitrobenzyl)-1,2,3,5-tetrahydrobenzo[e][1,4]oxazepin-3-yl)methanol (2k)

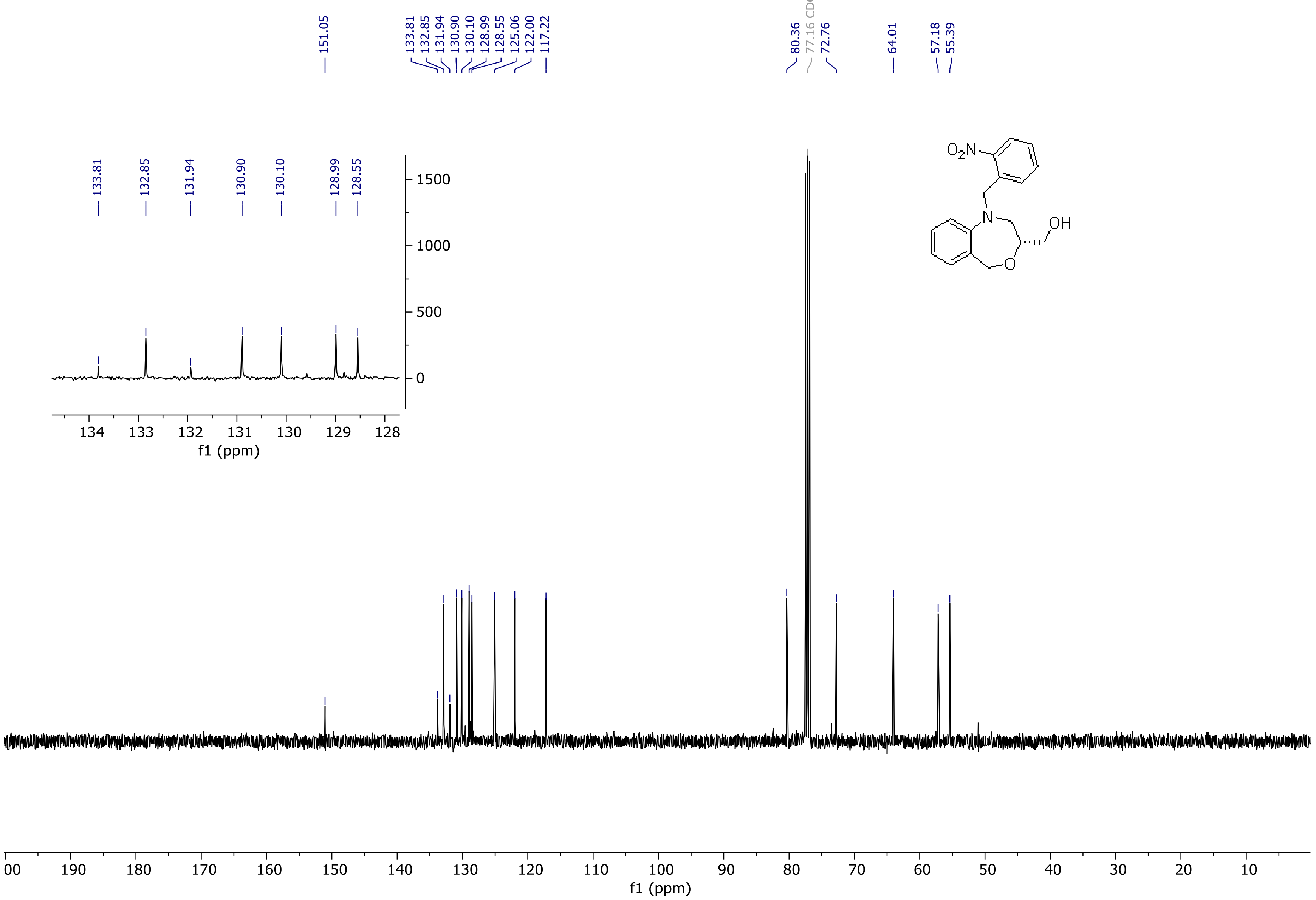

1H NMR: (400 MHz, CDCl3): (R)-(1-(Naphthalen-2-ylmethyl)-1,2,3,5-tetrahydrobenzo[e][1,4]oxazepin-3-yl)methanol (2l)

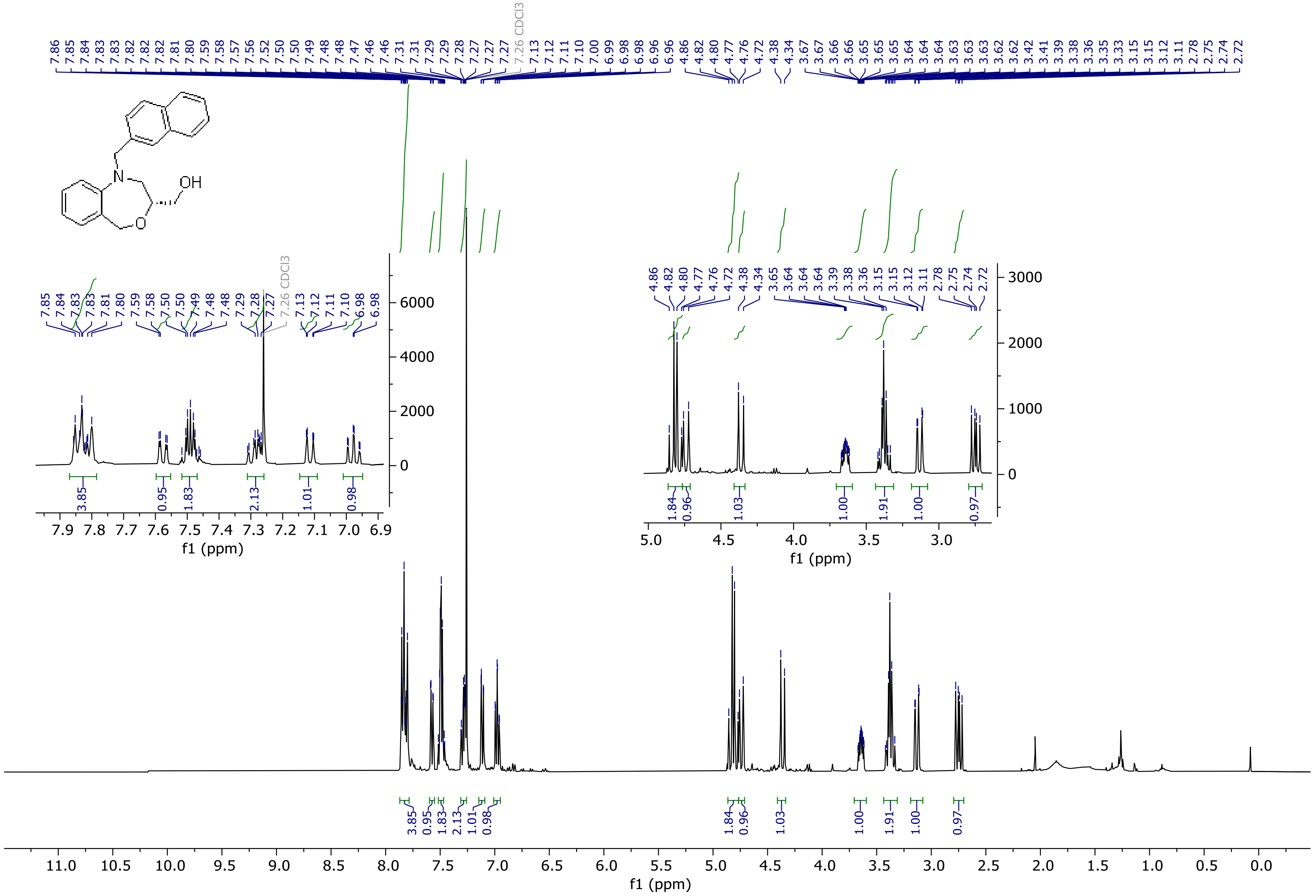

13C{1H}NMR (101 MHz, CDCl3): (R)-(1-(Naphthalen-2-ylmethyl)-1,2,3,5-tetrahydrobenzo[e][1,4]oxazepin-3-yl)methanol (2l)

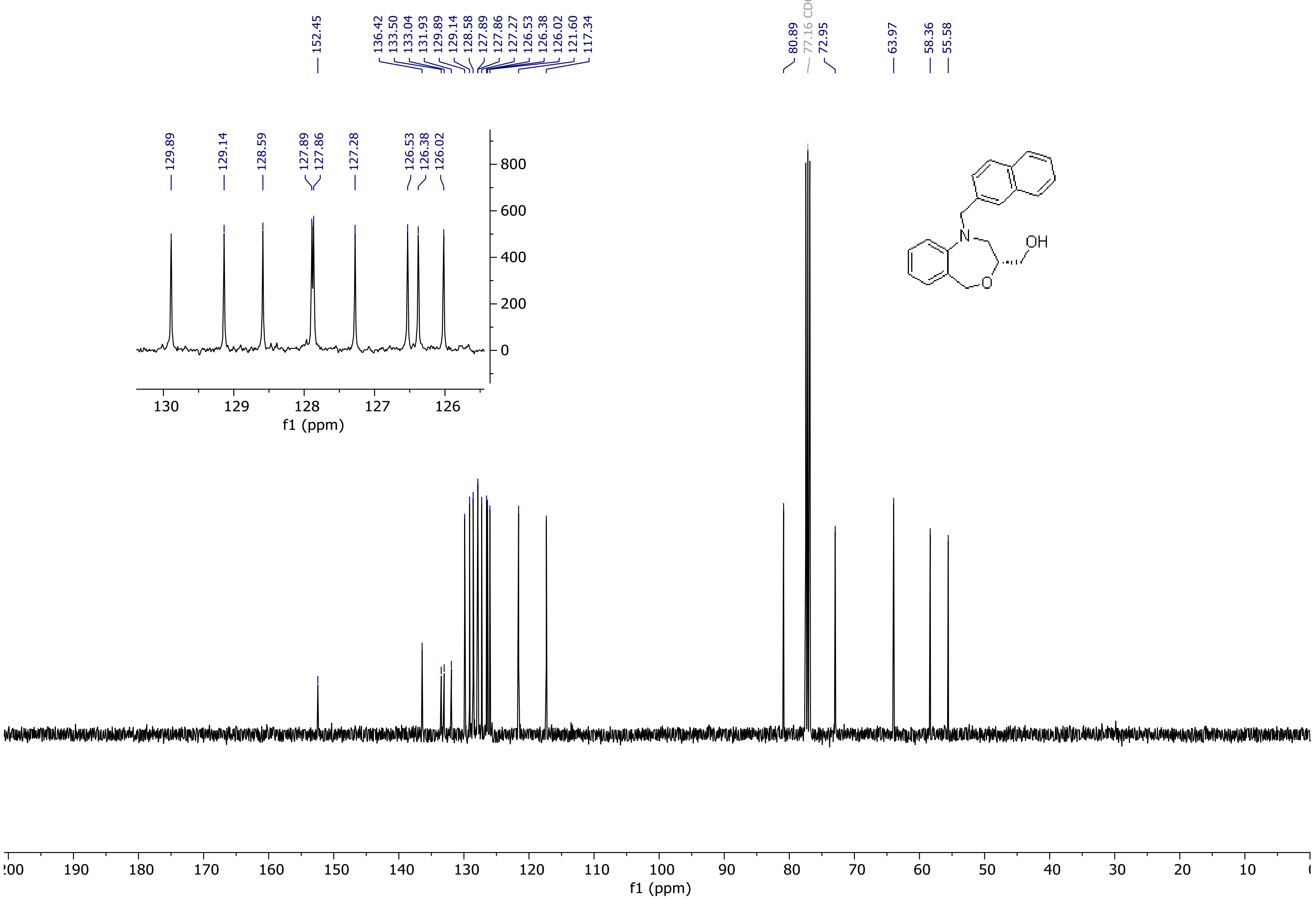

1H NMR: (400 MHz, CDCl3): (R)-(1-(Thiophen-2-ylmethyl)-1,2,3,5-tetrahydrobenzo[e][1,4]oxazepin-3-yl)methanol (2m)

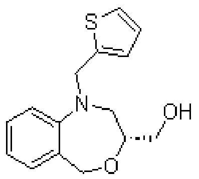

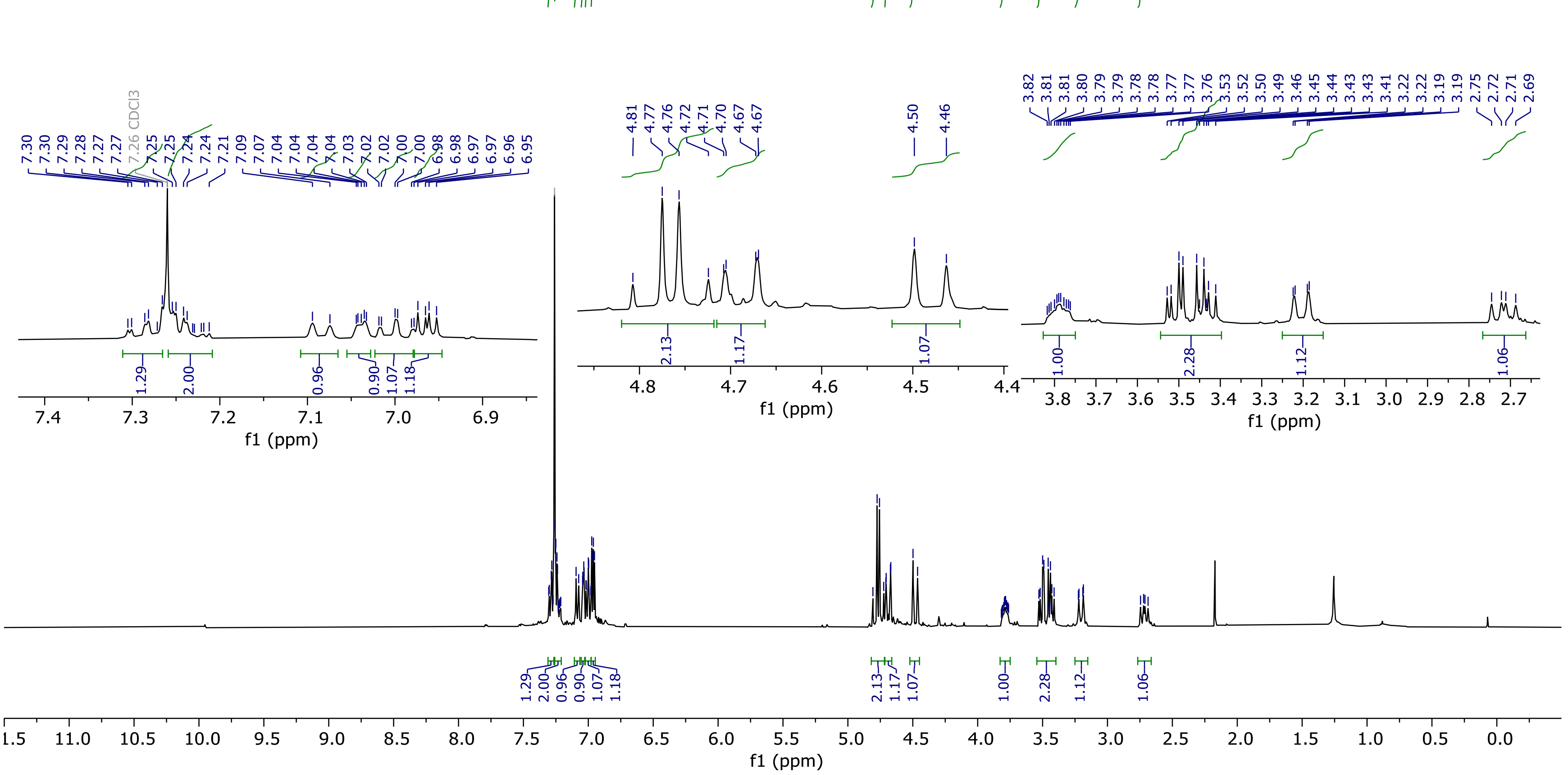

13C{1H}NMR (101 MHz, CDCl3): (R)-(1-(Thiophen-2-ylmethyl)-1,2,3,5-tetrahydrobenzo[e][1,4]oxazepin-3-yl)methanol (2m)

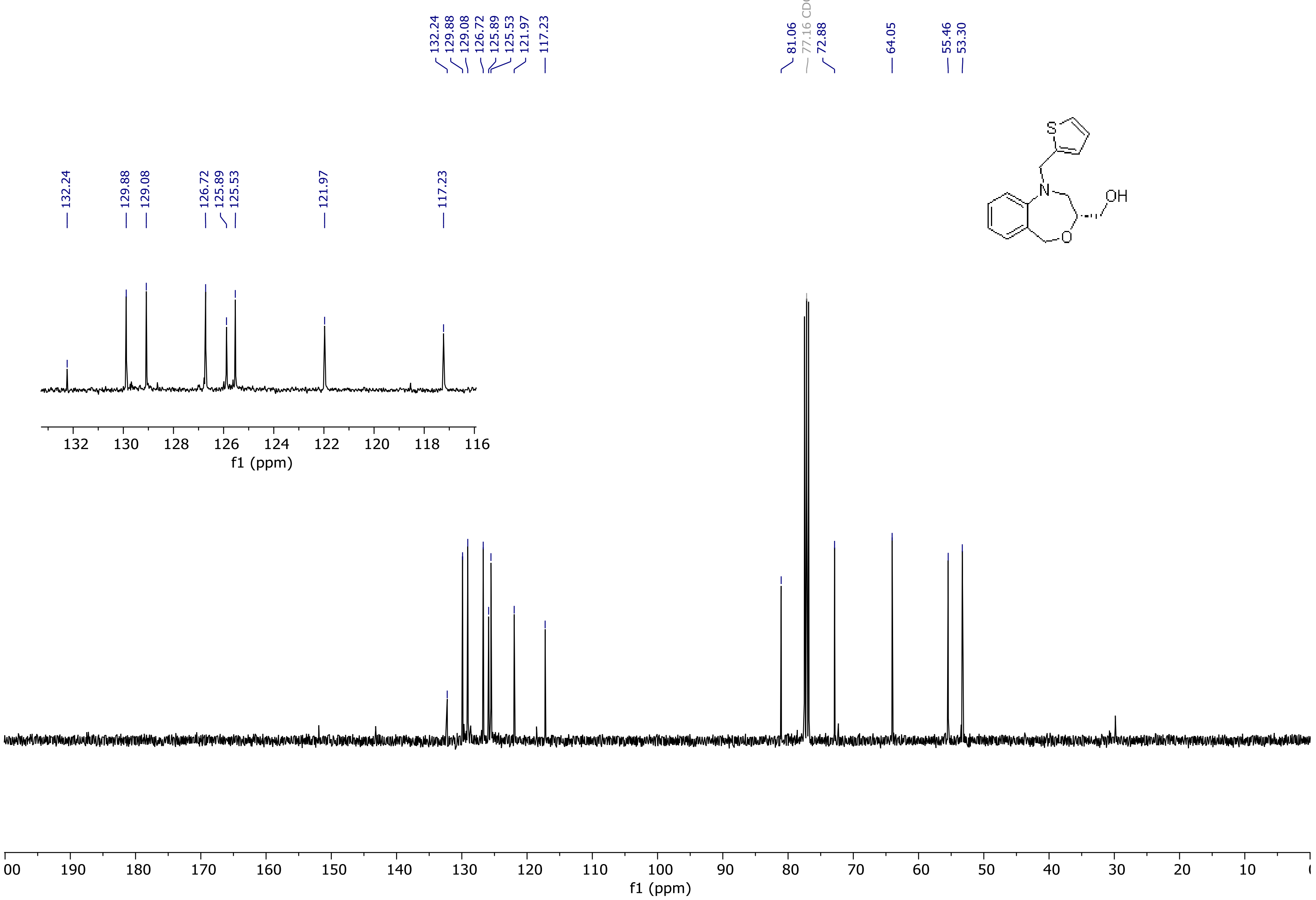

1H NMR: (400 MHz, CDCl3): (R)-(1-Allyl-1,2,3,5-tetrahydrobenzo[e][1,4]oxazepin-3-yl)methanol (2n)

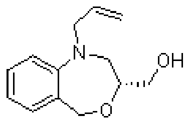

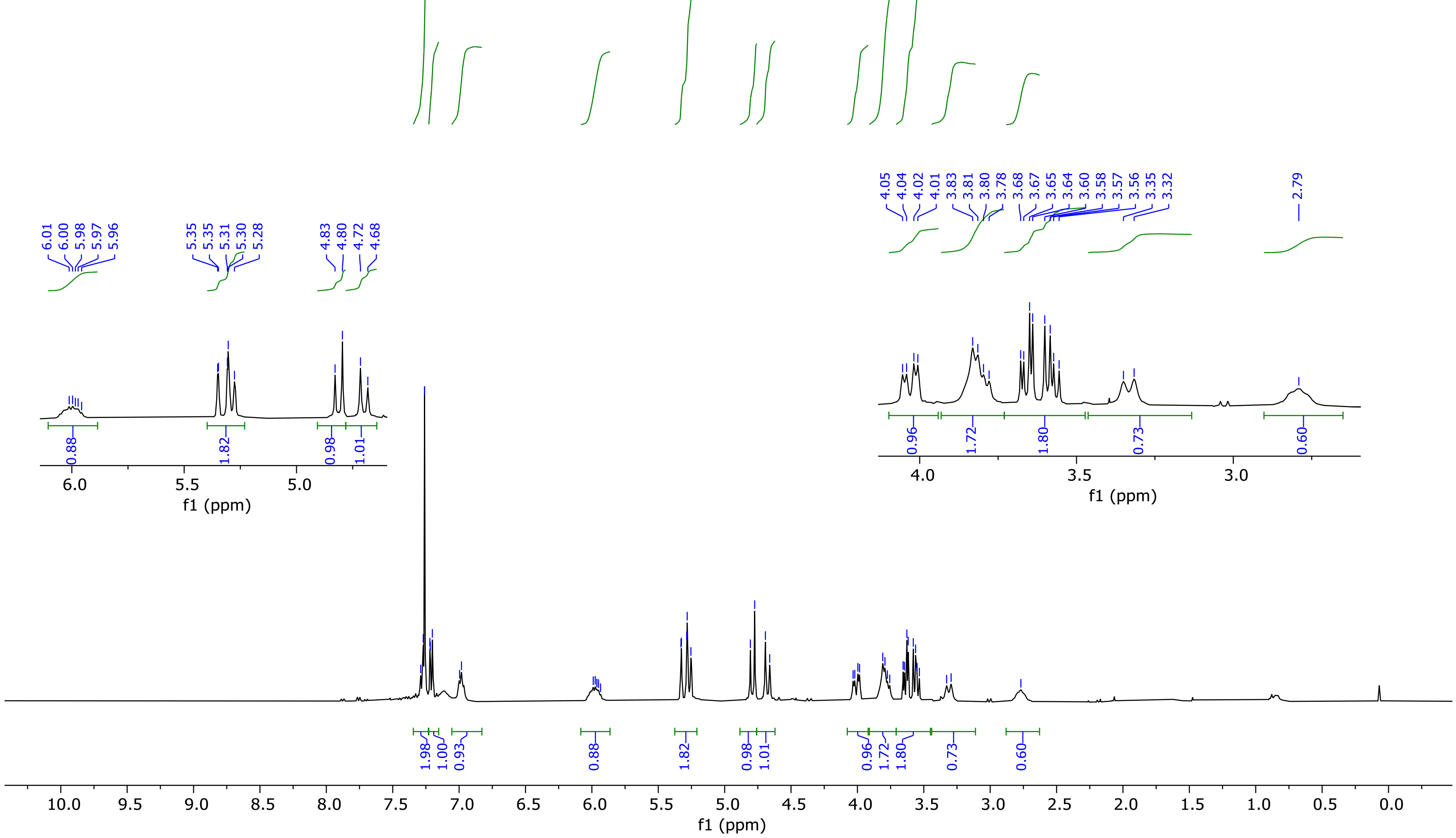

13C{1H}NMR (101 MHz, CDCl3): (R)-(1-Allyl-1,2,3,5-tetrahydrobenzo[e][1,4]oxazepin-3-yl)methanol (2n)

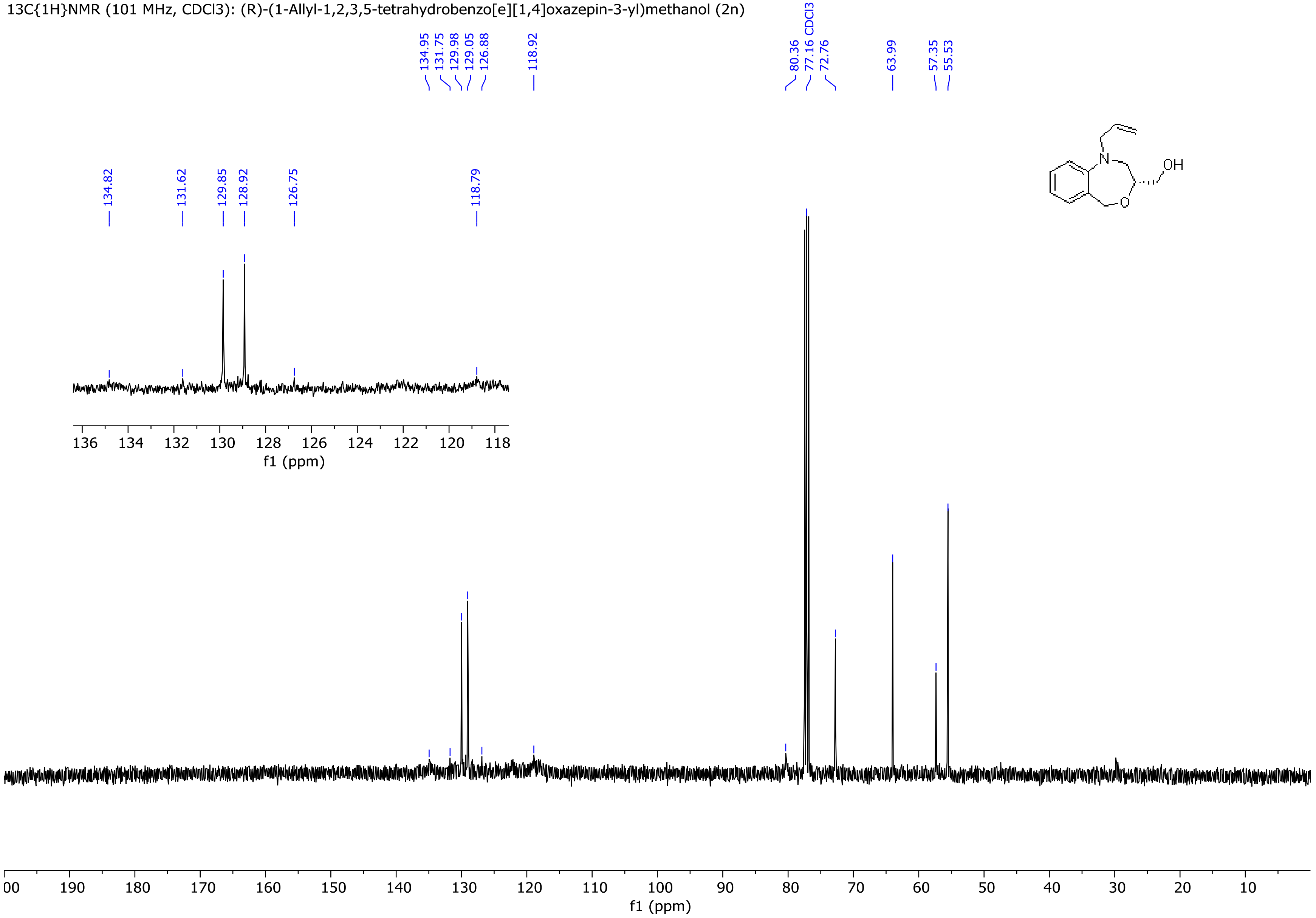

1H NMR: (400 MHz, CDCl3): (R)-(1-Methyl-1,2,3,5-tetrahydrobenzo[e][1,4]oxazepin-3-yl)methanol (2o)

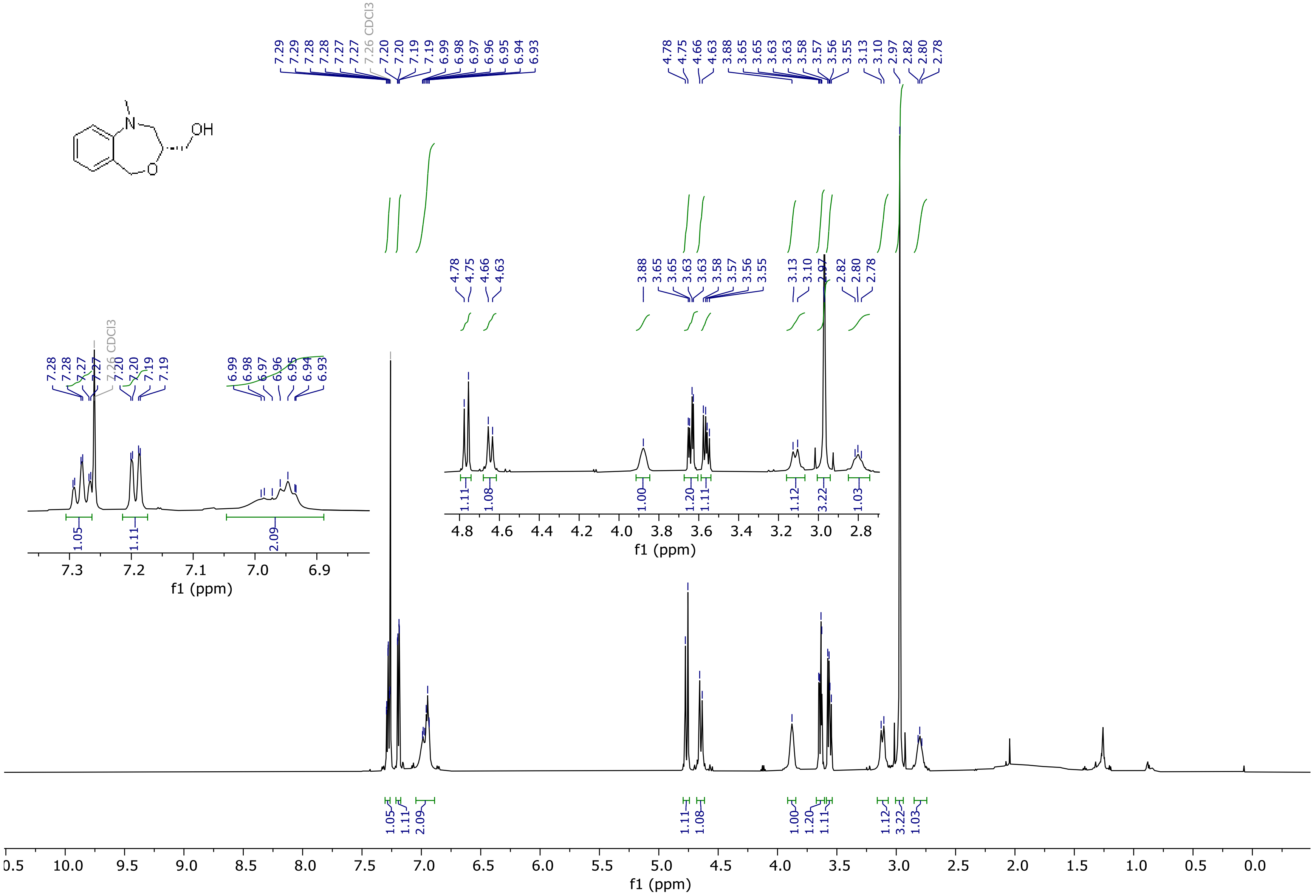

13C{1H}NMR (101 MHz, CDCl3): (R)-(1-Methyl-1,2,3,5-tetrahydrobenzo[e][1,4]oxazepin-3-yl)methanol (2o)

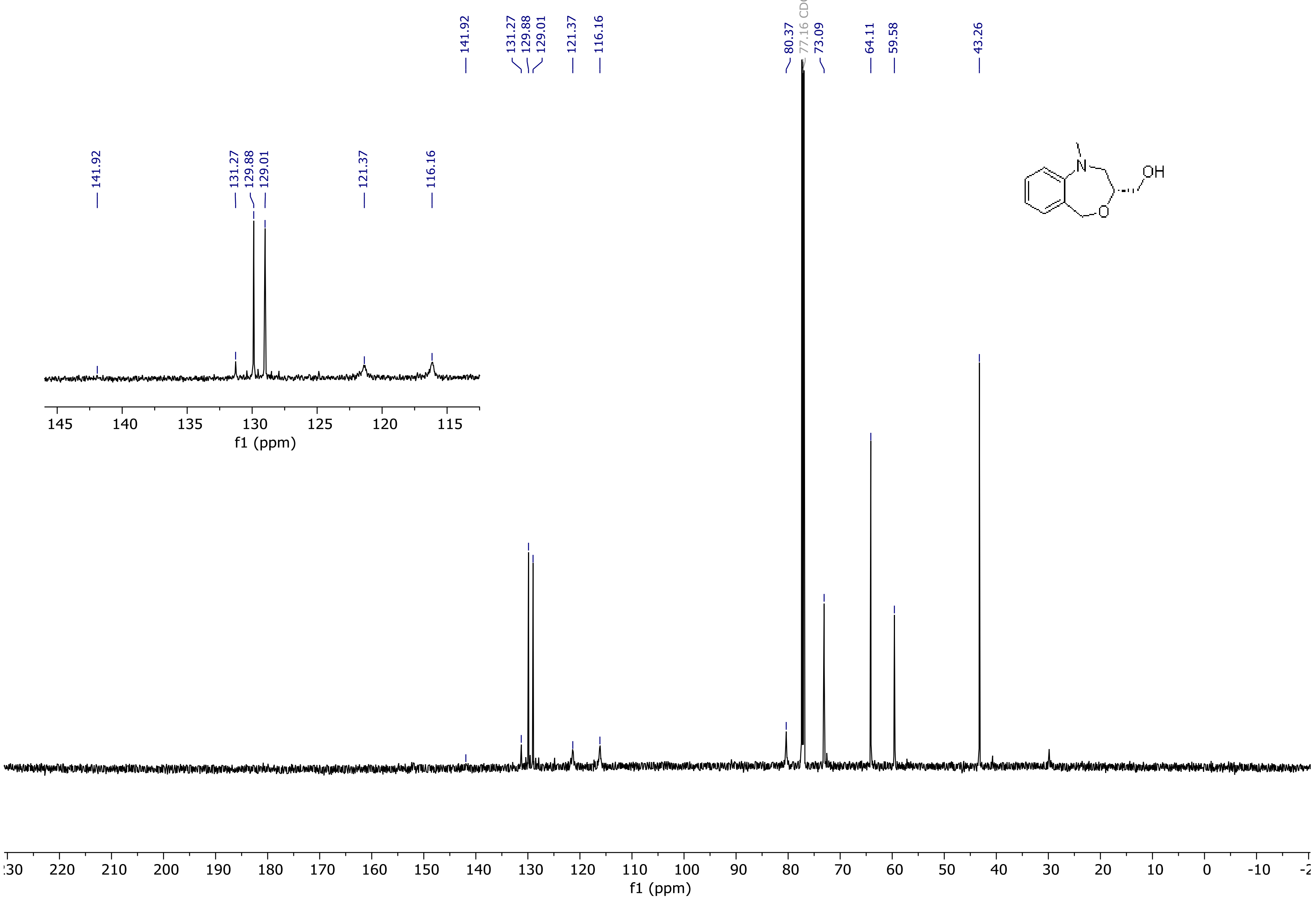

1H NMR: (400 MHz, CDCl3): (R)-(1-Phenyl-1,2,3,5-tetrahydrobenzo[e][1,4]oxazepin-3-yl)methanol (2q)

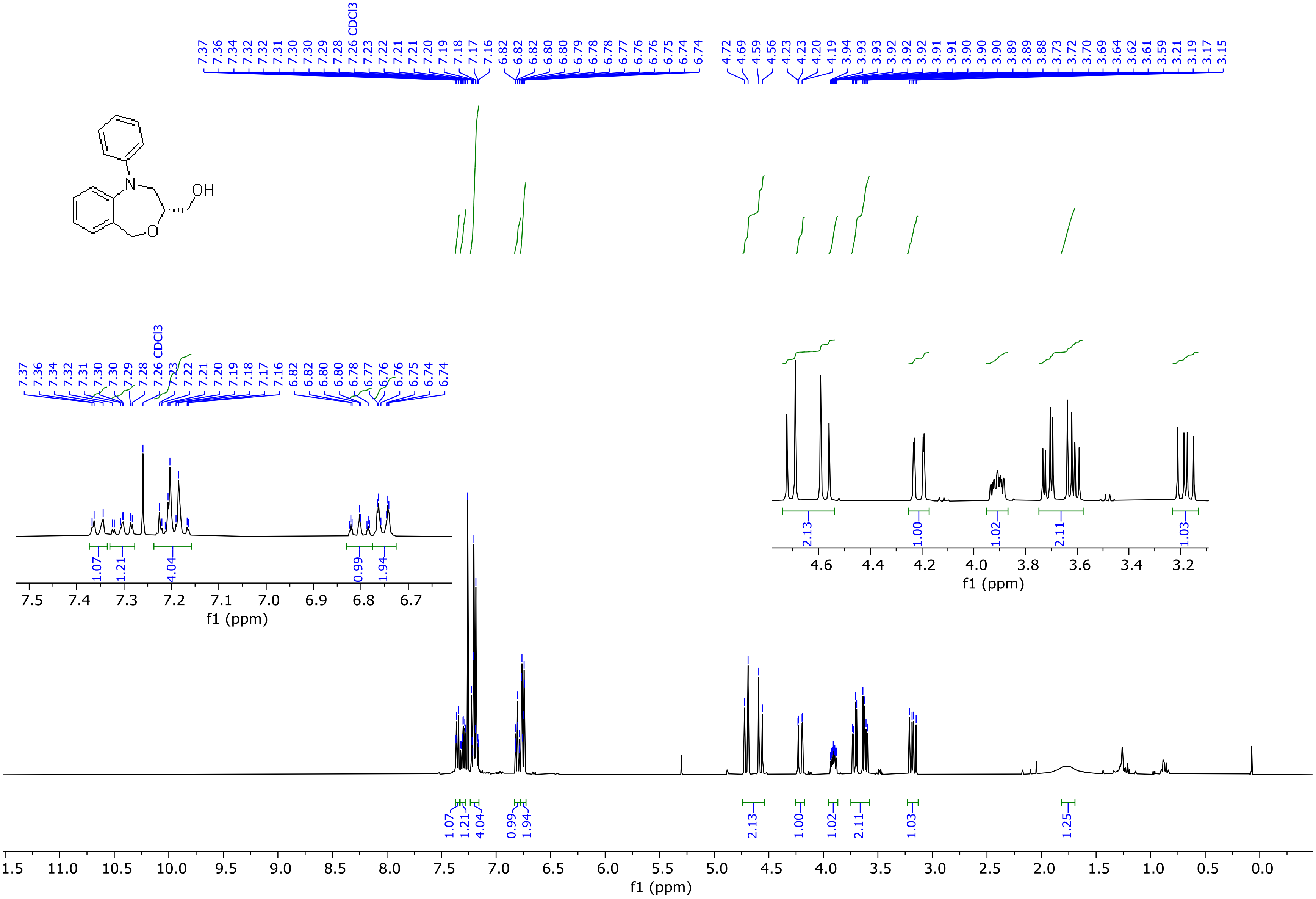

13C{1H}NMR (101 MHz, CDCl3): (R)-(1-Phenyl-1,2,3,5-tetrahydrobenzo[e][1,4]oxazepin-3-yl)methanol (2q)

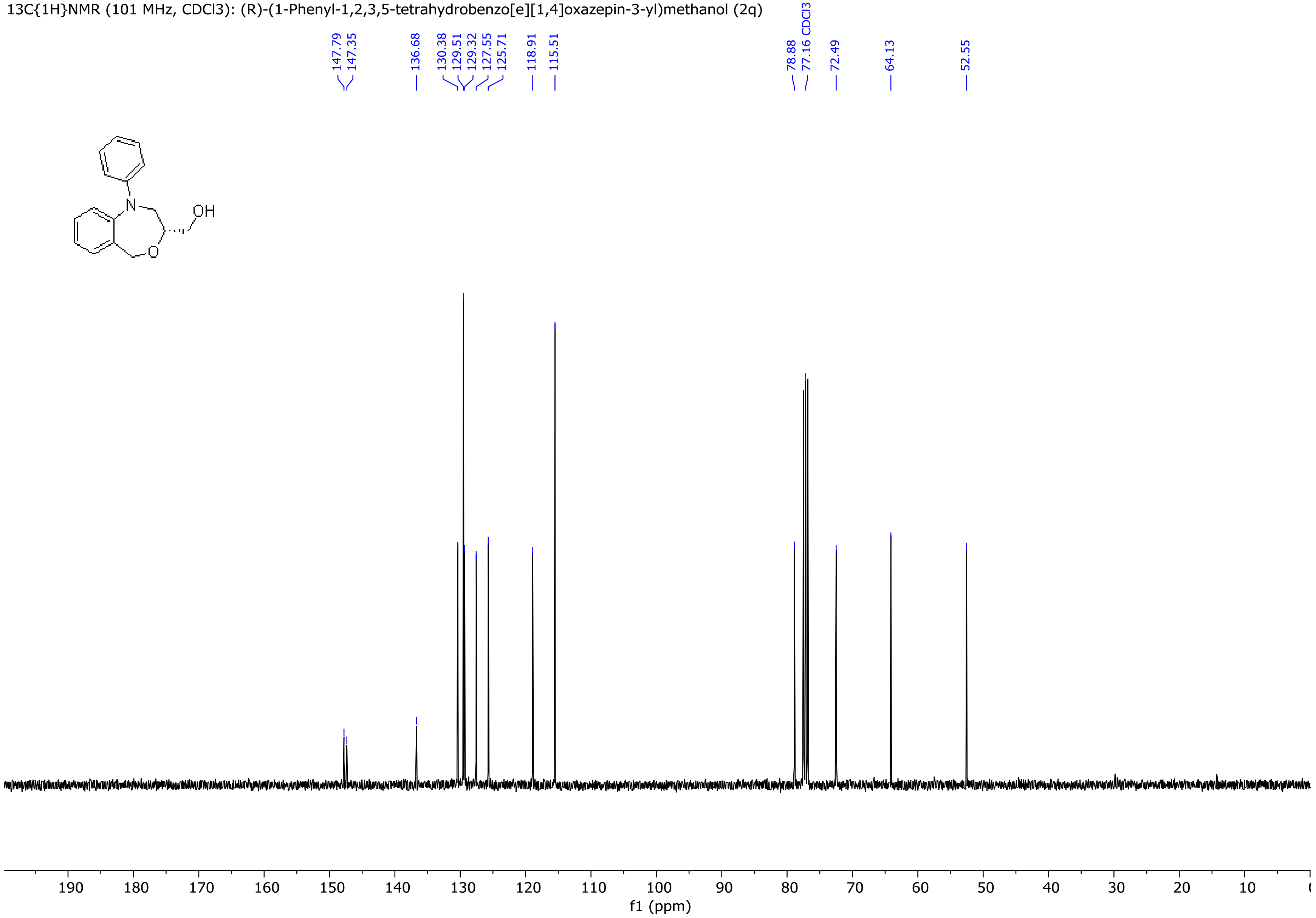

1H NMR: (400 MHz, CDCl3): (R)-(1-(4-Methoxyphenyl)-1,2,3,5-tetrahydrobenzo[e][1,4]oxazepin-3-yl)methanol (2r)

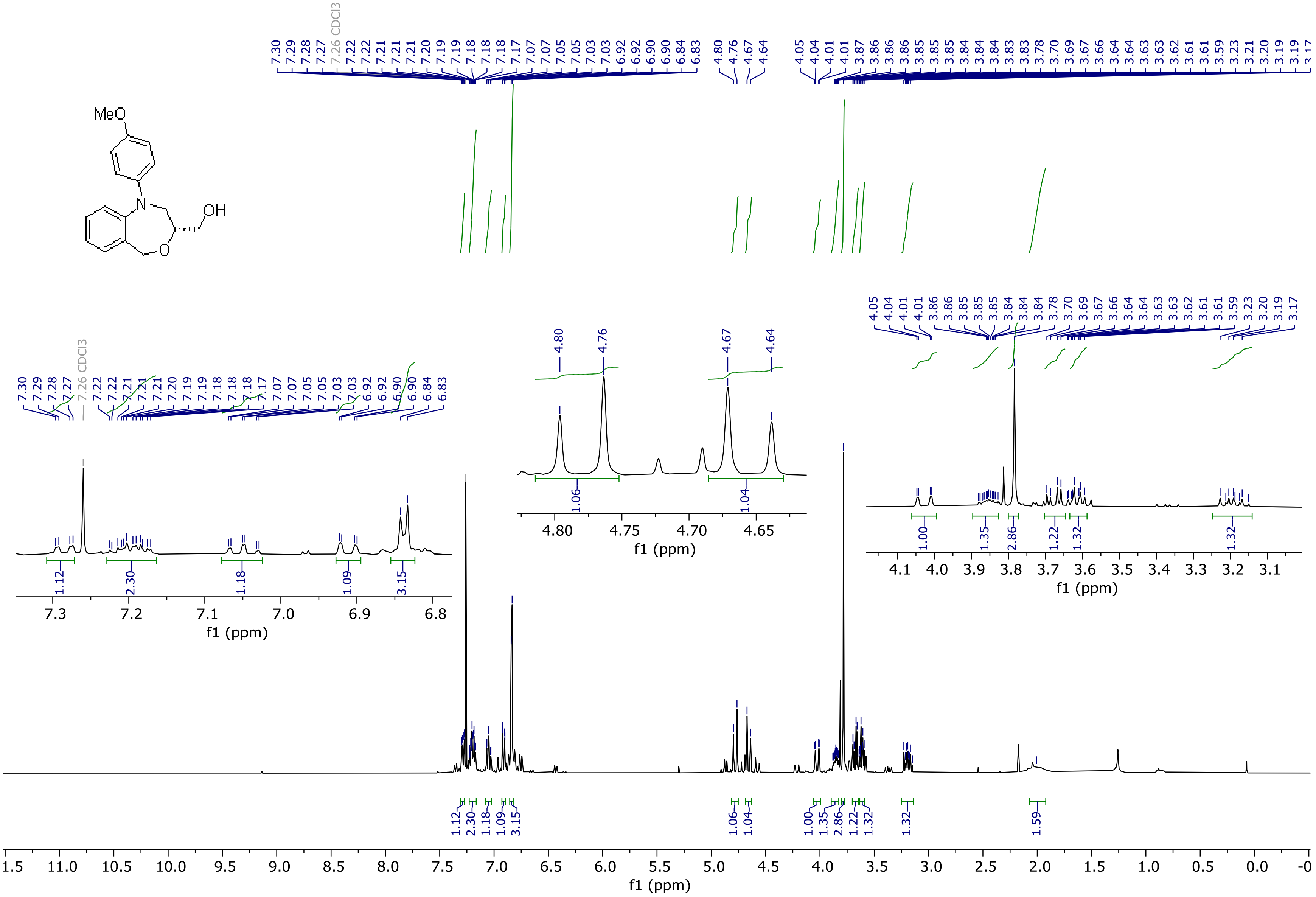

13C{1H}NMR (101 MHz, CDCl3): (R)-(1-(4-Methoxyphenyl)-1,2,3,5-tetrahydrobenzo[e][1,4]oxazepin-3-yl)methanol (2r)

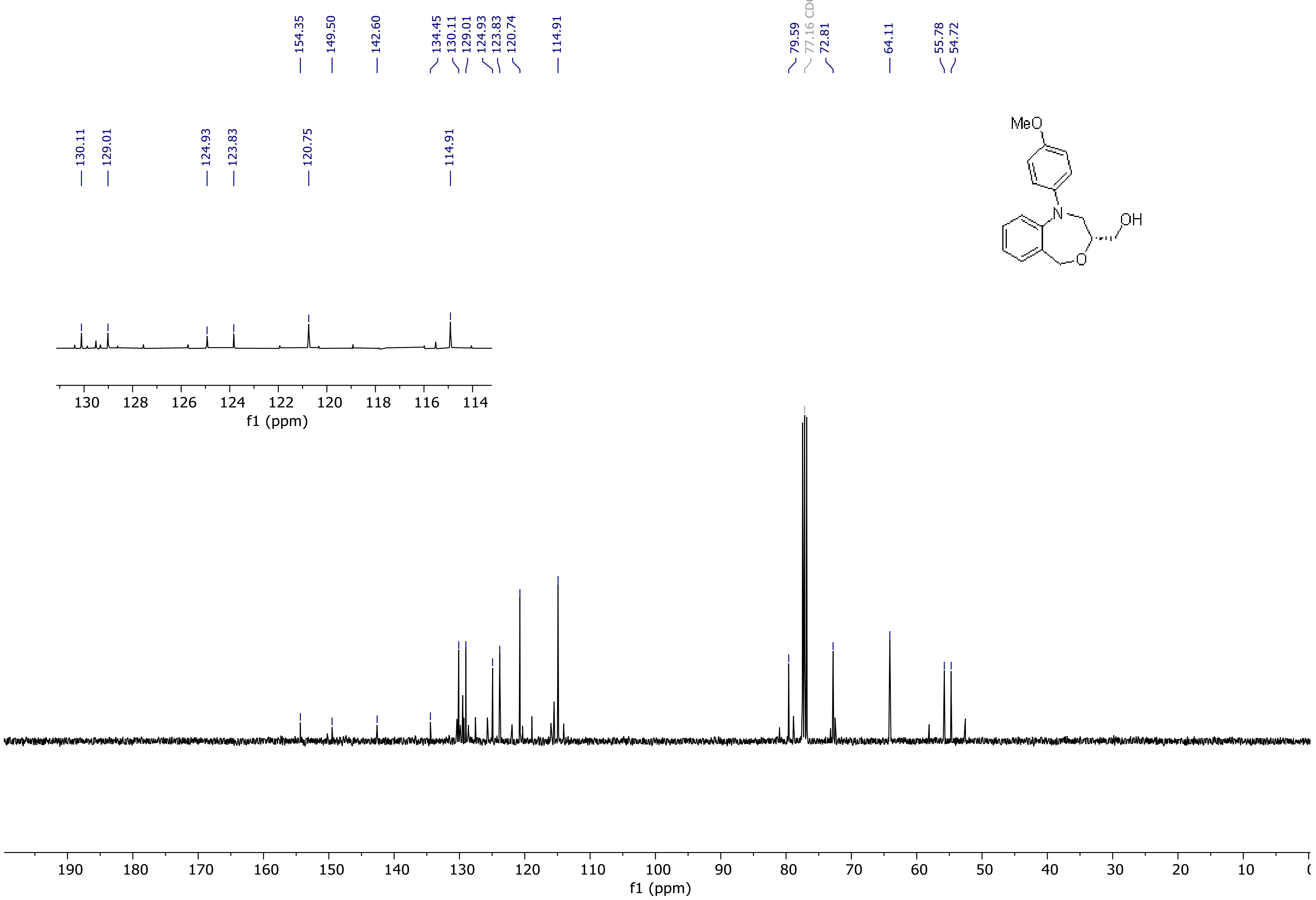

1H NMR (400 MHz, CDCl3): (R)-(1-(4-bromophenyl)-1,2,3,5-tetrahydrobenzo[e][1,4]oxazepin-3-yl)methanol (2u)

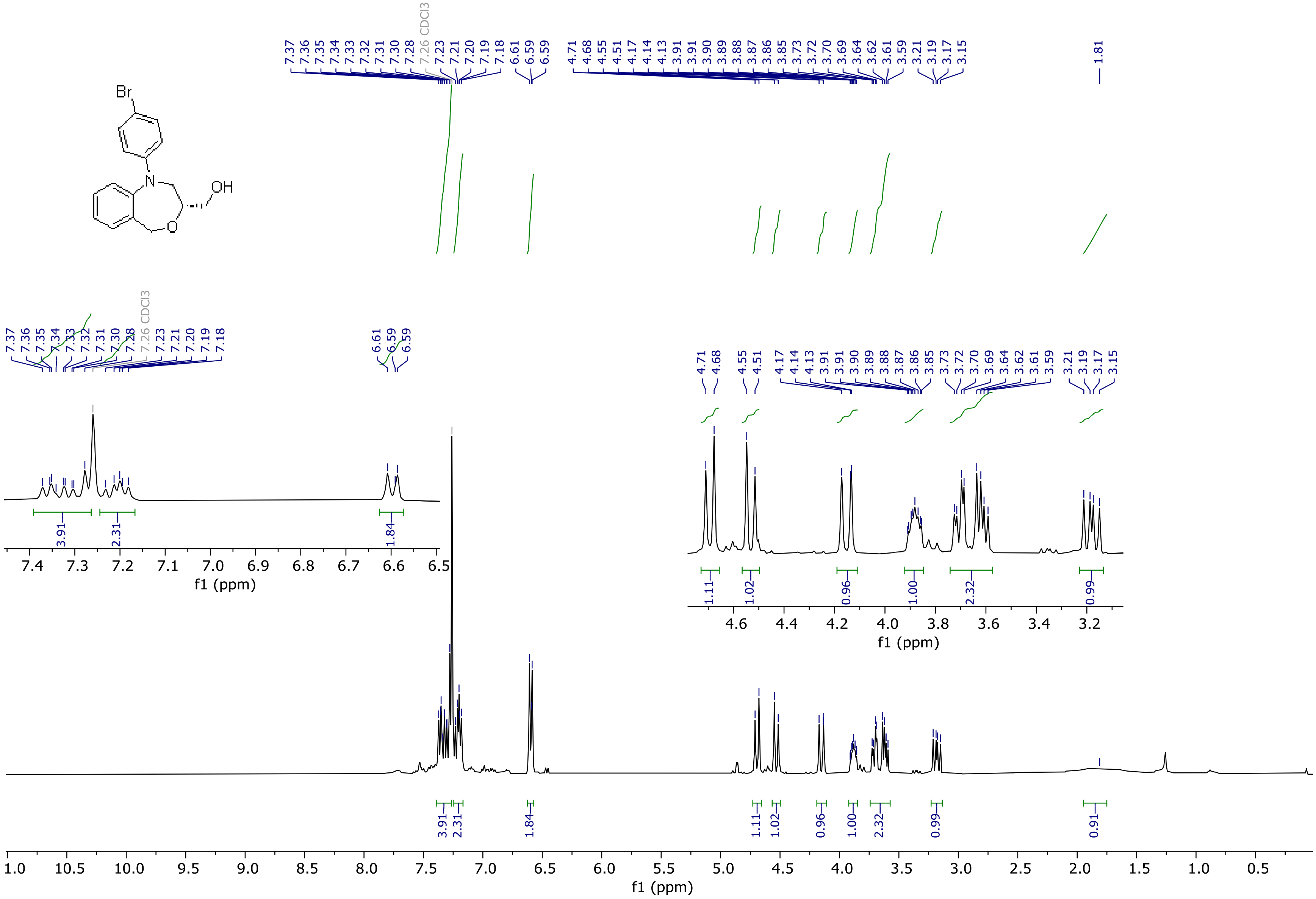

13C{1H}NMR (101 MHz, CDCl3): (R)-(1-(4-Bromophenyl)-1,2,3,5-tetrahydrobenzo[e][1,4]oxazepin-3-yl)methanol (2u)

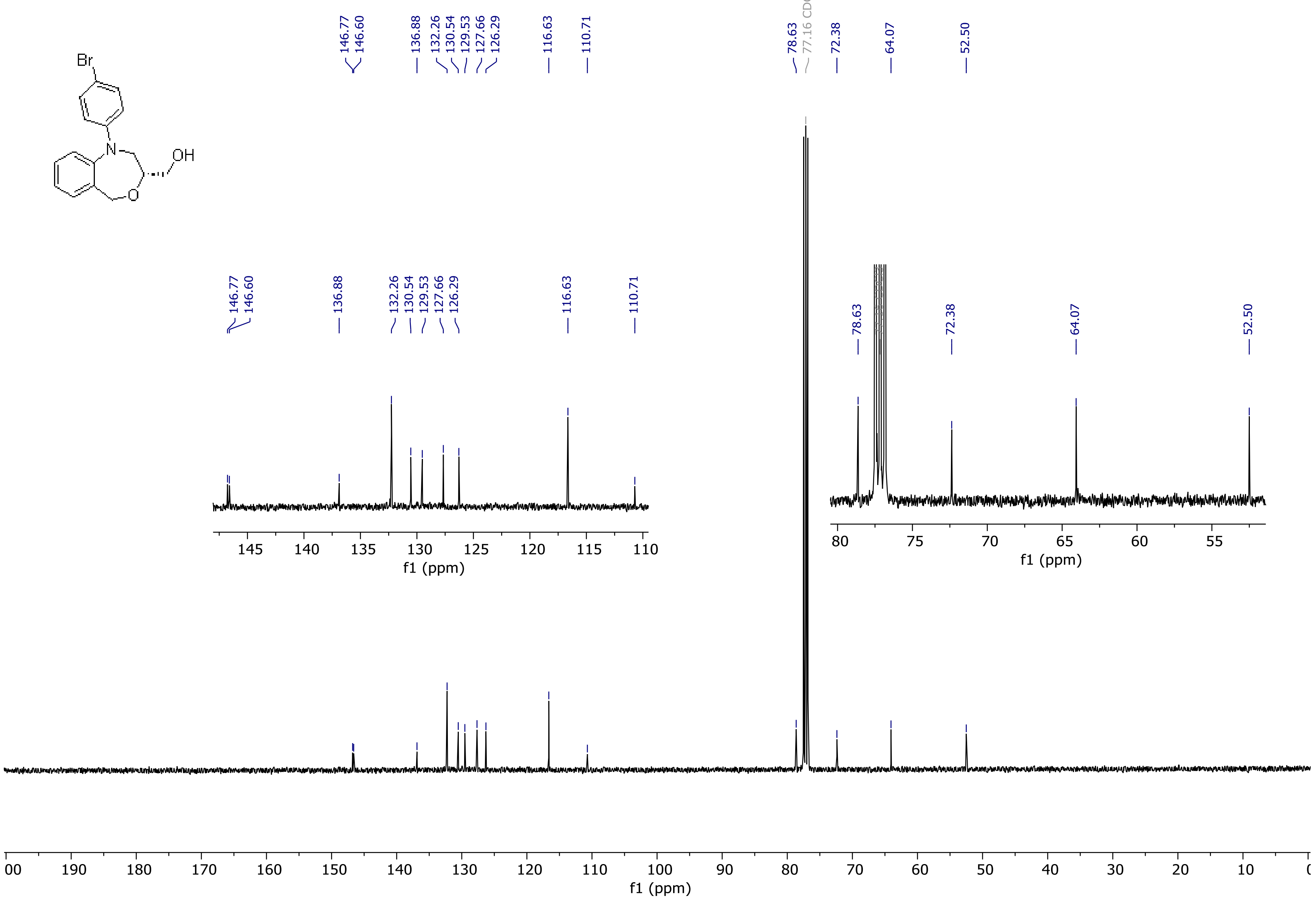

1H NMR: (400 MHz, CDCl3): (R)-(1-Benzyl-9-methyl-1,2,3,5-tetrahydrobenzo[e][1,4]oxazepin-3-yl)methanol (2v)

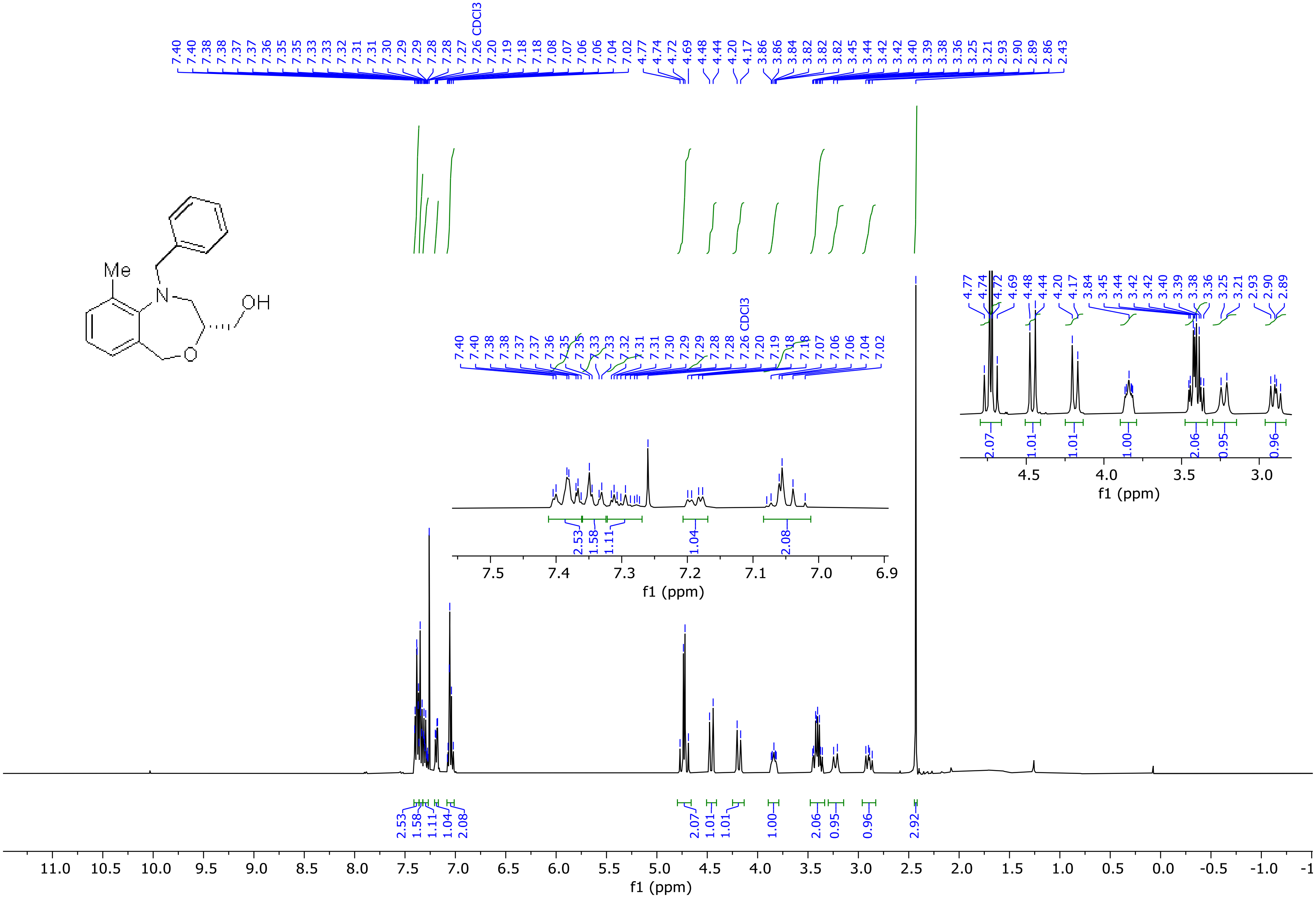

13C{1H}NMR (101 MHz, CDCl3): (R)-(1-Benzyl-9-methyl-1,2,3,5-tetrahydrobenzo[e][1,4]oxazepin-3-yl)methanol (2v)

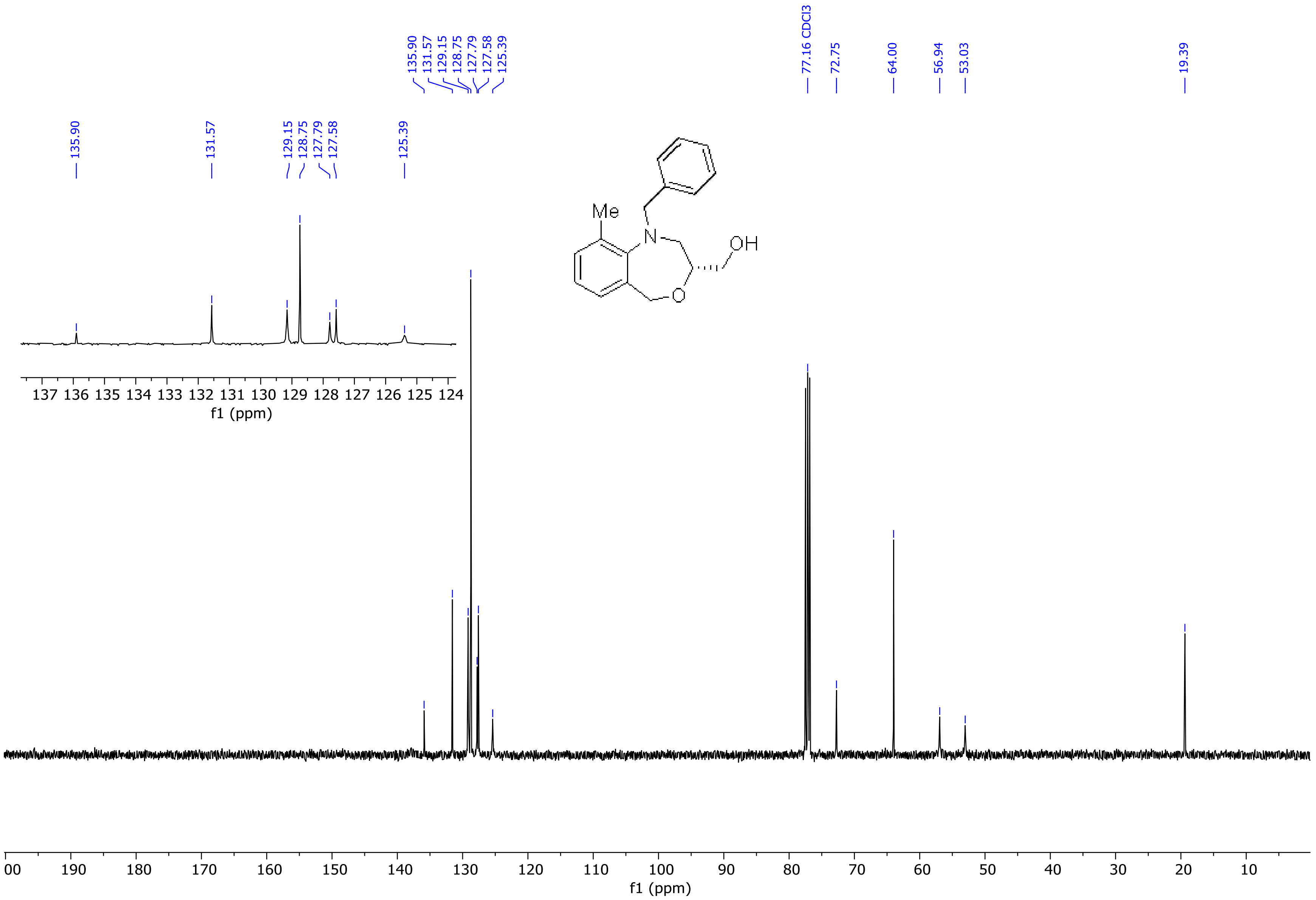

1H NMR: (400 MHz, CDCl3): (R)-(1-Benzyl-8-chloro-1,2,3,5-tetrahydrobenzo[e][1,4]oxazepin-3-yl)methanol (2w)

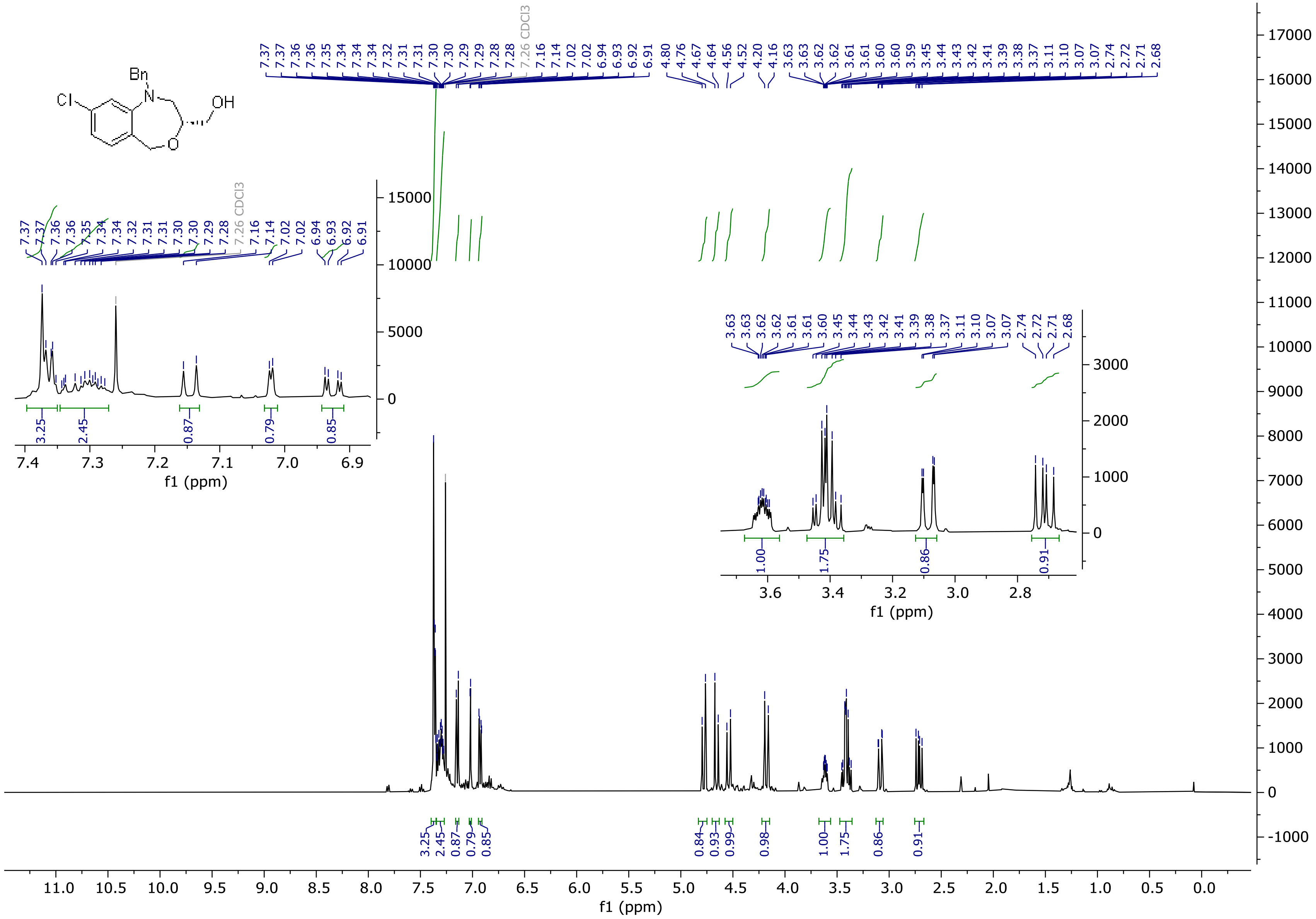

13C{1H}NMR (101 MHz, CDCl3): (R)-(1-Benzyl-8-chloro-1,2,3,5-tetrahydrobenzo[e][1,4]oxazepin-3-yl)methanol (2w)

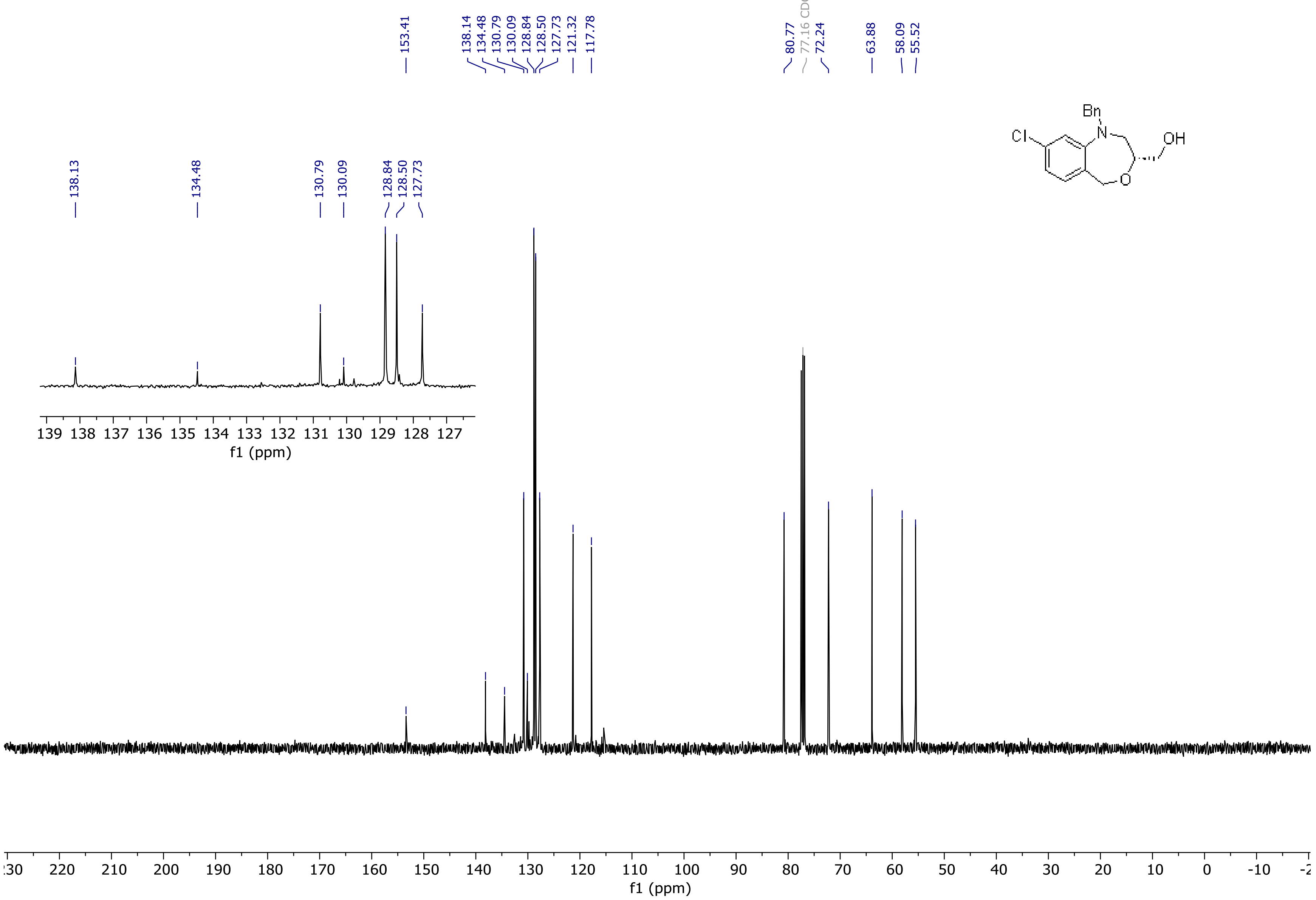

1H NMR: (400 MHz, CDCl3): (R)-(1-Benzyl-8-fluoro-1,2,3,5-tetrahydrobenzo[e][1,4]oxazepin-3-yl)methanol (2x)

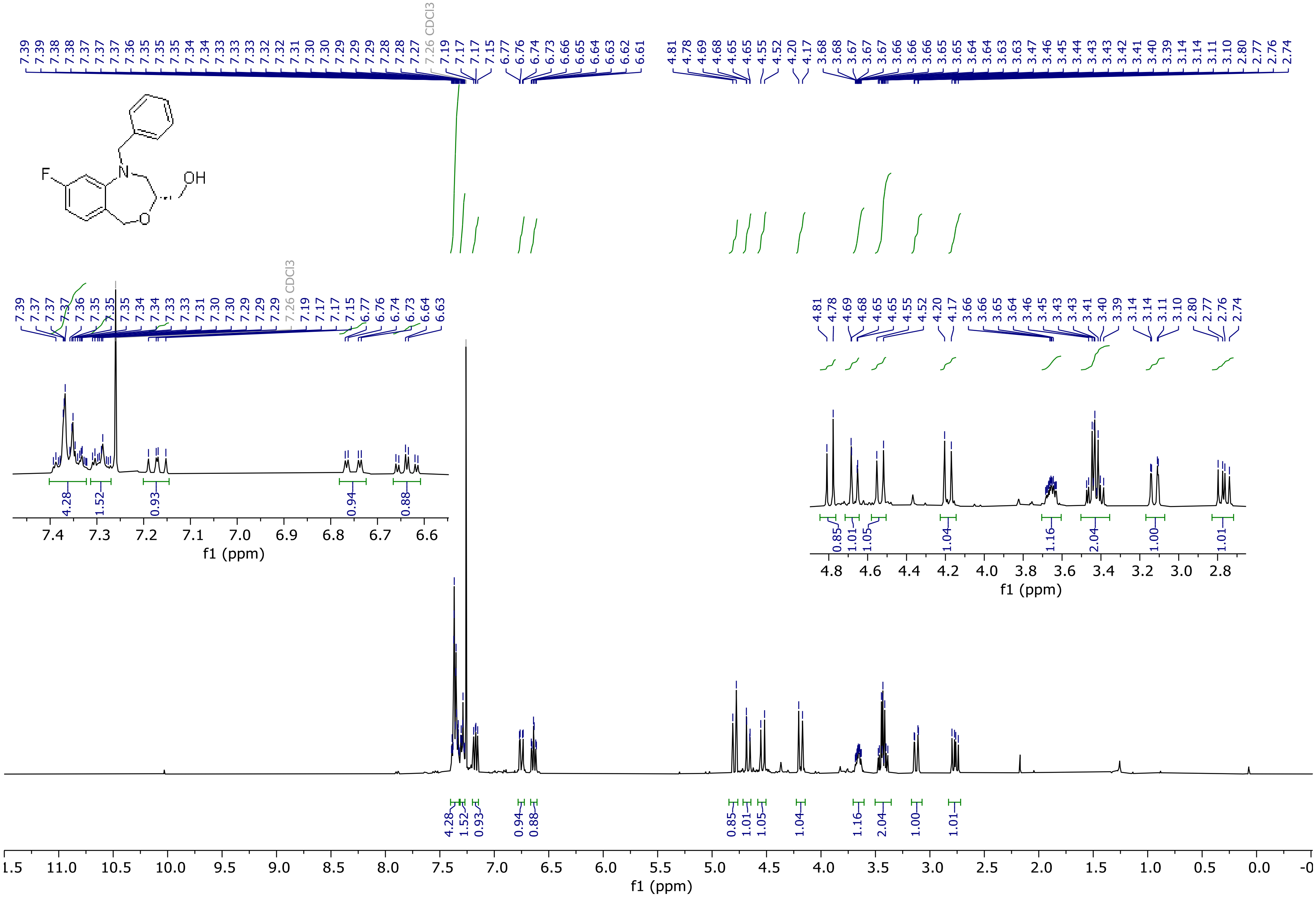

13C{1H}NMR (101 MHz, CDCl3): (R)-(1-Benzyl-8-fluoro-1,2,3,5-tetrahydrobenzo[e][1,4]oxazepin-3-yl)methanol (2x)

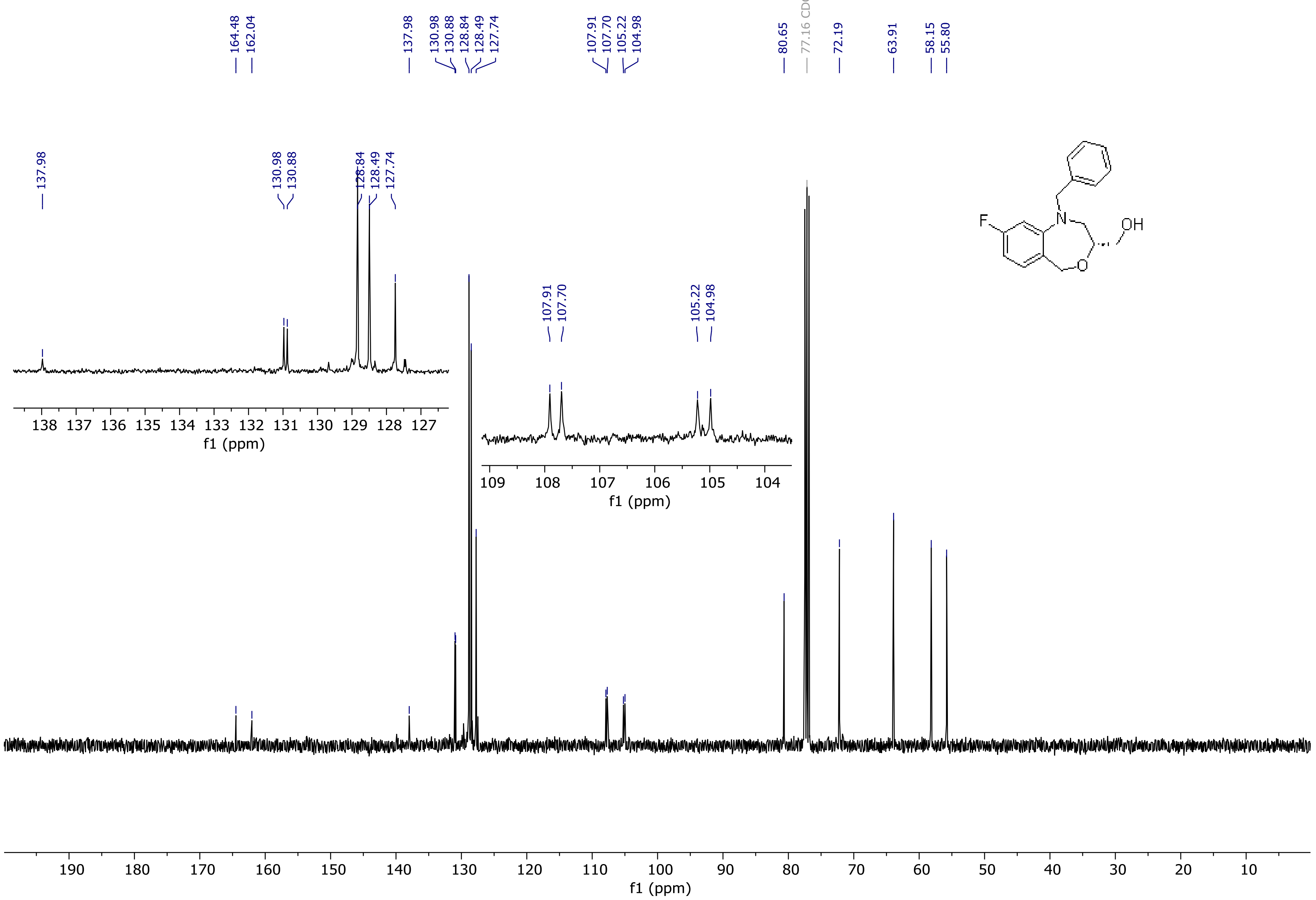

19F NMR (376 MHz, CDCl3): (R)-(1-Benzyl-8-fluoro-1,2,3,5-tetrahydrobenzo[e][1,4]oxazepin-3-yl)methanol (2x)

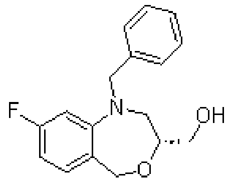

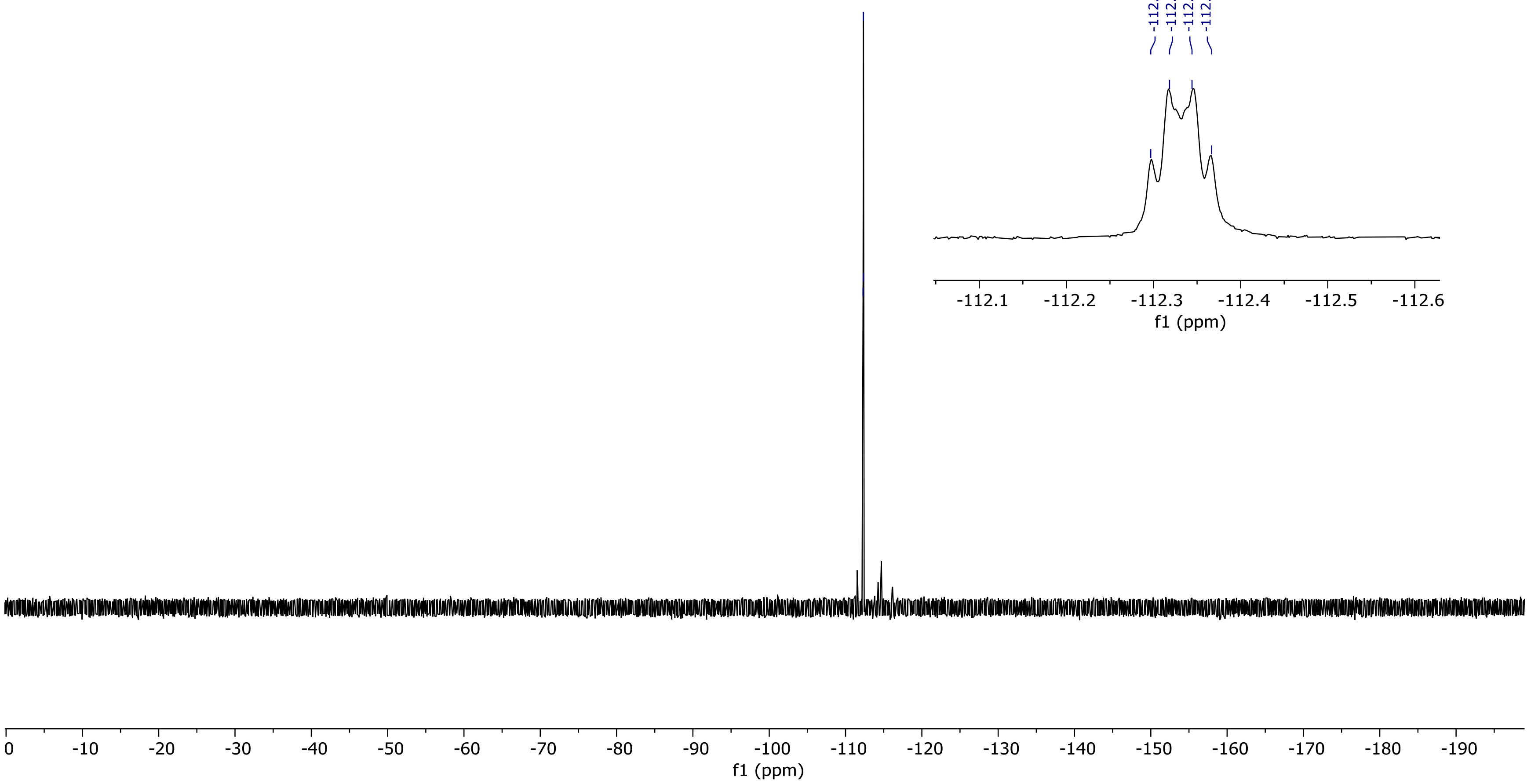

1H NMR (600 MHz, CDCl3): (R)-(1-Benzyl-8-methyl-1,2,3,5-tetrahydrobenzo[e][1,4]oxazepin-3-yl)methanol (2y)

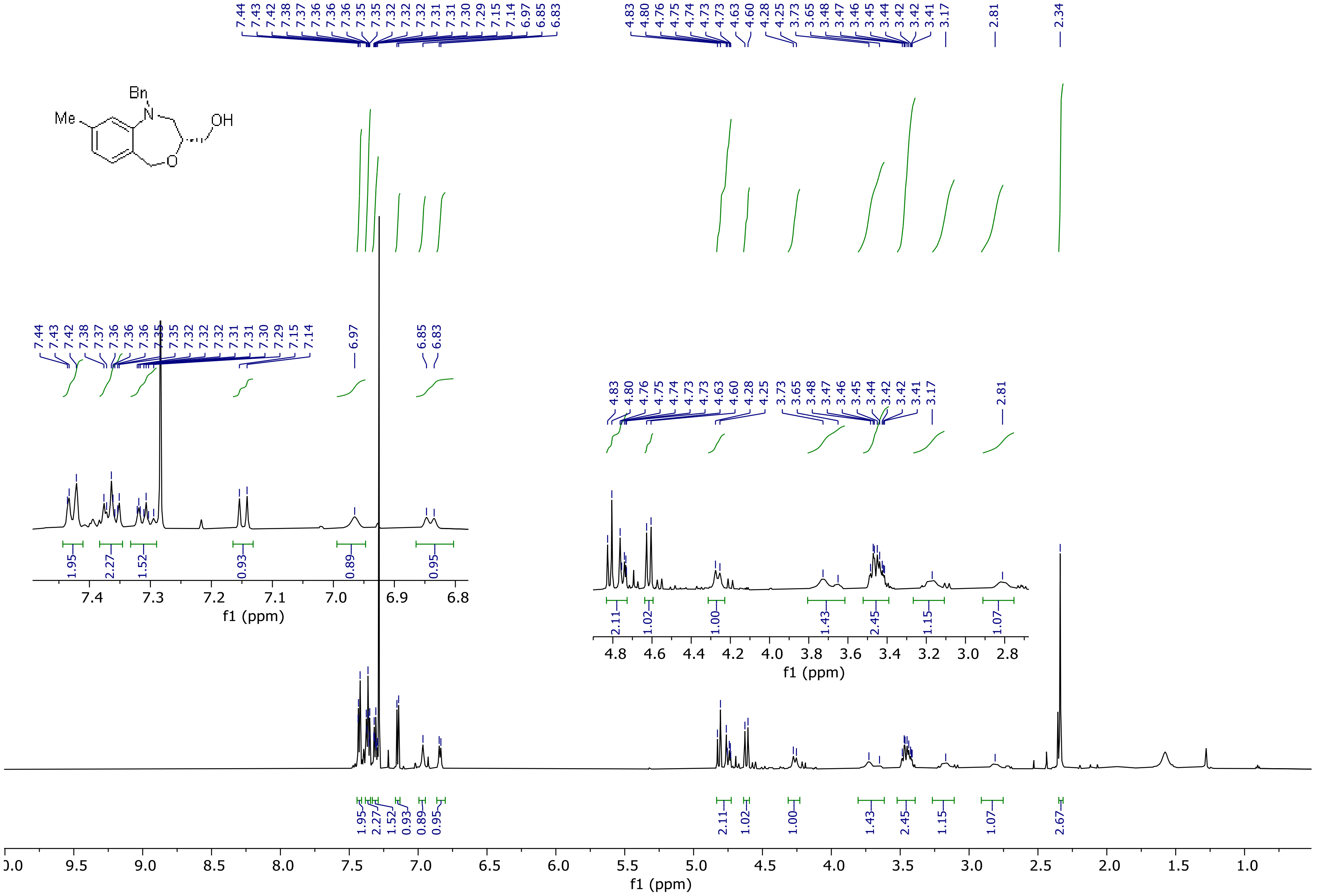

13C{1H}NMR (151 MHz, CDCl3): (R)-(1-Benzyl-8-methyl-1,2,3,5-tetrahydrobenzo[e][1,4]oxazepin-3-yl)methanol (2y)

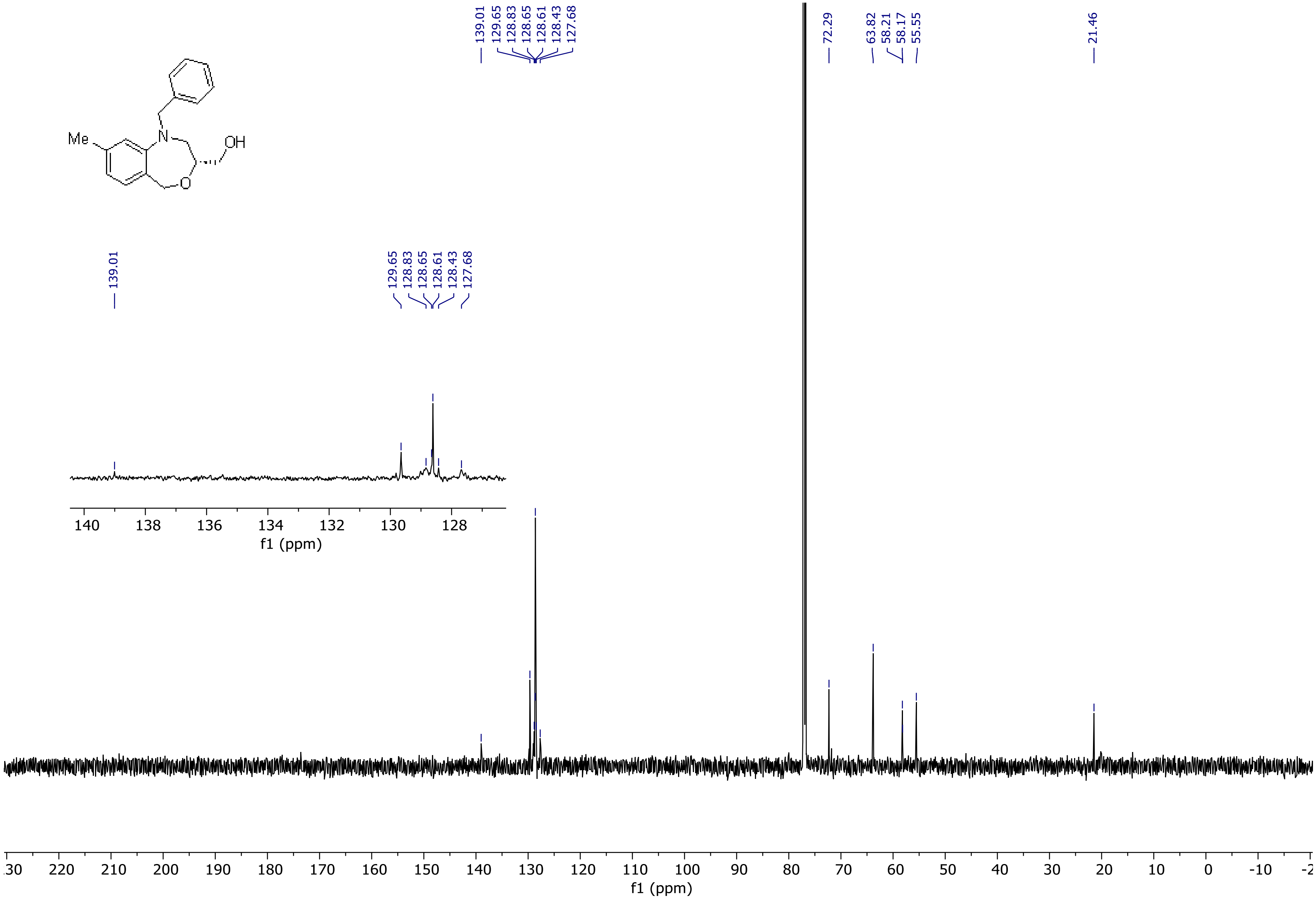

1H NMR: (400 MHz, CDCl3): (R)-(1,2,3,5-Tetrahydrobenzo[e][1,4]oxazepin-3-yl)methanol (3a)

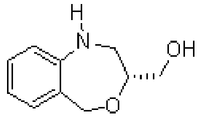

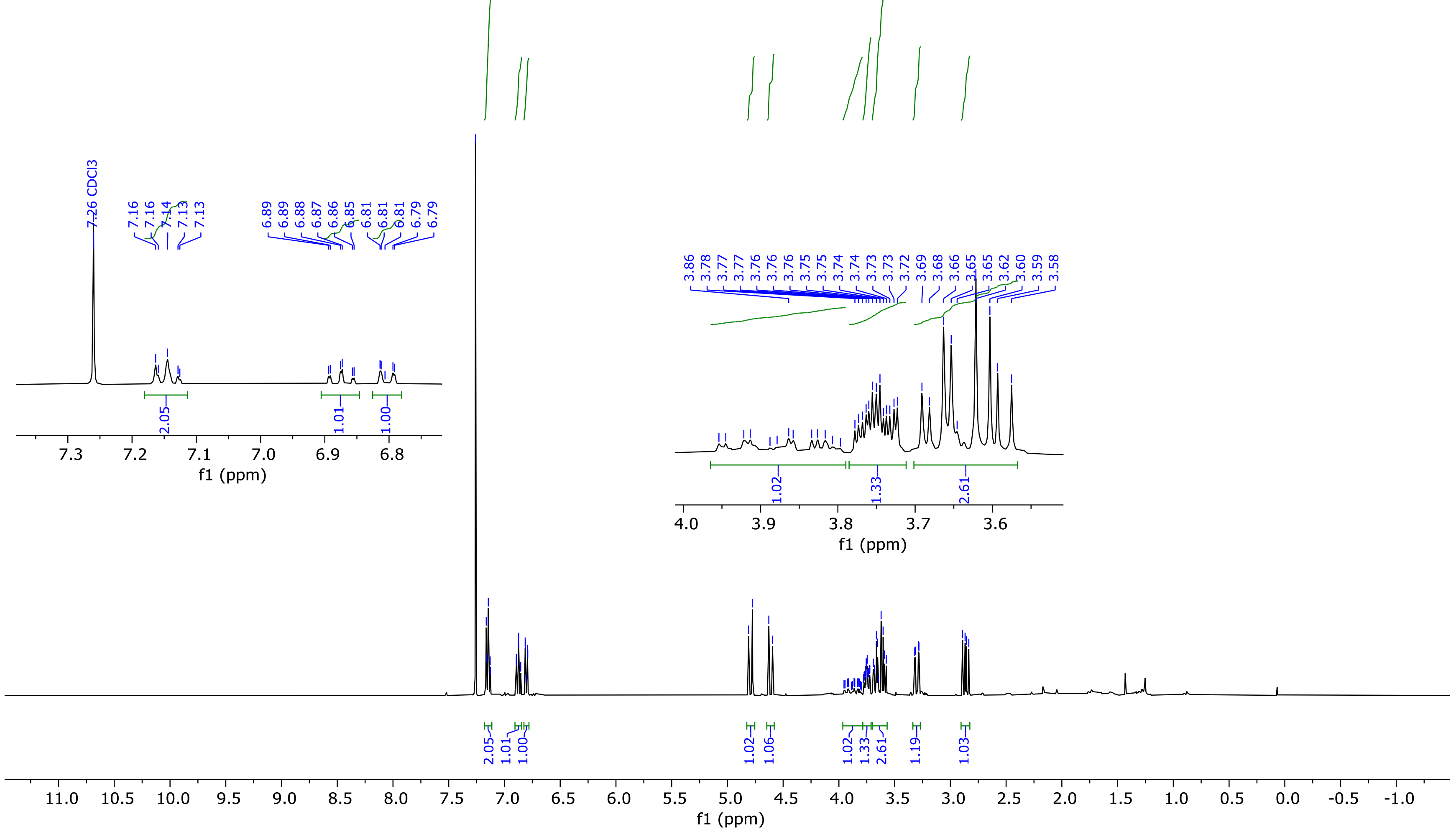

13C{1H}NMR (101 MHz, CDCl3): 1H NMR: (400 MHz, CDCl3): (R)-(1,2,3,5-Tetrahydrobenzo[e][1,4]oxazepin-3-yl)methanol (3a)

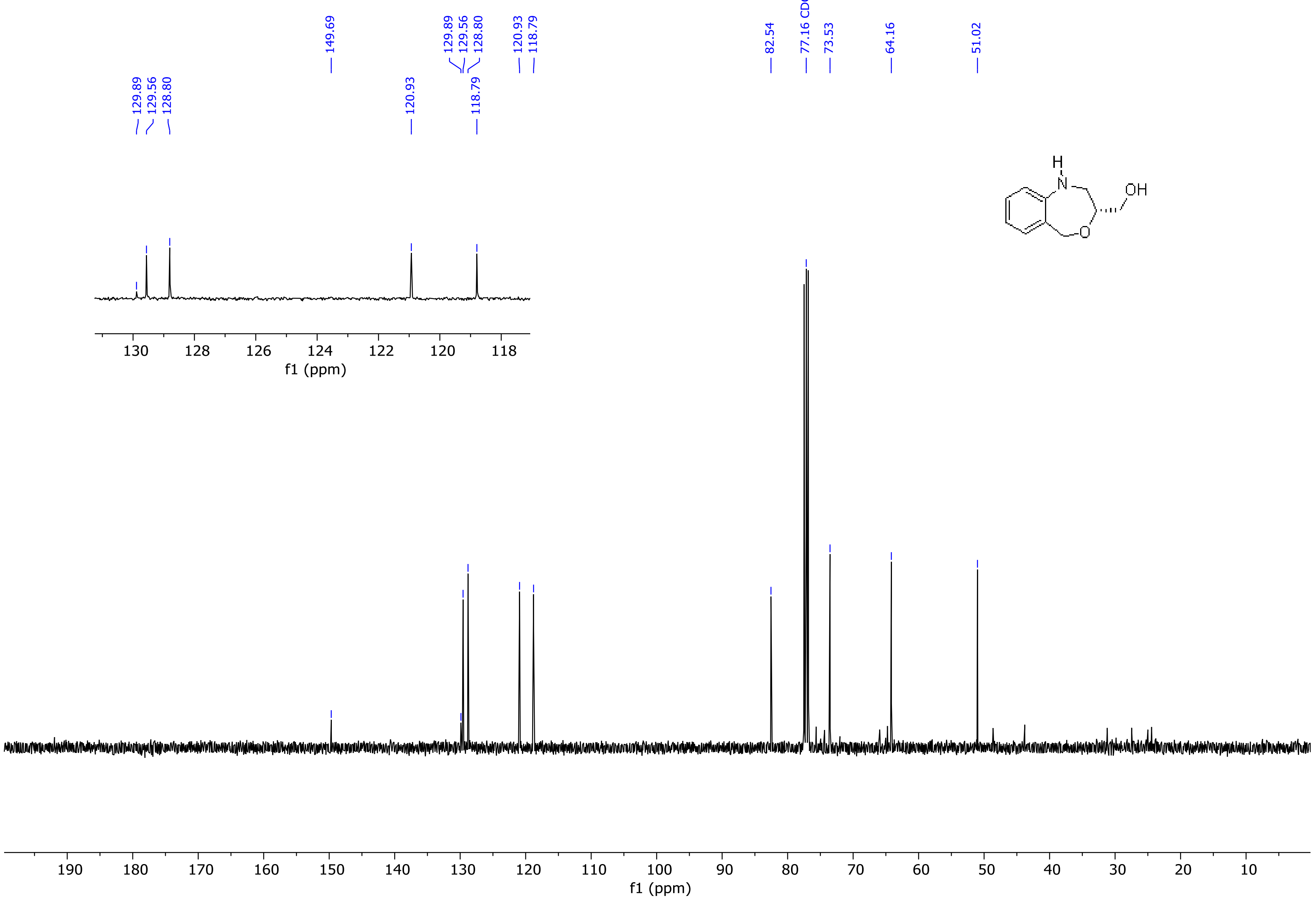

1H NMR: (400 MHz, CDCl3): (R)-(1-Benzyl-1,2,3,5-tetrahydrobenzo[e][1,4]oxazepin-3-yl)methyl 4-methylbenzenesulfonate (4a)

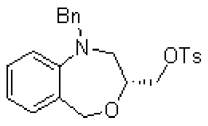

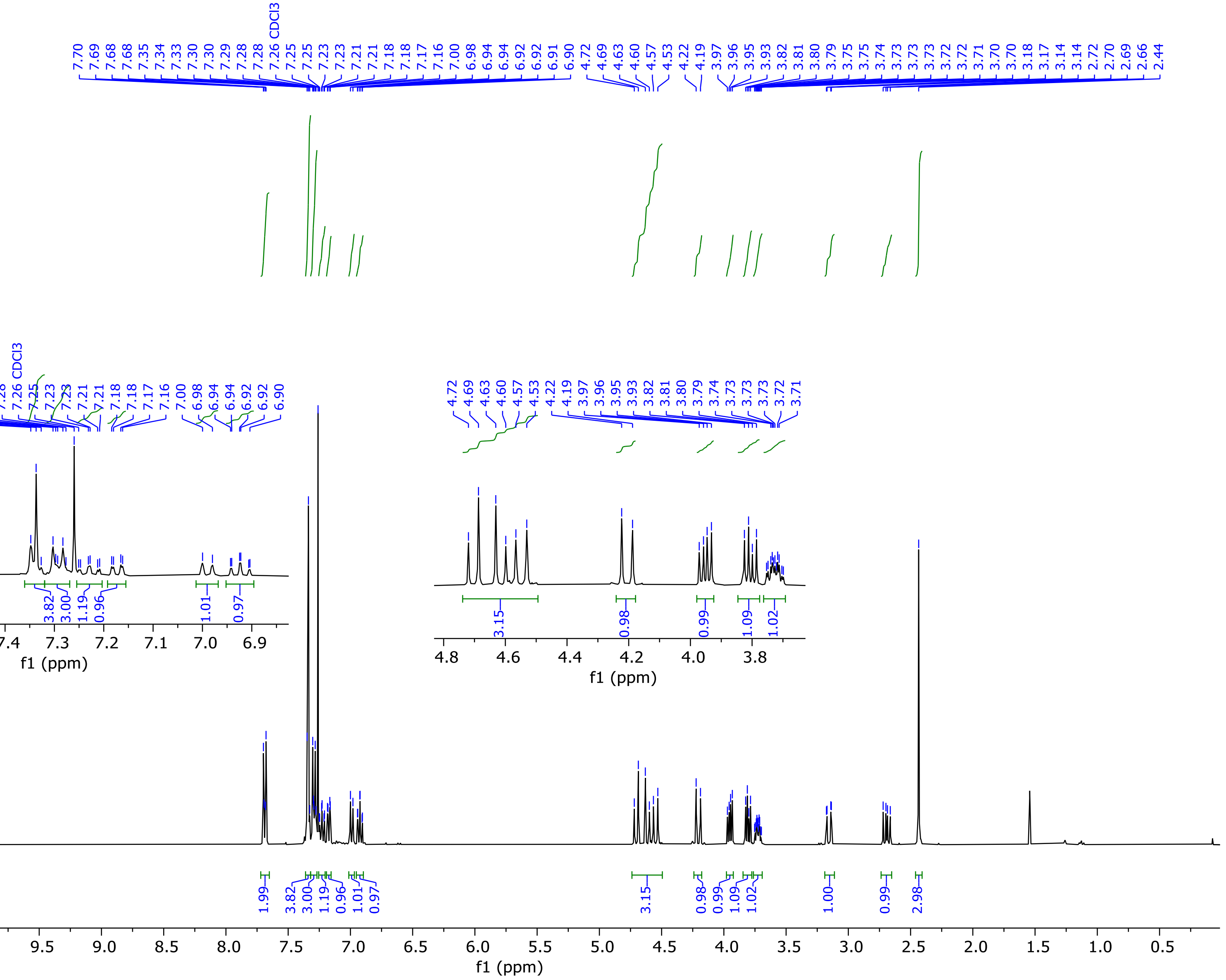

13C{1H}NMR (101 MHz, CDCl3): (R)-(1-Benzyl-1,2,3,5-tetrahydrobenzo[e][1,4]oxazepin-3-yl)methyl 4-methylbenzenesulfonate (4a)

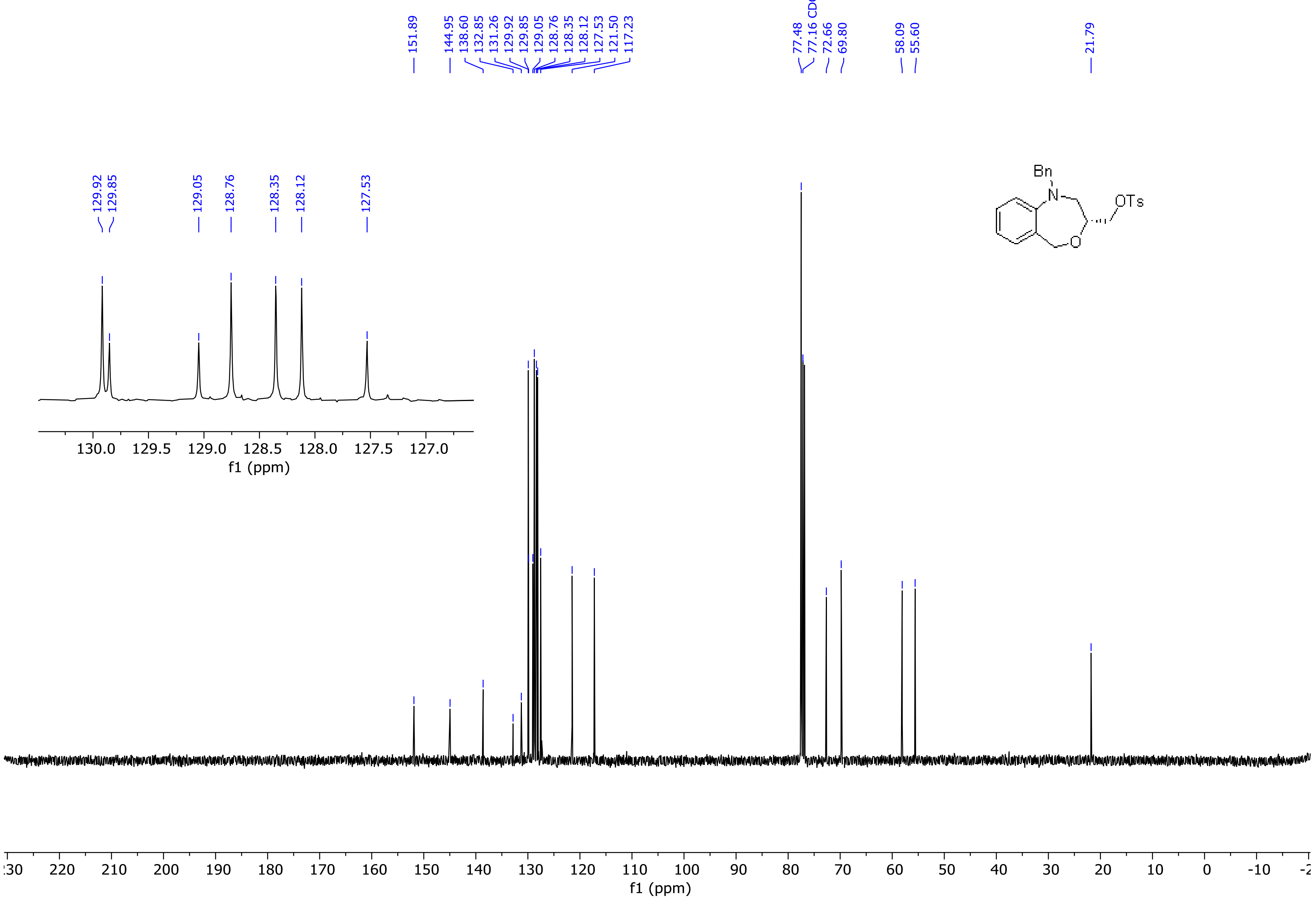

1H NMR: (400 MHz, CDCl3): (R)-3-(Azidomethyl)-1-benzyl-1,2,3,5-tetrahydrobenzo[e][1,4]oxazepine (5a)

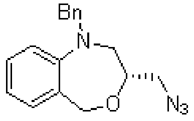

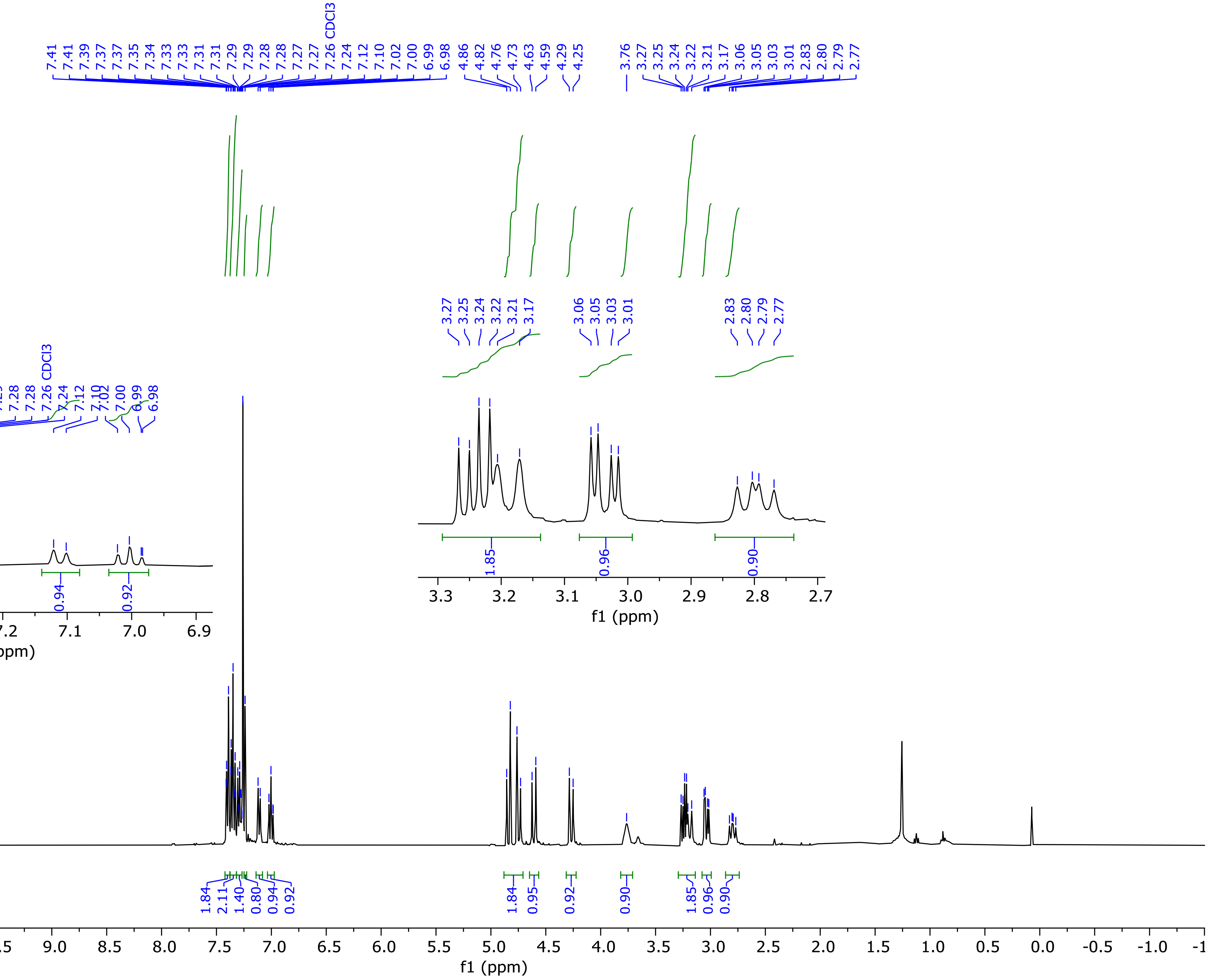

13C{1H}NMR (101 MHz, CDCl3): (R)-3-(Azidomethyl)-1-benzyl-1,2,3,5-tetrahydrobenzo[e][1,4]oxazepine (5a)

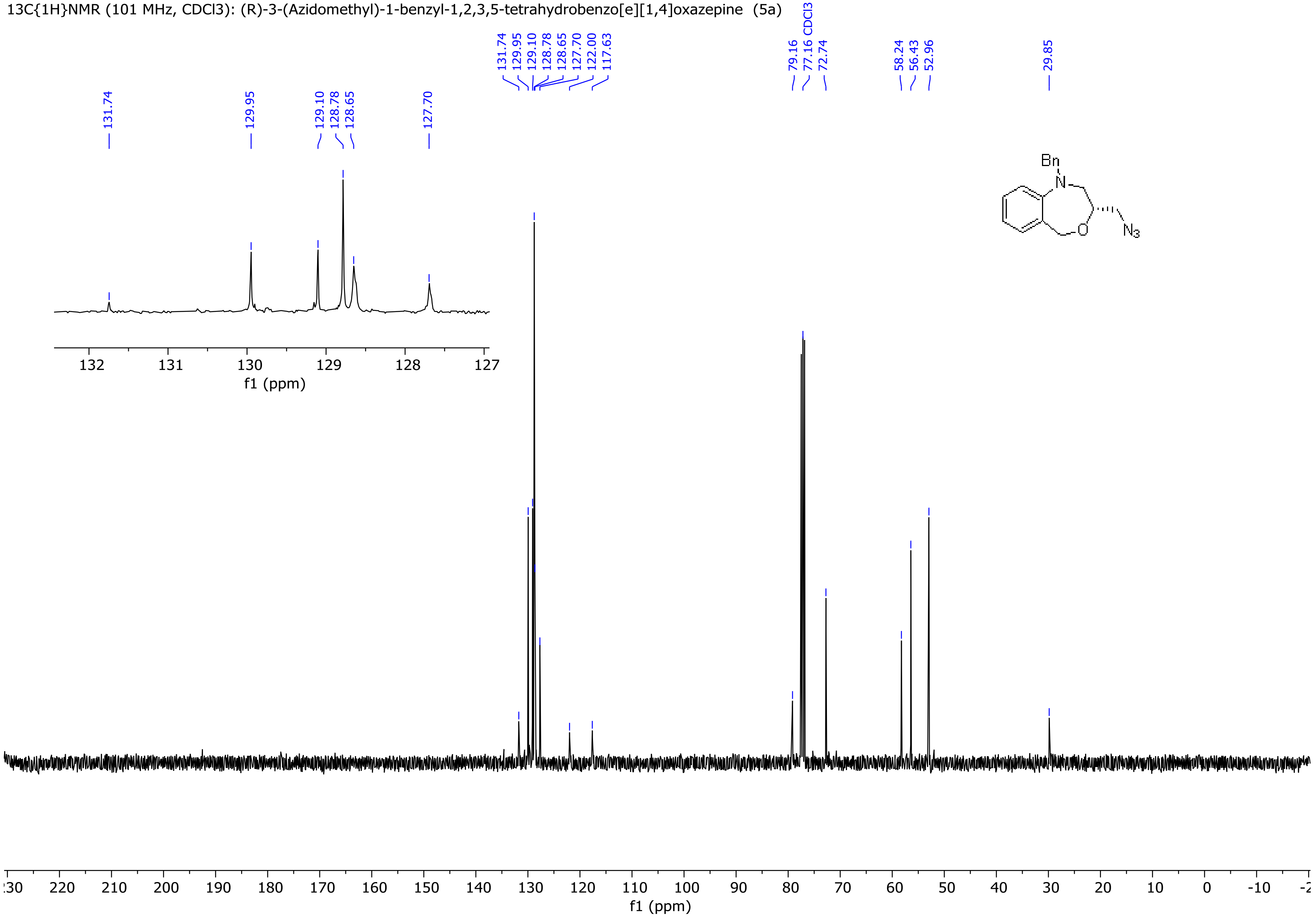

1H NMR: (400 MHz, CDCl3): (R)-1-Benzyl-3-(chloromethyl)-1,2,3,5-tetrahydrobenzo[e][1,4]oxazepine (6a)

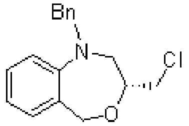

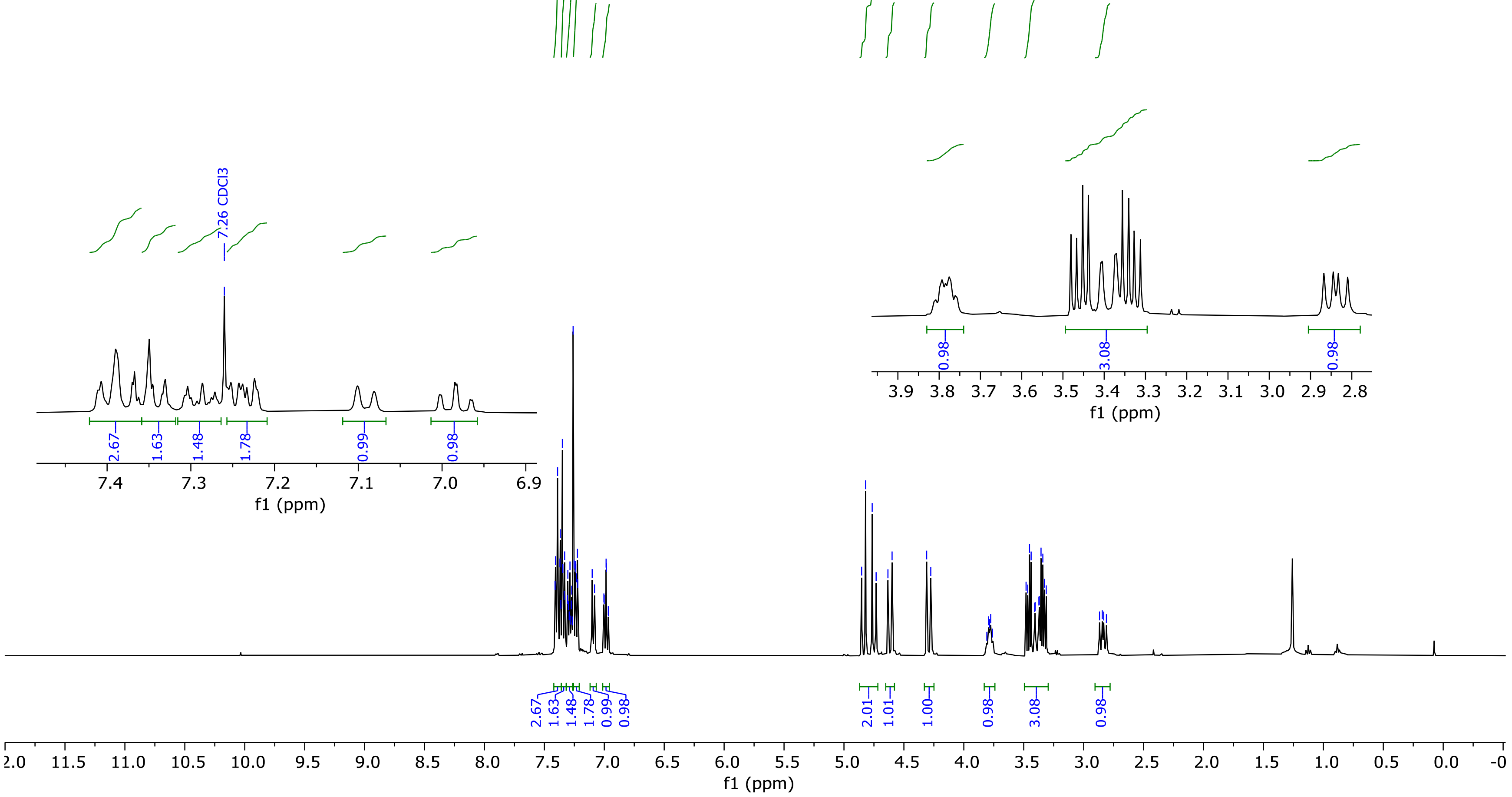

13C{1H}NMR (101 MHz, CDCl3): (R)-1-Benzyl-3-(chloromethyl)-1,2,3,5-tetrahydrobenzo[e][1,4]oxazepine (6a)

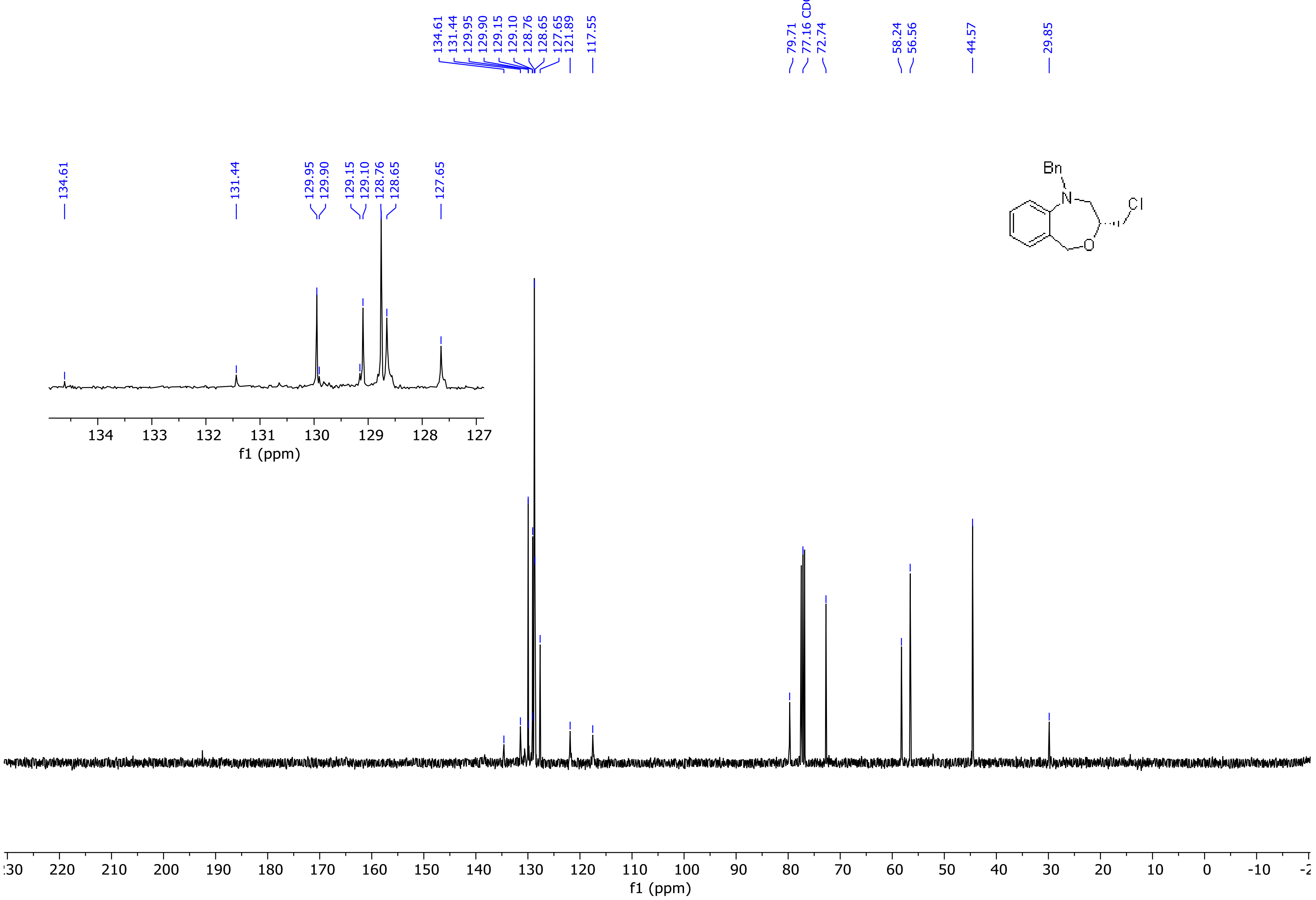

HPLC DATA

(R)-(1-Benzyl-1,2,3,5-tetrahydrobenzo[e][1,4]oxazepin-3-yl)methanol ***(2a)***

Racemate:

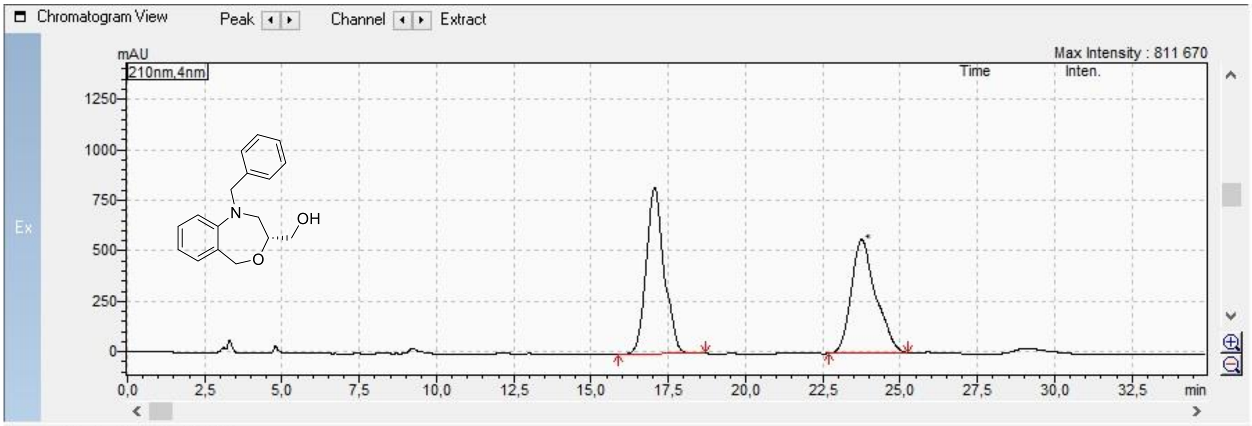

Results View - Peak Table

Peak Table | Compound | Group | Calibration Curve

Peak#	Ret. Time	Area	Height	Peak Start	Peak End	Mark	Conc.	Unit	Area%
1	17,060	33247632	822079	15,893	18,709	M	51,703		51,703
2	23,754	31057932	562458	22,709	25,248	M	48,297		48,297
Total		64305564	1384537				100,000		100,000

Chiral: 92% *e.e.*

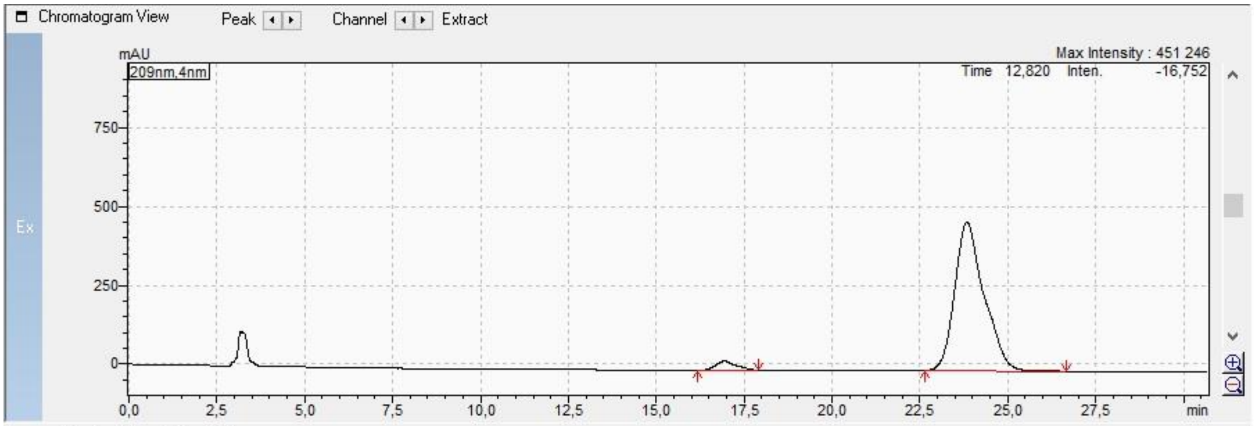

Results View - Peak Table

Peak Table | Compound | Group | Calibration Curve

Peak#	Ret. Time	Area	Height	Peak Start	Peak End	Mark	Conc.	Unit	Area%
1	16,941	1140357	28298	16,171	17,909	M	4,038		4,038
2	23,837	27097703	471424	22,645	26,667	M	95,962		95,962
Total		28238061	499722				100,000		100,000

(R)-(1-(4-Methoxybenzyl)-1,2,3,5-tetrahydrobenzo[e][1,4]oxazepin-3-yl)methanol ***(2b)***

Racemate:

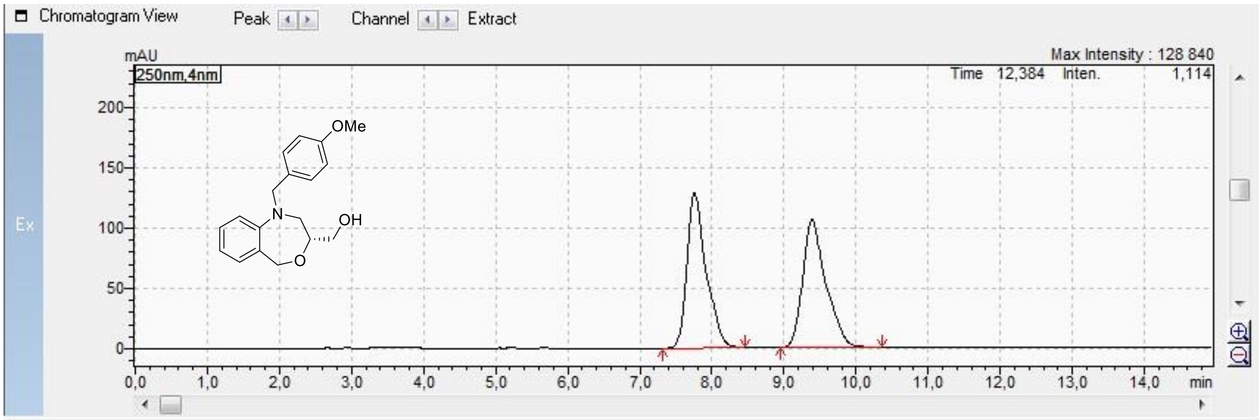

Results View - Peak Table

Peak Table | Compound | Group | Calibration Curve

Peak#	Ret. Time	Conc.	Area	Height	Similarity Index	Mark	Peak Start	Peak End	Area%
1	7,761	48,471	2365577	128013	0,000000	M	7,317	8,459	48,471
2	9,390	48,789	2381065	106036	0,000000	M	8,949	10,357	48,789
3	17,214	2,740	133726	3001	0,000000		16,437	18,304	2,740
Total		100,000	4880368	237050					100,000

Chiral: 88% *e.e.*

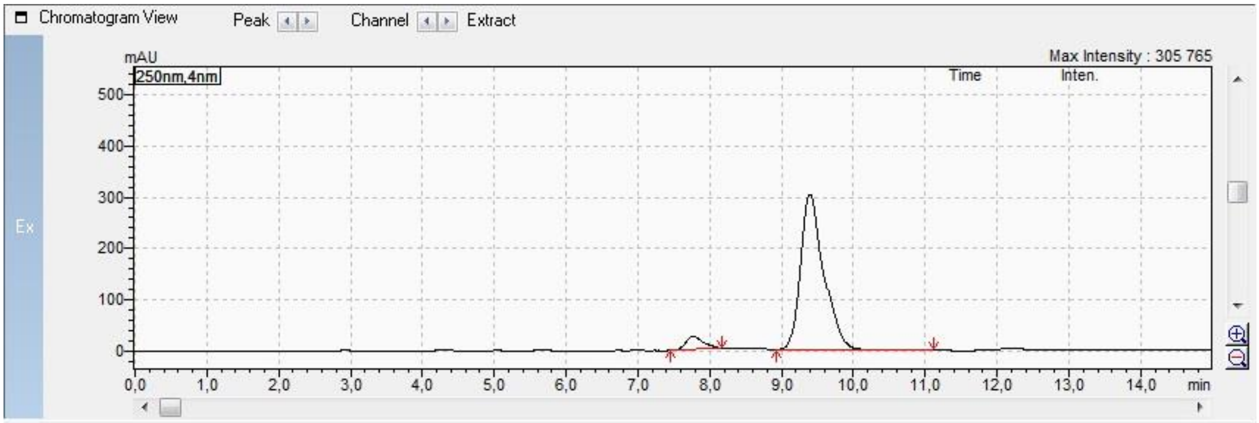

Results View - Peak Table

Peak Table | Compound | Group | Calibration Curve

Peak#	Ret. Time	Conc.	Area	Height	Similarity Index	Mark	Peak Start	Peak End	Area%
1	7,772	6,148	444151	26201	0,000000	M	7,445	8,160	6,148
2	9,395	93,852	6780078	303508	0,000000	M	8,928	11,115	93,852
Total		100,000	7224229	329709					100,000

(R)-(1-(4-Methylbenzyl)-1,2,3,5-tetrahydrobenzo[e][1,4]oxazepin-3-yl)methanol ***(2c)***

Racemate:

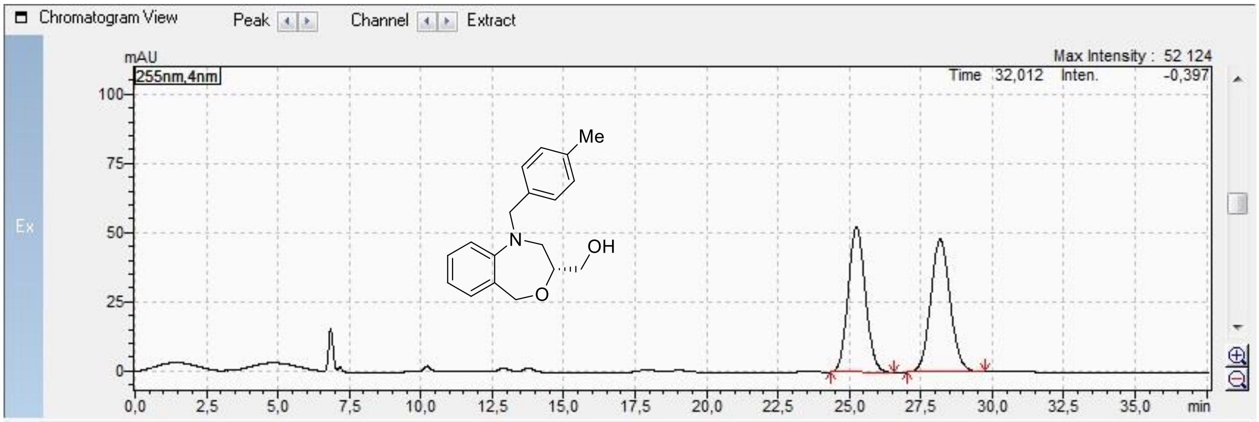

Results View - Peak Table

Peak Table | Compound | Group | Calibration Curve

Peak#	Ret. Time	Conc.	Area	Height	Similarity Index	Mark	Peak Start	Peak End	Area%
1	25,241	49,138	2197755	52526	0,000000	M	24,341	26,560	49,138
2	28,167	50,862	2274884	48009	0,000000	M	27,019	29,749	50,862
Total		100,000	4472639	100535					100,000

Chiral: 88% *e.e.*

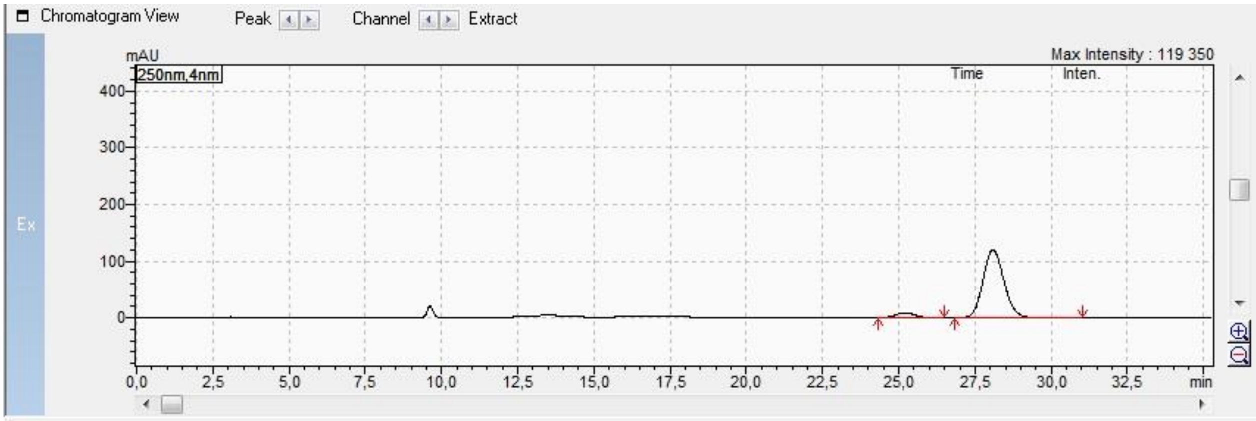

Results View - Peak Table

Peak Table | Compound | Group | Calibration Curve

Peak#	Ret. Time	Conc.	Area	Height	Similarity Index	Mark	Peak Start	Peak End	Area%
1	25,198	6,059	366934	8721	0,000000	M	24,320	26,475	6,059
2	28,085	93,941	5688595	119344	0,000000	M	26,837	31,029	93,941
Total		100,000	6055528	128065					100,000

(R)-(1-(4-Nitrobenzyl)-1,2,3,5-tetrahydrobenzo[e][1,4]oxazepin-3-yl)methanol ***(2d)***

Racemate:

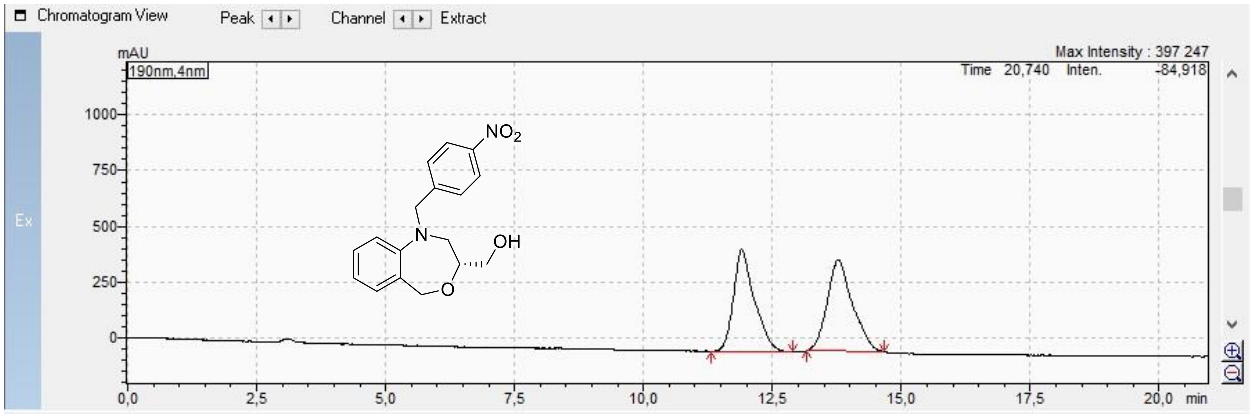

Results View - Peak Table

Peak Table | Compound | Group | Calibration Curve

Peak#	Ret. Time	Area	Height	Peak Start	Peak End	Mark	Conc.	Unit	Area%
1	11,911	13054830	460225	11,307	12,896	M	48,256		48,256
2	13,792	13998288	412713	13,163	14,667	M	51,744		51,744
Total		27053118	872938				100,000		100,000

Chiral: 94% *e.e.*

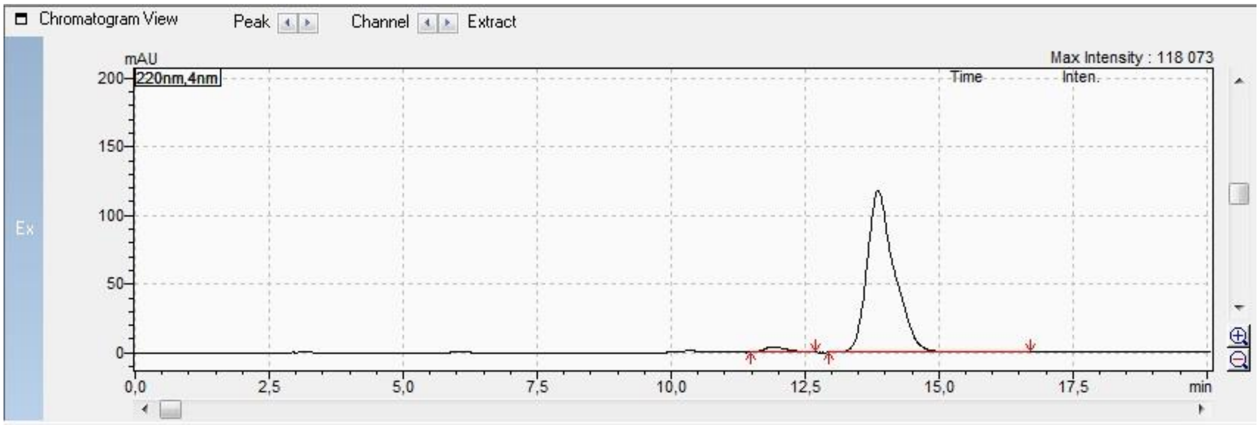

Results View - Peak Table

Peak Table | Compound | Group | Calibration Curve

Peak#	Ret. Time	Conc.	Area	Height	Similarity Index	Mark	Peak Start	Peak End	Area%
1	11,908	2,806	120564	4197	0,000000	M	11,499	12,693	2,806
2	13,865	97,194	4176602	117759	0,000000	M	12,949	16,715	97,194
Total		100,000	4297165	121956					100,000

(R)-4-((3-(Hydroxymethyl)-2,3-dihydrobenzo[e][1,4]oxazepin-1(5H)-yl)methyl)benzonitrile ***(2e)***

Racemate:

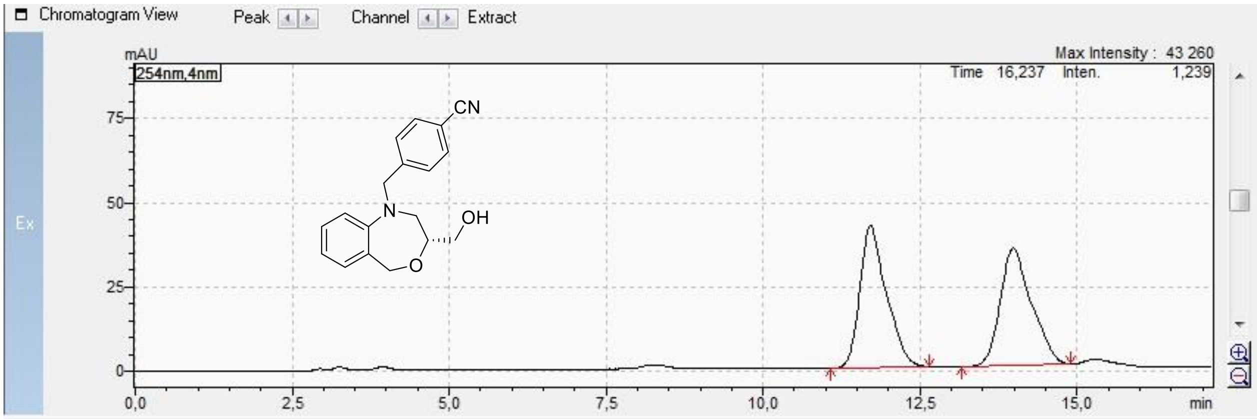

Results View - Peak Table

Peak Table | Compound | Group | Calibration Curve

Peak#	Ret. Time	Conc.	Area	Height	Similarity Index	Mark	Peak Start	Peak End	Area%
1	11,712	50,688	1239520	42151	0,000000	M	11,072	12,651	50,688
2	13,985	49,312	1205887	34839	0,000000	M	13,163	14,901	49,312
Total		100,000	2445407	76990					100,000

Chiral: 93% *e.e.*

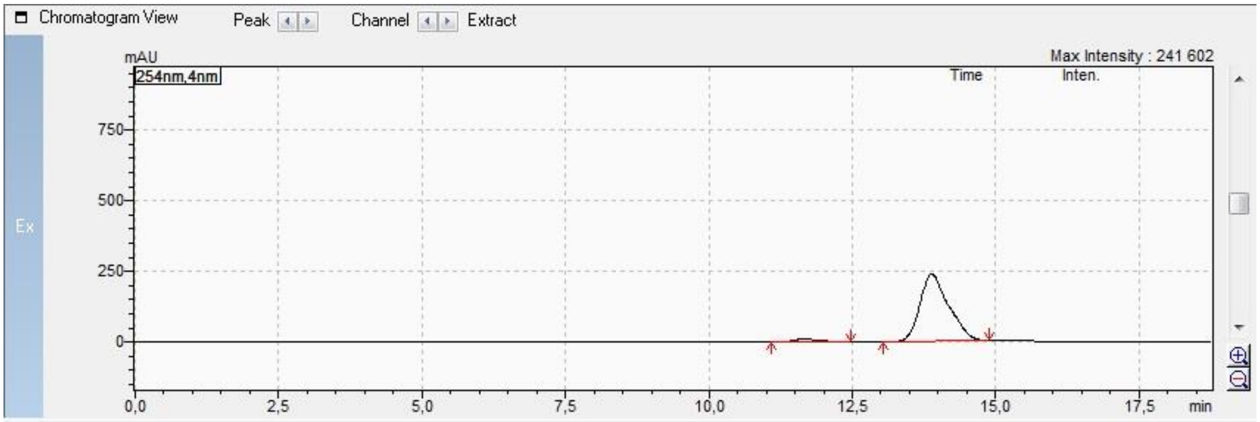

Results View - Peak Table

Peak Table | Compound | Group | Calibration Curve

Peak#	Ret. Time	Conc.	Area	Height	Similarity Index	Mark	Peak Start	Peak End	Area%
1	11,660	3,663	315807	10779	0,000000	M	11,093	12,469	3,663
2	13,885	96,337	8306501	238646	0,000000	M	13,035	14,891	96,337
Total		100,000	8622308	249425					100,000

(R)-(1-(4-(Trifluoromethyl)benzyl)-1,2,3,5-tetrahydrobenzo[e][1,4]oxazepin-3-yl)methanol ***(2f)***

Racemate:

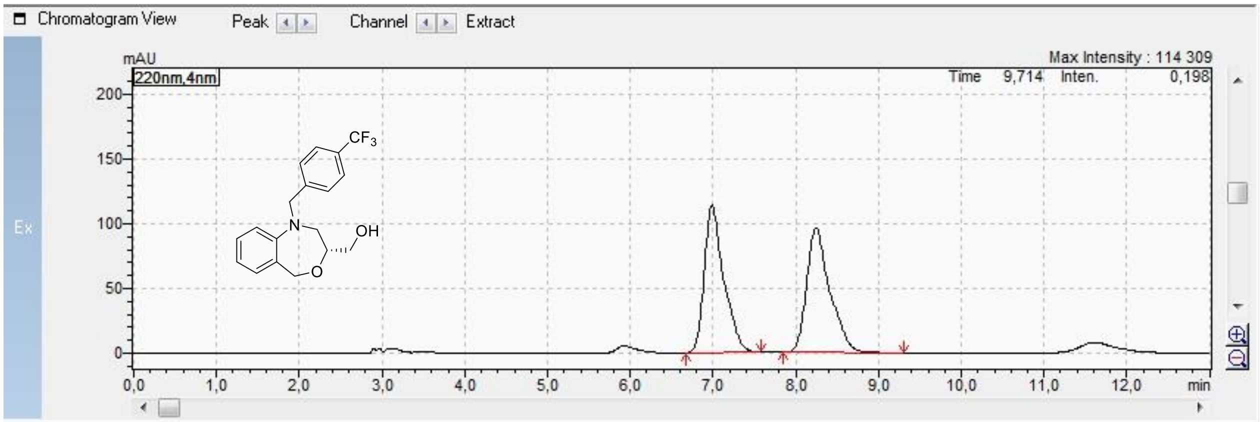

Results View - Peak Table

Peak Table | Compound | Group | Calibration Curve

Peak#	Ret. Time	Conc.	Area	Height	Similarity Index	Mark	Peak Start	Peak End	Area%
1	6,990	49,855	1866673	113911	0,000000	M	6,667	7,584	49,855
2	8,244	50,145	1877528	96581	0,000000	M	7,840	9,312	50,145
Total		100,000	3744201	210492					100,000

Chiral: 91% *e.e.*

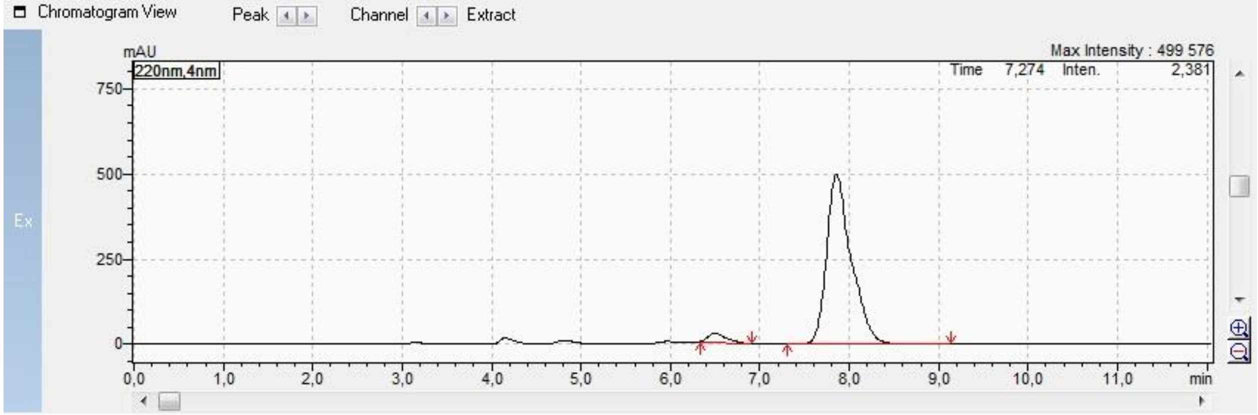

Results View - Peak Table

Peak Table | Compound | Group | Calibration Curve

Peak#	Ret. Time	Conc.	Area	Height	Similarity Index	Mark	Peak Start	Peak End	Area%
1	6,502	3,936	387000	27926	0,000000	M	6,325	6,912	3,936
2	7,856	96,064	9446404	497534	0,000000	M	7,307	9,131	96,064
Total		100,000	9833404	525460					100,000

(R)-(1-(4-Fluorobenzyl)-1,2,3,5-tetrahydrobenzo[e][1,4]oxazepin-3-yl)methanol ***(2g)***

Racemate:

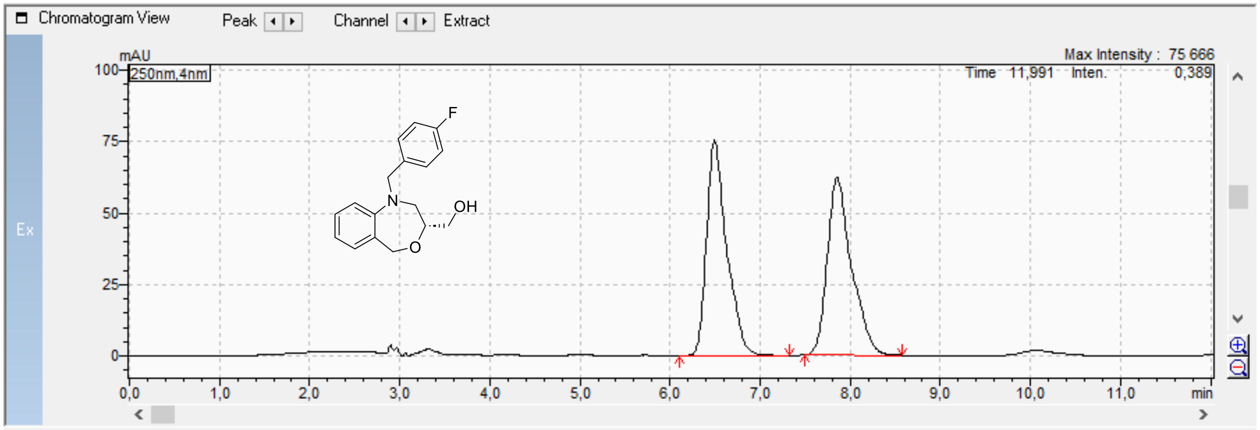

Results View - Peak Table

Peak Table | Compound | Group | Calibration Curve

Peak#	Ret. Time	Area	Height	Peak Start	Peak End	Mark	Conc.	Unit	Area%
1	6,494	1168752	75539	6,101	7,317	M	50,147		50,147
2	7,854	1161890	62220	7,499	8,576	M	49,853		49,853
Total		2330642	137758				100,000		100,000

Chiral: 92% *e.e.*

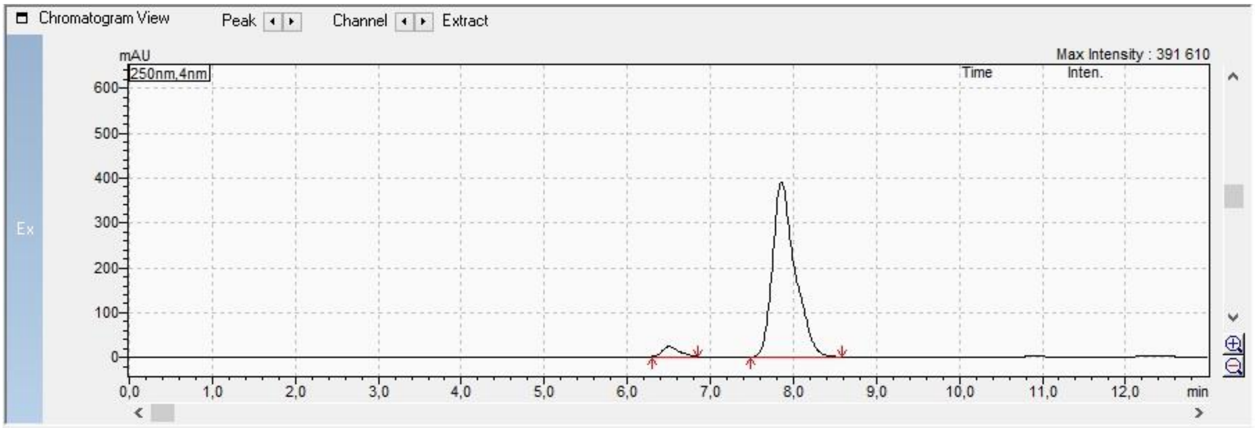

Results View - Peak Table

Peak Table | Compound | Group | Calibration Curve

Peak#	Ret. Time	Area	Height	Peak Start	Peak End	Mark	Conc.	Unit	Area%
1	6,504	325094	22679	6,293	6,848	M	4,264		4,264
2	7,857	7299128	389843	7,488	8,587	M	95,736		95,736
Total		7624221	412522				100,000		100,000

(R)-(1-(4-Bromobenzyl)-1,2,3,5-tetrahydrobenzo[e][1,4]oxazepin-3-yl)methanol ***(2h)***

Racemate:

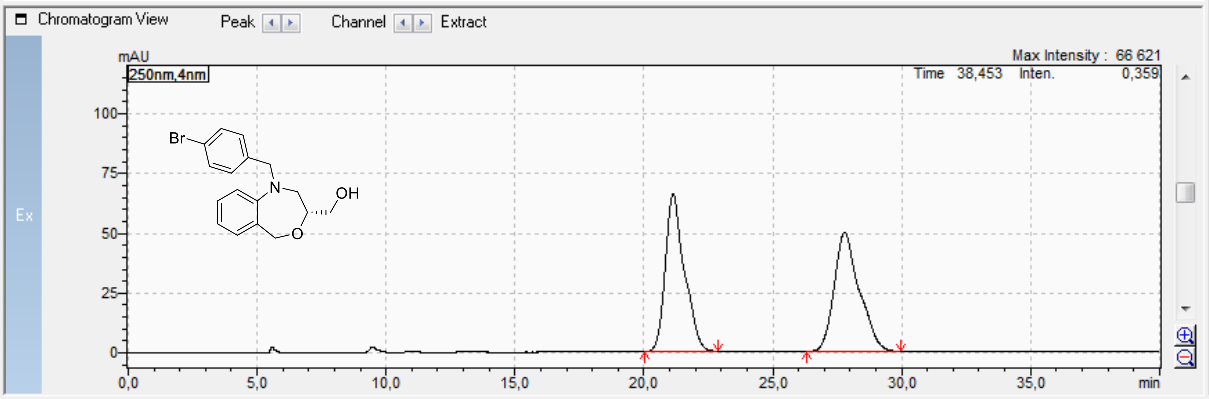

Results View - Peak Table

Peak Table | Compound | Group | Calibration Curve

Peak#	Ret. Time	Conc.	Area	Height	Similarity Index	Mark	Peak Start	Peak End	Area%
1	21,140	50,045	3382380	65972	0,000000		20,043	22,869	50,045
2	27,792	49,955	3376232	49896	0,000000		26,336	29,984	49,955
Total		100,000	6758612	115868					100,000

Chiral: 94% *e.e.*

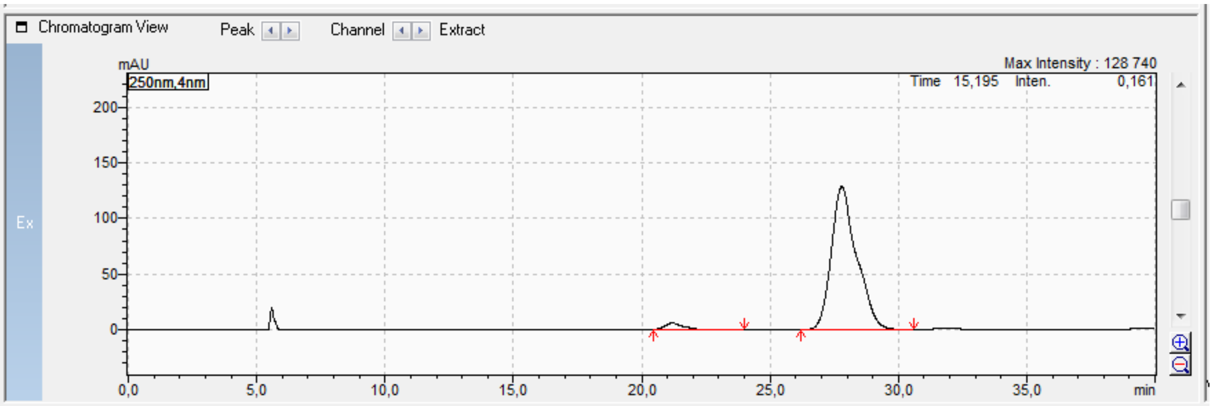

Results View - Peak Table

Peak Table | Compound | Group | Calibration Curve

Peak#	Ret. Time	Conc.	Area	Height	Similarity Index	Mark	Peak Start	Peak End	Area%
1	21,189	3,095	281925	5785	0,000000	M	20,459	24,000	3,095
2	27,796	96,905	8827184	128665	0,000000	M	26,197	30,603	96,905
Total		100,000	9109109	134450					100,000

(R)-(1-(3-Bromobenzyl)-1,2,3,5-tetrahydrobenzo[e][1,4]oxazepin-3-yl)methanol ***(2i)***

Racemate:

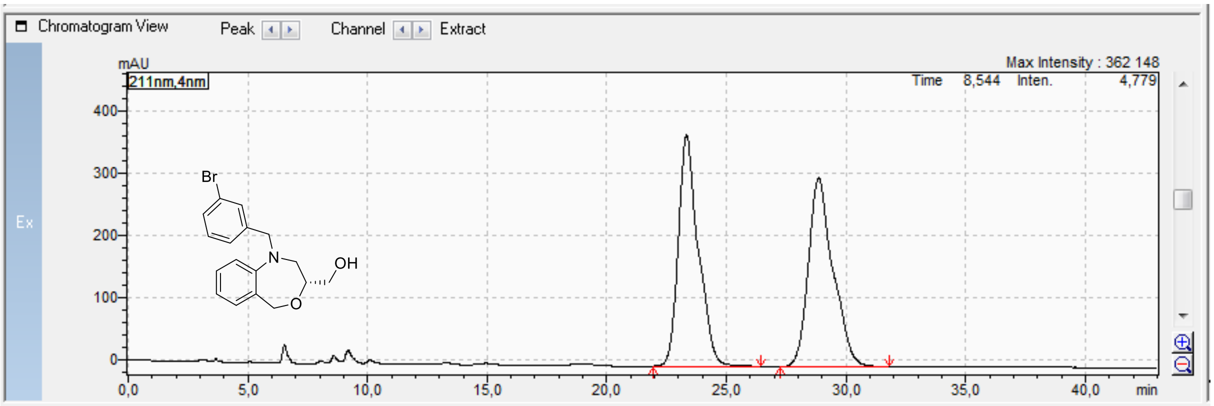

Results View - Peak Table

Peak Table | Compound | Group | Calibration Curve

Peak#	Ret. Time	Conc.	Area	Height	Similarity Index	Mark	Peak Start	Peak End	Area%
1	23,334	49,901	21598753	372091	0,000000	M	21,941	26,443	49,901
2	28,850	50,099	21684712	303698	0,000000	M	27,253	31,808	50,099
Total		100,000	43283465	675789					100,000

Chiral: 92% *e.e.*

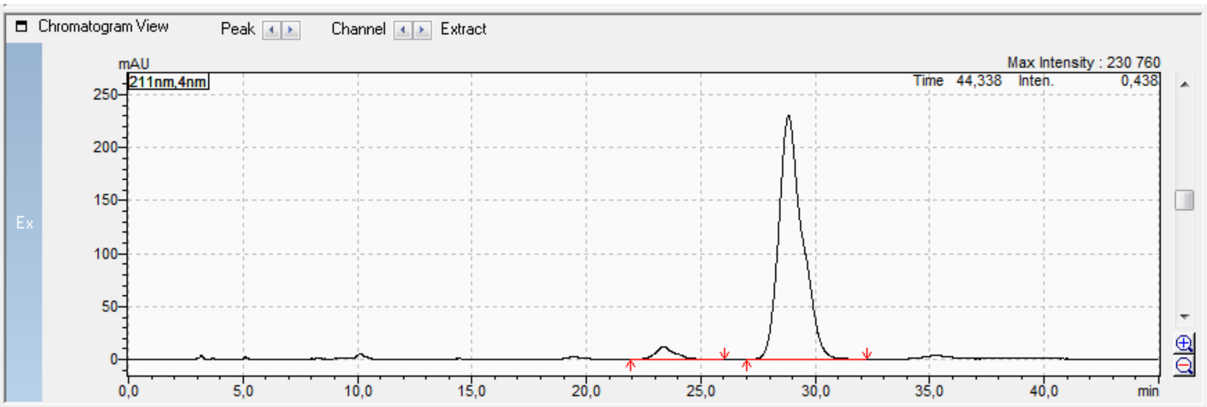

Results View - Peak Table

Peak Table | Compound | Group | Calibration Curve

Peak#	Ret. Time	Conc.	Area	Height	Similarity Index	Mark	Peak Start	Peak End	Area%
1	23,354	3,967	676462	11481	0,000000	M	21,931	26,037	3,967
2	28,832	96,033	16374632	230464	0,000000	M	26,997	32,256	96,033
Total		100,000	17051094	241945					100,000

(R)-(1-(2-Bromobenzyl)-1,2,3,5-tetrahydrobenzo[e][1,4]oxazepin-3-yl)methanol **(2j)**

Racemate:

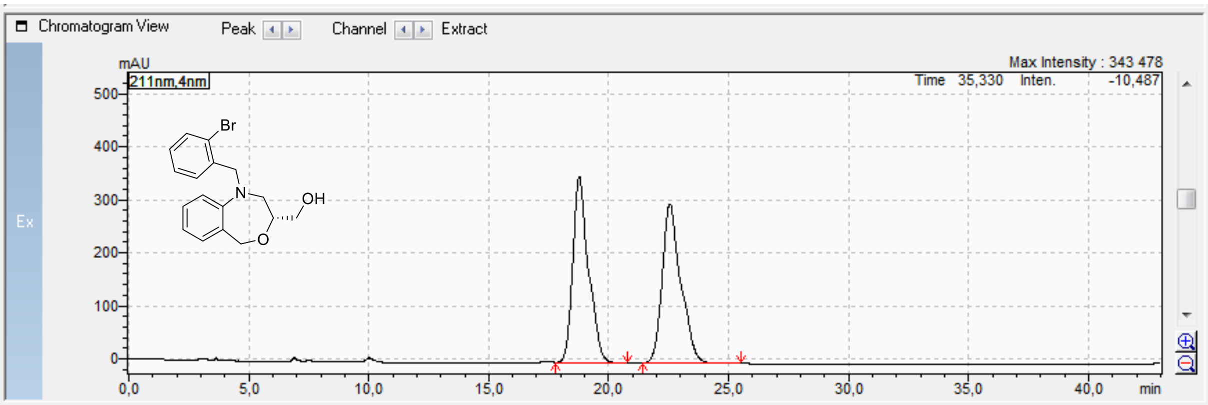

Results View - Peak Table

Peak Table | Compound | Group | Calibration Curve

Peak#	Ret. Time	Conc.	Area	Height	Similarity Index	Mark	Peak Start	Peak End	Area%
1	18,773	49,936	16119055	351766	0,000000		17,803	20,768	49,936
2	22,553	50,064	16160634	300547	0,000000	M	21,419	25,515	50,064
Total		100,000	32279689	652313					100,000

Chiral: 90% *e.e.*

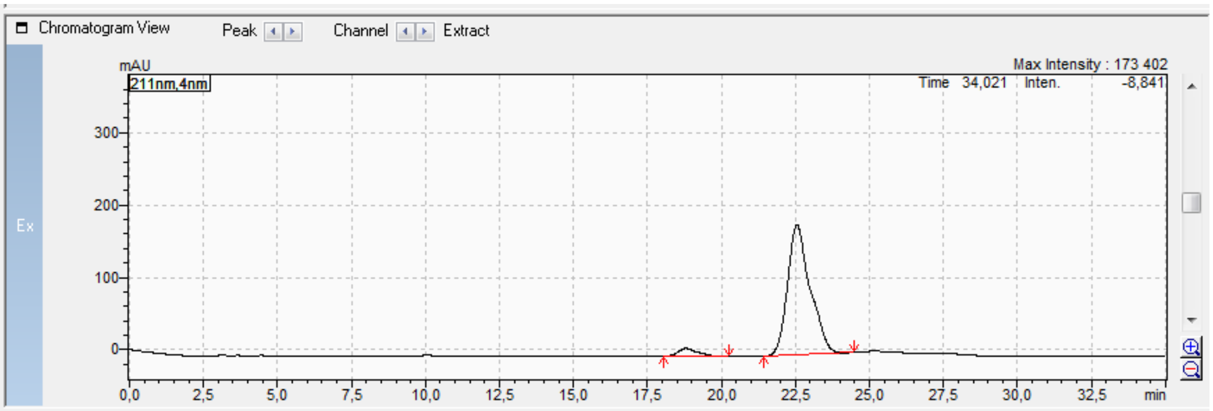

Results View - Peak Table

Peak Table | Compound | Group | Calibration Curve

Peak#	Ret. Time	Conc.	Area	Height	Similarity Index	Mark	Peak Start	Peak End	Area%
1	18,811	4,906	489682	10867	0,000000	M	18,048	20,256	4,906
2	22,551	95,094	9492030	180145	0,000000	M	21,429	24,480	95,094
Total		100,000	9981712	191012					100,000

(R)-(1-(2-Nitrobenzyl)-1,2,3,5-tetrahydrobenzo[e][1,4]oxazepin-3-yl)methanol ***(2k)***

Racemate:

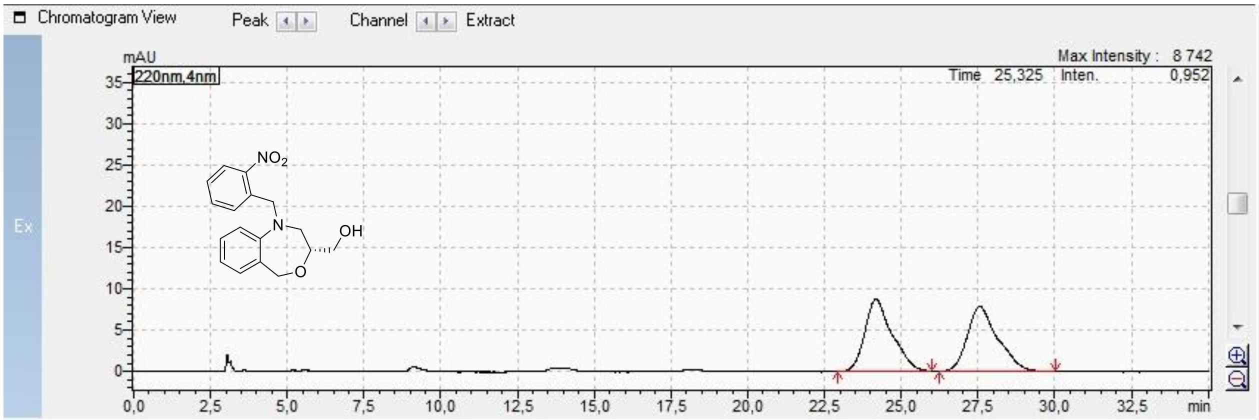

Results View - Peak Table

Peak Table | Compound | Group | Calibration Curve

Peak#	Ret. Time	Conc.	Area	Height	Similarity Index	Mark	Peak Start	Peak End	Area%
1	24,185	50,002	540099	8742	0,000000	M	22,923	25,995	50,002
2	27,562	49,998	540065	7824	0,000000	M	26,240	30,027	49,998
Total		100,000	1080164	16566					100,000

Chiral: 88% *e.e.*

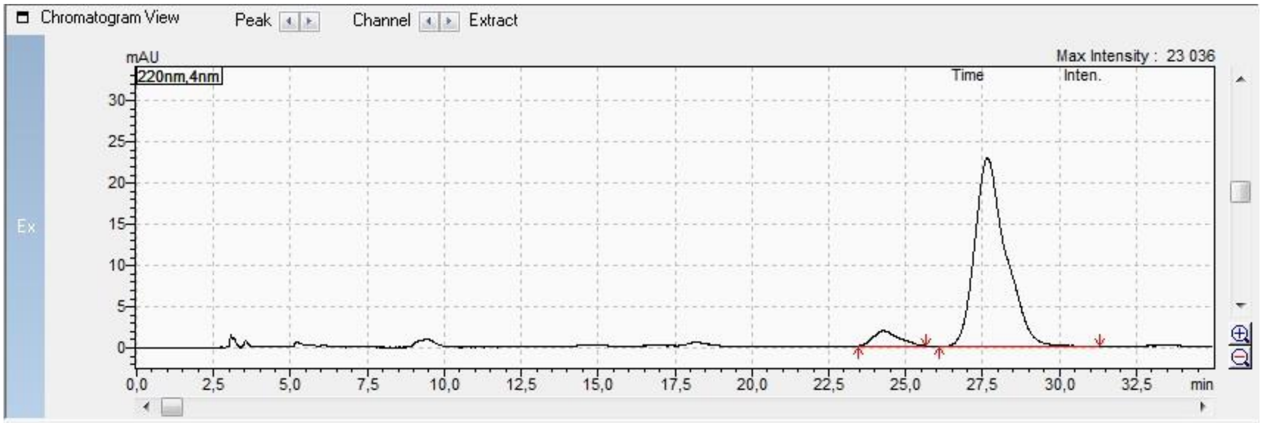

Results View - Peak Table

Peak Table | Compound | Group | Calibration Curve

Peak#	Ret. Time	Conc.	Area	Height	Similarity Index	Mark	Peak Start	Peak End	Area%
1	24,291	6,218	105907	1819	0,000000	M	23,467	25,664	6,218
2	27,667	93,782	1597282	22827	0,000000	M	26,091	31,296	93,782
Total		100,000	1703189	24647					100,000

(R)-(1-(Naphthalen-2-ylmethyl)-1,2,3,5-tetrahydrobenzo[e][1,4]oxazepin-3-yl)methanol ***(2l)***

Racemate:

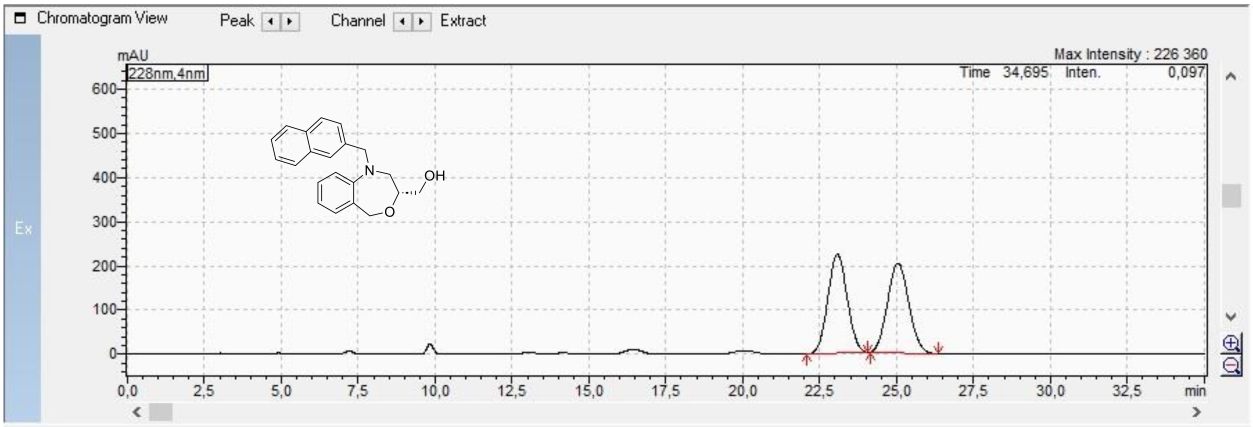

Results View - Peak Table

Peak Table | Compound | Group | Calibration Curve

Peak#	Ret. Time	Area	Height	Peak Start	Peak End	Mark	Conc.	Unit	Area%
1	23,076	9830282	224076	22,080	24,064	M	50,084		50,084
2	25,042	9797117	202748	24,149	26,357	M	49,916		49,916
Total		19627398	426824				100,000		100,000

Chiral: 92% *e.e.*

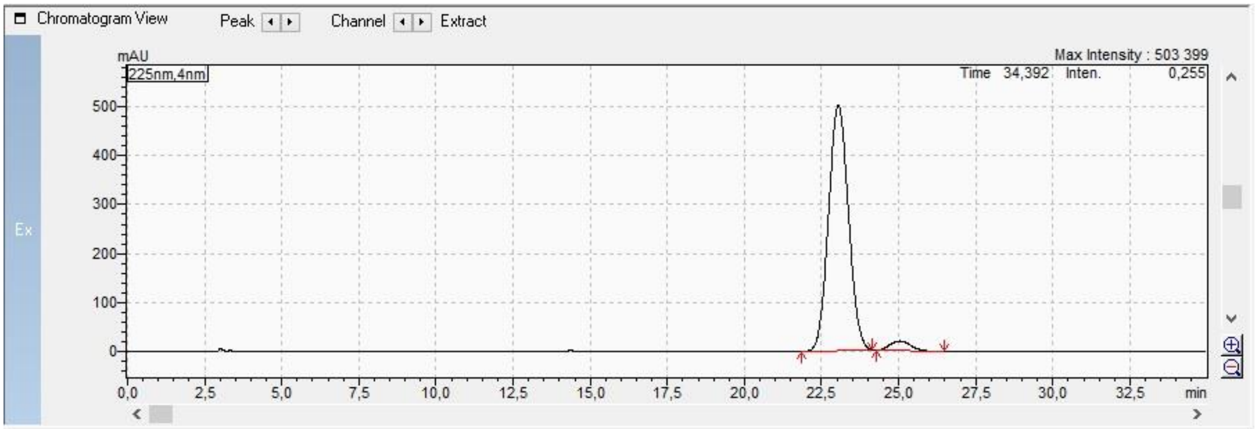

Results View - Peak Table

Peak Table | Compound | Group | Calibration Curve

Peak#	Ret. Time	Area	Height	Peak Start	Peak End	Mark	Conc.	Unit	Area%
1	23,044	22505608	501452	21,845	24,139	M	96,065		96,065
2	25,028	921760	19723	24,277	26,485	M	3,935		3,935
Total		23427368	521175				100,000		100,000

(R)-(1-(Thiophen-2-ylmethyl)-1,2,3,5-tetrahydrobenzo[e][1,4]oxazepin-3-yl)methanol ***(2m)***

Racemate:

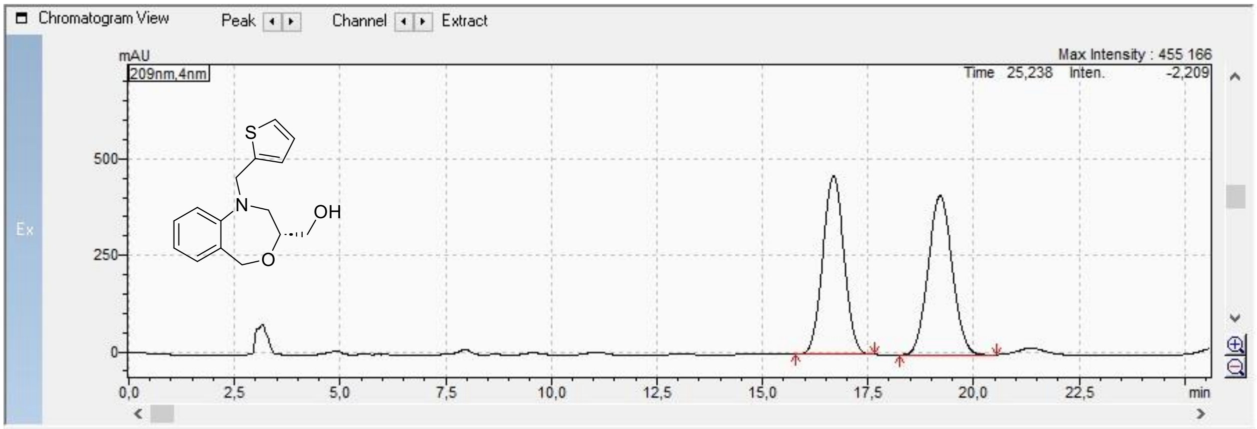

Results View - Peak Table

Peak#	Ret. Time	Area	Height	Peak Start	Peak End	Mark	Conc.	Unit	Area%
1	16,684	16142230	461084	15,787	17,653	M	49,726		49,726
2	19,209	16319912	411526	18,251	20,544	M	50,274		50,274
Total		32462142	872610				100,000		100,000

Chiral: 90% *e.e.*

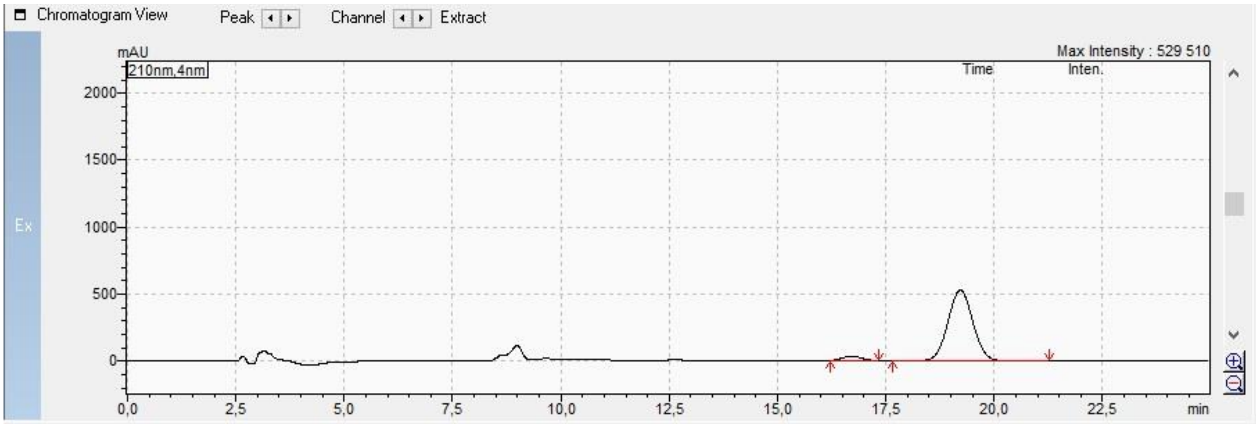

Results View - Peak Table

Peak#	Ret. Time	Area	Height	Peak Start	Peak End	Mark	Conc.	Unit	Area%
1	16,705	1047951	32812	16,213	17,333	M	4,754		4,754
2	19,216	20997099	526012	17,653	21,269	M	95,246		95,246
Total		22045049	558824				100,000		100,000

(R)-(1-Methyl-1,2,3,5-tetrahydrobenzo[e][1,4]oxazepin-3-yl)methanol ***(2o)***

Racemate:

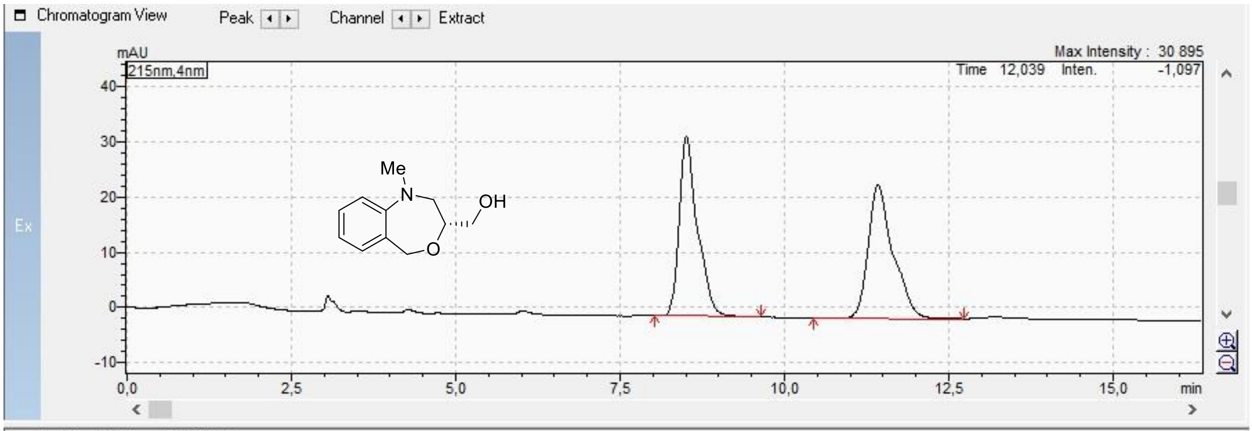

Results View - Peak Table

Peak Table | Compound | Group | Calibration Curve

Peak#	Ret. Time	Area	Height	Peak Start	Peak End	Mark	Conc.	Unit	Area%
1	8,507	631472	32431	8,021	9,653	M	49,793		49,793
2	11,426	636730	24130	10,443	12,736	M	50,207		50,207
Total		1268202	56561				100,000		100,000

Chiral: 65% *e.e.*

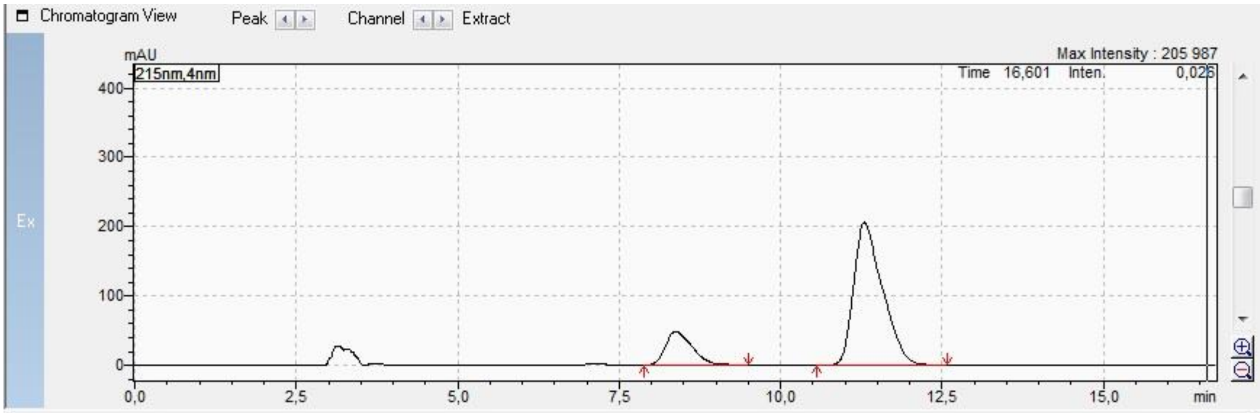

Results View - Peak Table

Peak Table | Compound | Group | Calibration Curve

Peak#	Ret. Time	Conc.	Area	Height	Similarity Index	Mark	Peak Start	Peak End	Area%
1	8,374	17,781	1381362	48228	0,000000	M	7,883	9,504	17,781
2	11,299	82,219	6387239	205725	0,000000	M	10,560	12,576	82,219
Total		100,000	7768600	253953					100,000

(R)-(1-Phenyl-1,2,3,5-tetrahydrobenzo[e][1,4]oxazepin-3-yl)methanol ***(2q)***

Racemate:

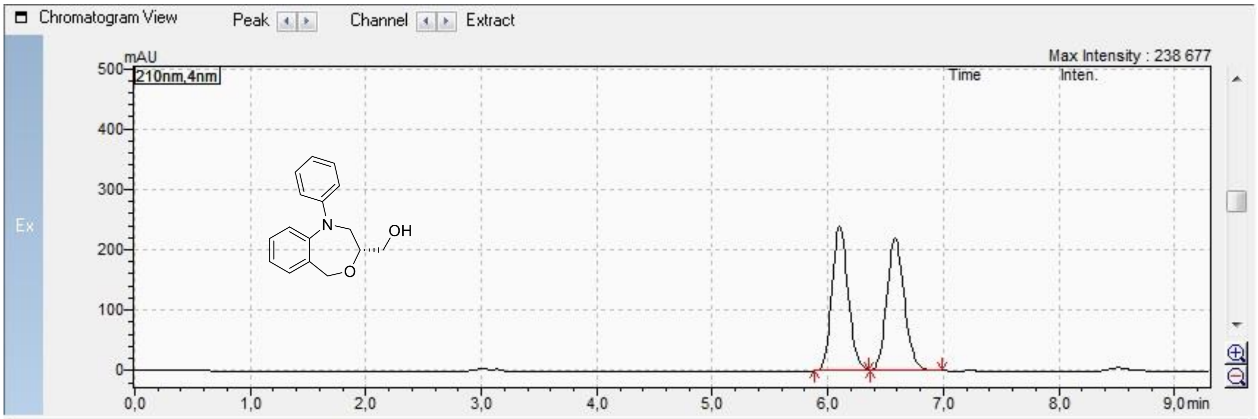

Results View - Peak Table

Peak Table | Compound | Group | Calibration Curve

Peak#	Ret. Time	Conc.	Area	Height	Similarity Index	Mark	Peak Start	Peak End	Area%
1	6,101	49,906	2294251	237945	0,000000	M	5,888	6,357	49,906
2	6,582	50,094	2302887	217534	0,000000	M	6,368	6,987	50,094
Total		100,000	4597138	455480					100,000

Chiral: 90% *e.e.*

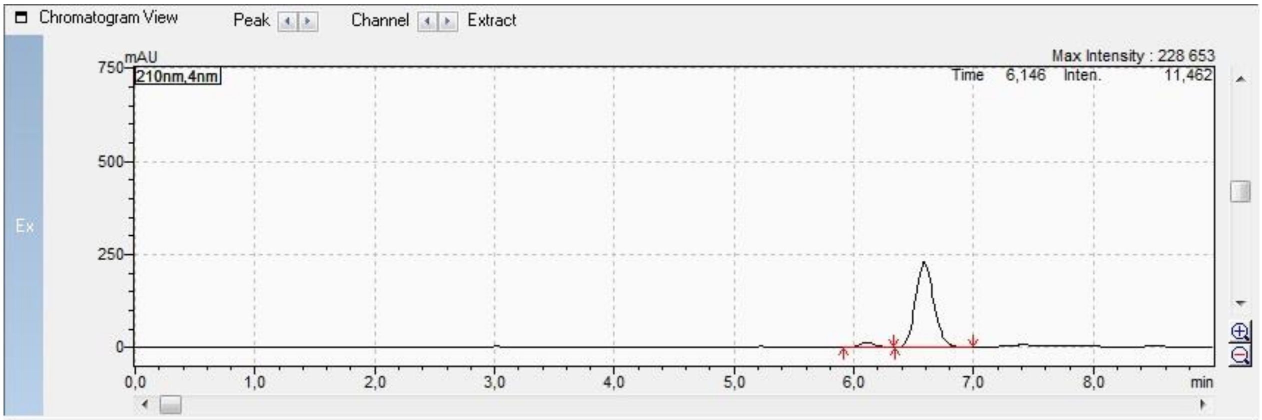

Results View - Peak Table

Peak Table | Compound | Group | Calibration Curve

Peak#	Ret. Time	Conc.	Area	Height	Similarity Index	Mark	Peak Start	Peak End	Area%
1	6,107	4,807	123642	12955	0,000000	M	5,909	6,336	4,807
2	6,587	95,193	2448488	227680	0,000000	M	6,347	6,997	95,193
Total		100,000	2572130	240634					100,000

(R)-(1-(4-Methoxyphenyl)-1,2,3,5-tetrahydrobenzo[e][1,4]oxazepin-3-yl)methanol ***(2r)***

Racemate:

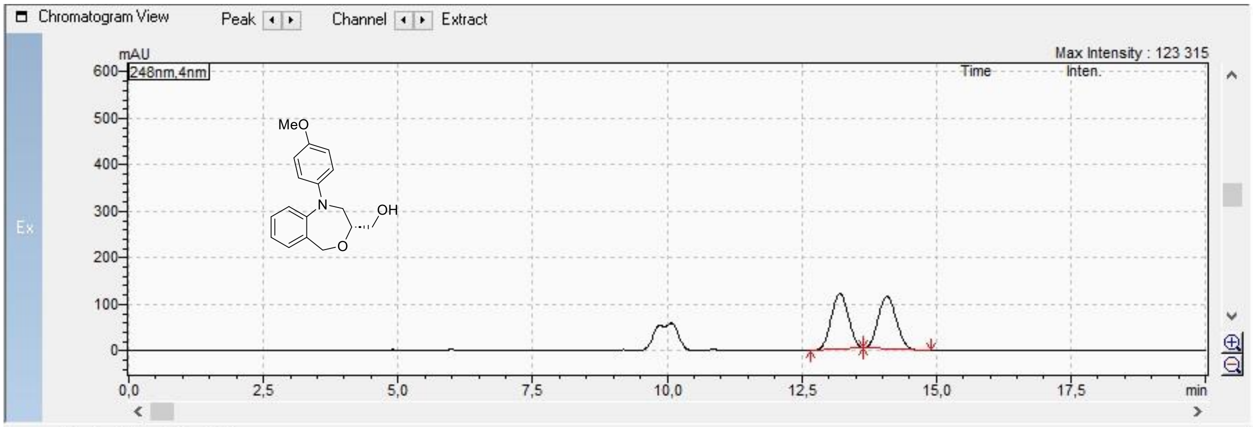

Results View - Peak Table

Peak Table | Compound | Group | Calibration Curve

Peak#	Ret. Time	Area	Height	Peak Start	Peak End	Mark	Conc.	Unit	Area%
1	13,207	2682366	119850	12,661	13,643	M	50,314		50,314
2	14,083	2648860	113267	13,643	14,901	M	49,686		49,686
Total		5331226	233117				100,000		100,000

Chiral: 94% *e.e.*

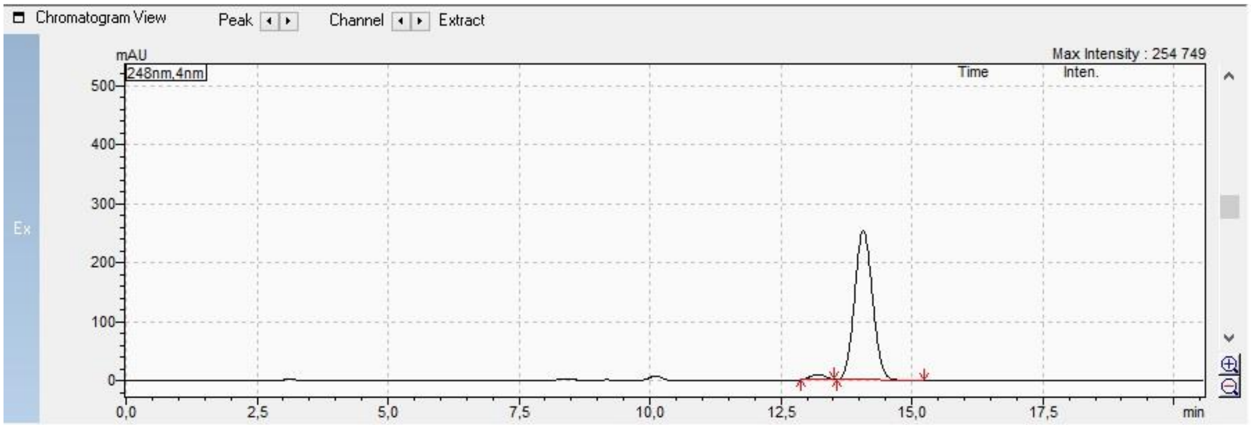

Results View - Peak Table

Peak Table | Compound | Group | Calibration Curve

Peak#	Ret. Time	Area	Height	Peak Start	Peak End	Mark	Conc.	Unit	Area%
1	13,207	182660	9084	12,885	13,525	M	2,919		2,919
2	14,070	6074878	253261	13,579	15,221	M	97,081		97,081
Total		6257539	262344				100,000		100,000

(R)-(1-benzyl-9-methyl-1,2,3,5-tetrahydrobenzo[e][1,4]oxazepin-3-yl)methanol ***(2v)***

Racemate:

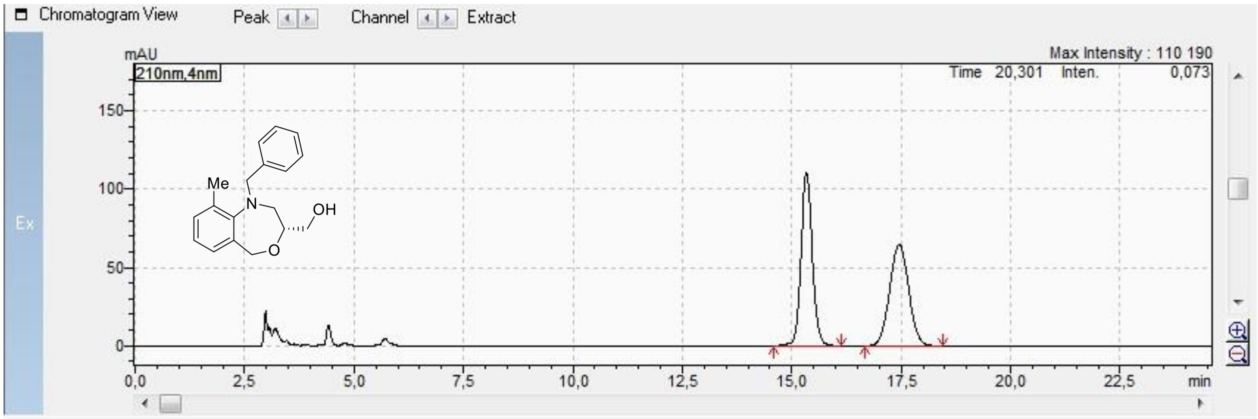

Results View - Peak Table

Peak Table | Compound | Group | Calibration Curve

Peak#	Ret. Time	Conc.	Area	Height	Similarity Index	Mark	Peak Start	Peak End	Area%
1	15,336	50,319	1986624	110138	0,000000	M	14,592	16,128	50,319
2	17,457	49,681	1961469	64983	0,000000	M	16,672	18,453	49,681
Total		100,000	3948093	175122					100,000

Chiral: 88% *e.e.*

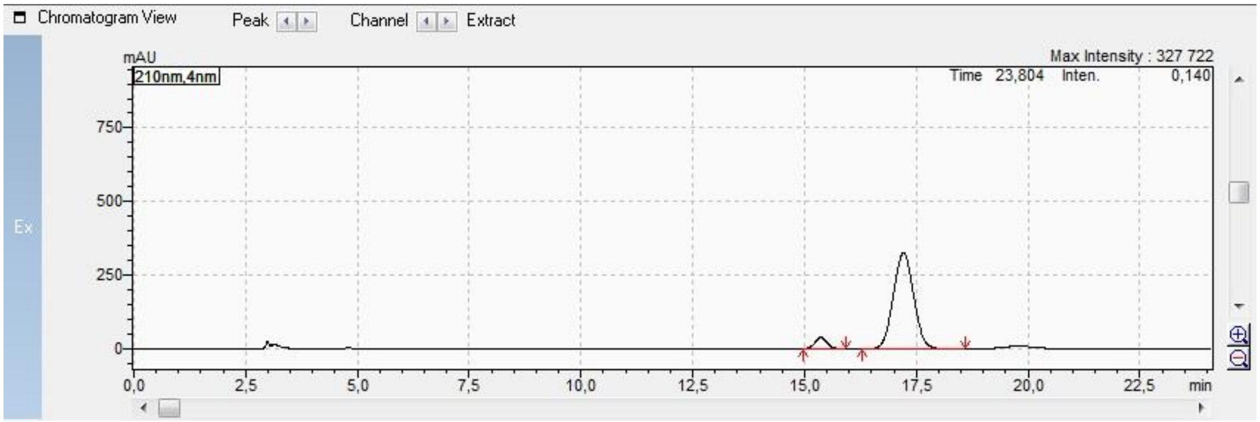

Results View - Peak Table

Peak Table | Compound | Group | Calibration Curve

Peak#	Ret. Time	Conc.	Area	Height	Similarity Index	Mark	Peak Start	Peak End	Area%
1	15,361	6,180	677547	37212	0,000000	M	14,976	15,936	6,180
2	17,215	93,820	10285750	327624	0,000000	M	16,288	18,603	93,820
Total		100,000	10963297	364836					100,000

(R)-(1-Benzyl-8-chloro-1,2,3,5-tetrahydrobenzo[e][1,4]oxazepin-3-yl)methanol ***(2w)***

Racemate:

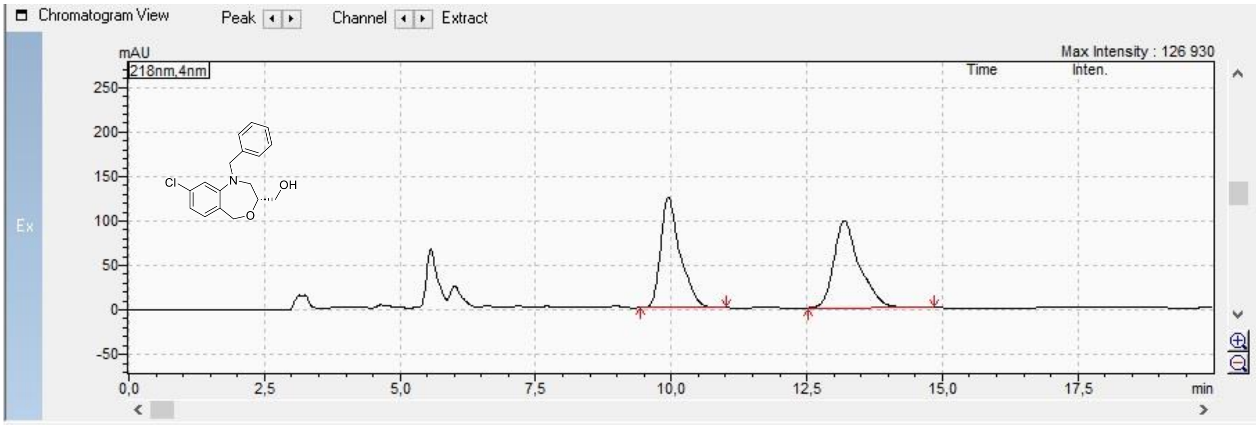

Results View - Peak Table

Peak Table | Compound | Group | Calibration Curve

Peak#	Ret. Time	Area	Height	Peak Start	Peak End	Mark	Conc.	Unit	Area%
1	9,949	3103121	124324	9,429	11,019	M	48,904		48,904
2	13,192	3242188	98149	12,523	14,848	M	51,096		51,096
Total		6345309	222473				100,000		100,000

Chiral: 90% *e.e.*

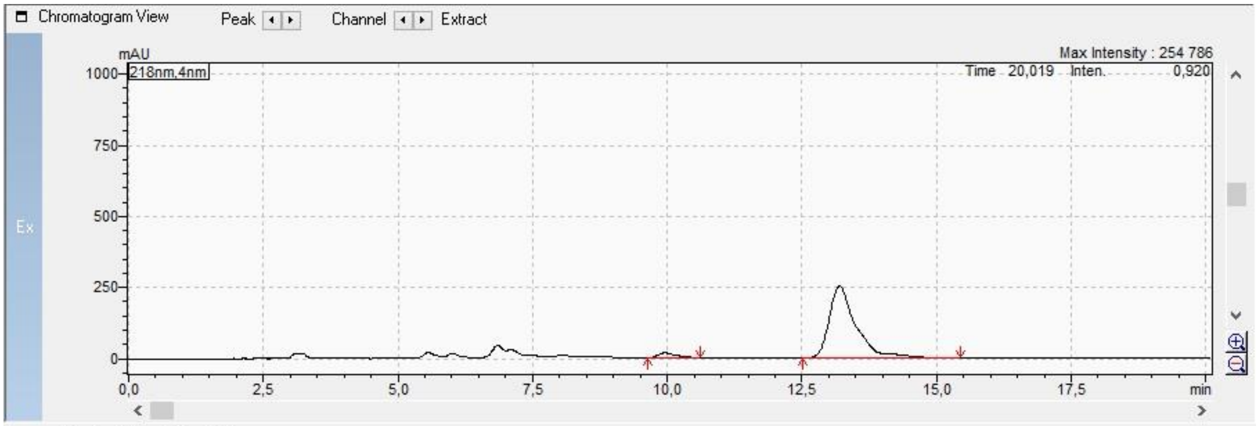

Results View - Peak Table

Peak Table | Compound | Group | Calibration Curve

Peak#	Ret. Time	Area	Height	Peak Start	Peak End	Mark	Conc.	Unit	Area%
1	9,959	441448	18193	9,632	10,613	M	4,780		4,780
2	13,198	8793405	253304	12,512	15,445	M	95,220		95,220
Total		9234853	271498				100,000		100,000

(R)-(1-Benzyl-8-fluoro-1,2,3,5-tetrahydrobenzo[e][1,4]oxazepin-3-yl)methanol (**2x**)

Racemate:

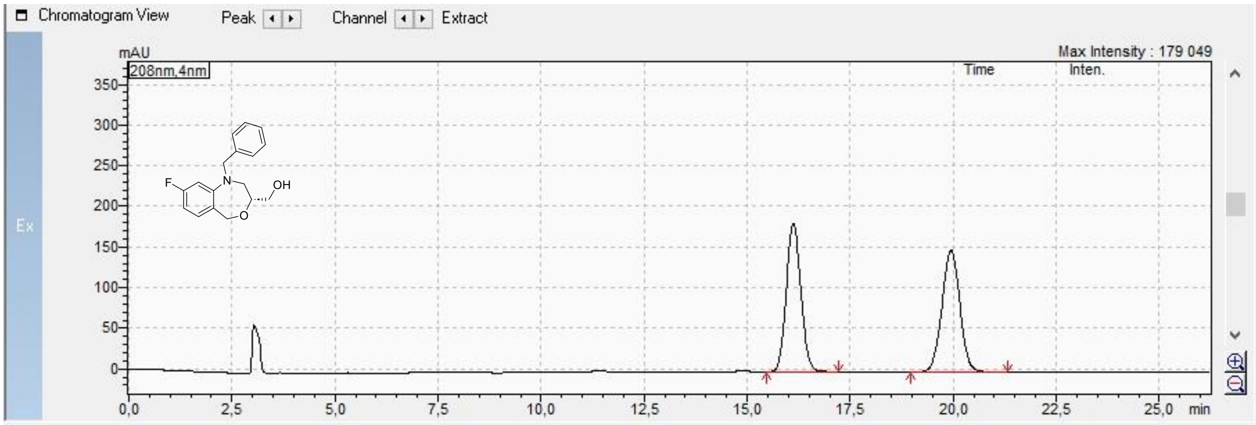

Results View - Peak Table

Peak Table | Compound | Group | Calibration Curve

Peak#	Ret. Time	Area	Height	Peak Start	Peak End	Mark	Conc.	Unit	Area%
1	16,119	4509070	182545	15,467	17,216	M	49,897		49,897
2	19,948	4527688	149883	18,965	21,323	M	50,103		50,103
Total		9036758	332428				100,000		100,000

Chiral: 91% *e.e.*

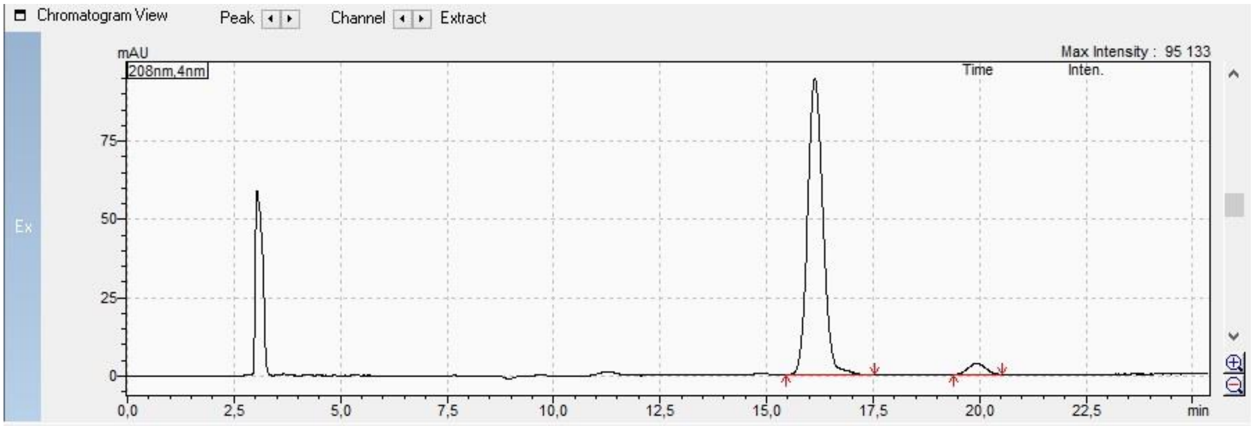

Results View - Peak Table

Peak Table | Compound | Group | Calibration Curve

Peak#	Ret. Time	Area	Height	Peak Start	Peak End	Mark	Conc.	Unit	Area%
1	16,137	2356797	94698	15,456	17,536	M	95,766		95,766
2	19,910	104200	3581	19,392	20,533	M	4,234		4,234
Total		2460998	98278				100,000		100,000

(R)-(1-Benzyl-8-fluoro-1,2,3,5-tetrahydrobenzo[e][1,4]oxazepin-3-yl)methanol ***(2y)***

Racemate:

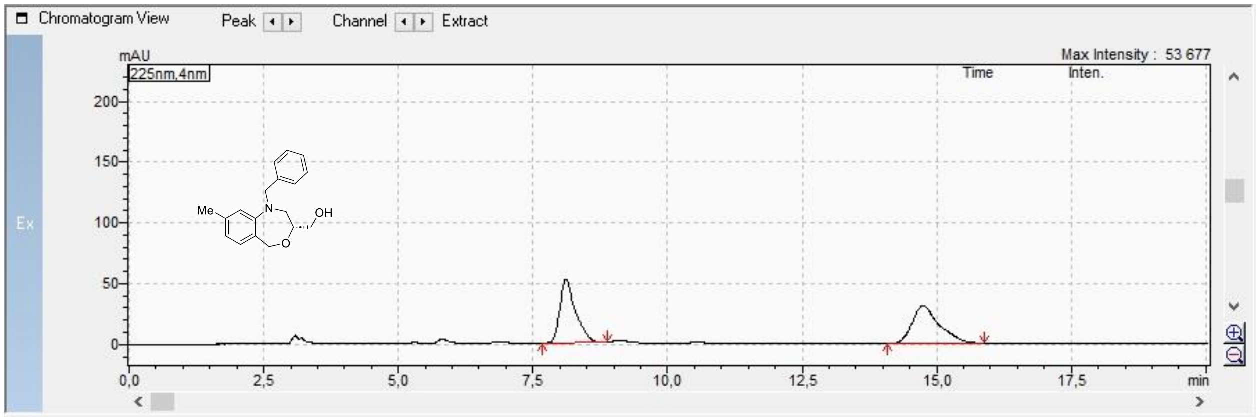

Results View - Peak Table

Peak Table | Compound | Group | Calibration Curve

Peak#	Ret. Time	Area	Height	Peak Start	Peak End	Mark	Conc.	Unit	Area%
1	8,116	1060461	52395	7,669	8,885	M	49,286		49,286
2	14,747	1091197	31128	14,080	15,883	M	50,714		50,714
Total		2151658	83523				100,000		100,000

Chiral: 92% *e.e.*

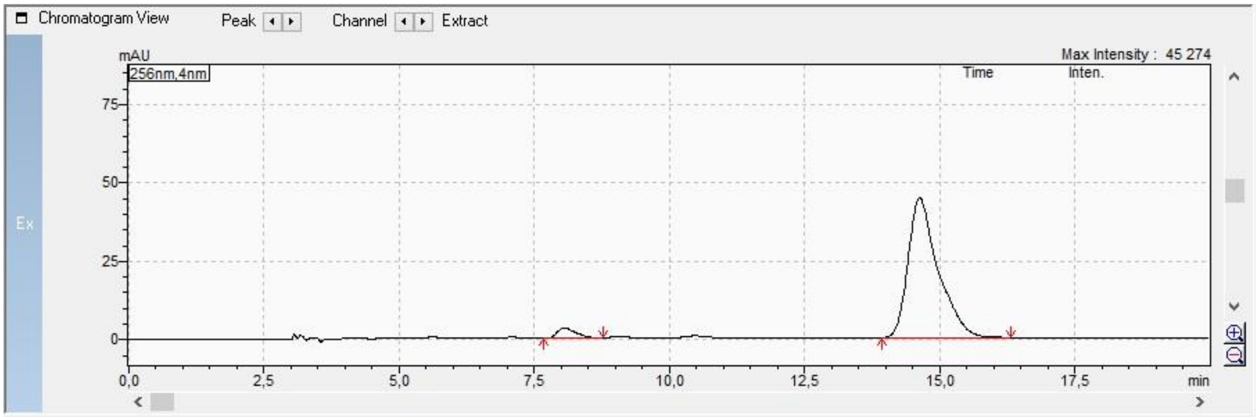

Results View - Peak Table

Peak Table | Compound | Group | Calibration Curve

Peak#	Ret. Time	Area	Height	Peak Start	Peak End	Mark	Conc.	Unit	Area%
1	8,061	80461	3323	7,680	8,768	M	4,470		4,470
2	14,633	1719625	44619	13,931	16,309	M	95,530		95,530
Total		1800086	47943				100,000		100,000

(R)-(1,2,3,5-Tetrahydrobenzo[e][1,4]oxazepin-3-yl)methanol **(3a)**

Racemate:

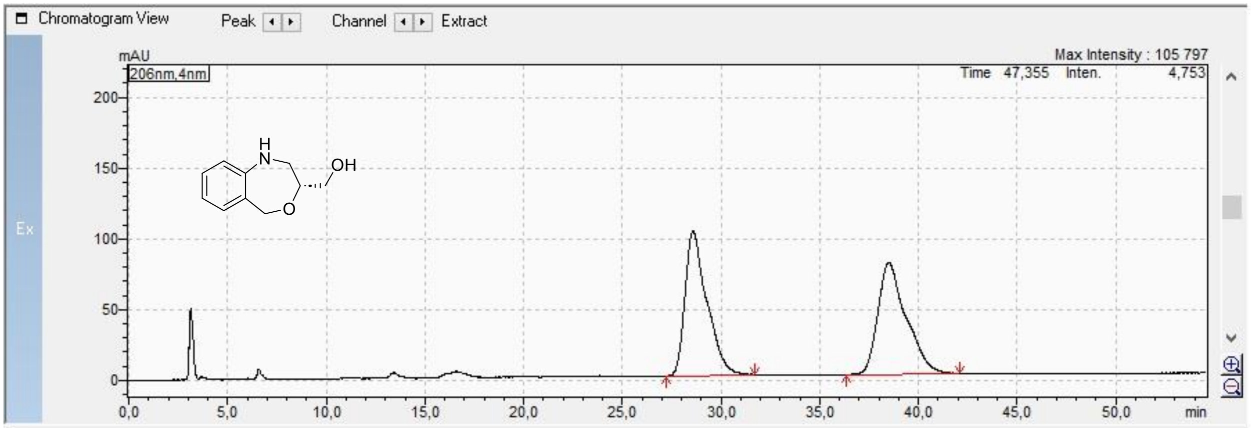

Results View - Peak Table

Peak Table | Compound | Group | Calibration Curve

Peak#	Ret. Time	Area	Height	Peak Start	Peak End	Mark	Conc.	Unit	Area%
1	28,583	7812050	102238	27,221	31,712	M	49,880		49,880
2	38,484	7849606	78501	36,341	42,091	M	50,120		50,120
Total		15661657	180739				100,000		100,000

Chiral: 92% *e.e.*

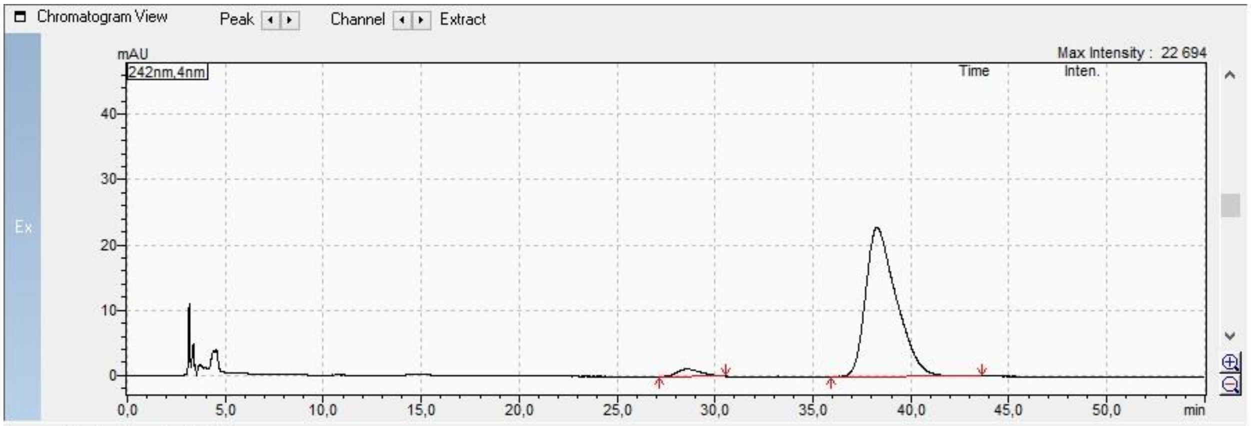

Results View - Peak Table

Peak Table | Compound | Group | Calibration Curve

Peak#	Ret. Time	Area	Height	Peak Start	Peak End	Mark	Conc.	Unit	Area%
1	28,532	98330	1112	27,147	30,549	M	3,826		3,826
2	38,273	2471881	22831	35,915	43,627	M	96,174		96,174
Total		2570211	23943				100,000		100,000

(R)-(1-Benzyl-1,2,3,5-tetrahydrobenzo[e][1,4]oxazepin-3-yl)methyl 4-methylbenzenesulfonate ***(4a)***

Racemate:

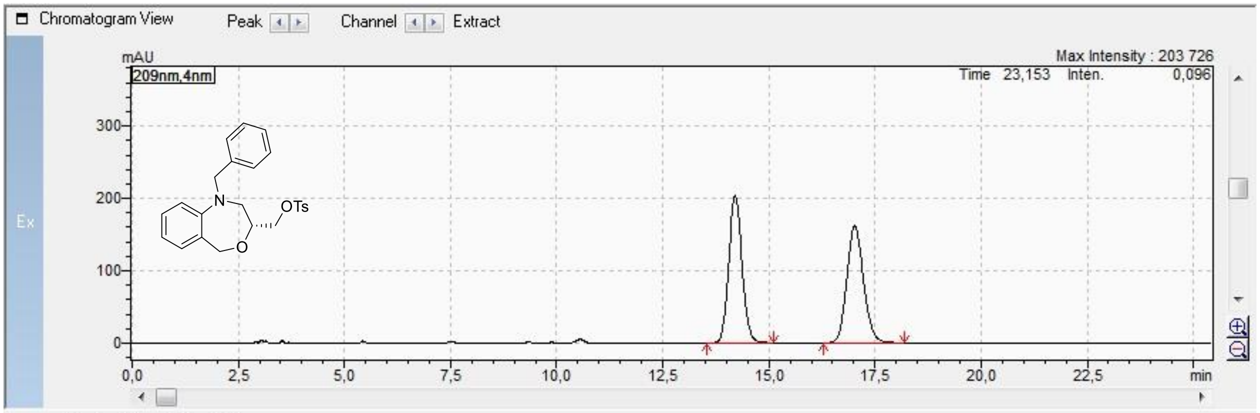

Results View - Peak Table

Peak Table | Compound | Group | Calibration Curve

Peak#	Ret. Time	Conc.	Area	Height	Similarity Index	Mark	Peak Start	Peak End	Area%
1	14,206	49,978	4429832	203517	0,000000	M	13,536	15,115	49,978
2	17,023	50,022	4433764	161728	0,000000	M	16,288	18,197	50,022
Total		100,000	8863597	365245					100,000

Chiral: 91% *e.e.*

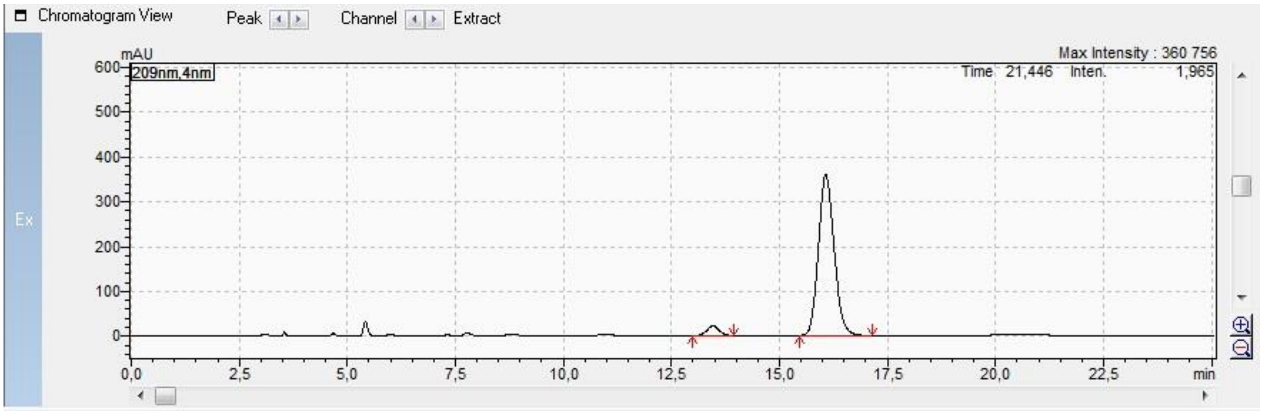

Results View - Peak Table

Peak Table | Compound | Group | Calibration Curve

Peak#	Ret. Time	Conc.	Area	Height	Similarity Index	Mark	Peak Start	Peak End	Area%
1	13,458	4,529	431474	21511	0,000000	M	12,981	13,941	4,529
2	16,073	95,471	9094670	358942	0,000000	M	15,467	17,152	95,471
Total		100,000	9526144	380452					100,000

(R)-3-(Azidomethyl)-1-benzyl-1,2,3,5-tetrahydrobenzo[e][1,4]oxazepine ***(5a)***

Racemate:

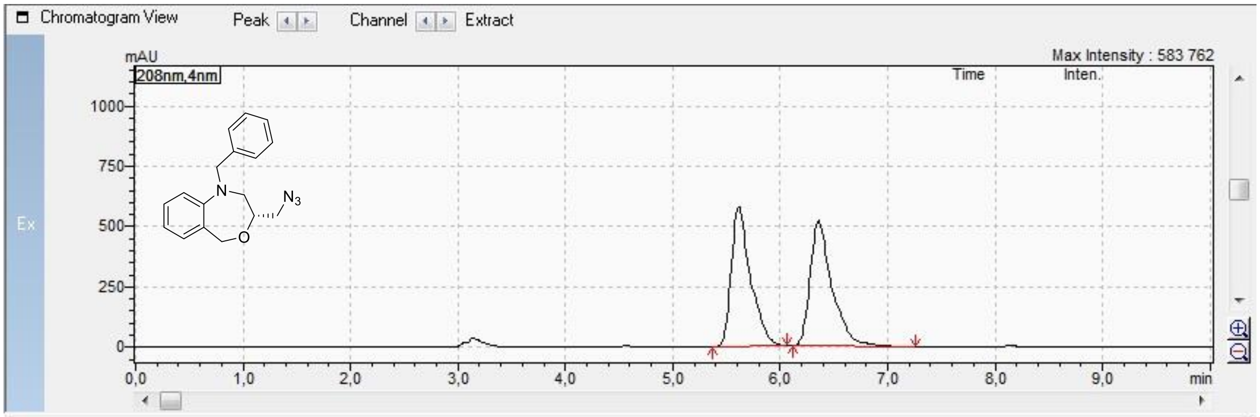

Results View - Peak Table

Peak Table | Compound | Group | Calibration Curve

Peak#	Ret. Time	Conc.	Area	Height	Similarity Index	Mark	Peak Start	Peak End	Area%
1	5,615	49,333	7198584	579694	0,000000	M	5,376	6,059	49,333
2	6,358	50,667	7393182	519755	0,000000	M	6,112	7,253	50,667
Total		100,000	14591766	1099449					100,000

Chiral: 92% *e.e.*

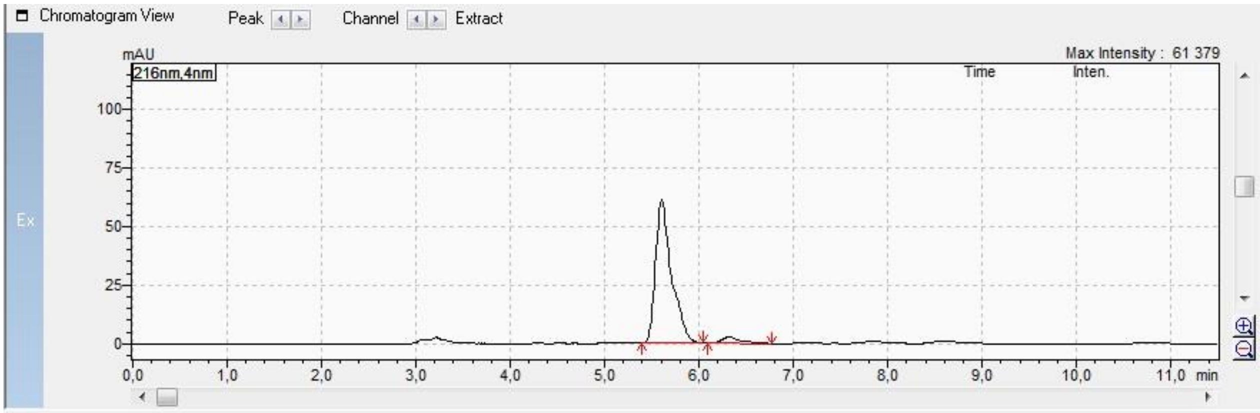

Results View - Peak Table

Peak Table | Compound | Group | Calibration Curve

Peak#	Ret. Time	Conc.	Area	Height	Similarity Index	Mark	Peak Start	Peak End	Area%
1	5,605	95,935	721266	61040	0,000000	M	5,397	6,048	95,935
2	6,318	4,065	30564	2408	0,000000	M	6,091	6,773	4,065
Total		100,000	751830	63448					100,000

(R)-1-Benzyl-3-(chloromethyl)-1,2,3,5-tetrahydrobenzo[e][1,4]oxazepine ***(6a)***

Racemate:

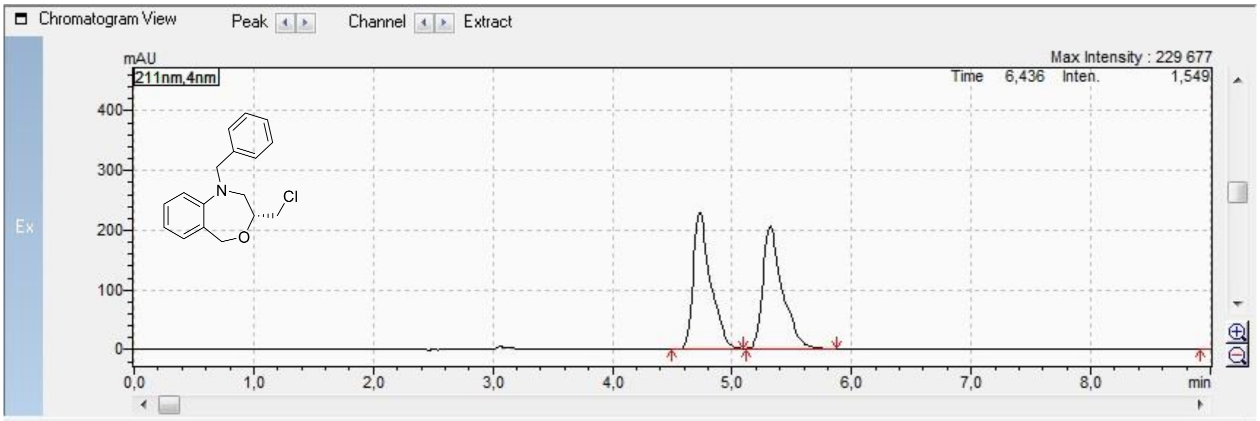

Results View - Peak Table

Peak Table | Compound | Group | Calibration Curve

Peak#	Ret. Time	Conc.	Area	Height	Similarity Index	Mark	Peak Start	Peak End	Area%
1	4,731	49,544	2294138	228062	0,000000	M	4,491	5,088	49,544
2	5,320	50,397	2333639	205523	0,000000	M	5,120	5,877	50,397
3	9,113	0,059	2718	68	0,000000		8,917	9,120	0,059
Total		100,000	4630496	433654					100,000

Chiral: 92% *e.e.*

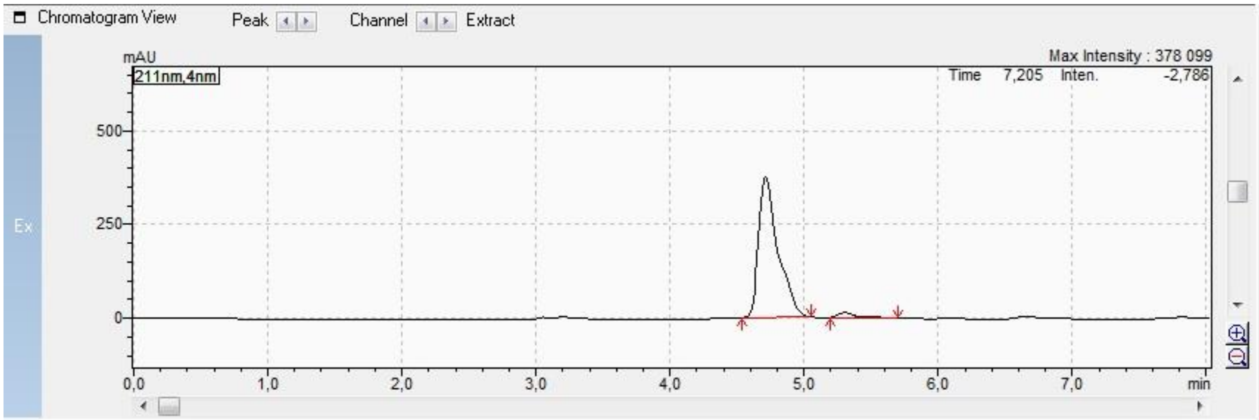

Results View - Peak Table

Peak Table | Compound | Group | Calibration Curve

Peak#	Ret. Time	Conc.	Area	Height	Similarity Index	Mark	Peak Start	Peak End	Area%
1	4,717	96,375	3736088	375636	0,000000	M	4,533	5,056	96,375
2	5,301	3,625	140541	13718	0,000000	M	5,195	5,696	3,625
Total		100,000	3876629	389355					100,000
